# N‐Heterocyclic Carbene Catalyzed Photoenolization/Diels–Alder Reaction of Acid Fluorides

**DOI:** 10.1002/anie.201914456

**Published:** 2020-01-09

**Authors:** Andreas Mavroskoufis, Keerthana Rajes, Paul Golz, Arush Agrawal, Vincent Ruß, Jan P. Götze, Matthew N. Hopkinson

**Affiliations:** ^1^ Institute of Chemistry and Biochemistry Freie Universität Berlin Takustraße 3 14195 Berlin Germany

**Keywords:** acid fluorides, N-heterocyclic carbenes, organocatalysis, photochemistry, photoenolization

## Abstract

The combination of light activation and N‐heterocyclic carbene (NHC) organocatalysis has enabled the use of acid fluorides as substrates in a UVA‐light‐mediated photochemical transformation previously observed only with aromatic aldehydes and ketones. Stoichiometric studies and TD‐DFT calculations support a mechanism involving the photoactivation of an *ortho*‐toluoyl azolium intermediate, which exhibits “ketone‐like” photochemical reactivity under UVA irradiation. Using this photo‐NHC catalysis approach, a novel photoenolization/Diels–Alder (PEDA) process was developed that leads to diverse isochroman‐1‐one derivatives.

## Graphical abstract

**Photo‐NHC catalysis** is introduced as a strategy for engaging hitherto unsuitable carbonyl substrates at the carboxylic acid oxidation level in photochemical transformations of aldehydes and ketones. This approach enables an NHC‐catalyzed photoenolization/Diels–Alder (PEDA) reaction of acid fluorides to isochroman‐1‐ones. The NHC likely acts as a “transient aryl group” that temporarily changes the photochemical properties of the carbonyl substrate.

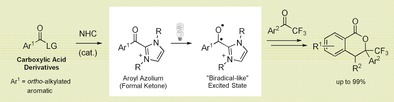

Recent years have seen a resurgence of interest in photochemical activation as a means of accessing new reactivity modes in organic synthesis. In contrast to thermally activated processes, photochemical reactions proceed via excited‐state species and duly exhibit dramatically different reactivity and selectivity. Reactions involving the activation of aromatic aldehydes and ketones with UVA light are among the most widely studied photochemical transformations. Upon excitation, the biradical‐like (n,π*) states of these compounds undergo a range of synthetically important processes, such as Norrish fragmentations, Yang cyclizations, and Paternò–Büchi cycloadditions.^1^ While such reactions are well established for aldehydes and ketones, analogous transformations with substrates at the carboxylic acid oxidation level are scarce. These compounds typically absorb light at shorter wavelengths than the corresponding ketones while many populate lowest‐energy (π,π*) excited states with inherently different photochemical reactivity.^2^, ^3^

Inspired by the recent successes achieved by combining light activation with other catalysis modes,^4^, ^5^ we wondered whether the scope of carbonyl photochemistry could be expanded by merging light activation with N‐heterocyclic carbene (NHC) organocatalysis.^6^ An overview of our proposed photo‐NHC catalysis concept is shown in Scheme 1. As demonstrated in several elegant processes, NHCs can react with activated aryl carboxylic acid derivatives to afford benzoyl azolium intermediates. These species are formally ketones and could be expected to populate excited states comparable to those of structurally related benzophenone derivatives upon light irradiation. Furthermore, in providing a second aromatic system for π‐conjugation, the NHC can influence the absorption characteristics of the carbonyl function, enabling excitation at longer wavelengths compared with the parent carboxylic acid derivative. Following a light‐mediated process of the type typically observed with aromatic ketones and elimination of the NHC organocatalyst, new classes of product for carbonyl photochemistry would be provided.

**Scheme 1:**
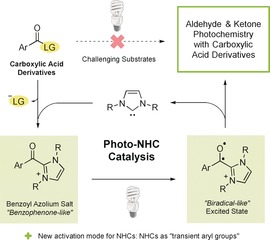
Overview of photo‐NHC catalysis. LG=leaving group.

The photo‐NHC concept represents a new catalysis mode for NHCs. These compounds most often facilitate umpolung reactions of aldehydes while transformations involving acyl azolium salts or enolates have also been developed.^7^ Most processes are considered to proceed through polar mechanisms; however, recent studies have revealed new radical activation modes, which exploit the ability of NHCs to stabilize unpaired electron density.^8^, ^9^, ^10^ In photo‐NHC catalysis, the role of the NHC is again different. In temporarily changing the absorption profile and photochemical reactivity of a carbonyl group during the catalytic cycle, the NHC can be considered as a “transient aryl group” that enables hitherto unsuitable substrates to engage in photochemical transformations. Moreover, as the catalyst remains bound to the substrate throughout the cycle, enantioselective variants could be potentially realized by using chiral NHCs.

We initially sought to validate the photo‐NHC catalysis concept by investigating an independently synthesized stoichiometric acyl azolium salt. As a proof‐of‐concept transformation, a photoenolization process was selected starting from *ortho*‐toluoyl imidazolium salt **1**.^11^ Upon irradiation with UVA light, excitation of the carbonyl group followed by intramolecular 1,5‐hydrogen atom transfer (HAT) would lead to a hydroxy‐*o*‐quinodimethane (*o*‐QDM) species. Compound **1** was thus prepared (see the Supporting Information) and subjected to irradiation from UVA LEDs (λ_max_=365 nm) in a mixture of degassed CD_3_CN and CD_3_CO_2_D (9:1). After 16 h, we were delighted to observe extensive incorporation of deuterium (68 %) at the *o*‐methyl group consistent with deuteration of an *o*‐QDM intermediate while no reaction was observed without light (Scheme 2 a). This result demonstrates that acyl azolium species are capable of both absorbing light at wavelengths similar to other aromatic ketones and of exhibiting analogous photochemical reactivity. In a further experiment, a photoenolization/Diels–Alder (PEDA) process leading to isochroman‐1‐ones was investigated by reacting **1** with ketone **2 a**. Analogous non‐NHC‐catalyzed reactions affording the corresponding hemiacetals have been described for aromatic aldehydes and ketones.^12^, ^13^ After 16 h of irradiation in degassed MeCN, clean conversion into the desired product **3 aa** was observed in 62 % NMR yield while, as for the deuteration experiment, no reaction occurred without light (Scheme 2 b).

**Scheme 2:**
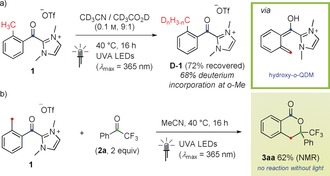
Validation of the photo‐NHC catalysis concept with acyl azolium salt **1**. a) Deuteration of the *o*‐benzylic position via photoenolization. b) PEDA reaction with ketone **2 a**. Tf=triflyl.

Annulation reactions are among the most important classes of NHC‐catalyzed transformation.^14^ As such, novel reactivity modes for NHCs that open up new strategies for annulation reactions are especially desirable. The proposed PEDA reaction has some attractive features in comparison to alternative NHC‐catalyzed routes to isochroman‐1‐ones **3**. Previously published processes have employed either *o*‐CH_2_Br‐substituted benzaldehydes^15^ or *o*‐CH_2_SiMe_3_‐substituted esters,^16^ which can be laborious to prepare. The only example of directly using simple *o*‐toluic acid derivatives was reported recently by Li, Yao, and co‐workers.^17^, ^18^ This non‐photochemical process, however, was successful only for highly electron‐deficient substrates whose *o*‐benzylic positions are sufficiently acidic to be deprotonated by an amine base.

We next turned our attention to the development of an NHC‐catalyzed PEDA process. After evaluating different leaving groups, *o*‐toluoyl fluorides **4** 19 were identified as the most efficient substrates with ketones **2**. Optimization of the reaction conditions resulted in an efficient system featuring the simple NHC precursor 1,3‐dimethylimidazolium triflate (IMe⋅HOTf, 20 mol %) and Cs_2_CO_3_ (2 equiv) in degassed MeCN. After 16 h under irradiation from UVA LEDs, **3 aa** was isolated in 84 % yield while control reactions without the NHC, base, or light led only to trace amounts of product (see the Supporting Information).^20^

The scope of the PEDA reaction was tested with a range of substrates **4** and **2** (Scheme 3). As shown for the methyl‐substituted acid fluorides **4 b**–**4 e**, the yield of products **3** was found to be dependent on the site of aromatic substitution. While the 5‐, 6‐, and 7‐methylisochroman‐1‐ones were generated in good yields (65–86 %), the PEDA process with sterically demanding 2,6‐dimethylbenzoyl fluoride (**4 e**) was comparatively inefficient (17 %). Halogen substituents amenable to subsequent cross‐coupling were well tolerated, and the corresponding isochroman‐1‐ones **3 fa**–**3 na** were obtained in excellent yields of up to 95 %. The successful application of the *o*‐ethyl‐ and *o*‐benzyl‐substituted acid fluorides **4 o** (93 %, 2.6:1 d.r.) and **4 p** (48 %, 10:1 d.r.) is particularly noteworthy as substrates with substituted *o*‐benzyl positions were not employed in previously reported NHC‐catalyzed syntheses of isochroman‐1‐ones.^15^, ^16^, ^17^ A range of electronically diverse dienophiles **2** were tolerated, including CF_3_‐ (**2 b**, **2 c**) and MeO‐substituted (**2 d**) derivatives. Unfortunately, other classes of non‐enolizable ketones, such as isatins or 2‐ketoesters, could not be employed, potentially because of competitive absorption of UVA light by these substrates (see the Supporting Information).

**Scheme 3:**
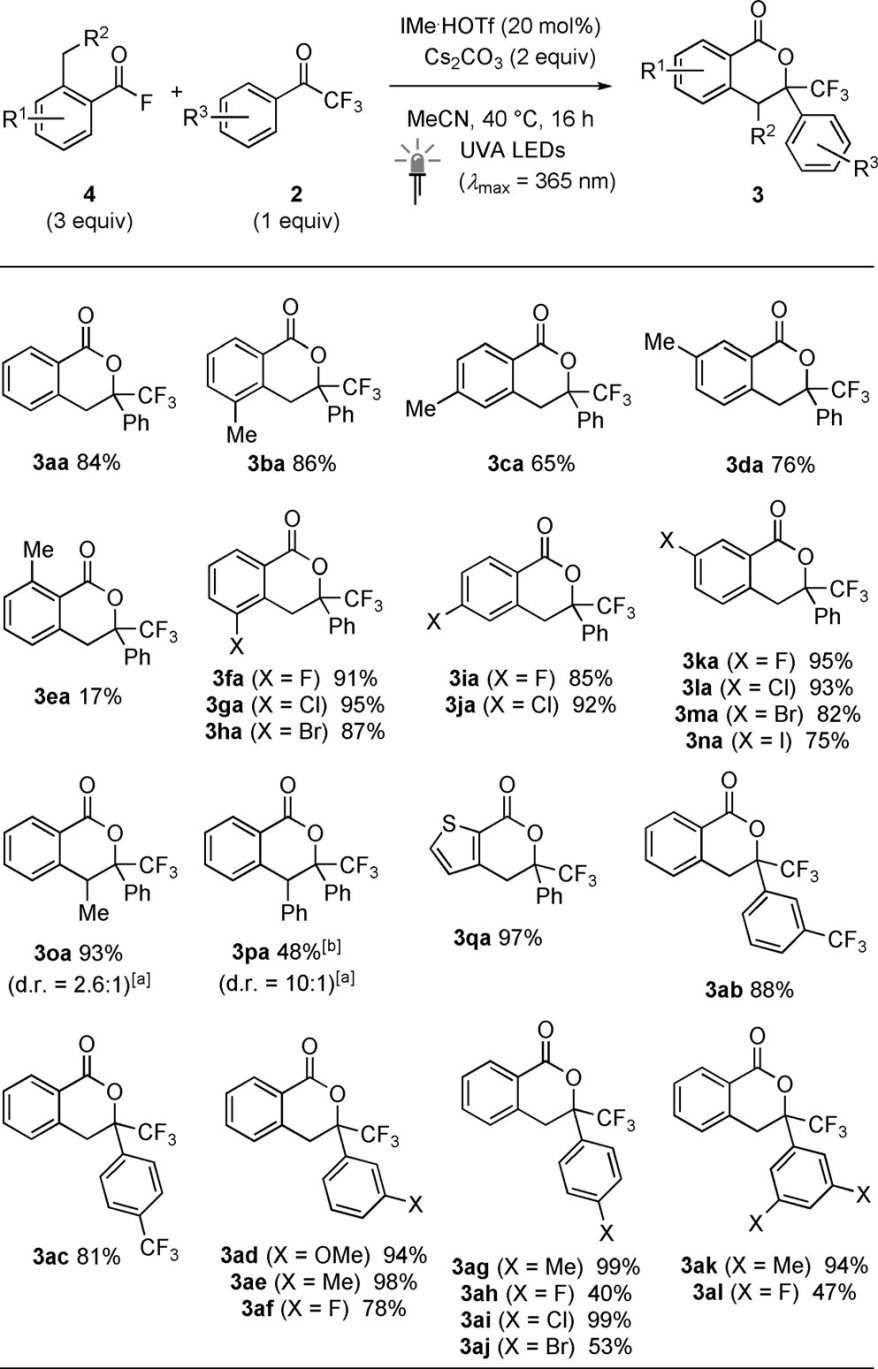
Scope of the NHC‐catalyzed PEDA reaction. Conditions: **4 a**–**4 p** (0.90 mmol), **2 a**–**2 l** (0.30 mmol), IMe⋅HOTf (0.060 mmol), Cs_2_CO_3_ (0.60 mmol), degassed MeCN (3 mL), 40 °C, 16 h, UVA LEDs. Yields of isolated products. [a] Diastereomeric ratios (d.r.) determined by ^1^H NMR analysis of the crude reaction mixture. [b] Major diastereomer isolated.

A mechanistic proposal consistent with PEDA reactions of ketones is shown in Scheme 4.^11^, ^12^, ^13^ Upon deprotonation of the NHC precursor with Cs_2_CO_3_, the imidazolylidene IMe reacts with acid fluoride **4**, releasing F^−^ and generating the *o*‐toluoyl azolium intermediate **A**.^19^ This benzophenone‐like species is excited under UVA irradiation affording, after intersystem crossing (isc), a triplet excited state **T_1_(A)**.^21^ Fast 1,5‐HAT from the *o*‐benzylic position to the radical‐like carbonyl oxygen atom gives rise to the triplet dienol biradical **T_1_(B)**. Rotation of this species before relaxation leads to the ground‐state *o*‐QDM (*E*)‐**B**, which can react with the dienophile **2** in a cycloaddition process. This reaction could feasibly occur in a concerted Diels–Alder fashion or, as shown in Scheme 4, through a two‐step sequence involving intermediate **C**. Finally, elimination of the NHC from cycloadduct **D** in the presence of the base releases the product **3** and completes the catalytic cycle.

**Scheme 4:**
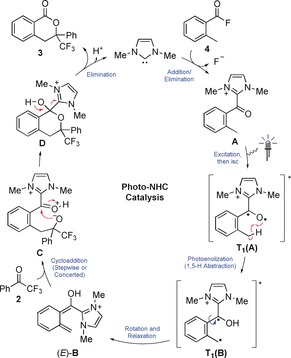
Proposed reaction mechanism.

Time‐dependent DFT calculations ((TD‐)CAM‐B3LYP/6‐311G**)^22^ on *o*‐toluoyl azolium intermediate **A** provide support for the above mechanism. Irradiation of this species in the UVA region results in a triplet excited state (**T_1_(A)**) with significant unpaired electron density at the oxygen atom (Figure 1). 1,5‐HAT proceeds readily from this species, resulting in **T_1_(B)**. Conversely, excitation of acid fluoride **4 a** occurs only at shorter wavelengths, with the analogous HAT from the triplet state **T_1_(4 a)** being highly endergonic (see the Supporting Information). The lack of photoenolization was experimentally corroborated by subjecting **4 a** to the deuteration conditions with CD_3_CN/CD_3_CO_2_D (see Scheme 2 a). No incorporation of deuterium was observed after 16 h, highlighting the key role of the NHC in modifying the photochemistry of the carbonyl function.

**Figure 1:**
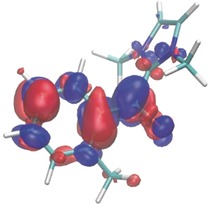
**T_1_(A)** equilibrium geometry showing the difference in DFT electronic densities between the **T_1_(A)** state and the electronic ground state at 0.002 contour value (gains in blue, losses in red upon **T_1_(A)** formation).

In conclusion, the merger of NHC organocatalysis with light activation has enabled the annulation of *o*‐toluoyl fluorides with trifluoroacetophenones. Stoichiometric studies and TD‐DFT calculations support a mechanistic scenario where addition of the NHC to the acid fluoride results in a temporary change in the absorption characteristics and photochemical reactivity of the carbonyl function during the catalytic cycle. This allows these hitherto unsuitable substrates at the carboxylic acid oxidation level to engage in a UVA‐light‐mediated photochemical reaction previously observed only with aromatic aldehydes and ketones. We believe that photo‐NHC catalysis could prove useful for expanding the scope of carbonyl photochemistry, and further investigations are underway in our laboratory.

## Conflict of interest

The authors declare no conflict of interest.

## Supporting information

As a service to our authors and readers, this journal provides supporting information supplied by the authors. Such materials are peer reviewed and may be re‐organized for online delivery, but are not copy‐edited or typeset. Technical support issues arising from supporting information (other than missing files) should be addressed to the authors.

Supplementary
