# Supplementary material for: N‐Heterocyclic Carbene Catalyzed Photoenolization/Diels–Alder Reaction of Acid Fluorides
Source: Angew Chem Int Ed Engl. 2020 Jan 9;59(8):3190–4. doi: 10.1002/anie.201914456 (PMC7027522; doi:10.1002/anie.201914456)
Supplement: Supplementary file 1 — Supplementary [file ANIE-59-3190-s001.pdf]

Angewandte Chemie
Eine Zeitschrift der Gesellschaft Deutscher Chemiker

Supporting Information

N-Heterocyclic Carbene Catalyzed Photoenolization/Diels–Alder Reaction of Acid Fluorides

*Andreas Mavroskoufis, Keerthana Rajes, Paul Golz, Arush Agrawal, Vincent Ruß, Jan P. Götze, and Matthew N. Hopkinson**

anie_201914456_sm_miscellaneous_information.pdf

Contents

1 General Information **S2**

2 Synthesis and Stoichiometric Studies of *ortho*-Toluoyl Imidazolium Salt 1 **S3**
2.1 Synthesis of *ortho*-Toluoyl Imidazolium Salt 1 . . . . S3
2.2 Deuteration of *ortho*-Toluoyl Imidazolium Salt 1 . . . . S5
2.3 PEDA Reaction of *ortho*-Toluoyl Imidazolium Salt 1 . . . . S7

3 Synthesis of Starting Materials and Reagents **S8**
3.1 Synthesis of Acid Fluorides 4 . . . . S8
3.2 Characterization Data of Aryl Fluorides 4 . . . . S9
3.3 Preparation of IMe·HOTf Stock Solution S17

4 Optimization of the NHC-catalyzed PEDA Reaction **S18**
4.1 Optimization Table S18
4.2 Control Reactions S18
4.3 Screening of NHC-precatalysts S19

5 Scope of the NHC-catalyzed PEDA Reaction **S20**
5.1 NHC-catalyzed PEDA Reaction S20
5.2 Characterization Data of Isochroman-1-ones 3 . . . . S21
5.3 Limitations of the NHC-catalyzed PEDA Reaction S36

6 UV-Vis Studies **S37**

7 Computational Studies **S42**

8 References **S43**

9 NMR Spectra of Novel Compounds **S44**

1 General Information

All air- and moisture-sensitive reactions were performed in heat-gun-dried glassware under an atmosphere of argon. Anhydrous solvents were either purchased (J&K Scientific, Acros Organics) or provided by a solvent purification system MB-SPS-800 (MBraun). Triethylamine was refluxed over potassium hydroxide, distilled and stored over 3Å molecular sieves under argon. Cesium carbonate was dried at 200°C under vacuum for 72 h and stored under argon. Solvents for extraction, crystallization and flash column chromatography were purchased in technical grade and distilled under reduced pressure prior to use. All other commercially purchased starting materials and reagents were used without further purification.

Optimization and screening reactions were performed on a 0.1 mmol scale under irradiation with a UV-LED strip from Luxalight (365 nm, IP64) purchased from LEDTuning (Type: LS24UV240X3528PLX) mounted to the inside of a water-cooled aluminium cylinder. The synthesis of isochroman-1-ones **3** was performed on a 0.3 mmol scale under irradiation with a UV-LED chip by MERUEM TOPSION (365-370 nm, 20 W) mounted to an aluminium-heat sink. Reaction flasks were placed ~2 cm away from the light source while the internal temperature did not exceed 40° C.

Thin-layer chromatography was performed on silica gel coated aluminium plates ALUGRAM® Xtra SIL G/UV_{254} (Macherey-Nagel). Product spots were detected by UV light (254 nm) or as permanganate stains. Flash column chromatography was performed with silica gel 60 M (0.040–0.063 mm, 230–400 mesh, Macherey-Nagel).

NMR spectra were recorded at ambient temperature on JEOL (ECX 400, ECP 500, ECZ 600) and Bruker (AVANCE 700) spectrometers. Chemical shifts (δ) are reported in parts per million relative to tetramethylsilane. Proton and carbon spectra are referenced to the residual solvent signal of chloroform (^{1}H: δ 7.26 ppm; ^{13}C: δ 77.16 ppm), fluorine spectra are not calibrated. Coupling constants (J) are given in hertz. Multiplicity is denoted as follows: s (singlet), d (doublet), t (triplet), q (quartet), m (multiplet). NMR yields of crude mixtures were determined by adding dibromomethane as an internal standard. Diastereomeric ratios were determined by proton NMR on the crude reaction mixture.

High-resolution mass spectra were measured with an Agilent (6210 ESI-TOF; 4 μL/min, 1.0 bar, 4 kV) or Varian MAT (modified MAT 711; 80 eV) instrument. Infrared spectra were measured with a JASCO spectrometer (FT/IR-4100) equipped with an ATR unit and only diagnostic absorption bands are reported. Ultraviolet-visible measurements were carried out on a Varian Cary 50 Bio Photospectrometer equipped with a xenon lamp.

2 Synthesis and Stoichiometric Studies of *ortho*-Toluoyl Imidazolium Salt 1

2.1 Synthesis of *ortho*-Toluoyl Imidazolium Salt 1

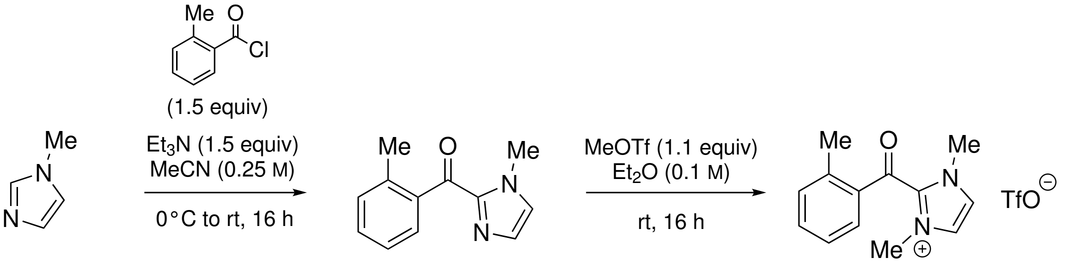

Scheme S1: Two-step synthesis of *ortho*-toluoyl imidazolium salt **1**.

(1-Methyl-1*H*-imidazol-2-yl)(*o*-tolyl)methanone (S1)

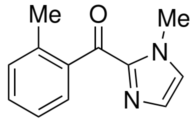

S1

To a solution of 1-methylimidazole (821 mg, 790 μL, 10.0 mmol, 1 equiv) and *o*-toluoyl chloride (2.32 g, 1.96 mL, 15.0 mmol, 1.5 equiv) in anhydrous acetonitrile (40 mL, 0.25 M) was added triethylamine (1.52 g, 2.09 mL, 15.0 mmol, 1.5 equiv) dropwise at 0° C. The reaction mixture was allowed to slowly warm up to rt and stirred overnight. After addition of water (25 mL), the phases were separated and the aqueous layer was extracted with diethyl ether (3 x 25 mL). The combined organic phases were washed with brine (40 mL), dried over sodium sulfate and concentrated under vacuum. Purification by column chromatography on silica gel (*n*-pentane/Et_2O 1:2) afforded **S1** as an off-white solid (1.90 g, 9.50 mmol, 95%).

R$_f$: 0.30 (*n*-pentane/Et_2O 1:2); ^{1}**H-NMR** (400 MHz, $CDCl_3$): δ 7.62 (dd, J = 7.9, 1.6 Hz, 1H), 7.37 (td, J = 7.4, 1.5 Hz, 1H), 7.28–7.26 (m, 1H), 7.26–7.24 (m, 1H), 7.19 (s, 1H), 7.10 (s, 1H), 4.12 (s, 3H), 2.41 (s, 3H) ppm.

The characterization data agree with literature precedents.[1]

1,3-Dimethyl-2-(2-methylbenzoyl)-1*H*-imidazol-3-ium triflate (1)

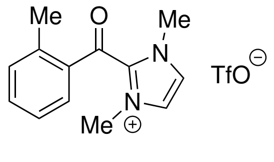

1

To a solution of *ortho*-toluoyl imidazole **S1** (500 mg, 2.50 mmol, 1 equiv) in anhydrous diethyl ether (25 mL, 0.1 M) was added methyl triflate (451 mg, 311 μL, 2.75 mmol, 1.1 equiv) at rt and the reaction mixture was stirred overnight. The resulting white precipitate was filtered off and washed with diethyl ether (3 x 10 mL). Drying under vacuum afforded **1** as a white solid (879 mg, 2.41 mmol, 97%).

^{1}**H-NMR** (600 MHz, $CDCl_3$): δ 7.78 (s, 2H), 7.68–7.61 (m, 2H), 7.48–7.41 (m, 2H), 3.83 (s, 6H), 2.59 (s, 3H) ppm; ^{13}**C-NMR** (151 MHz, $CDCl_3$) δ 181.3, 141.0, 139.6, 135.4, 133.6, 133.1, 131.9, 127.6, 125.8, 120.8 (q, J = 320.3 Hz), 37.6, 20.9 ppm; ^{19}**F-NMR** (565 MHz, $CDCl_3$): δ –78.4 ppm; **HRMS-ESI**: m/z calculated for $[C_{13}H_{15}N_2O]^+$ ($[M–TfO]^+$) 215.1179, found 215.1189; **IR** (ATR): $\tilde{\nu}$ = 1681 (m) cm^{-1} (C=O).

2.2 Deuteration of *ortho*-Toluoyl Imidazolium Salt 1

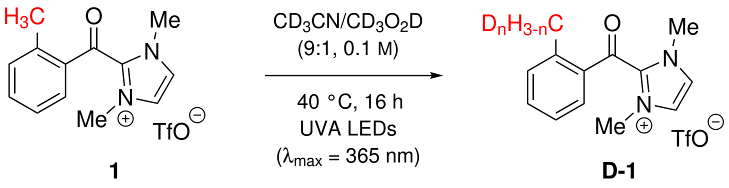

Scheme S2: Deuteration experiment of *ortho*-toluoyl imidazolium salt **1**.

Entry	Comment	Recovered SM (NMR)	Deuteration (NMR)
1	–	72 %	68 %
2	without Light	100 %	0 %
3	**4a** instead of **1**	100 %	0 %

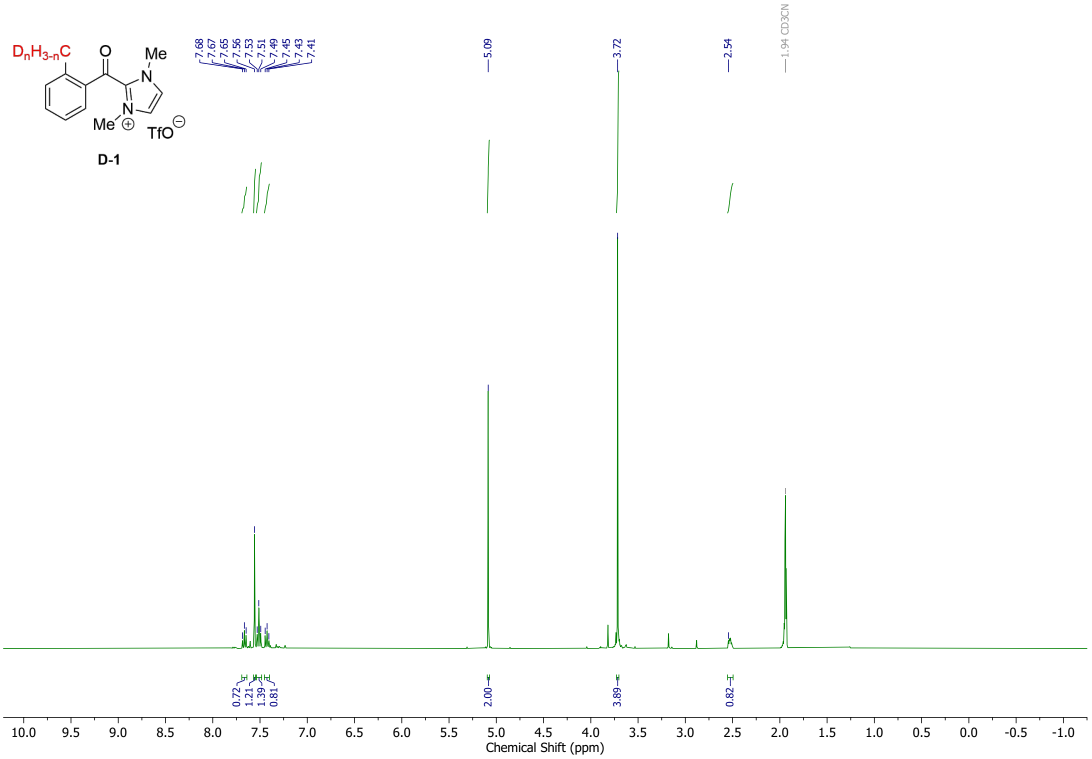

Figure S1: Proton spectrum of **1** in CD_3CN/CD_3CO_2D (9:1, 0.1 M) after irradiation at 365 nm for 16 h.

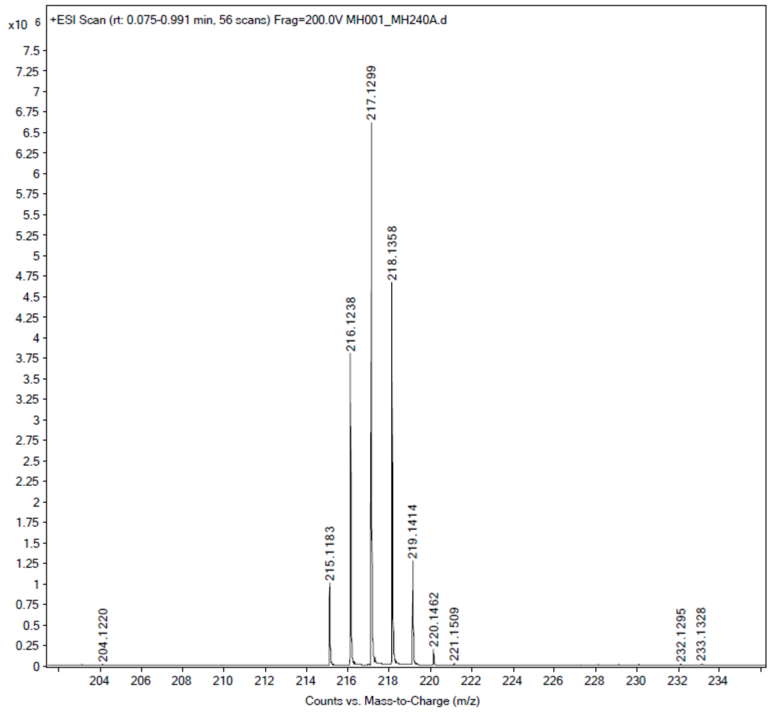

Figure S2: HRMS-ESI spectrum of **1** in CD_3CN/CD_3CO_2D (9:1, 0.1 M) after irradiation at 365 nm for 16 h.

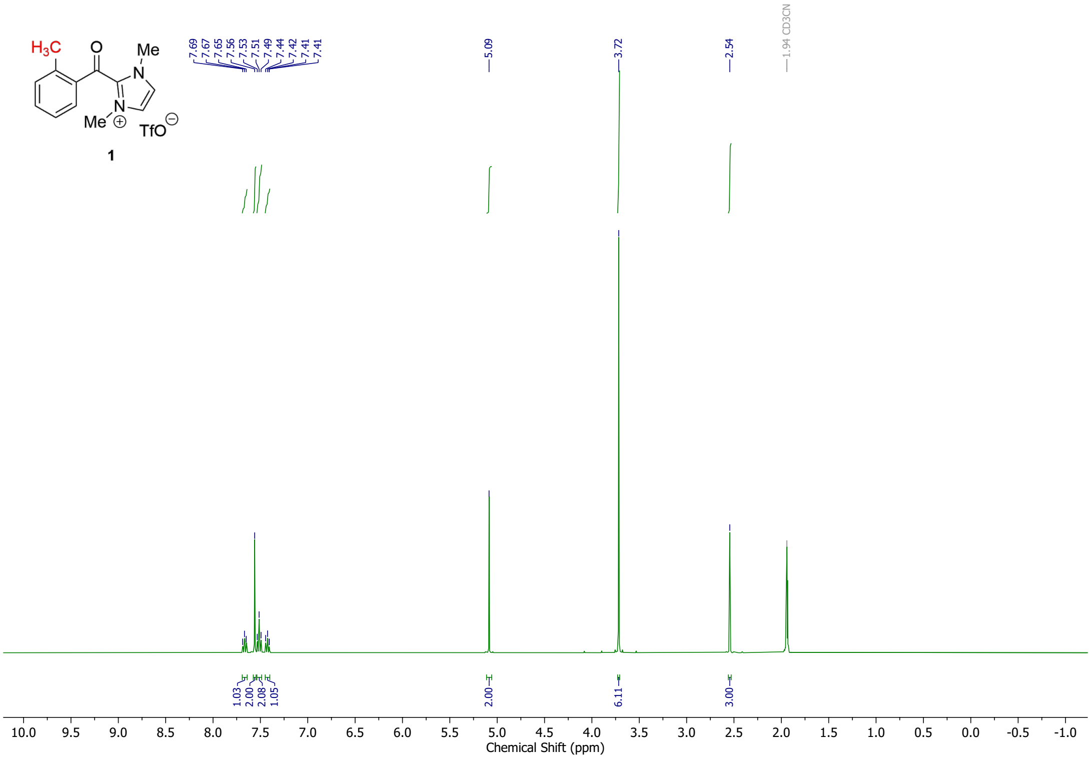

Figure S3: Proton spectrum of **1** in CD_3CN/CD_3CO_2D (9:1, 0.1 M) after 16 h in the dark.

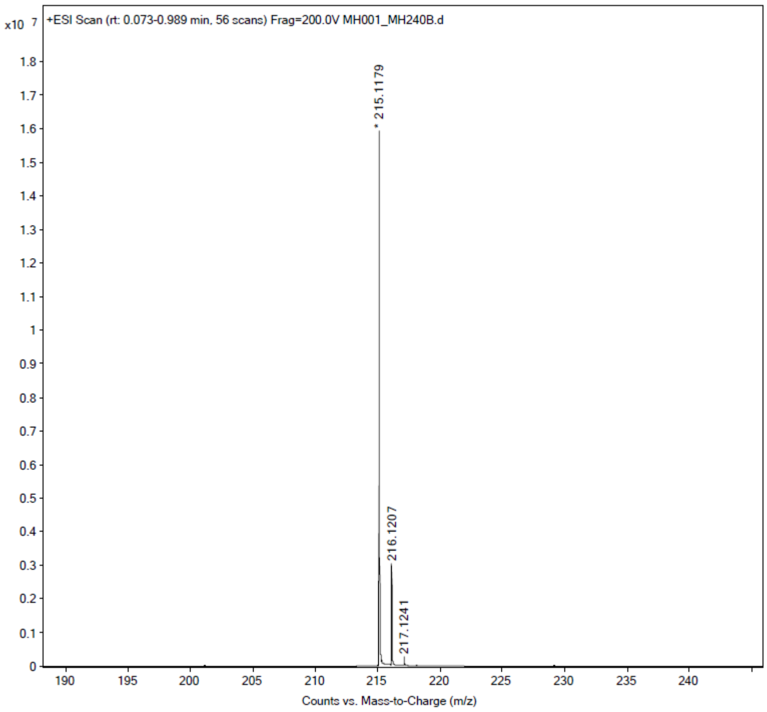

Figure S4: HRMS-ESI spectrum of **1** in CD_3CN/CD_3CO_2D (9:1, 0.1 M) after 16 h in the dark.

2.3 PEDA Reaction of *ortho*-Toluoyl Imidazolium Salt 1

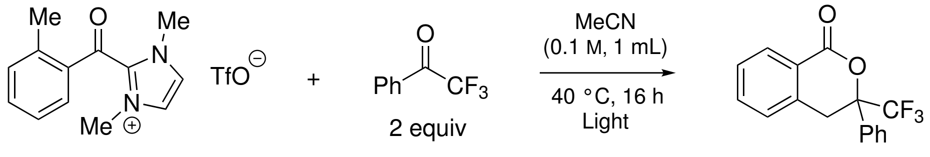

1 **2a** **3aa**

Entry	Light Source	Notes	Yield (NMR)
1	UV LEDs (365 nm)	–	**3aa** (62%)
2	UV LEDs (365 nm)	1 equiv of **2a**	**3aa** (39%)
3	Blue LEDs (455 nm)	–	**3aa** (10%)
4	UV LEDs (365 nm)	no degassing	**3aa** (24%)
5	–	no light	– (**1**, 63%)

3 Synthesis of Starting Materials and Reagents

3.1 Synthesis of Acid Fluorides 4

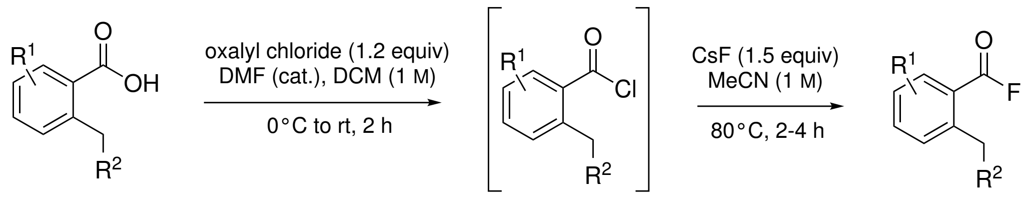

4

Scheme S3: Two-step synthesis of acid fluorides **4**.

General Procedure A: In a procedure modified from Malapit and co-workers,[2] the benzoic acid (5.00 mmol, 1 equiv) was dissolved or suspended in anhydrous dichloromethane (5 mL, 1 M) and five drops of anhydrous *N,N*-dimethylformamide were added. Upon cooling to 0° C, oxalyl chloride (762 mg, 515 μL, 6.00 mmol, 1.2 equiv) was cautiously added dropwise. The reaction mixture was allowed to warm up to rt and stirred for 2 h. Volatiles were removed under vacuum and the crude benzoyl chloride was dissolved in anhydrous acetonitrile (5 mL, 1 M). Cesium fluoride (1.14 g, 7.50 mmol, 1.5 equiv) was added and the mixture was stirred for 2-4 h (monitored by TLC) at 80° C. After completion, the reaction mixture was filtered, the filtration residue washed with *n*-pentane (3 x 5 mL) and the combined organic solutions concentrated under vacuum. The resulting crude product was purified by column chromatography on silica gel.

3.2 Characterization Data of Aryl Fluorides 4

2-Methylbenzoyl fluoride (4a)

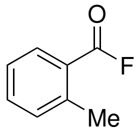

4a

According to General Procedure A, the reaction of *o*-toluoyl chloride (3.10 g, 2.61 mL, 20.0 mmol, 1 equiv) and cesium fluoride (4.56 g, 30.0 mmol, 1.5 equiv) in anhydrous acetonitrile (20 mL, 1 M) afforded after column chromatography (*n*-pentane) 2-methylbenzoyl fluoride **4a** as a colourless liquid (2.11 g, 15.3 mmol, 76%).

$\mathbf{R}_f$: 0.22 (*n*-pentane); ^{1}**H-NMR** (600 MHz, $CDCl_3$): δ 8.01–7.97 (m, 1H), 7.55 (td, J = 7.5, 1.5 Hz, 1H), 7.33 (t, J = 7.5 Hz, 2H), 2.65 (d, J = 2.0 Hz, 3H) ppm; ^{13}**C-NMR** (151 MHz, $CDCl_3$): δ 156.8 (d, J = 345.5 Hz), 143.8 (d, J = 7.2 Hz), 134.8, 132.6 (d, J = 1.9 Hz), 132.3 (d, J = 4.4 Hz), 126.5, 123.6 (d, J = 56.4 Hz), 22.0 ppm; ^{19}**F-NMR** (565 MHz, $CDCl_3$): δ 29.2 ppm.

The characterization data agree with literature precedents.[3]

2,3-Dimethylbenzoyl fluoride (4b)

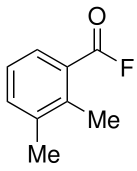

4b

The reaction of 2,3-dimethylbenzoic acid (750 mg, 5.00 mmol) following General Procedure A afforded after column chromatography (*n*-pentane) 2,3-dimethylbenzoyl fluoride **4b** as a colourless liquid (520 mg, 3.42 mmol, 68%).

$\mathbf{R}_f$: 0.16 (*n*-pentane); ^{1}**H-NMR** (500 MHz, $CDCl_3$): δ 7.82 (dd, J = 7.9, 1.4 Hz, 1H), 7.44 (d, J = 7.5 Hz, 1H), 7.21 (t, J = 7.8 Hz, 1H), 2.56 (s, 3H), 2.37 (s, 3H) ppm; ^{13}**C-NMR** (126 MHz, $CDCl_3$): δ 157.3 (d, J = 345.9 Hz), 141.8 (d, J = 7.3 Hz), 138.9 (d, J = 4.4 Hz), 136.3, 130.2 (d, J = 2.5 Hz), 125.8, 124.3 (d, J = 55.5 Hz), 20.7, 16.8 ppm; ^{19}**F-NMR** (565 MHz, $CDCl_3$): δ 32.7 ppm; **HRMS-EI**: m/z calculated for $[C_9H_9FO]^{\cdot+}$ ($[M]^{\cdot+}$) 152.0632, found 152.0638; **IR** (ATR): $\tilde{\nu}$ = 1799 (vs) cm^{-1} (C=O).

2,4-Dimethylbenzoyl fluoride (4c)

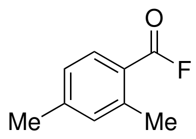

4c

The reaction of 2,4-dimethylbenzoic acid (750 mg, 5.00 mmol) following General Procedure A afforded after column chromatography (*n*-pentane) 2,4-dimethylbenzoyl fluoride **4c** as a colourless liquid (389 mg, 2.56 mmol, 51%).

R$_f$: 0.18 (*n*-pentane); ^{1}**H-NMR** (400 MHz, $CDCl_3$): δ 7.88 (d, J = 7.9 Hz, 1H), 7.16–7.10 (m, 2H), 2.61 (d, J = 0.9 Hz, 3H), 2.40 (s, 3H) ppm; ^{13}**C-NMR** (151 MHz, $CDCl_3$): δ 156.9 (d, J = 343.8 Hz), 146.0, 143.8 (d, J = 7.1 Hz), 133.1 (d, J = 4.3 Hz), 132.7 (d, J = 2.0 Hz), 127.2, 120.8 (d, J = 56.7 Hz), 21.9, 21.8 ppm; ^{19}**F-NMR** (376 MHz, $CDCl_3$): δ 28.2 ppm; **HRMS-EI**: *m/z* calculated for $[C_9H_9FO]^{\cdot+}$ ($[M]^{\cdot+}$) 152.0632, found 152.0643; **IR** (ATR): $\tilde{\nu}$ = 1806 (vs) cm^{-1} (C=O).

2,5-Dimethylbenzoyl fluoride (4d)

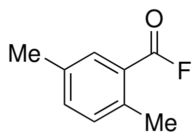

4d

The reaction of 2,5-dimethylbenzoic acid (750 mg, 5.00 mmol) following General Procedure A afforded after column chromatography (*n*-pentane) 2,5-dimethylbenzoyl fluoride **4d** as a colourless liquid (421 mg, 2.77 mmol, 55%).

R$_f$: 0.18 (*n*-pentane); ^{1}**H-NMR** (500 MHz, $CDCl_3$): δ 7.80 (s, 1H), 7.35 (d, J = 7.8 Hz, 1H), 7.22 (d, J = 7.8 Hz, 1H), 2.60 (s, 3H), 2.37 (s, 3H) ppm; ^{13}**C-NMR** (126 MHz, $CDCl_3$): δ 157.0 (d, J = 345.9 Hz), 140.6 (d, J = 6.8 Hz), 136.2, 135.6, 132.9 (d, J = 2.1 Hz), 132.2 (d, J = 4.5 Hz), 123.4 (d, J = 56.0 Hz), 21.5 (d, J = 1.6 Hz), 20.8 ppm; ^{19}**F-NMR** (376 MHz, $CDCl_3$): δ 29.1 ppm; **HRMS-EI**: *m/z* calculated for $[C_9H_9FO]^{\cdot+}$ ($[M]^{\cdot+}$) 152.0632, found 152.0638; **IR** (ATR): $\tilde{\nu}$ = 1803 (vs) cm^{-1} (C=O).

2,6-Dimethylbenzoyl fluoride (4e)

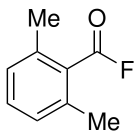

4e

The reaction of 2,6-dimethylbenzoic acid (750 mg, 5.00 mmol) following General Procedure A afforded after column chromatography (*n*-pentane) 2,6-dimethylbenzoyl fluoride **4e** as a colourless liquid (531 mg, 3.49 mmol, 70%).

R$_f$: 0.21 (*n*-pentane); ^{1}**H-NMR** (500 MHz, $CDCl_3$): δ 7.32 (t, J = 7.7 Hz, 1H), 7.12 (d, J = 7.7 Hz, 2H), 2.47 (d, J = 3.2 Hz, 6H) ppm; ^{13}**C-NMR** (126 MHz, $CDCl_3$): δ 158.6 (d, J = 354.9 Hz), 138.8, 132.0, 128.7 (d, J = 1.6 Hz), 127.0 (d, J = 54.0 Hz), 21.0 (d, J = 2.9 Hz) ppm; ^{19}**F-NMR** (376 MHz, $CDCl_3$): δ 53.9 ppm.

The characterization data agree with literature precedents.[2]

3-Fluoro-2-methylbenzoyl fluoride (4f)

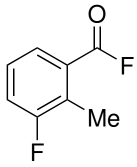

4f

The reaction of 3-fluoro-2-methylbenzoic acid (770 mg, 5.00 mmol) following General Procedure A afforded after column chromatography (*n*-pentane) 3-fluoro-2-methylbenzoyl fluoride **4f** as a colourless liquid (508 mg, 3.26 mmol, 65%).

R$_f$: 0.23 (*n*-pentane); ^{1}**H-NMR** (600 MHz, $CDCl_3$): δ 7.79 (d, J = 8.1 Hz, 1H), 7.36–7.32 (m, 1H), 7.32–7.28 (m, 1H), 2.55 (t, J = 2.0 Hz, 3H) ppm; ^{13}**C-NMR** (151 MHz, $CDCl_3$): δ 161.6 (dd, J = 245.4, 5.7 Hz), 156.0 (dd, J = 346.0, 3.9 Hz), 130.5 (dd, J = 18.2, 7.0 Hz), 128.2, 127.2 (d, J = 8.7 Hz), 125.8 (dd, J = 58.3, 4.6 Hz), 121.7 (d, J = 24.0 Hz), 12.0 (d, J = 6.2 Hz) ppm; ^{19}**F-NMR** (565 MHz, $CDCl_3$): δ 31.9, –113.9 ppm; **HRMS-EI**: m/z calculated for $[C_8H_6F_2O]^{\cdot+}$ ($[M]^{\cdot+}$) 156.0381, found 156.0381; **IR** (ATR): $\tilde{\nu}$ = 1803 (vs) cm^{-1} (C=O).

3-Chloro-2-methylbenzoyl fluoride (4g)

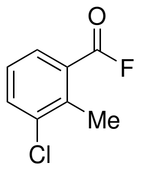

4g

The reaction of 3-chloro-2-methylbenzoic acid (853 mg, 5.00 mmol) following General Procedure A afforded after column chromatography (*n*-pentane) 3-chloro-2-methylbenzoyl fluoride **4g** as a colourless liquid (547 mg, 3.16 mmol, 63%).

R$_f$: 0.35 (*n*-pentane); ^{1}**H-NMR** (500 MHz, $CDCl_3$): δ 7.89 (dd, J = 7.9, 1.3 Hz, 1H), 7.66 (dd, J = 8.1, 1.4 Hz, 1H), 7.27 (t, J = 7.9 Hz, 1H), 2.70 (d, J = 1.2 Hz, 3H) ppm; ^{13}**C-NMR** (126 MHz, $CDCl_3$): δ 156.2 (d, J = 346.6 Hz), 140.9 (d, J = 7.2 Hz), 137.0 (d, J = 5.7 Hz), 135.6, 130.9 (d, J = 2.1 Hz), 127.0, 126.1 (d, J = 58.1 Hz), 17.6 ppm; ^{19}**F-NMR** (565 MHz, $CDCl_3$): δ 32.9 ppm; **HRMS-EI**: *m/z* calculated for $[C_8H_6ClFO]^{\cdot+}$ ($[M]^{\cdot+}$) 172.0086, found 172.0077; **IR** (ATR): $\tilde{\nu}$ = 1809 (vs) cm^{-1} (C=O).

3-Bromo-2-methylbenzoyl fluoride (4h)

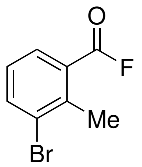

4h

The reaction of 3-bromo-2-methylbenzoic acid (1.08 g, 5.00 mmol) following General Procedure A afforded after column chromatography (*n*-pentane) 3-bromo-2-methylbenzoyl fluoride **4h** as a colourless liquid (759 mg, 3.50 mmol, 70%).

R$_f$: 0.33 (*n*-pentane); ^{1}**H-NMR** (500 MHz, $CDCl_3$): δ 7.92 (dd, J = 7.9, 1.3 Hz, 1H), 7.87–7.84 (m, 1H), 7.19 (t, J = 8.0, 1H), 2.74 (s, 3H) ppm; ^{13}**C-NMR** (126 MHz, $CDCl_3$): δ 156.1 (d, J = 346.8 Hz), 142.4 (d, J = 6.9 Hz), 138.9, 131.5 (d, J = 2.2 Hz), 127.8 (d, J = 5.3 Hz), 127.3, 126.1 (d, J = 57.9 Hz), 20.9 ppm; ^{19}**F-NMR** (471 MHz, $CDCl_3$): δ 32.8 ppm; **HRMS-EI**: *m/z* calculated for $[C_8H_6BrFO]^{\cdot+}$ ($[M]^{\cdot+}$) 215.9581, found 215.9576; **IR** (ATR): $\tilde{\nu}$ = 1808 (vs) cm^{-1} (C=O).

4-Fluoro-2-methylbenzoyl fluoride (4i)

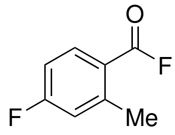

4i

The reaction of 4-fluoro-2-methylbenzoic acid (770 mg, 5.00 mmol) following General Procedure A afforded after column chromatography (*n*-pentane) 4-fluoro-2-methylbenzoyl fluoride **4i** as a colourless liquid (452 mg, 2.90 mmol, 58%).

R$_f$: 0.18 (*n*-pentane); ^{1}**H-NMR** (500 MHz, $CDCl_3$): δ 8.02 (dd, J = 8.7, 5.8 Hz, 1H), 7.06–6.98 (m, 2H), 2.65 (d, J = 1.9 Hz, 3H) ppm; ^{13}**C-NMR** (126 MHz, $CDCl_3$): δ 166.3 (d, J = 258.0 Hz), 155.9 (d, J = 343.7 Hz), 147.6 (dd, J = 9.6, 7.4 Hz), 135.5 (dd, J = 10.2, 2.1 Hz), 120.0 (dd, J = 58.1, 2.9 Hz), 119.4 (dd, J = 21.8, 4.2 Hz), 113.8 (d, J = 21.9 Hz), 22.1 ppm; ^{19}**F-NMR** (471 MHz, $CDCl_3$): δ 29.1, –102.2 (q, J = 8.4 Hz) ppm; **HRMS-EI**: *m/z* calculated for $[C_8H_6F_2O]^{\cdot+}$ ($[M]^{\cdot+}$) 156.0381, found 156.0383; **IR** (ATR): $\tilde{\nu}$ = 1809 (vs) cm^{-1} (C=O).

4-Chloro-2-methylbenzoyl fluoride (4j)

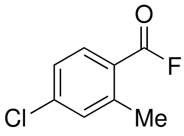

4j

The reaction of 4-chloro-2-methylbenzoic acid (853 mg, 5.00 mmol) following General Procedure A afforded after column chromatography (*n*-pentane) 4-chloro-2-methylbenzoyl fluoride **4j** as a colourless liquid (660 mg, 3.82 mmol, 76%).

R$_f$: 0.35 (*n*-pentane); ^{1}**H-NMR** (500 MHz, $CDCl_3$): δ 7.92 (d, J = 8.4 Hz, 1H), 7.35–7.33 (m, 1H), 7.31 (dd, J = 8.5, 2.1 Hz, 1H), 2.63 (d, J = 1.8 Hz, 3H) ppm; ^{13}**C-NMR** (126 MHz, $CDCl_3$): δ 156.0 (d, J = 344.6 Hz), 145.6 (d, J = 6.9 Hz), 141.3, 133.9 (d, J = 2.0 Hz), 132.4 (d, J = 4.1 Hz), 126.9, 122.2 (d, J = 58.0 Hz), 21.8 ppm; ^{19}**F-NMR** (565 MHz, $CDCl_3$): δ 32.9 ppm; **HRMS-EI**: *m/z* calculated for $[C_8H_6ClFO]^{\cdot+}$ ($[M]^{\cdot+}$) 172.0086, found 172.0082; **IR** (ATR): $\tilde{\nu}$ = 1807 (vs) cm^{-1} (C=O).

5-Fluoro-2-methylbenzoyl fluoride (4k)

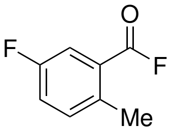

4k

The reaction of 5-fluoro-2-methylbenzoic acid (770 mg, 5.00 mmol) following General Procedure A afforded after column chromatography (*n*-pentane) 5-fluoro-2-methylbenzoyl fluoride **4k** as a colourless liquid (643 mg, 4.12 mmol, 82%).

R$_f$: 0.21 (*n*-pentane); ^{1}**H-NMR** (600 MHz, $CDCl_3$): δ 7.66 (dd, J = 8.9, 2.8 Hz, 1H), 7.33–7.30 (m, 1H), 7.27 (td, J = 8.1, 2.8 Hz, 1H), 2.61 (s, 3H) ppm; ^{13}**C-NMR** (151 MHz, $CDCl_3$): δ 160.7 (d, J = 246.2 Hz), 155.7 (dd, J = 346.0, 3.0 Hz), 139.5 (dd, J = 7.0, 3.5 Hz), 133.8 (dd, J = 7.3, 4.6 Hz), 124.9 (dd, J = 58.8, 7.4 Hz), 122.0 (d, J = 20.8 Hz), 119.0 (dd, J = 23.7, 2.0 Hz), 21.2 ppm; ^{19}**F-NMR** (565 MHz, $CDCl_3$): δ 29.7, –115.5 (q, J = 7.8 Hz) ppm; **HRMS-EI**: *m/z* calculated for $[C_8H_6F_2O]^{\cdot+}$ ($[M]^{\cdot+}$) 156.0381, found 156.0385; **IR** (ATR): $\tilde{\nu}$ = 1809 (vs) cm^{-1} (C=O).

5-Chloro-2-methylbenzoyl fluoride (4l)

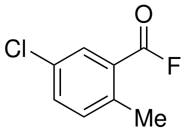

4l

The reaction of 5-chloro-2-methylbenzoic acid (853 mg, 5.00 mmol) following General Procedure A afforded after column chromatography (*n*-pentane) 5-chloro-2-methylbenzoyl fluoride **4l** as a white solid (660 mg, 3.82 mmol, 76%).

R$_f$: 0.21 (*n*-pentane); ^{1}**H-NMR** (500 MHz, $CDCl_3$): δ 7.96 (d, J = 2.4 Hz, 1H), 7.52 (dd, J = 8.2, 2.3 Hz, 1H), 7.29 (d, J = 8.2 Hz, 1H), 2.62 (d, J = 1.8 Hz, 3H) ppm; ^{13}**C-NMR** (126 MHz, $CDCl_3$): δ 155.7 (d, J = 346.4 Hz), 142.1 (d, J = 6.7 Hz), 134.7, 133.6 (d, J = 4.5 Hz), 132.3, 132.2 (d, J = 2.2 Hz), 125.1 (d, J = 58.1 Hz), 21.4 ppm; ^{19}**F-NMR** (471 MHz, $CDCl_3$): δ 29.9 ppm; **HRMS-EI**: *m/z* calculated for $[C_8H_6ClFO]^{\cdot+}$ ($[M]^{\cdot+}$) 172.0086, found 172.0093; **IR** (ATR): $\tilde{\nu}$ = 1795 (vs) cm^{-1} (C=O).

5-Bromo-2-methylbenzoyl fluoride (4m)

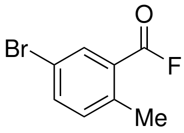

4m

The reaction of 5-bromo-2-methylbenzoic acid (1.08 g, 5.00 mmol) following General Procedure A afforded after column chromatography (*n*-pentane) 5-bromo-2-methylbenzoyl fluoride **4m** as a white solid (635 mg, 2.93 mmol, 59%).

R$_f$: 0.22 (*n*-pentane); ^{1}**H-NMR** (500 MHz, $CDCl_3$): δ 7.92 (dd, J = 7.9, 1.3 Hz, 1H), 7.87–7.84 (m, 1H), 7.19 (t, J = 8.0, 1H), 2.74 (s, 3H) ppm; ^{13}**C-NMR** (126 MHz, $CDCl_3$): δ 156.1 (d, J = 346.8 Hz), 142.4 (d, J = 6.9 Hz), 138.9, 131.5 (d, J = 2.2 Hz), 127.8 (d, J = 5.3 Hz), 127.3, 126.1 (d, J = 57.9 Hz), 20.9 ppm; ^{19}**F-NMR** (471 MHz, $CDCl_3$): δ 32.8 ppm; **HRMS-EI**: *m/z* calculated for $[C_8H_6BrFO]^{\cdot+}$ ($[M]^{\cdot+}$) 215.9581, found 215.9580; **IR** (ATR): $\tilde{\nu}$ = 1794 (vs) cm^{-1} (C=O).

5-Iodo-2-methylbenzoyl fluoride (4n)

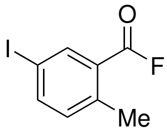

4n

The reaction of 5-iodo-2-methylbenzoic acid (1.31 g, 5.00 mmol) following General Procedure A afforded after column chromatography (*n*-pentane) 5-iodo-2-methylbenzoyl fluoride **4n** as a white solid (1.12 g, 4.24 mmol, 84%).

R$_f$: 0.16 (*n*-pentane); ^{1}**H-NMR** (500 MHz, $CDCl_3$): δ 8.28 (d, J = 2.0 Hz, 1H), 7.84 (dd, J = 8.1, 2.0 Hz, 1H), 7.08 (d, J = 8.2 Hz, 1H), 2.59 (d, J = 1.8 Hz, 3H) ppm; ^{13}**C-NMR** (126 MHz, $CDCl_3$): δ 155.4 (d, J = 346.6 Hz), 143.5, 143.2 (d, J = 6.6 Hz), 140.9, 134.0 (d, J = 4.2 Hz), 125.7 (d, J = 57.4 Hz), 90.3, 21.6 ppm; ^{19}**F-NMR** (471 MHz, $CDCl_3$): δ 30.0 ppm; **HRMS-EI**: *m/z* calculated for $[C_8H_6FIO]^{\cdot+}$ ($[M]^{\cdot+}$) 263.9442, found 263.9449; **IR** (ATR): $\tilde{\nu}$ = 1789 (vs) cm^{-1} (C=O).

2-Ethylbenzoyl fluoride (4o)

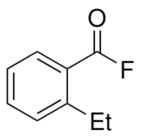

4o

The reaction of 2-ethylbenzoic acid (751 mg, 5.00 mmol) following General Procedure A afforded after column chromatography (*n*-pentane) 2-ethylbenzoyl fluoride **4o** as a colourless oil (372 mg, 2.45 mmol, 49%).

R$_f$: 0.19 (*n*-pentane); ^{1}**H-NMR** (400 MHz, $CDCl_3$): δ 7.99 (dd, J = 7.9, 1.2 Hz, 1H), 7.59 (td, J = 7.5, 1.3 Hz, 1H), 7.37 (d, J = 7.8 Hz, 1H), 7.33 (t, J = 8.0 Hz, 1H), 3.05 (q, J = 7.5 Hz, 2H), 1.26 (t, J = 7.5 Hz, 3H) ppm; ^{13}**C-NMR** (126 MHz, $CDCl_3$): δ 156.6 (d, J = 345.7 Hz), 149.7 (d, J = 7.7 Hz), 135.0, 132.7 (d, J = 2.3 Hz), 130.9 (d, J = 4.2 Hz), 126.4, 123.1 (d, J = 56.2 Hz), 27.8 (d, J = 1.5 Hz), 15.4 ppm; ^{19}**F-NMR** (376 MHz, $CDCl_3$): δ 30.3 ppm; **HRMS-EI**: *m/z* calculated for $[C_9H_9FO]^{\cdot+}$ ($[M]^{\cdot+}$) 152.0632, found 152.0629; **IR** (ATR): $\tilde{\nu}$ = 1805 (vs) cm^{-1} (C=O).

2-Benzylbenzoyl fluoride (4p)

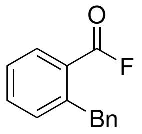

4p

The reaction of 2-benzylbenzoic acid (1.07 g, 5.00 mmol) following General Procedure A afforded after column chromatography (*n*-pentane) 2-benzylbenzoyl fluoride **4p** as a colourless oil (686 mg, 3.21 mmol, 64%).

R$_f$: 0.19 (*n*-pentane); ^{1}**H-NMR** (500 MHz, $CDCl_3$): δ 8.05 (dd, J = 7.9, 1.5 Hz, 1H), 7.60 (td, J = 7.6, 1.5 Hz, 1H), 7.39 (td, J = 7.7, 1.3 Hz, 1H), 7.33–7.29 (m, 3H), 7.26–7.22 (m, 1H), 7.20–7.17 (m, 2H), 4.46 (s, 2H) ppm; ^{13}**C-NMR** (126 MHz, $CDCl_3$): δ 156.6 (d, J = 346.1 Hz), 146.0 (d, J = 7.4 Hz), 139.7, 134.9 (d, J = 8.7 Hz), 132.9 (d, J = 13.8 Hz), 132.2 (d, J = 4.2 Hz), 129.2, 128.6, 127.0, 126.5, 123.6 (d, J = 56.7 Hz), 39.7 (t, J = 5.4 Hz) ppm; ^{19}**F-NMR** (376 MHz, $CDCl_3$): δ 30.4 ppm; **HRMS-EI**: *m/z* calculated for $[C_{14}H_{11}FO]^{\cdot+}$ ($[M]^{\cdot+}$) 214.0788, found 214.0785; **IR** (ATR): $\tilde{\nu}$ = 1801 (vs) cm^{-1} (C=O).

3-Methylthiophene-2-carbonyl fluoride (4q)

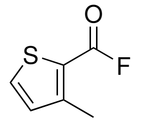

4q

The reaction of 3-methylthiophene-2-carboxylic acid (710 mg, 5.00 mmol) following General Procedure A afforded after column chromatography (*n*-pentane) 3-methylthiophene-2-carbonyl fluoride **4q** as a colourless liquid (530 mg, 3.68 mmol, 74%).

R$_f$: 0.22 (*n*-pentane); ^{1}**H-NMR** (600 MHz, $CDCl_3$): δ 7.62 (d, J = 5.0 Hz, 1H), 7.02 (dd, J = 5.0, 3.6 Hz, 1H), 2.58 (d, J = 1.4 Hz, 3H) ppm; ^{13}**C-NMR** (151 MHz, $CDCl_3$): δ 152.7 (d, J = 329.0 Hz), 151.8 (d, J = 8.2 Hz), 134.4, 132.4 (d, J = 4.8 Hz), 120.9 (d, J = 69.8 Hz), 16.2 (d, J = 2.2 Hz) ppm; ^{19}**F-NMR** (376 MHz, $CDCl_3$): δ 32.4 ppm; **IR** (ATR): $\tilde{\nu}$ = 1783 (vs) cm^{-1} (C=O); a satisfactory mass spectrum for this compound could not be obtained.

3.3 Preparation of IMe·HOTf Stock Solution

1,3-Dimethyl-1*H*-imidazol-3-ium triflate (IMe·HOTf)

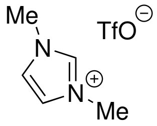

IMe·HOTf

To a solution of 1-methylimidazole (205 mg, 199 μL, 2.50 mmol, 1 equiv) in anhydrous diethyl ether (25 mL, 0.1 M) was added methyl triflate (451 mg, 311 μL, 2.75 mmol, 1.1 equiv). The reaction mixture was stirred at rt for 16 h. The white suspension was allowed to settle and the supernatant solution pipetted off. The white precipitate was dried under vacuum (rt, 30 min) and dissolved in anhydrous acetonitrile (2.5 mL), affording **IMe·HOTf** as a 0.95 M solution in acetonitrile (584 mg, 2.38 mmol, 95%).

^{1}**H-NMR** (600 MHz, $CDCl_3$): δ 8.80 (s, 1H), 7.26 (d, J = 1.6 Hz, 2H), 3.84 (s, 6H) ppm; ^{19}**F-NMR** (565 MHz, $CDCl_3$): δ –78.6 ppm.

The characterization data agree with literature precedents.[4]

4 Optimization of the NHC-catalyzed PEDA Reaction

4.1 Optimization Table

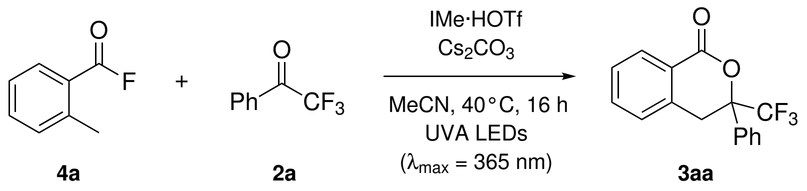

Entry	Equiv of **4a**	Equiv of **2a**	Mol% of IMe·HOTf	Equiv of Cs_2CO_3	Notes	Yield (NMR)
1	1	2	33	2	with anh. $MgSO_4$[a]	**3aa** (44%)
2	1	2	33	1	with anh. $MgSO_4$[a]	**3aa** (20%)
3	3	1	33	2	with anh. $MgSO_4$[a]	**3aa** (61%)
4	3	1	33	3	with anh. $MgSO_4$[a]	**3aa** (79%)
5	3	1	33	–	with anh. $MgSO_4$[a], DABCO (3 equiv)	**3aa** (traces)
6	3	1	33	3	–	**3aa** (81%)
7	3	1	33	2	–	**3aa** (86%)
8	3	1	30	2	–	**3aa** (83%)
9	3	1	20	2	–	**3aa** (88%)
10	3	1	10	2	–	**3aa** (48%)
11	2	1	20	2	–	**3aa** (76%)
12	1.5	1	20	2	–	**3aa** (51%)
13	3	1	20	1.5	–	**3aa** (69%)
14	3	1	20	2.5	–	**3aa** (75%)
15	3	1	20	2	MeCN (0.15 M)	**3aa** (83%)
16	3	1	20	2	MeCN (0.2 M)	**3aa** (81%)

[a] Anhydrous $MgSO_4$ (10 equiv) pre-dried by heating under vacuum.

4.2 Control Reactions

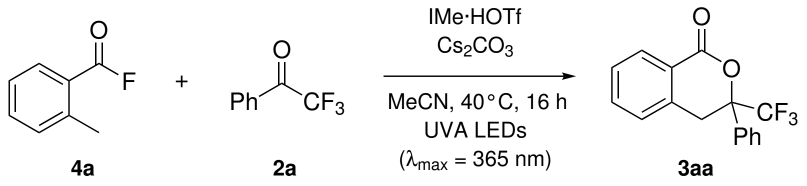

Entry	Equiv of **4a**	Equiv of **2a**	Mol% of IMe·HOTf	Equiv of Cs_2CO_3	Notes	Yield (NMR)
1	3	1	20	2	without light	**3aa** (2%)
2	3	1	–	2	–	**3aa** (1%)
3	3	1	20	–	–	**3aa** (2%)
4	3	1	–	–	–	**3aa** (2%)
5	3	1	20	2	not degassed	**3aa** (85%)

4.3 Screening of NHC-precatalysts

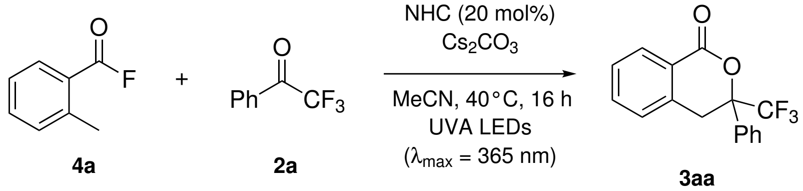

Entry	Equiv of **4a**	Equiv of **2a**	NHC·HX	Equiv of Cs_2CO_3	Notes	Yield (NMR)
1	3	1	IMe·HOTf	2	–	**3aa** (88%)
2	3	1	I^{i}Pr·HCl	2	–	**3aa** (27%)
3	3	1	ICy·HBF_4	2	–	**3aa** (18%)
4	3	1	IMes·HCl	2	–	**3aa** (7%)
5	3	1	IDipp·HCl	2	–	**3aa** (5%)
6	3	1	SIMes	–	–	**3aa** (6%)
7	3	1	BIMe·HOTf	2	–	**3aa** (43%)
8	3	1	TMe·HOTf	2	–	–
9	3	1	BTMe·HOTf	2	–	–
10	3	1	TriMe·HI	2	–	**3aa** (8%)
11	3	1	Bode Catalyst 2	2	–	**3aa** (2%)
12	3	1	Bode Catalyst 3	2	–	**3aa** (4%)
13	3	1	Rovis Triazolium	2	–	–

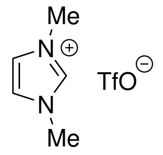
IMe·HOTf

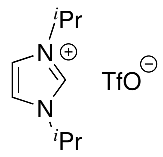
I^{i}Pr·HOTf

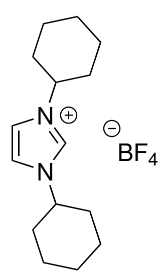
ICy·HBF_4

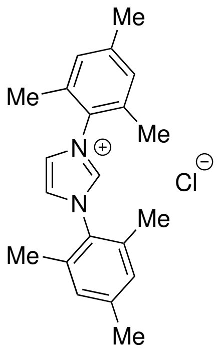
IMes·HCl

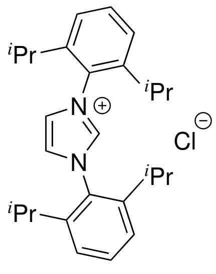
IDipp·HCl

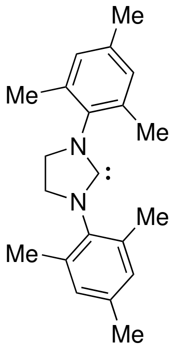
SIMes

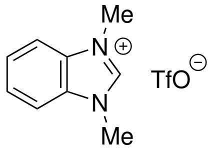
BIMe·HOTf

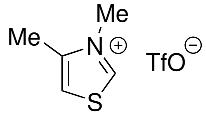
TMe·HOTf

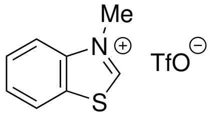
BTMe·HOTf

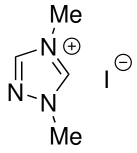
TriMe·HI

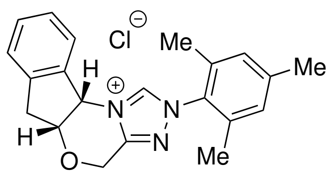
Bode Catalyst 2

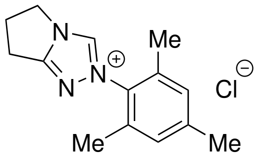
Bode Catalyst 3

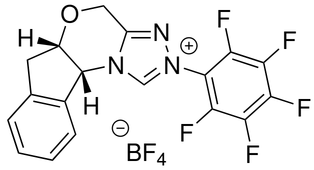
Rovis Triazolium

5 Scope of the NHC-catalyzed PEDA Reaction

5.1 NHC-catalyzed PEDA Reaction

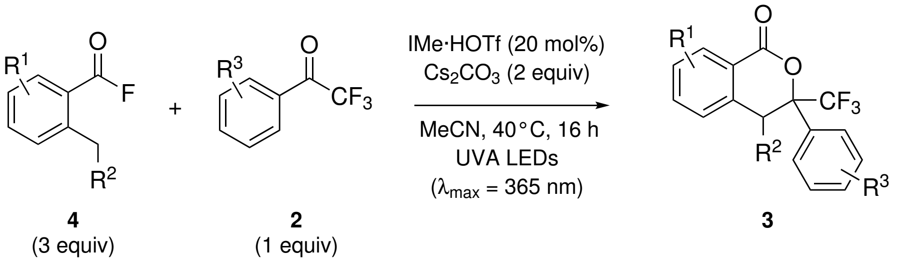

Scheme S4: General protocol for the synthesis of isochroman-1-ones **3**.

General Procedure B: A degassed suspension of acid fluoride **4** (0.90 mmol, 3 equiv), 2,2,2-trifluoroacetophenone **2** (0.30 mmol, 1 equiv), IMe·HOTf stock solution (1 M in MeCN, 60 μL, 15 mg, 0.060 mmol, 20 mol%) and anhydrous cesium carbonate (196 mg, 0.60 mmol, 2 equiv) in anhydrous acetonitrile (3 mL, 0.1 M) was irradiated with UVA LEDs (λ_{max} 365 nm) and stirred for 16 h at 40° C. Then, the reaction mixture was filtered through aluminium oxide (neutral, Brockmann activity I), the filtration residue was washed with dichloromethane (3 x 1 mL) and the combined organic solutions were concentrated under vacuum. The resulting crude product was purified by column chromatography on silica gel.

5.2 Characterization Data of Isochroman-1-ones 3

3-Phenyl-3-(trifluoromethyl)isochroman-1-one (3aa)

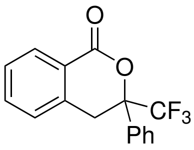

3aa

The reaction of 2-methylbenzoyl fluoride **4a** (124 mg, 0.90 mmol) and 2,2,2-trifluoroacetophenone **2a** (52 mg, 42 μL, 0.30 mmol) following General Procedure B afforded after column chromatography (*n*-pentane/Et_2O 9:1) isochroman-1-one **3aa** as a white solid (74 mg, 0.24 mmol, 84%).

R$_f$: 0.30 (*n*-pentane/Et_2O 5:1); ^{1}**H-NMR** (600 MHz, $CDCl_3$): δ 7.96 (dd, J = 8.2, 1.4 Hz, 1H), 7.58–7.51 (m, 2H), 7.49 (td, J = 7.6, 1.4 Hz, 1H), 7.35–7.25 (m, 5H), 3.83 (d, J = 16.3 Hz, 1H), 3.70 (d, J = 16.3 Hz, 1H) ppm; ^{13}**C-NMR** (151 MHz, $CDCl_3$): δ 162.5, 135.4, 134.8, 133.6, 130.4, 129.6, 128.8, 128.2, 128.0, 127.1, 124.5, 123.4 (q, J = 283.6 Hz), 83.3 (q, J = 30.6 Hz), 31.1 ppm; ^{19}**F-NMR** (565 MHz, $CDCl_3$): δ –79.5 ppm.

The characterization data agree with literature precedents.[5–7]

5-Methyl-3-phenyl-3-(trifluoromethyl)isochroman-1-one (3ba)

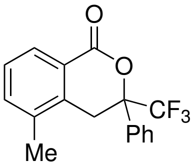

3ba

The reaction of 2,3-dimethylbenzoyl fluoride **4b** (137 mg, 0.90 mmol) and 2,2,2-trifluoroacetophenone **2a** (52 mg, 42 μL, 0.30 mmol) following General Procedure B afforded after column chromatography (*n*-pentane/EtOAc 20:1) isochroman-1-one **3ba** as a white solid (79 mg, 0.25 mmol, 86%).

R$_f$: 0.23 (*n*-pentane/EtOAc 10:1); ^{1}**H-NMR** (700 MHz, $CDCl_3$): δ 7.84 (d, J = 7.8 Hz, 1H), 7.51–7.49 (m, 2H), 7.35 (d, J = 7.5 Hz, 1H), 7.34–7.29 (m, 3H), 7.19 (t, J = 7.6, 1H), 3.76 (d, J = 16.5 Hz, 1H), 3.59 (d, J = 16.5 Hz, 1H), 2.40 (s, 3H) ppm; ^{13}**C-NMR** (176 MHz, $CDCl_3$): δ 162.9, 136.1, 135.5, 134.0, 133.9, 129.7, 128.9, 128.3, 127.6, 126.9, 124.6, 123.5 (q, J = 283.5 Hz), 83.00 (q, J = 30.6 Hz), 28.0, 19.1 ppm; ^{19}**F-NMR** (376 MHz, $CDCl_3$): δ –79.5 ppm; **HRMS-ESI**: *m/z* calculated for $[C_{17}H_{14}F_3O_2]^+$ ($[M+H]^+$) 307.0940, found 307.0930; **IR** (ATR): $\tilde{\nu}$ = 1732 (s) cm^{-1} (C=O).

6-Methyl-3-phenyl-3-(trifluoromethyl)isochroman-1-one (3ca)

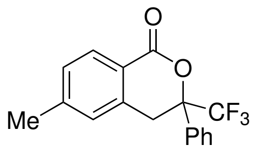

3ca

The reaction of 2,4-dimethylbenzoyl fluoride **4c** (137 mg, 0.90 mmol) and 2,2,2-trifluoroacetophenone **2a** (52 mg, 42 μL, 0.30 mmol) following General Procedure B afforded after column chromatography (n-pentane/EtOAc 20:1) isochroman-1-one **3ca** as a white solid (60 mg, 0.20 mmol, 65%).

R$_f$: 0.22 (n-pentane/EtOAc 10:1); ^{1}**H-NMR** (600 MHz, $CDCl_3$): δ 7.84 (d, J = 7.9 Hz, 1H), 7.53 (d, J = 7.4 Hz, 2H), 7.36–7.27 (m, 3H), 7.10–7.05 (m, 2H), 3.78 (d, J = 16.3 Hz, 1H), 3.63 (d, J = 16.3 Hz, 1H), 2.34 (s, 3H) ppm; ^{13}**C-NMR** (151 MHz, $CDCl_3$): δ 162.6, 146.0, 135.4, 133.8, 130.5, 129.6, 129.2, 128.8, 128.5, 127.1, 123.4 (q, J = 283.6 Hz), 121.8, 83.2 (q, J = 30.5 Hz), 31.1, 21.9 ppm; ^{19}**F-NMR** (565 MHz, $CDCl_3$): δ –79.5 ppm.

The characterization data agree with literature precedents.[6]

7-Methyl-3-phenyl-3-(trifluoromethyl)isochroman-1-one (3da)

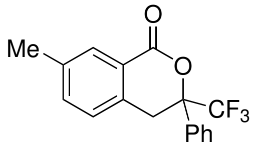

3da

The reaction of 2,5-dimethylbenzoyl fluoride **4d** (137 mg, 0.90 mmol) and 2,2,2-trifluoroacetophenone **2a** (52 mg, 42 μL, 0.30 mmol) following General Procedure B afforded after column chromatography (n-pentane/EtOAc 20:1) isochroman-1-one **3da** as a white solid (70 mg, 0.23 mmol, 76%).

R$_f$: 0.27 (n-pentane/EtOAc 10:1); ^{1}**H-NMR** (500 MHz, $CDCl_3$): δ 7.73 (s, 1H), 7.49 (d, J = 7.4 Hz, 2H), 7.30–7.23 (m, 4H), 7.13 (d, J = 7.7 Hz, 1H), 3.74 (d, J = 16.2 Hz, 1H), 3.62 (d, J = 16.2 Hz, 1H), 2.23 (s, 3H) ppm; ^{13}**C-NMR** (176 MHz, $CDCl_3$): δ 162.7, 138.2, 135.6, 133.7, 132.4, 130.7, 129.6, 128.8, 127.9, 127.2, 124.2, 123.4 (q, J = 283.7 Hz), 83.3 (q, J = 30.3 Hz), 30.8, 21.0 ppm; ^{19}**F-NMR** (471 MHz, $CDCl_3$): δ –79.5 ppm; **HRMS-ESI**: m/z calculated for $[C_{17}H_{14}F_3O_2]^+$ ($[M+H]^+$) 307.0940, found 307.0969; **IR** (ATR): $\tilde{\nu}$ = 1732 (s) cm^{-1} (C=O).

8-Methyl-3-phenyl-3-(trifluoromethyl)isochroman-1-one (3ea)

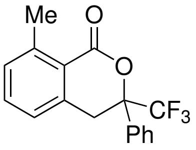

3ea

The reaction of 2,6-dimethylbenzoyl fluoride **4e** (137 mg, 0.90 mmol) and 2,2,2-trifluoroacetophenone **2a** (52 mg, 42 μL, 0.30 mmol) following General Procedure B afforded after column chromatography (n-pentane/EtOAc 20:1) isochroman-1-one **3ea** as a white solid (16 mg, 0.05 mmol, 17%).

R$_f$: 0.28 (n-pentane/EtOAc 10:1); ^{1}**H-NMR** (700 MHz, $CDCl_3$): δ 7.50–7.47 (m, 2H), 7.33–7.27 (m, 4H), 7.08 (d, J = 7.7 Hz, 2H), 3.80 (d, J = 16.0 Hz, 1H), 3.63 (d, J = 16.0 Hz, 1H), 2.57 (s, 3H) ppm; ^{13}**C-NMR** (176 MHz, $CDCl_3$): δ 161.6, 143.5, 136.3, 133.68, 133.66, 131.7, 129.5, 128.8, 127.1, 126.0, 123.4 (q, J = 283.4 Hz), 123.0, 82.5 (q, J = 30.8 Hz), 32.1, 22.3 ppm; ^{19}**F-NMR** (471 MHz, $CDCl_3$): δ –79.6 ppm; **HRMS-ESI**: m/z calculated for $[C_{17}H_{13}F_3NaO_2]^+$ ($[M+Na]^+$) 329.0760, found 329.0753; **IR** (ATR): $\tilde{\nu}$ = 1740 (s) cm^{-1} (C=O).

5-Fluoro-3-phenyl-3-(trifluoromethyl)isochroman-1-one (3fa)

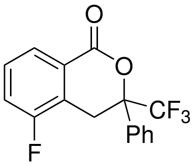

3fa

The reaction of 3-fluoro-2-methylbenzoyl fluoride **4f** (140 mg, 0.90 mmol) and 2,2,2-trifluoroacetophenone **2a** (52 mg, 42 μL, 0.30 mmol) following General Procedure B afforded after column chromatography (n-pentane/EtOAc 20:1) isochroman-1-one **3fa** as a white solid (85 mg, 0.27 mmol, 91%).

R$_f$: 0.28 (n-pentane/EtOAc 10:1); ^{1}**H-NMR** (600 MHz, $CDCl_3$): δ 7.78 (dd, J = 7.0, 1.9 Hz, 1H), 7.52 (d, J = 7.4 Hz, 2H), 7.38–7.27 (m, 3H), 7.29–7.23 (m, 2H), 3.96 (d, J = 16.7 Hz, 1H), 3.60 (d, J = 16.7 Hz, 1H) ppm; ^{13}**C-NMR** (151 MHz, $CDCl_3$): δ 161.3 (d, J = 3.9 Hz), 159.2 (d, J = 248.4 Hz), 133.3, 129.9, 129.1 (d, J = 7.8 Hz), 129.0, 126.99, 126.98, 126.1 (d, J = 3.7 Hz), 123.2 (q, J = 283.6 Hz), 122.8 (d, J = 18.6 Hz), 121.2 (d, J = 21.0 Hz), 83.3 (q, J = 30.8 Hz), 24.3 ppm; ^{19}**F-NMR** (565 MHz, $CDCl_3$): δ –79.6, –119.2 ppm.

The characterization data agree with literature precedents.[7]

5-Chloro-3-phenyl-3-(trifluoromethyl)isochroman-1-one (3ga)

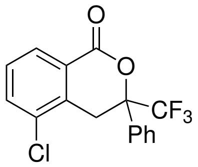

3ga

The reaction of 3-chloro-2-methylbenzoyl fluoride **4g** (156 mg, 0.90 mmol) and 2,2,2-trifluoroacetophenone **2a** (52 mg, 42 μL, 0.30 mmol) following General Procedure B afforded after column chromatography (*n*-pentane/EtOAc 20:1) isochroman-1-one **3ga** as a white solid (93 mg, 0.29 mmol, 95%).

R$_f$: 0.35 (*n*-pentane/EtOAc 10:1); ^{1}**H-NMR** (600 MHz, $CDCl_3$): δ 7.88 (dd, J = 7.8, 1.2 Hz, 1H), 7.55 (dd, J = 8.1, 1.2 Hz, 1H), 7.51 (d, J = 7.2 Hz, 2H), 7.38–7.26 (m, 3H), 7.23 (t, J = 7.9 Hz, 1H), 4.11 (d, J = 16.9 Hz, 1H), 3.64 (d, J = 16.9 Hz, 1H) ppm; ^{19}**F-NMR** (565 MHz, $CDCl_3$): δ –79.6 ppm.

The characterization data agree with literature precedents.[7]

5-Bromo-3-phenyl-3-(trifluoromethyl)isochroman-1-one (3ha)

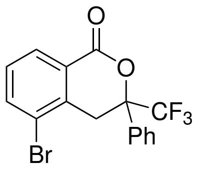

3ha

The reaction of 3-bromo-2-methylbenzoyl fluoride **4h** (195 mg, 0.90 mmol) and 2,2,2-trifluoroacetophenone **2a** (52 mg, 42 μL, 0.30 mmol) following General Procedure B afforded after column chromatography (*n*-pentane/Et_2O 9:1) isochroman-1-one **3ha** as a white solid (97 mg, 0.26 mmol, 87%).

R$_f$: 0.33 (*n*-pentane/Et_2O 5:1); ^{1}**H-NMR** (600 MHz, $CDCl_3$): δ 7.93 (d, J = 7.8 Hz, 1H), 7.72 (dd, J = 8.0, 1.2 Hz, 1H), 7.51 (d, J = 7.7 Hz, 2H), 7.36–7.30 (m, 3H), 7.18 (t, J = 7.9 Hz, 1H), 4.09 (d, J = 16.8 Hz, 1H), 3.65 (d, J = 16.8 Hz, 1H) ppm; ^{13}**C-NMR** (151 MHz, $CDCl_3$): δ 161.7, 138.2, 135.5, 133.3, 129.9, 129.7, 129.3, 129.0, 127.1, 126.6, 123.4, 123.2 (q, J = 283.5 Hz), 83.2 (q, J = 30.8 Hz), 31.0 ppm; ^{19}**F-NMR** (565 MHz, $CDCl_3$): δ –79.6 ppm; **HRMS-ESI**: *m/z* calculated for $[C_{16}H_{10}BrF_3NaO_2]^+$ ($[M+Na]^+$) 392.9708, found 392.9697; **IR** (ATR): $\tilde{\nu}$ = 1737 (s) cm^{-1} (C=O).

6-Fluoro-3-phenyl-3-(trifluoromethyl)isochroman-1-one (3ia)

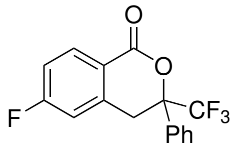

3ia

The reaction of 4-fluoro-2-methylbenzoyl fluoride **4i** (140 mg, 0.90 mmol) and 2,2,2-trifluoroacetophenone **2a** (52 mg, 42 μL, 0.30 mmol) following General Procedure B afforded after column chromatography (n-pentane/EtOAc 20:1) isochroman-1-one **3ia** as a white solid (79 mg, 0.27 mmol, 85%).

R$_f$: 0.30 (n-pentane/EtOAc 10:1); ^{1}**H-NMR** (600 MHz, $CDCl_3$): δ 7.98 (dd, J = 8.7, 5.5 Hz, 1H), 7.52 (d, J = 7.2 Hz, 2H), 7.36–7.28 (m, 3H), 7.01–6.93 (m, 2H), 3.82 (d, J = 16.4 Hz, 1H), 3.70 (d, J = 16.4 Hz, 1H) ppm; ^{13}**C-NMR** (151 MHz, $CDCl_3$): δ 166.3 (d, J = 257.9 Hz), 161.5, 138.5 (d, J = 9.6 Hz), 133.5 (d, J = 10.0 Hz), 133.2, 129.8, 128.9, 127.0, 123.2 (q, J = 283.6 Hz), 120.8 (d, J = 2.9 Hz), 115.9 (d, J = 22.1 Hz), 115.1 (d,J = 23.0 Hz), 83.2 (q, J = 30.6 Hz), 31.2 ppm; ^{19}**F-NMR** (565 MHz, $CDCl_3$): δ –79.6, –101.4 (q, J = 7.7 Hz) ppm.

The characterization data agree with literature precedents.[5–7]

6-Chloro-3-phenyl-3-(trifluoromethyl)isochroman-1-one (3ja)

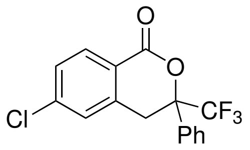

3ja

The reaction of 4-chloro-2-methylbenzoyl fluoride **4j** (156 mg, 0.90 mmol) and 2,2,2-trifluoroacetophenone **2a** (52 mg, 42 μL, 0.30 mmol) following General Procedure B afforded after column chromatography (n-pentane/Et_2O 9:1) isochroman-1-one **3ja** as a white solid (90 mg, 0.28 mmol, 92%).

R$_f$: 0.37 (n-pentane/Et_2O 5:1); ^{1}**H-NMR** (600 MHz, $CDCl_3$): δ 7.90 (d, J = 8.2 Hz, 1H), 7.50 (dd, J = 7.5, 2.1 Hz, 2H), 7.37–7.30 (m, 3H), 7.31–7.23 (m, 2H), 3.81 (d, J = 16.4 Hz, 1H), 3.67 (d, J = 16.4 Hz, 1H) ppm; ^{19}**F-NMR** (565 MHz, $CDCl_3$): δ –79.5 ppm.

The characterization data agree with literature precedents.[7]

7-Fluoro-3-phenyl-3-(trifluoromethyl)isochroman-1-one (3ka)

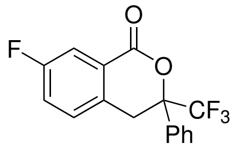

3ka

The reaction of 5-fluoro-2-methylbenzoyl fluoride **4k** (140 mg, 0.90 mmol) and 2,2,2-trifluoroacetophenone **2a** (52 mg, 42 μL, 0.30 mmol) following General Procedure B afforded after column chromatography (*n*-pentane/EtOAc 20:1) isochroman-1-one **3ka** as a white solid (88 mg, 0.28 mmol, 95%).

R$_f$: 0.21 (*n*-pentane/EtOAc 10:1); ^{1}**H-NMR** (600 MHz, $CDCl_3$): δ 7.63 (dd, J = 8.3, 2.8 Hz, 1H), 7.51 (dd, J = 7.4, 2.2 Hz, 2H), 7.35–7.30 (m, 3H), 7.28 (dd, J = 8.4, 4.8 Hz, 1H), 7.20 (td,J = 8.3, 2.8 Hz, 1H), 3.79 (d, J = 16.3 Hz, 1H), 3.71 (d, J = 16.3 Hz, 1H) ppm; ^{13}**C-NMR** (151 MHz, $CDCl_3$): δ 162.0 (d, J = 248.5 Hz), 161.5 (d, J = 2.9 Hz), 133.3, 131.2 (d, J = 3.2 Hz), 129.9 (d, J = 7.4 Hz), 129.8, 128.9, 127.1, 126.1 (d, J = 7.8 Hz), 123.3 (q, J = 283.6 Hz), 122.2 (d, J = 22.1 Hz), 116.9 (d, J = 23.5 Hz), 83.6 (q, J = 30.7 Hz), 30.5 (q, J = 1.5 Hz) ppm; ^{19}**F-NMR** (565 MHz, $CDCl_3$): δ –79.5, –111.9 (q, J = 7.1, 6.7 Hz) ppm; **HRMS-ESI**: *m/z* calculated for $[C_{16}H_{10}F_4NaO_2]^+$ ($[M+Na]^+$) 333.0509, found 333.0499; **IR** (ATR): $\tilde{\nu}$ = 1743 (s) cm^{-1} (C=O).

7-Chloro-3-phenyl-3-(trifluoromethyl)isochroman-1-one (3la)

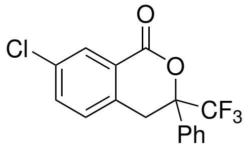

3la

The reaction of 5-chloro-2-methylbenzoyl fluoride **4l** (156 mg, 0.90 mmol) and 2,2,2-trifluoroacetophenone **2a** (52 mg, 42 μL, 0.30 mmol) following General Procedure B afforded after column chromatography (*n*-pentane/EtOAc 20:1) isochroman-1-one **3la** as a white solid (91 mg, 0.28 mmol, 93%).

R$_f$: 0.24 (*n*-pentane/EtOAc 10:1); ^{1}**H-NMR** (700 MHz, $CDCl_3$): δ 7.93 (d, J = 2.3 Hz, 1H), 7.52–7.48 (m, 2H), 7.45 (dd, J = 8.2, 2.3 Hz, 1H), 7.33 (h, J = 3.8 Hz, 3H), 7.24 (d, J = 8.2 Hz, 1H), 3.79 (d, J = 16.4 Hz, 1H), 3.70 (d, J = 16.4 Hz, 1H) ppm; ^{13}**C-NMR** (176 MHz, $CDCl_3$): δ 161.3, 134.8, 134.3, 133.8, 133.2, 130.2, 129.9, 129.5, 129.0, 127.1, 125.9, 123.2 (q, J = 283.6 Hz), 83.4 (q, J = 30.7 Hz), 30.7 ppm; ^{19}**F-NMR** (565 MHz, $CDCl_3$): δ –79.5. ppm.

The characterization data agree with literature precedents.[7]

7-Bromo-3-phenyl-3-(trifluoromethyl)isochroman-1-one (3ma)

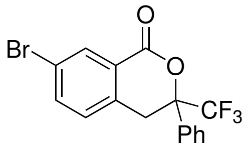

3ma

The reaction of 5-bromo-2-methylbenzoyl fluoride **4m** (195 mg, 0.90 mmol) and 2,2,2-trifluoroacetophenone **2a** (52 mg, 42 μL, 0.30 mmol) following General Procedure B afforded after column chromatography (n-pentane/EtOAc 20:1) isochroman-1-one **3ma** as a white solid (91 mg, 0.25 mmol, 82%).

R$_f$: 0.20 (n-pentane/EtOAc 10:1); ^{1}**H-NMR** (700 MHz, $CDCl_3$): δ 8.10 (d, J = 2.1 Hz, 1H), 7.61 (dd, J = 8.2, 2.1 Hz, 1H), 7.51–7.47 (m, 2H), 7.36–7.31 (m, 3H), 7.17 (dd, J = 8.2, 1.1 Hz, 1H), 3.76 (d, J = 16.4 Hz, 1H), 3.67 (d, J = 16.4 Hz, 1H) ppm; ^{13}**C-NMR** (151 MHz, $CDCl_3$): δ 161.7, 138.2, 135.5, 133.3, 129.9, 129.7, 129.2, 129.0, 127.1, 126.6, 123.4, 123.2 (q, J = 283.6 Hz), 83.2 (q, J = 30.8 Hz), 31.0 ppm; ^{19}**F-NMR** (565 MHz, $CDCl_3$): δ –79.5 ppm; **HRMS-ESI**: m/z calculated for $[C_{16}H_{11}BrF_3O_2]^+$ ($[M+H]^+$) 370.9889, found 370.9915; **IR** (ATR): $\tilde{\nu}$ = 1737 (s) cm^{-1} (C=O).

7-Iodo-3-phenyl-3-(trifluoromethyl)isochroman-1-one (3na)

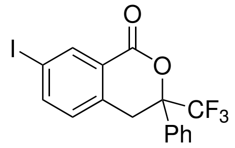

3na

The reaction of 5-iodo-2-methylbenzoyl fluoride **4n** (238 mg, 0.90 mmol) and 2,2,2-trifluoroacetophenone **2a** (52 mg, 42 μL, 0.30 mmol) following General Procedure B afforded after column chromatography (n-pentane/EtOAc 20:1) isochroman-1-one **3na** as a white solid (91 mg, 0.22 mmol, 75%).

R$_f$: 0.27 (n-pentane/EtOAc 10:1); ^{1}**H-NMR** (700 MHz, $CDCl_3$): δ 8.28 (s, 1H), 7.79 (d, J = 8.1 Hz, 1H), 7.52–7.48 (m, 2H), 7.36–7.31 (m, 3H), 7.04 (d, J = 8.1 Hz, 1H), 3.75 (d, J = 16.4 Hz, 1H), 3.67 (d, J = 16.4 Hz, 1H) ppm; ^{13}**C-NMR** (176 MHz, $CDCl_3$): δ 161.0, 143.5, 139.0, 134.8, 133.2, 129.9, 129.7, 129.0, 127.1, 126.1, 123.2 (q, J = 283.6 Hz), 92.8, 83.3 (q, J = 30.7 Hz), 30.8 ppm; ^{19}**F-NMR** (565 MHz, $CDCl_3$): δ –79.5 ppm; **HRMS-ESI**: m/z calculated for $[C_{16}H_{10}F_3INaO_2]^+$ ($[M+Na]^+$) 440.9570, found 440.9554; **IR** (ATR): $\tilde{\nu}$ = 1742 (s) cm^{-1} (C=O).

4-Methyl-3-phenyl-3-(trifluoromethyl)isochroman-1-one (3oa)

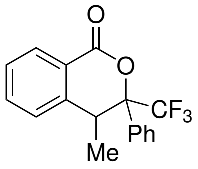

3oa

The reaction of 2-ethylbenzoyl fluoride **4o** (137 mg, 0.9 mmol) and 2,2,2-trifluoroacetophenone **2a** (52 mg, 42 μL, 0.30 mmol) following General Procedure B afforded a diastereomeric mixture (d.r. 2.6:1). Column chromatography (n-pentane/EtOAc 20:1) afforded the major diastereomer of **3oa** as a white solid (63 mg, 0.21 mmol, 69%) and the minor diastereomer of **3oa** as a pale yellow oil (22 mg, 0.07 mmol, 24%).

Major Diastereomer: $\mathbf{R}_f$: 0.33 (n-pentane/EtOAc 10:1); ^{1}**H-NMR** (600 MHz, $CDCl_3$): δ 8.14 (dd, J = 7.8, 1.4 Hz, 1H), 7.72 (d, J = 7.8 Hz, 2H), 7.61 (td, J = 7.6, 1.4 Hz, 1H), 7.51–7.46 (m, 2H), 7.45–7.41 (m, 2H), 7.33 (d, J = 7.6 Hz, 1H), 3.78 (q, J = 7.1 Hz, 1H), 0.99 (d, J = 7.1 Hz, 3H) ppm; ^{13}**C-NMR** (151 MHz, $CDCl_3$): δ 162.5, 142.6, 134.8, 134.2, 130.1, 129.2, 128.8, 128.1, 126.7, 126.0, 124.7 (q, J = 288.8 Hz), 123.3, 85.5 (q, J = 27.9 Hz), 36.1, 20.9 ppm; ^{19}**F-NMR** (565 MHz, $CDCl_3$): δ –72.6 ppm; **HRMS-ESI**: *m/z* calculated for $[C_{17}H_{13}F_3NaO_2]^+$ ($[M+Na]^+$) 329.0760, found 329.0753; **IR** (ATR): $\tilde{\nu}$ = 1732 (s) cm^{-1} (C=O).

Minor Diastereomer: $\mathbf{R}_f$: 0.23 (n-pentane/EtOAc 10:1); ^{1}**H-NMR** (600 MHz, $CDCl_3$): δ 7.91 (dd, J = 7.8, 1.3 Hz, 1H), 7.50–7.39 (m, 3H), 7.31–7.17 (m, 4H), 7.16 (d, J = 8.0 Hz, 1H), 3.94 (q, J = 7.0 Hz, 1H), 1.59–1.55 (m, 3H) ppm; ^{13}**C-NMR** (126 MHz, $CDCl_3$): δ 162.8, 143.3, 135.8, 135.0, 130.3, 129.3, 128.8, 128.0, 127.2, 126.5, 123.41, 123.37 (q, J = 284.6 Hz), 85.4 (q, J = 30.1 Hz), 38.4, 19.2 (q, J = 3.1 Hz) ppm; ^{19}**F-NMR** (565 MHz, $CDCl_3$): δ –74.1 ppm; **HRMS-ESI**: *m/z* calculated for $[C_{17}H_{13}F_3NaO_2]^+$ ($[M+Na]^+$) 329.0760, found 329.0752; **IR** (ATR): $\tilde{\nu}$ = 1736 (s) cm^{-1} (C=O).

3,4-Diphenyl-3-(trifluoromethyl)isochroman-1-one (3pa)

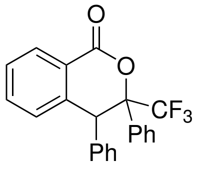

3pa

The reaction of 2-benzylbenzoyl fluoride **4p** (137 mg, 0.90 mmol) and 2,2,2-trifluoroacetophenone **2a** (52 mg, 42 μL, 0.30 mmol) following General Procedure B afforded a diastereomeric mixture (d.r. 10:1). Column chromatography (n-pentane/EtOAc 20:1) afforded the major diastereomer of **3pa** as a white solid (53 mg, 0.14 mmol, 48%). The minor diastereomer was not isolated.

Major Diastereomer: **R$_f$**: 0.27 (n-pentane/EtOAc 10:1); ^{1}**H-NMR** (600 MHz, $CDCl_3$): δ 8.25 (d, J = 7.8 Hz, 1H), 7.58–7.51 (m, 3H), 7.42 (t, J = 7.6 Hz, 1H), 7.31 (d, J = 7.7 Hz, 1H), 7.25–7.20 (m, 2H), 7.20–7.15 (m, 1H), 7.03–6.98 (m, 2H), 6.98–6.93 (m, 3H), 4.94 (s, 1H) ppm; ^{13}**C-NMR** (151 MHz, $CDCl_3$): δ 162.9, 140.1, 138.3, 135.0, 133.9, 130.1, 128.70, 128.69, 128.68, 128.3, 128.14, 128.08, 127.4, 126.4, 124.6 (q, J = 289.4 Hz), 123.1, 85.9 (q, J = 27.9 Hz), 47.4 ppm; ^{19}**F-NMR** (565 MHz, $CDCl_3$): δ –72.8 ppm.

The characterization data agree with literature precedents.[6]

5-Phenyl-5-(trifluoromethyl)-4,5-dihydro-7*H*-thieno[2,3-*c*]pyran-7-one (3qa)

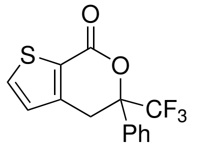

3qa

The reaction of 3-methylthiophene-2-carbonyl fluoride **4q** (130 mg, 0.90 mmol) and 2,2,2-trifluoroacetophenone **2a** (52 mg, 42 μL, 0.30 mmol) following General Procedure B afforded after column chromatography (n-pentane/EtOAc 20:1) isochroman-1-one **3qa** as a white solid (87 mg, 0.29 mmol, 97%).

R$_f$: 0.31 (n-pentane/EtOAc 10:1); ^{1}**H-NMR** (600 MHz, $CDCl_3$): δ 7.61 (d, J = 5.0 Hz, 1H), 7.58–7.55 (m, 2H), 7.37–7.33 (m, 3H), 6.99 (d, J = 5.0 Hz, 1H), 3.81 (d, J = 16.8 Hz, 1H), 3.69 (d, J = 16.8 Hz, 1H) ppm; ^{13}**C-NMR** (151 MHz, $CDCl_3$): δ 158.0, 143.8, 136.0, 133.8, 129.8, 128.9, 126.9, 126.8, 125.7, 123.4 (q, J = 283.6 Hz), 84.9 (q, J = 30.5 Hz), 28.8 ppm; ^{19}**F-NMR** (565 MHz, $CDCl_3$): δ –79.1 ppm; **HRMS-ESI**: *m/z* calculated for $[C_{14}H_9F_3NaO_2S]^+$ ($[M+Na]^+$) 321.0168, found 321.0162; **IR** (ATR): $\tilde{\nu}$ = 1725 (vs) cm^{-1} (C=O).

3-(Trifluoromethyl)-3-(3-(trifluoromethyl)phenyl)isochroman-1-one (3ab)

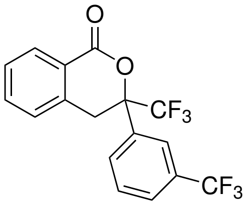

3ab

The reaction of 2-methylbenzoyl fluoride **4a** (124 mg, 0.90 mmol) and 2,2,2-trifluoro-1-(3-(trifluoromethyl)phenyl)ethan-1-one (73 mg, 51 μL, 0.30 mmol) following General Procedure B afforded after column chromatography (n-pentane/diethyl ether 9:1) isochroman-1-one **3ab** as a yellow oil (95 mg, 0.26 mmol, 88%).

$\mathbf{R}_f$: 0.17 (n-pentane/diethyl ether 5:1); ^{1}**H-NMR** (600 MHz, $CDCl_3$): δ 7.98 (d, J = 7.8 Hz, 1H), 7.79–7.75 (m, 2H), 7.58 (d, J = 7.8 Hz, 1H), 7.53 (td, J = 7.6, 1.3 Hz, 1H), 7.48 (t, J = 8.2 Hz, 1H), 7.35–7.29 (m, 2H), 3.89 (d, J = 16.5 Hz, 1H), 3.71 (d, J = 16.5 Hz, 1H) ppm; ^{13}**C-NMR** (151 MHz, $CDCl_3$): δ 161.9, 135.1, 134.9, 131.5 (q, J = 32.7 Hz), 130.64, 130.63, 129.6, 128.6, 127.984, 127.978, 126.7 (q, J = 3.6 Hz), 124.2, 123.9 (q, J = 4.0 Hz), 123.7 (q, J = 272.6 Hz), 123.2 (q, J = 283.9 Hz), 82.8 (q, J = 30.7 Hz), 31.1 ppm; ^{19}**F-NMR** (565 MHz, $CDCl_3$): δ –62.7, –79.3 ppm; **HRMS-ESI**: m/z calculated for $[C_{17}H_{10}F_6NaO_2]^+$ ($[M+Na]^+$) 383.0477, found 383.0469; **IR** (ATR): $\tilde{\nu}$ = 1740 (s) cm^{-1} (C=O).

3-(Trifluoromethyl)-3-(4-(trifluoromethyl)phenyl)isochroman-1-one (3ac)

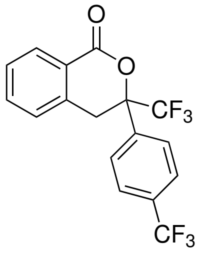

3ac

The reaction of 2-methylbenzoyl fluoride **4a** (124 mg, 0.90 mmol) and 2,2,2-trifluoro-1-(4-(trifluoromethyl)phenyl)ethan-1-one (73 mg, 51 μL, 0.30 mmol) following General Procedure B afforded after column chromatography (n-pentane/diethyl ether 9:1) isochroman-1-one **3ac** as a pale yellow oil (88 mg, 0.24 mmol, 81%).

$\mathbf{R}_f$: 0.24 (n-pentane/diethyl ether 4:1); ^{1}**H-NMR** (500 MHz, $CDCl_3$): δ 7.98 (dd, J = 7.8, 1.5 Hz, 1H), 7.68 (d, J = 8.5 Hz, 2H), 7.58 (d, J = 8.5 Hz, 2H), 7.52 (td, J = 7.6, 1.4 Hz, 1H), 7.35–7.29 (m, 2H), 3.88 (d, J = 16.3 Hz, 1H), 3.71 (d, J = 16.3 Hz, 1H) ppm; ^{13}**C-NMR** (126 MHz, $CDCl_3$): δ 162.0, 137.8, 135.1, 134.9, 131.9 (q, J = 33.0 Hz), 130.7, 128.6, 128.0, 127.7, 125.9 (q, J = 3.6 Hz), 124.2, 123.6 (q, J = 272.5 Hz), 123.1 (q, J = 283.8 Hz), 83.0 (q, J = 30.9 Hz), 31.1 ppm; ^{19}**F-NMR** (565 MHz, $CDCl_3$): δ –63.0, –79.3 ppm; **HRMS-ESI**: m/z calculated for $[C_{17}H_{10}F_6NaO_2]^+$ ($[M+Na]^+$) 383.0477, found 383.0465; **IR** (ATR): $\tilde{\nu}$ = 1733 (m) cm^{-1} (C=O).

3-(3-Methoxyphenyl)-3-(trifluoromethyl)isochroman-1-one (3ad)

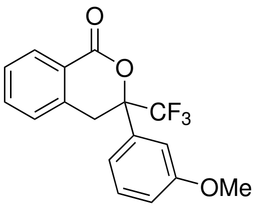

3ad

The reaction of 2-methylbenzoyl fluoride **4a** (124 mg, 0.90 mmol) and 2,2,2-trifluoro-1-(3-methoxyphenyl)ethan-1-one (61 mg, 47 μL, 0.30 mmol) following General Procedure B afforded after column chromatography (cyclohexane/methyl *t*-butyl ether 9:1) isochroman-1-one **3ad** as a yellow oil (91 mg, 0.28 mmol, 94%).

R$_f$: 0.27 (cyclohexane/methyl *t*-butyl ether 4:1); ^{1}**H-NMR** (600 MHz, $CDCl_3$): δ 7.96 (dd, J = 7.8, 1.4 Hz, 1H), 7.48 (td, J = 7.6, 1.4 Hz, 1H), 7.30–7.25 (m, 2H), 7.23–7.18 (m, 1H), 7.09–7.05 (m, 2H), 6.83–6.79 (m, 1H), 3.80 (d, J = 16.3 Hz, 1H), 3.73 (s, 3H), 3.66 (d, J = 16.3 Hz, 1H) ppm; ^{13}**C-NMR** (151 MHz, $CDCl_3$): δ 162.4, 159.9, 135.4, 135.1, 134.8, 130.4, 129.8, 128.2, 128.0, 124.4, 123.3 (q, J = 283.6 Hz), 119.4, 114.7, 113.5, 83.2 (q, J = 30.6 Hz), 55.4, 31.2 ppm; ^{19}**F-NMR** (565 MHz, $CDCl_3$): δ –79.4 ppm; **HRMS-ESI**: *m/z* calculated for $[C_{17}H_{13}F_3NaO_3]^+$ ($[M+Na]^+$) 345.0709, found 345.0707; **IR** (ATR): $\tilde{\nu}$ = 1739 (s) cm^{-1} (C=O).

3-(*m*-Tolyl)-3-(trifluoromethyl)isochroman-1-one (3ae)

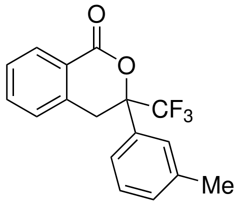

3ae

The reaction of 2-methylbenzoyl fluoride **4a** (124 mg, 0.90 mmol) and 2,2,2-trifluoro-1-(*m*-tolyl)ethan-1-one (56 mg, 0.30 mmol) following General Procedure B afforded after column chromatography (*n*-pentane/diethyl ether 9:1) isochroman-1-one **3ae** as a yellow oil (90 mg, 0.29 mmol, 98%).

R$_f$: 0.30 (*n*-pentane/diethyl ether 4:1); ^{1}**H-NMR** (600 MHz, $CDCl_3$): δ 7.97 (dd, J = 8.1, 1.3 Hz, 1H), 7.49 (td, J = 7.6, 1.4 Hz, 1H), 7.35 (s, 1H), 7.32–7.26 (m, 3H), 7.18 (t, J = 7.7 Hz, 1H), 7.09 (d, J = 7.6 Hz, 1H), 3.81 (d, J = 16.3 Hz, 1H), 3.69 (d, J = 16.3 Hz, 1H), 2.28 (s, 3H) ppm; ^{13}**C-NMR** (151 MHz, $CDCl_3$): δ 162.5, 138.6, 135.5, 134.7, 133.5, 130.4, 130.4, 128.6, 128.2, 128.0, 127.7, 124.4, 124.2, 123.4 (q, J = 283.7 Hz), 83.3 (q, J = 30.5 Hz), 31.1, 21.5 ppm; ^{19}**F-NMR** (565 MHz, $CDCl_3$): δ –79.4 ppm; **HRMS-ESI**: *m/z* calculated for $[C_{17}H_{13}F_3NaO_2]^+$ ($[M+Na]^+$) 329.0760, found 329.0751; **IR** (ATR): $\tilde{\nu}$ = 1732 (vs) cm^{-1} (C=O).

3-(3-Fluorophenyl)-3-(trifluoromethyl)isochroman-1-one (3af)

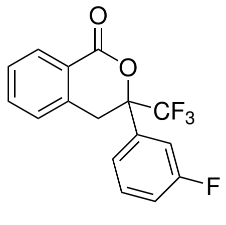

3af

The reaction of 2-methylbenzoyl fluoride **4a** (124 mg, 0.90 mmol) and 2,2,2-trifluoro-1-(3-fluorophenyl)ethan-1-one (58 mg, 42 μL, 0.30 mmol) following General Procedure B afforded after column chromatography (n-pentane/diethyl ether 9:1) isochroman-1-one **3af** as a white solid (73 mg, 0.24 mmol, 78%).

$\mathbf{R}_f$: 0.23 (n-pentane/diethyl ether 4:1); ^{1}**H-NMR** (600 MHz, $CDCl_3$): δ 7.97 (d, J = 7.8 Hz, 1H), 7.50 (td, J = 7.6, 1.6 Hz, 1H), 7.32–7.26 (m, 4H), 7.25–7.22 (m, 1H), 7.01–6.96 (m, 1H), 3.82 (d, J = 16.4 Hz, 1H), 3.63 (d, J = 16.4 Hz, 1H) ppm; ^{13}**C-NMR** (151 MHz, $CDCl_3$): δ 162.9 (d, J = 248.0 Hz), 162.1, 136.2 (d, J = 6.9 Hz), 135.1, 135.0, 130.6, 130.5 (d, J = 8.2 Hz), 128.4, 128.0, 124.3, 123.2 (q, J = 283.5 Hz), 122.9 (d, J = 3.0 Hz), 116.9 (d, J = 20.8 Hz), 114.7 (d, J = 24.3 Hz), 82.8 (q, J = 31.1 Hz), 31.2 (q, J = 1.5 Hz) ppm; ^{19}**F-NMR** (565 MHz, $CDCl_3$): δ –79.4, –111.1 (q, J = 7.9 Hz) ppm.

The characterization data agree with literature precedents.[8]

3-(*p*-Tolyl)-3-(trifluoromethyl)isochroman-1-one (3ag)

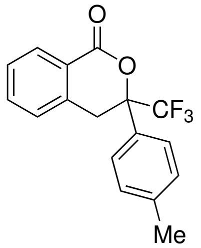

3ag

The reaction of 2-methylbenzoyl fluoride **4a** (124 mg, 0.90 mmol) and 2,2,2-trifluoro-1-(4-fluorophenyl)ethan-1-one (56 mg, 46 μL, 0.30 mmol) following General Procedure B afforded after column chromatography (n-pentane/diethyl ether 9:1) isochroman-1-one **3ag** as a white solid (91 mg, 0.29 mmol, 99%).

$\mathbf{R}_f$: 0.28 (n-pentane/diethyl ether 4:1); ^{1}**H-NMR** (600 MHz, $CDCl_3$): δ 7.96 (d, J = 8.1 Hz, 1H), 7.49 (td, J = 7.5, 1.4 Hz, 1H), 7.40 (d, J = 8.1 Hz, 2H), 7.28 (t, J = 7.3 Hz, 2H), 7.10 (d, J = 8.1 Hz, 2H), 3.81 (d, J = 16.3 Hz, 1H), 3.68 (d, J = 16.3 Hz, 1H), 2.25 (s, 3H) ppm; ^{13}**C-NMR** (151 MHz, $CDCl_3$): δ 162.6, 139.7, 135.6, 134.7, 130.5, 130.4, 129.5, 128.2, 128.0,

127.1, 124.5, 123.4 (q, J = 283.4 Hz), 83.3 (q, J = 30.6 Hz), 31.1, 21.1 ppm; ^{19}**F-NMR** (565 MHz, $CDCl_3$): δ –79.7 ppm.

The characterization data agree with literature precedents.[5,7]

3-(4-Fluorophenyl)-3-(trifluoromethyl)isochroman-1-one (3ah)

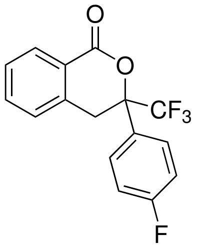

3ah

The reaction of 2-methylbenzoyl fluoride **4a** (124 mg, 0.90 mmol) and 2,2,2-trifluoro-1-(p-tolyl)ethan-1-one (58 mg, 42 μL, 0.30 mmol) following General Procedure B afforded after column chromatography (n-pentane/diethyl ether 9:1) isochroman-1-one **3ag** as a yellow oil (37 mg, 0.12 mmol, 40%).

^{1}**H-NMR** (400 MHz, $CDCl_3$): δ 7.98 (d, J = 7.8 Hz, 1H), 7.55–7.47 (m, 1H), 7.35–7.23 (m, 5H), 7.05–6.96 (m, 1H), 3.84 (d, J = 16.4 Hz, 1H), 3.62 (d, J = 16.4 Hz, 1H) ppm; ^{19}**F-NMR** (376 MHz, $CDCl_3$): δ –79.4 , –111.1 ppm.

The characterization data agree with literature precedents.[5,7]

3-(4-Chlorophenyl)-3-(trifluoromethyl)isochroman-1-one (3ai)

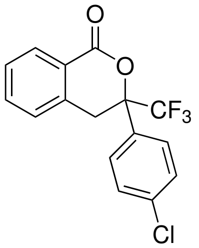

3ai

The reaction of 2-methylbenzoyl fluoride **4a** (124 mg, 0.90 mmol) and 1-(4-chlorophenyl)-2,2,2-trifluoroethan-1-one (63 mg, 45 μL, 0.30 mmol) following General Procedure B afforded after column chromatography (cyclohexane/methyl t-butyl ether 9:1) isochroman-1-one **3ai** as a pale yellow oil (90 mg, 0.29 mmol, 99%).

$\mathbf{R}_f$: 0.27 (cyclohexane/methyl t-butyl ether 4:1); ^{1}**H-NMR** (600 MHz, $CDCl_3$): δ 7.96 (d, J = 7.8 Hz, 1H), 7.51 (td, J = 7.6, 1.4 Hz, 1H), 7.46 (d, J = 8.6 Hz, 2H), 7.31 (t, J = 7.7 Hz, 1H), 7.29–7.26 (m, 3H), 3.83 (d, J = 16.4 Hz, 1H), 3.65 (d, J = 16.4 Hz, 1H) ppm; ^{13}**C-NMR** (151 MHz, $CDCl_3$): δ 162.1, 136.0, 135.1, 134.9, 132.2, 130.5, 129.1, 128.6, 128.4, 128.0, 124.3,

123.1 (q, J = 283.7 Hz), 82.9 (q, J = 30.8 Hz), 31.0 ppm; ^{19}**F-NMR** (565 MHz, $CDCl_3$): δ –79.6 ppm.

The characterization data agree with literature precedents.[5,7,8]

3-(4-Bromophenyl)-3-(trifluoromethyl)isochroman-1-one (3aj)

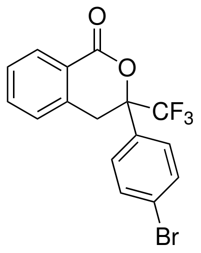

3aj

The reaction of 2-methylbenzoyl fluoride **4a** (124 mg, 0.90 mmol) and 1-(4-bromophenyl)-2,2,2-trifluoroethan-1-one (76 mg, 46 μL, 0.30 mmol) following General Procedure B afforded after column chromatography (n-pentane/diethyl ether 9:1) isochroman-1-one **3aj** as a white solid (59 mg, 0.16 mmol, 53%).

R$_f$: 0.24 (n-pentane/diethyl ether 5:1); ^{1}**H-NMR** (500 MHz, $CDCl_3$): δ 7.97 (dd, J = 7.8, 1.5 Hz, 1H), 7.51 (td, J = 7.5, 1.5 Hz, 1H), 7.46–7.42 (m, 2H), 7.41–7.38 (m, 2H), 7.35–7.30 (m, 1H), 7.27 (d, J = 7.6 Hz, 1H), 3.83 (d, J = 16.3 Hz, 1H), 3.64 (d, J = 16.3 Hz, 1H) ppm; ^{13}**C-NMR** (126 MHz, $CDCl_3$): δ 162.1, 135.1, 135.0, 132.8, 132.1, 130.6, 128.9, 128.5, 128.0, 124.31, 124.29, 124.2 (q, J = 283.9 Hz), 83.0 (q, J = 30.6 Hz), 31.0 (d, J = 1.6 Hz) ppm; ^{19}**F-NMR** (471 MHz, $CDCl_3$): δ –79.6 ppm.

The characterization data agree with literature precedents.[5,7]

3-(3,5-Dimethylphenyl)-3-(trifluoromethyl)isochroman-1-one (3ak)

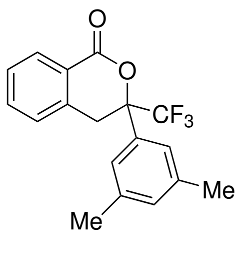

3ak

The reaction of 2-methylbenzoyl fluoride **4a** (124 mg, 0.90 mmol) and 1-(3,5-dimethylphenyl)-2,2,2-trifluoroethan-1-one (61 mg, 0.30 mmol) following General Procedure B afforded after column chromatography (n-pentane/diethyl ether 9:1) isochroman-1-one **3ak** as a white solid (90 mg, 0.28 mmol, 94%).

R$_f$: 0.35 (n-pentane/diethyl ether 4:1); ^{1}**H-NMR** (600 MHz, $CDCl_3$): δ 7.98 (d, J = 7.8

Hz, 1H), 7.49 (td, J = 7.6, 1.4 Hz, 1H), 7.33–7.24 (m, 2H), 7.12 (s, 2H), 6.91 (s, 1H), 3.79 (d, J = 16.3 Hz, 1H), 3.67 (d, J = 16.3 Hz, 1H), 2.25 (s, 6H) ppm; ^{13}**C-NMR** (151 MHz, $CDCl_3$): δ 162.6, 138.4, 135.6, 134.7, 133.5, 131.4, 130.4, 128.1, 128.0, 124.9, 124.5, 123.5 (q, J = 283.6 Hz), 83.3 (q, J = 30.5 Hz), 31.2, 21.4 ppm; ^{19}**F-NMR** (565 MHz, $CDCl_3$): δ –79.4 ppm; **HRMS-ESI**: m/z calculated for $[C_{18}H_{15}F_3NaO_2]^+$ ($[M+Na]^+$) 343.0916, found 343.0896; **IR** (ATR): $\tilde{\nu}$ = 1733 (s) cm^{-1} (C=O).

3-(3,5-Difluorophenyl)-3-(trifluoromethyl)isochroman-1-one (3al)

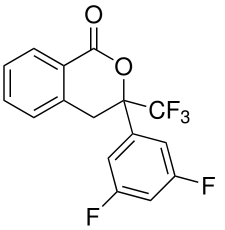

3al

The reaction of 2-methylbenzoyl fluoride **4a** (124 mg, 0.90 mmol) and 1-(3,5-difluorophenyl)-2,2,2-trifluoroethan-1-one (63 mg, 43 μL, 0.30 mmol) following General Procedure B afforded after column chromatography (*n*-pentane/diethyl ether 9:1) isochroman-1-one **3al** as a white solid (46 mg, 0.14 mmol, 47%).

$\mathbf{R}_f$: 0.29 (*n*-pentane/diethyl ether 4:1); ^{1}**H-NMR** (600 MHz, $CDCl_3$): δ 8.01 (d, J = 7.8 Hz, 1H), 7.55 (td, J = 7.5, 1.4 Hz, 1H), 7.36 (t, J = 7.6 Hz, 1H), 7.29 (d, J = 7.6 Hz, 1H), 7.11–7.04 (m, 2H), 6.77 (tt, J = 8.5, 2.3 Hz, 1H), 3.84 (d, J = 16.4 Hz, 1H), 3.57 (d, J = 16.4 Hz, 1H) ppm; ^{13}**C-NMR** (126 MHz, $CDCl_3$): δ 163.2 (dd, J = 250.7, 12.5 Hz), 161.7, 137.8 (t, J = 9.1 Hz), 135.1, 134.7, 130.7, 128.7, 128.0 (d, J = 3.7 Hz), 124.1, 123.0 (q, J = 283.7 Hz), 110.8 (dd, J = 21.1, 7.3 Hz), 105.6 (t, J = 25.2 Hz), 82.5 (q, J = 30.9 Hz), 31.2 ppm; ^{19}**F-NMR** (565 MHz, $CDCl_3$): δ –79.3, –107.3 (t, J = 7.9 Hz) ppm; **HRMS-ESI**: m/z calculated for $[C_{16}H_9F_5NaO_2]^+$ ($[M+Na]^+$) 351.0415, found 351.0403; **IR** (ATR): $\tilde{\nu}$ = 1741 (m) cm^{-1} (C=O).

5.3 Limitations of the NHC-catalyzed PEDA Reaction

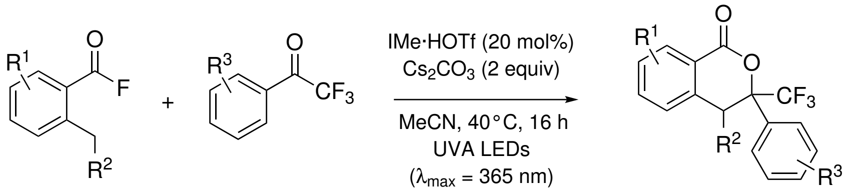

4 (3 equiv) **2** (1 equiv) **3**

Entry	R^1	R^2	R^3	Yield (NMR)
1	3-OAc	H	H	**3** (traces)
2	3-NO_2	H	H	**3** (4%)
3	5-NO_2	H	H	**3** (4%)
4	3,5-NO_2	H	H	**3** (traces)
5	H	SPh	H	–
6	H	OCOPh	H	–
7	H	H	4-NMe_2	–
8	H	H	Methyl benzoylformate	–
9	H	H	*N*-Methylisatin	–

6 UV-Vis Studies

A comparison of the UV-Vis spectra of trifluoroacetophenone **2a**, *ortho*-toluoyl fluoride **4a** and the *ortho*-toluoyl azolium salt **1** indicates that only the azolium salt absorbs significantly at the irradiation wavelength of 365 nm with the cut-off at ca. 400 nm (Figure S5). Conversely, ketone **2a** absorbs significantly up to just below the irradiation wavelength (Figure S7) and acid fluoride **4a** up to ca. 320 nm (Figure S9).

In order to assess the relative absorbance of each component in the catalytic reaction mixture at the irradiation wavelength (365 nm), UV-Vis spectra were measured of combinations of **4a** and **2a** at their relative reaction stoichiometries with and without the *ortho*-toluoyl azolium salt **1** (Figures S11 and S12). A reaction concentration of the catalytically generated intermediate (**1**) of 10 mol% (equivalent to 50% of the NHC catalyst loading) was assumed. This revealed that, even with large excesses of **2a** and **4a**, the majority of the light at 365 nm (ca. 90%) is absorbed by the *ortho*-toluoyl azolium salt **1**. Although these results suggest that direct excitation of the catalytically generated azolium intermediate is a feasible reaction pathway under the catalytic reaction conditions, triplet sensitization from either ketone **2a** or potentially even acid fluoride **4a** cannot be conclusively ruled out.

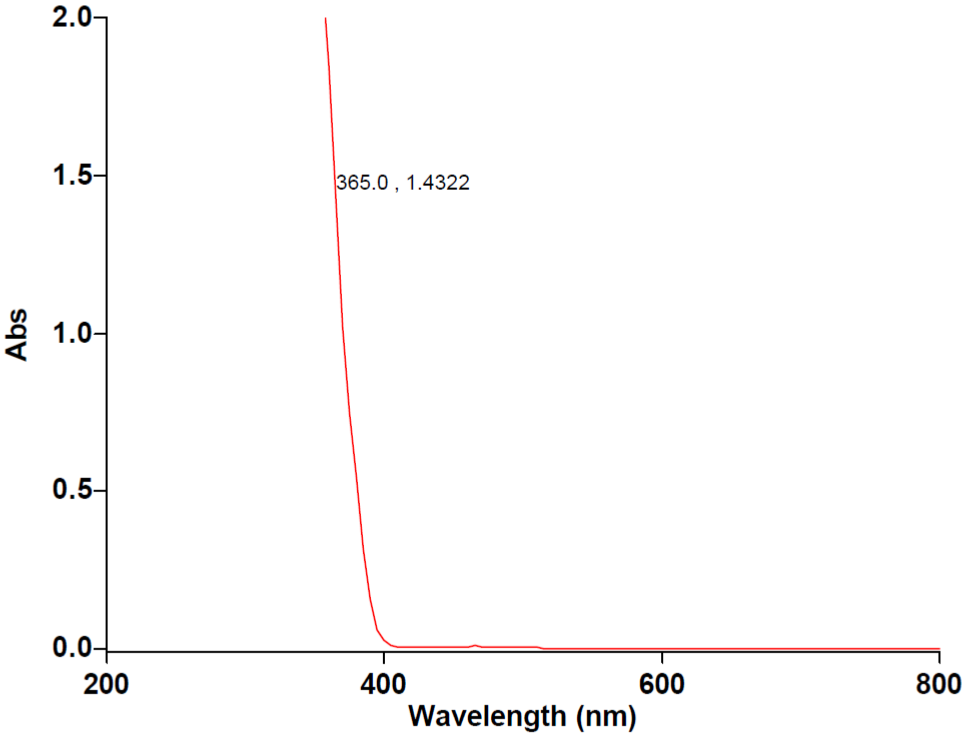

Figure S5: UV-Vis spectrum of imidazolium salt **1** in MeCN (10^{-2} M).

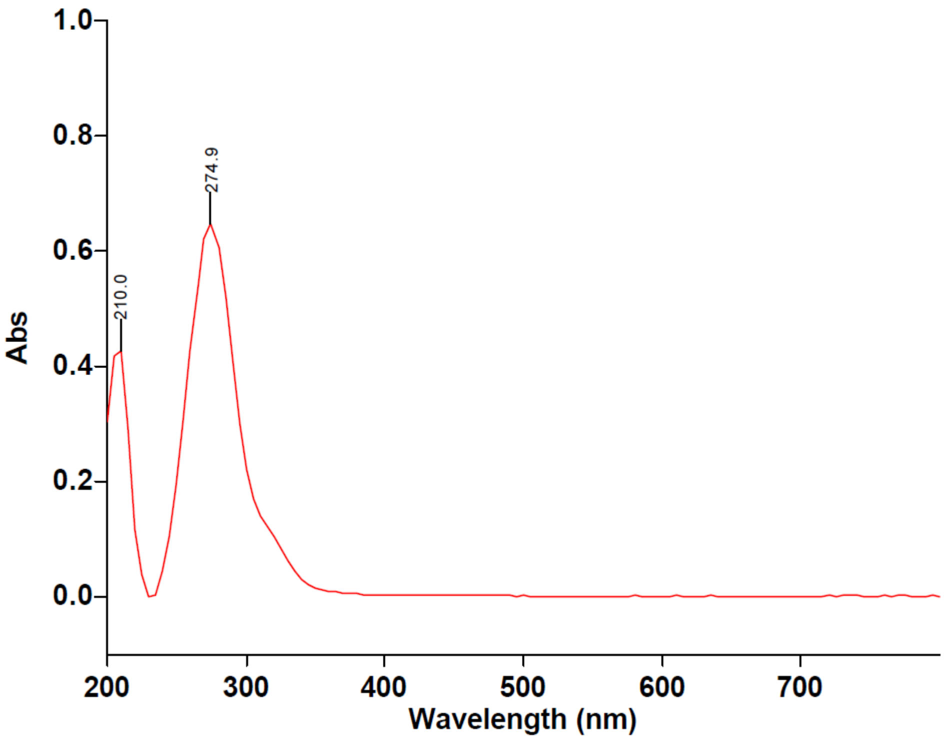

Figure S6: UV-Vis spectrum of imidazolium salt **1** in MeCN (10^{-5} M).

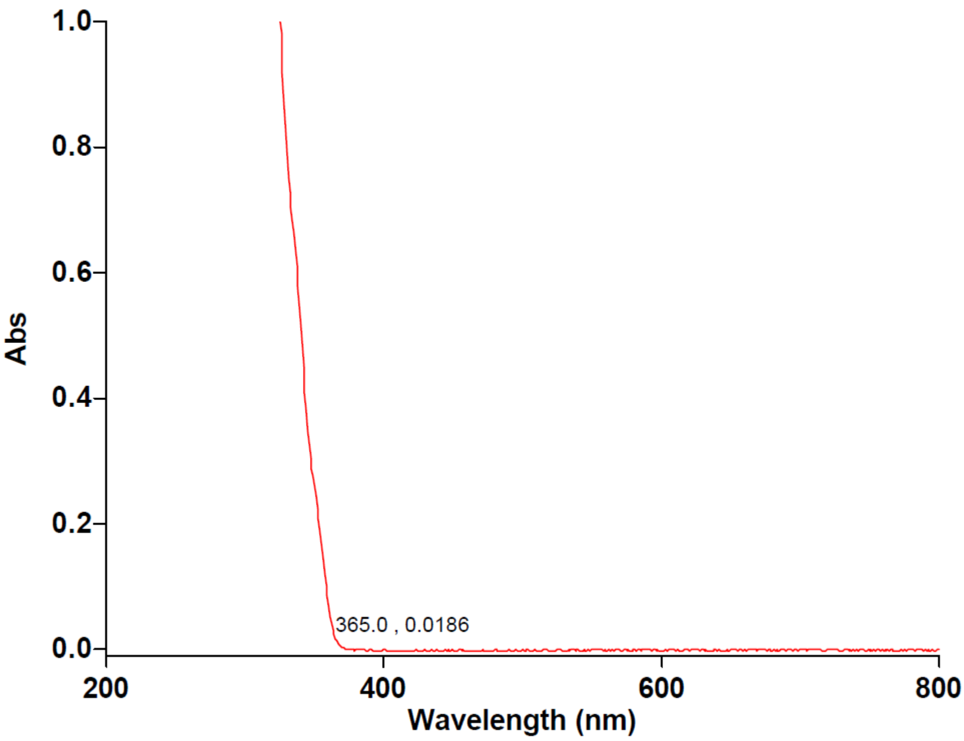

Figure S7: UV-Vis spectrum of ketone **2a** in MeCN (10^{-2} M).

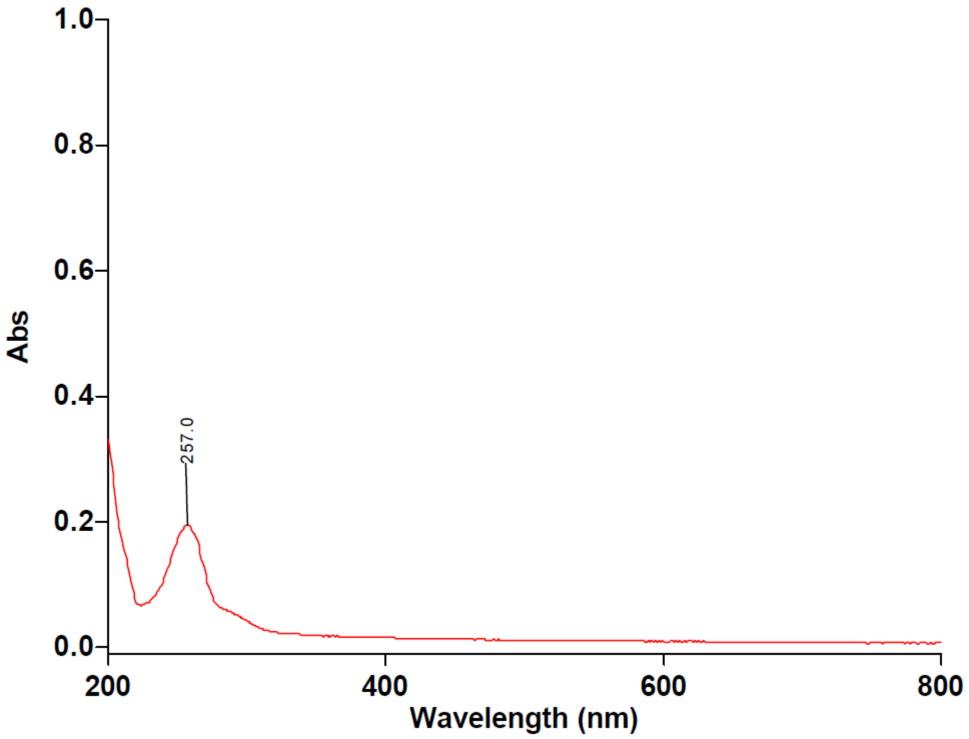

Figure S8: UV-Vis spectrum of ketone **2a** in MeCN (10^{-5} M).

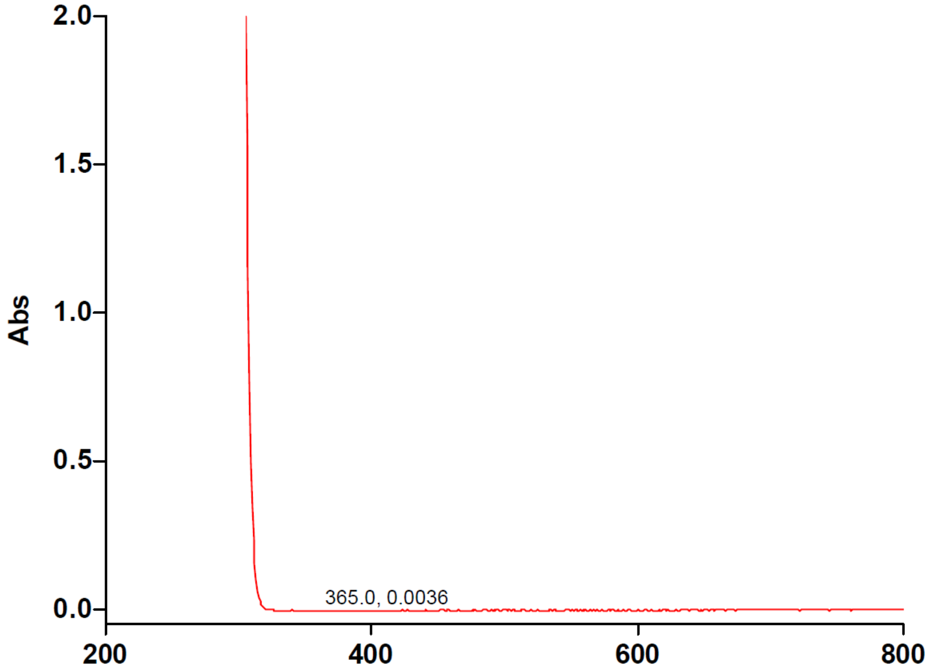

Figure S9: UV-Vis spectrum of acid fluoride **4a** in MeCN (10^{-2} M).

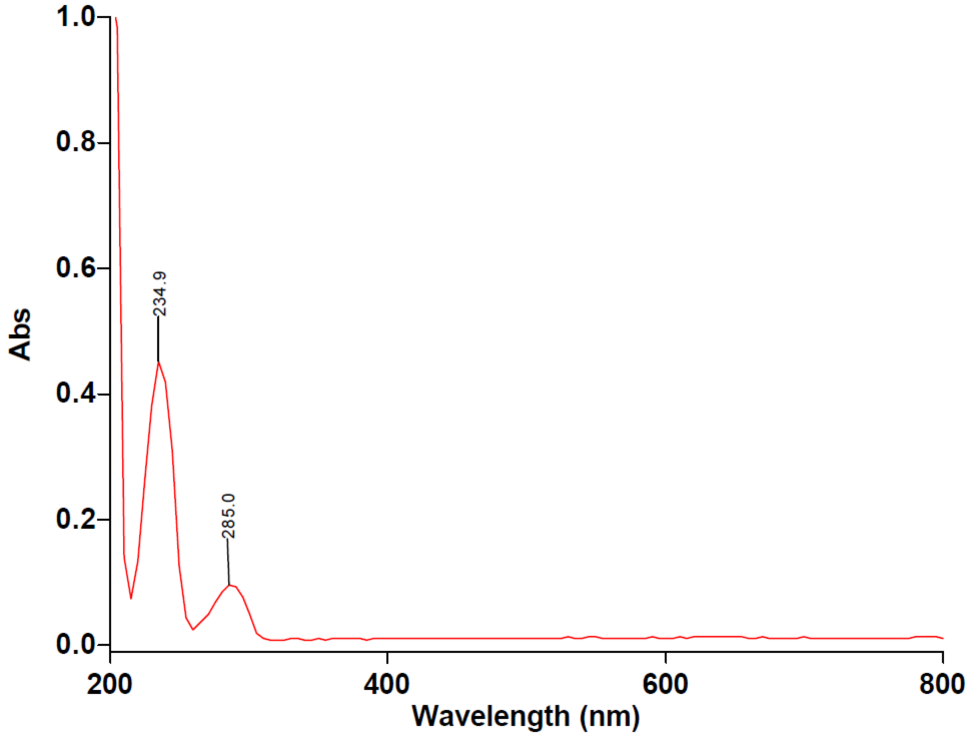

Figure S10: UV-Vis spectrum of acid fluoride **4a** in MeCN (10^{-5} M).

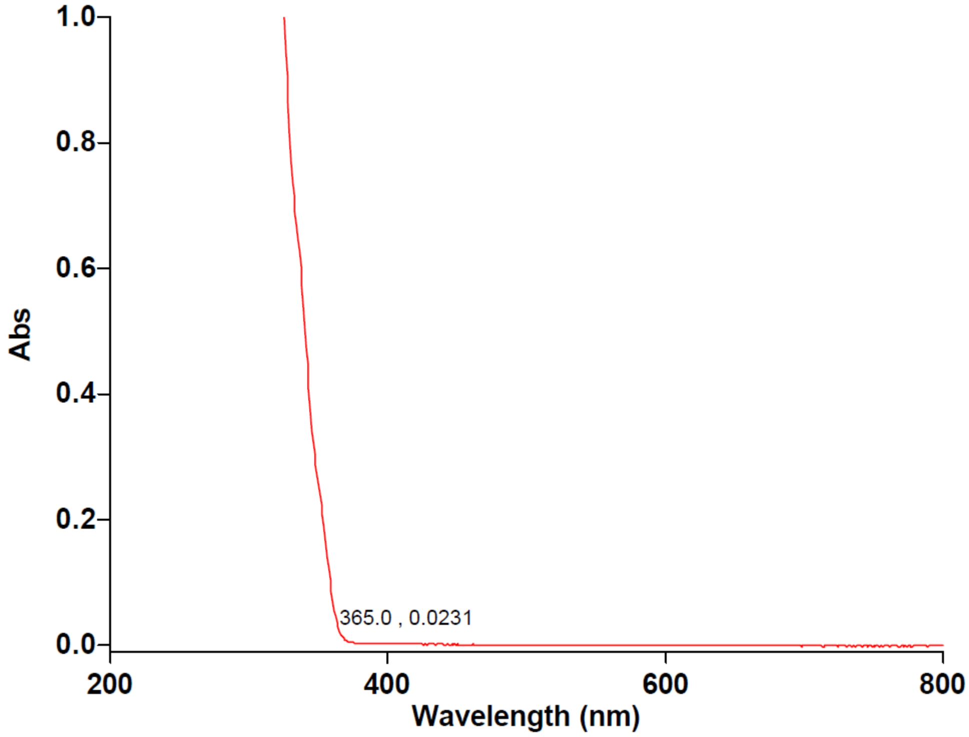

Figure S11: UV-Vis spectrum of ketone **2a** (10^{-2} M) and acid fluoride **4a** ($3*10^{-2}$ M) in MeCN.

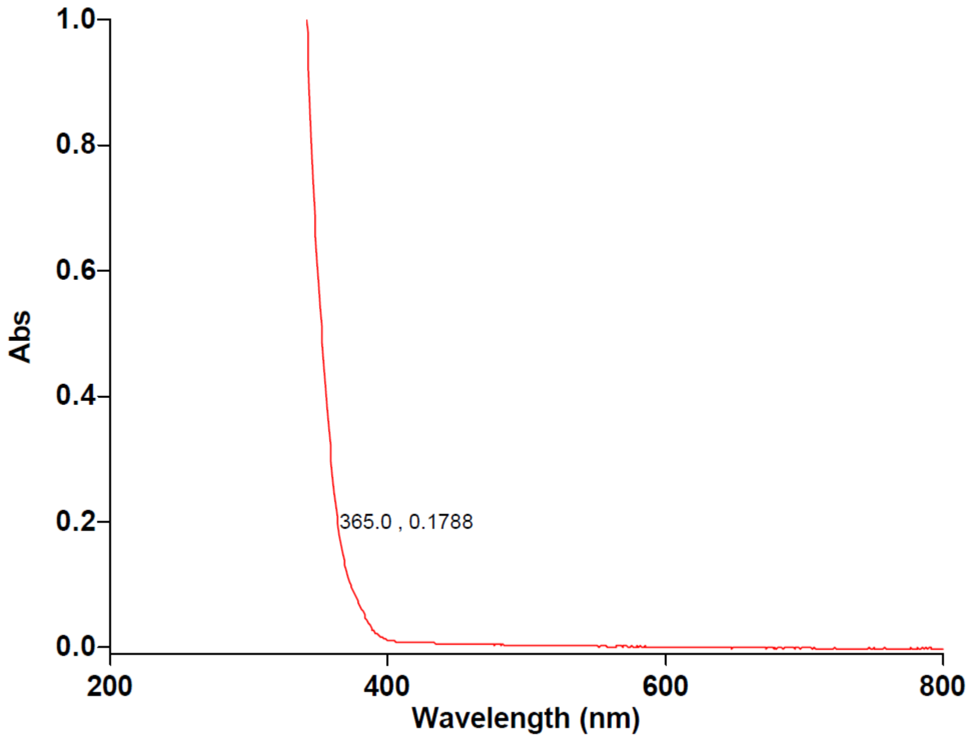

Figure S12: UV-Vis spectrum of imidazolium salt **1** (10^{-3} M), ketone **2a** (10^{-2} M) and acid fluoride **4a** ($2.9*10^{-2}$ M) in MeCN.

7 Computational Studies

Computational results were performed using the Gaussian16[9] package using the CAM-B3LYP[10] functional with a 6-311G(d,p)[11] basis set. For all investigated compounds, the singlet ground state geometries were optimized and subsequently vertical absorption spectra were obtained via time-dependent density functional theory (TD-DFT).[12,13] Triplet geometries were also optimized, without TD-DFT, starting from the optimized singlet geometries. Finally, linear scans along the proton transfer tautomerization coordinate were performed for the lowest singlet, triplet and ground state, to compare between the singlet and triplet pathways. All calculations made use of a CPCM solvation model for acetonitrile.[14] Images were created using VMD.[15] The results are summarized by the difference in electronic densities between ground and excited states (see Figure 1 main article).

We also found strongly different potential energy surfaces when comparing the $\mathbf{T_1(A)} \rightarrow \mathbf{T_1(B)}$ process to the analogous path in acid fluoride **4a** (along excited singlet and triplet surfaces). Namely, the normalized highest point in energy for the $\mathbf{T_1(A)} \rightarrow \mathbf{T_1(B)}$ process along a relaxed scan is 29.3 kJ/mol, the same process along the singlet surface faces a barrier of 42.3 kJ/mol. When we aim for the same chemistry in acid fluoride **4a**, we face a continuously uphill path when in the triplet state (no stable product) or a barrier of 20.5 kJ/mol for the lowest excited singlet state. At first glance, the singlet states of the NHC and acid fluoride compounds may also allow for a successful enolization, but this is blocked by an unproductive backwards reaction. Furthermore, the lowest singlet acid fluoride absorption is computed to be accessible only at wavelengths below 250 nm, making it inaccessible for the radiation used in our study.

8 References

[1] J. Tjutrins, B. A. Arndtsen, *J. Am. Chem. Soc.* **2015**, *137*, 12050–12054.

[2] C. A. Malapit, J. R. Bour, C. E. Brigham, M. S. Sanford, *Nature* **2018**, *563*, 100–104.

[3] Z. Wang, X. Wang, Y. Nishihara, *Chem. Commun.* **2018**, *54*, 13969–13972.

[4] N. V. Ignat'ev, P. Barthen, A. Kucheryna, H. Willner, P. Sartori, *Molecules* **2012**, *17*, 5319–5338.

[5] D. Janssen-Müller, S. Singha, T. Olyschläger, C. G. Daniliuc, F. Glorius, *Org. Lett.* **2016**, *18*, 4444–4447.

[6] K. Takaki, T. Fujii, H. Yonemitsu, M. Fujiwara, K. Komeyama, H. Yoshida, *Tetrahedron Lett.* **2012**, *53*, 3974–3976.

[7] H. Wang, X. Chen, Y. Li, J. Wang, S. Wu, W. Xue, S. Yang, Y. R. Chi, *Org. Lett.* **2018**, *20*, 333–336.

[8] D.-F. Chen, T. Rovis, *Synthesis* **2016**, *49*, 293–298.

[9] Gaussian 16, Revision C.01, M. J. Frisch et al., Gaussian, Inc. Wallingford CT, **2016**.

[10] T. Yanai, D. P. Tew, N. C. Handy, *Chem. Phys. Lett.* **2004**, *393*, 51–57.

[11] P. C. Hariharan, J. A. Pople, *Theor. Chim. Acta* **1973**, *28*, 213–222.

[12] C. Jamorski, M. E. Casida, D. R. Salahub, *J. Chem. Phys.* **1996**, *104*, 5134–5147.

[13] M. E. Casida, C. Jamorski, K. C. Casida, D. R. Salahub, *J. Chem. Phys.* **1998**, *108*, 4439–4449.

[14] M. Cossi, N. Rega, G. Scalmani, V. Barone, *J. Comput. Chem.* **2003**, *24*, 669–681.

[15] W. Humphrey, A. Dalke, K. Schulten, *J. Mol. Graph.* **1996**, *14*, 33–38.

9 NMR Spectra of Novel Compounds

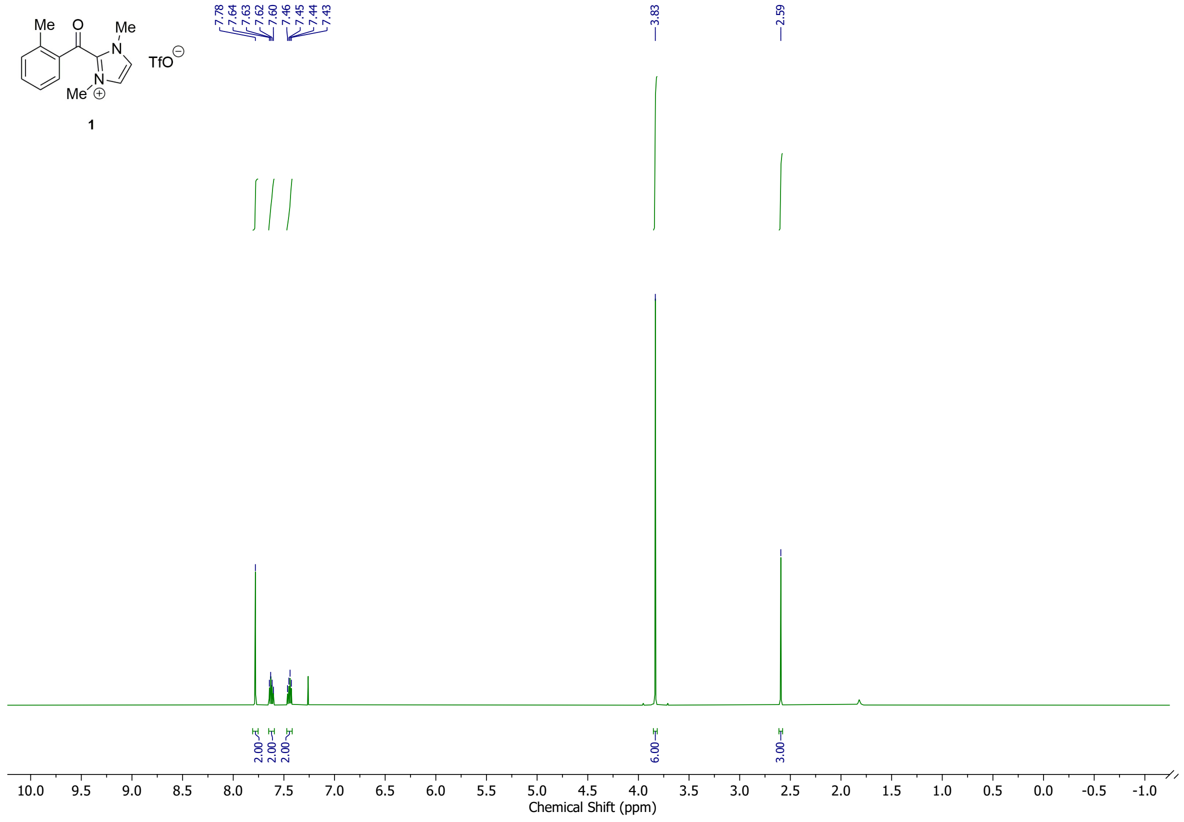

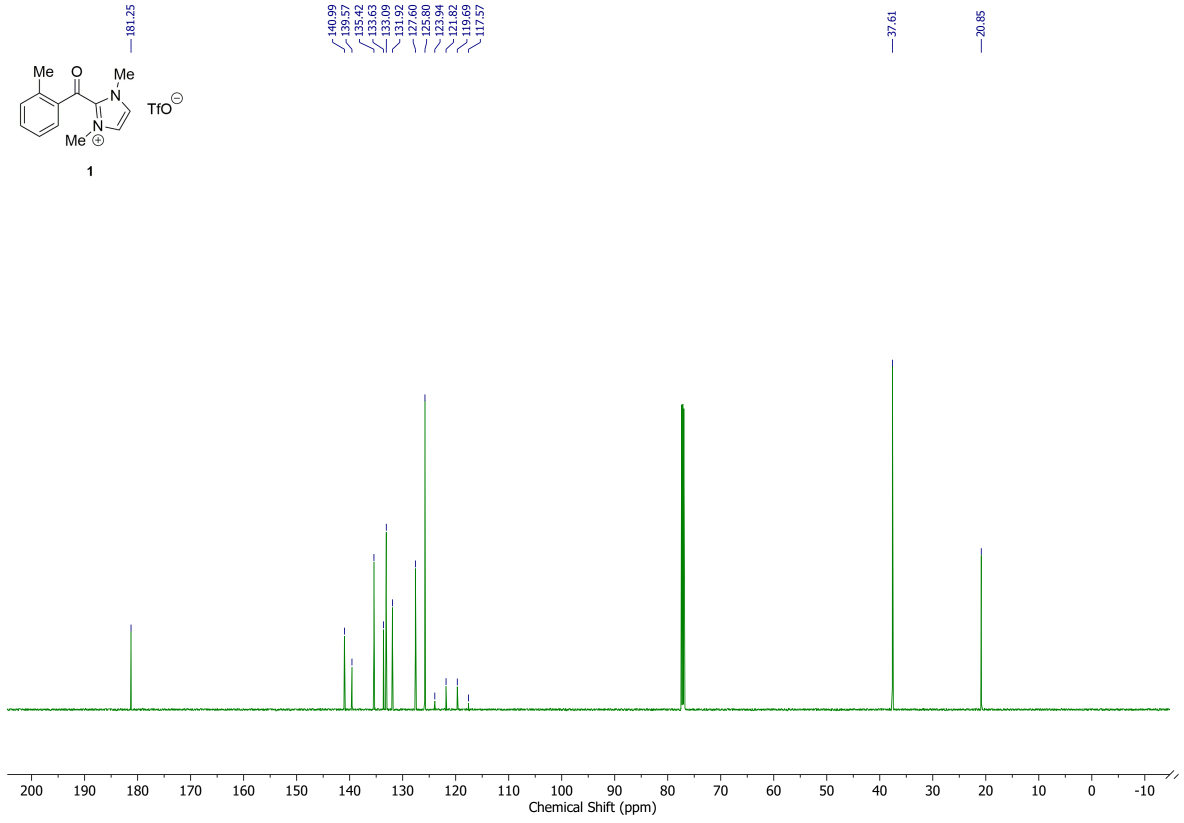

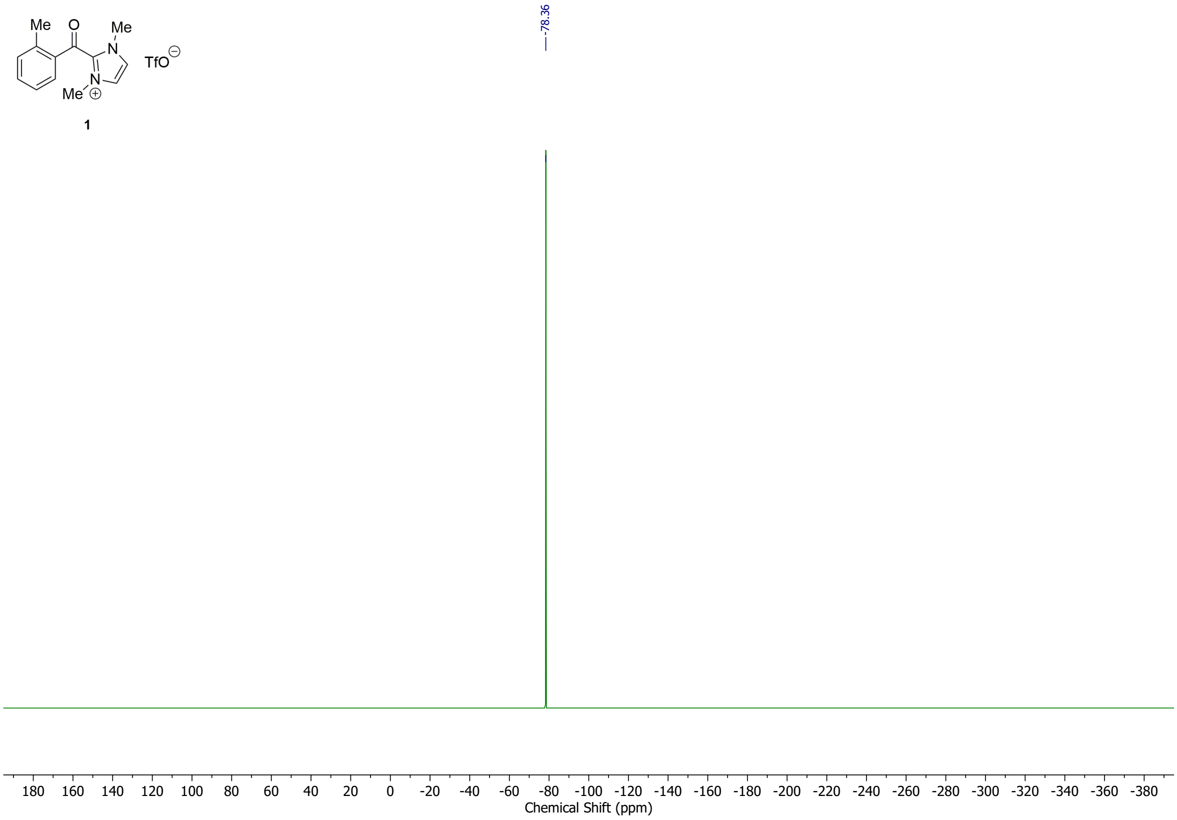
Me
O
Me
N
N
TfO
Me
1
-78.36
180 160 140 120 100 80 60 40 20 0 -20 -40 -60 -80 -100 -120 -140 -160 -180 -200 -220 -240 -260 -280 -300 -320 -340 -360 -380
Chemical Shift (ppm)

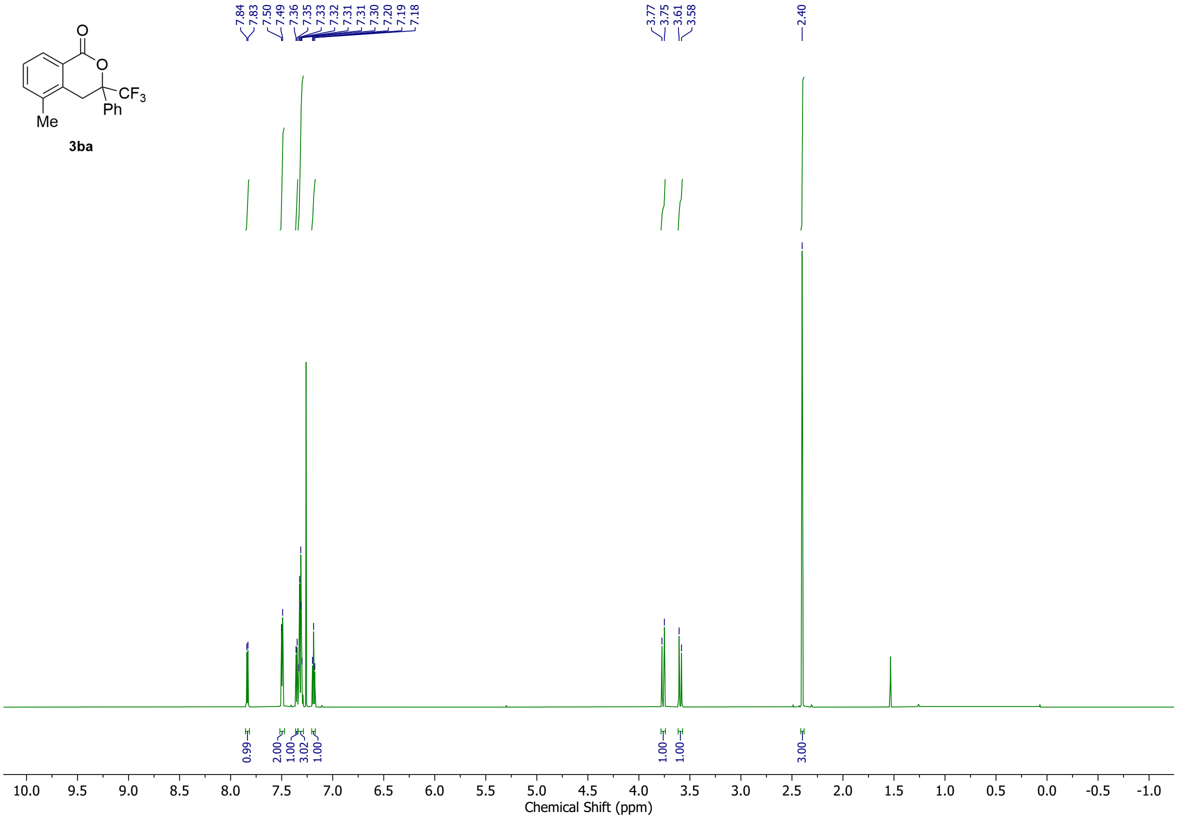
O
O
CF3
Ph
Me
3ba
7.84
7.83
7.50
7.49
7.36
7.35
7.33
7.32
7.31
7.31
7.30
7.20
7.19
7.18
3.77
3.75
3.61
3.58
2.40
0.99
2.00
1.00
3.02
1.00
1.00
1.00
3.00
10.0 9.5 9.0 8.5 8.0 7.5 7.0 6.5 6.0 5.5 5.0 4.5 4.0 3.5 3.0 2.5 2.0 1.5 1.0 0.5 0.0 -0.5 -1.0
Chemical Shift (ppm)

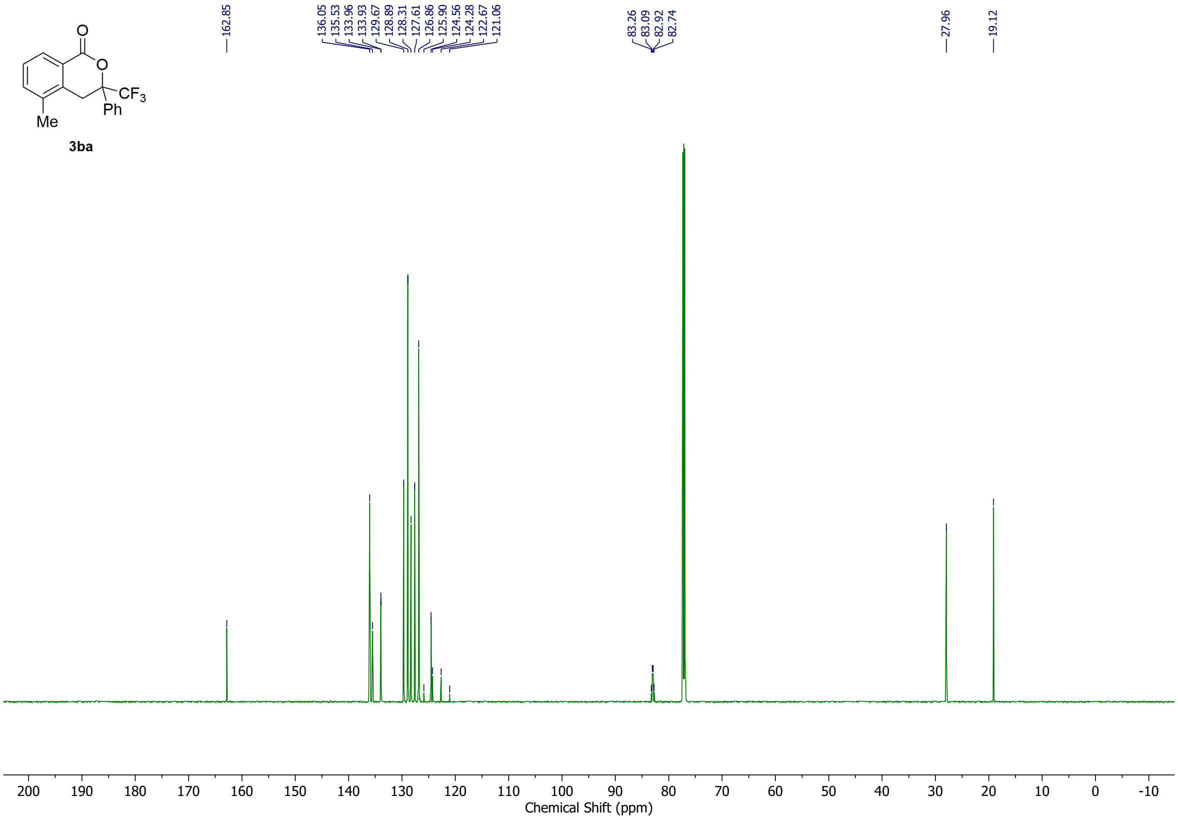
O
O
CF3
Ph
Me
3ba
162.85
136.05
135.53
133.96
133.93
129.67
128.89
128.31
127.61
126.86
125.90
124.56
124.28
122.67
121.06
83.26
83.09
82.92
82.74
27.96
19.12
Chemical Shift (ppm)

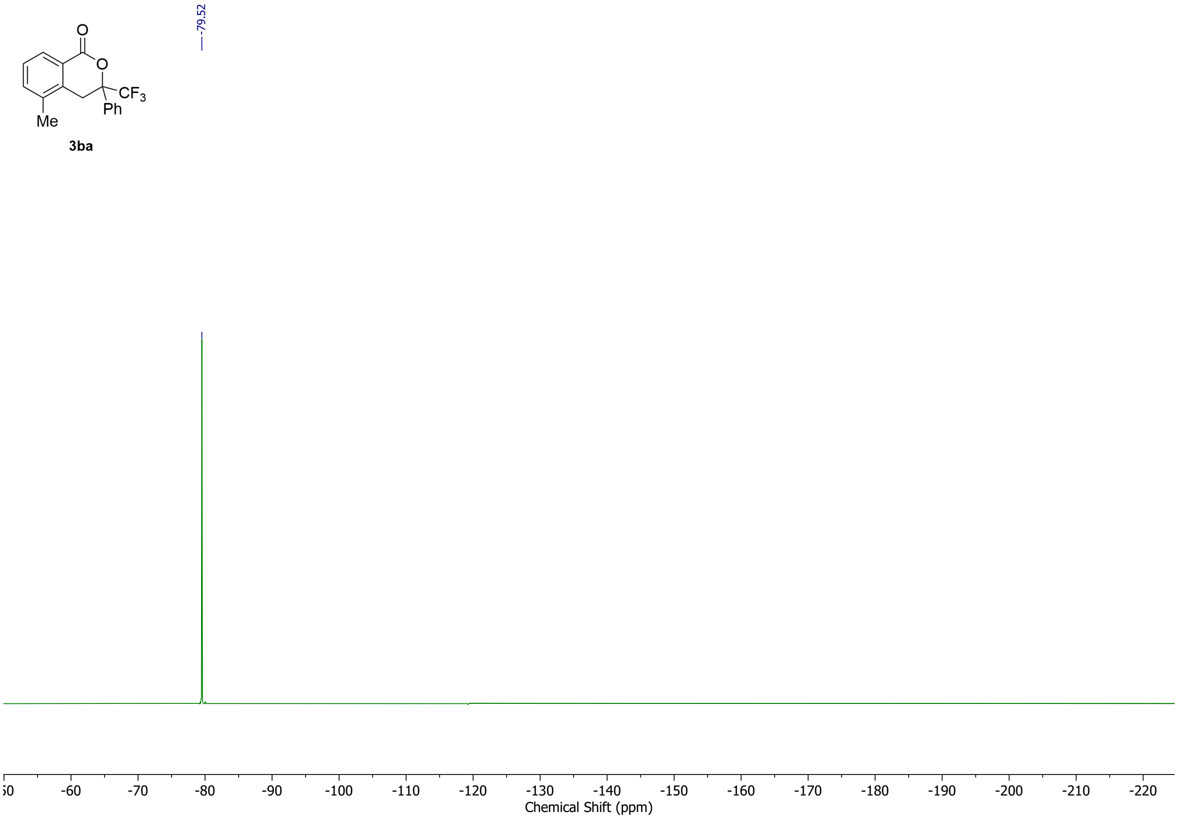
O
O
CF3
Ph
Me
3ba
-79.52
Chemical Shift (ppm)

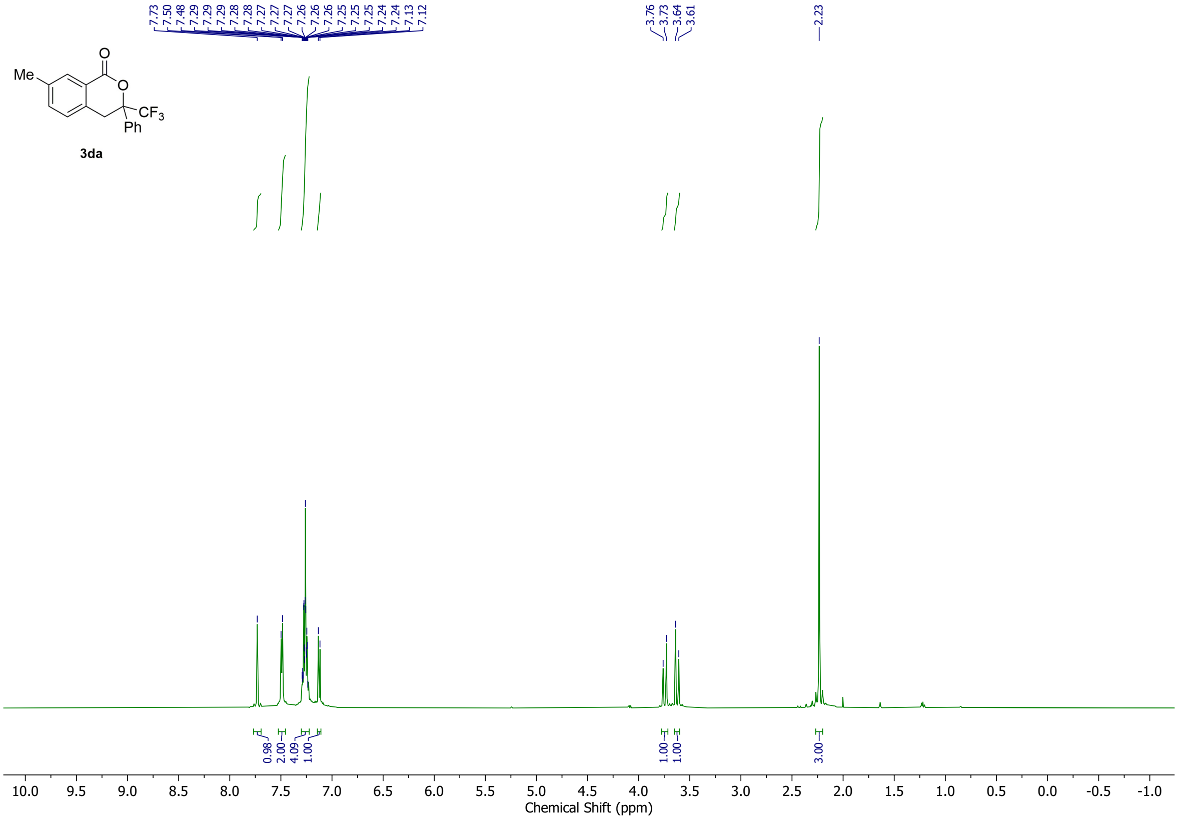
Me
O
O
CF3
Ph
3da
7.73 7.50 7.48 7.29 7.29 7.29 7.28 7.28 7.27 7.27 7.27 7.26 7.26 7.26 7.25 7.25 7.25 7.24 7.24 7.13 7.12
3.76 3.73 3.64 3.61
2.23
0.98 2.00 4.09 1.00
1.00 1.00
3.00
Chemical Shift (ppm)

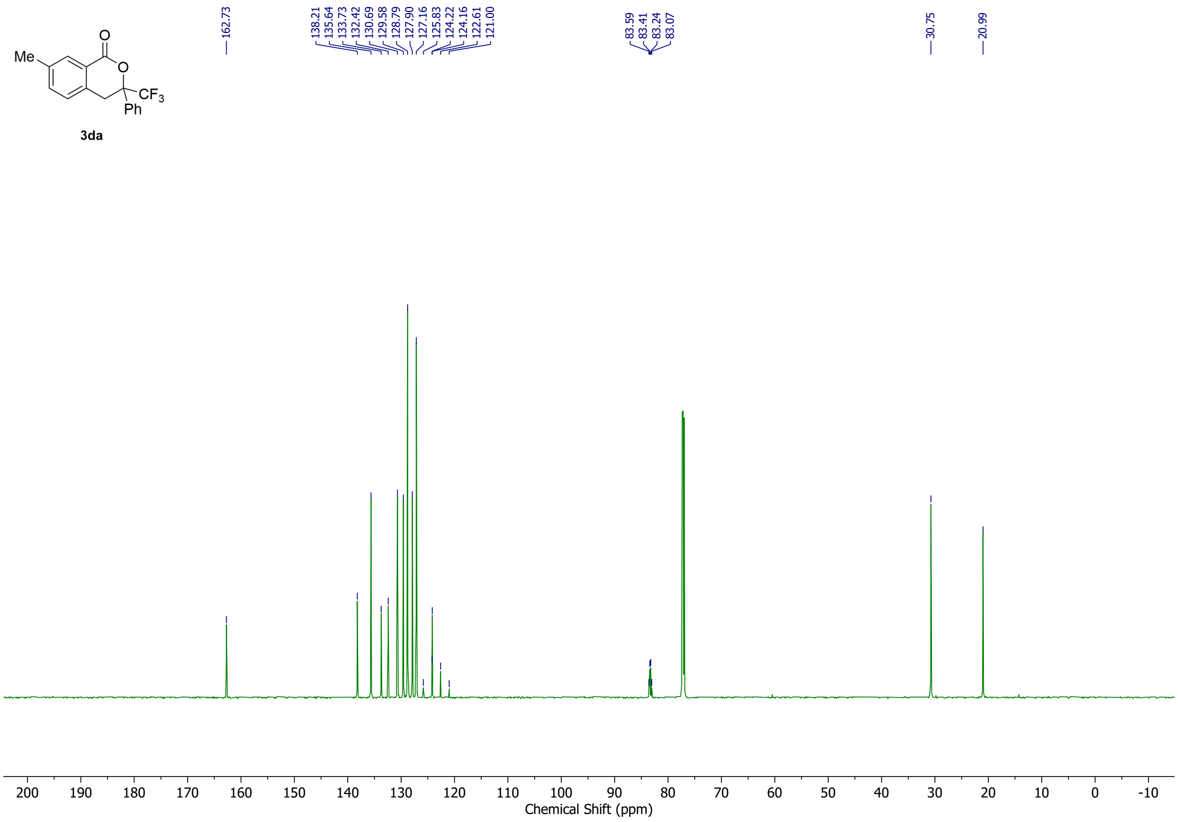
Me
O
O
CF3
Ph
3da
162.73
138.21 135.64 133.73 132.42 130.69 129.58 128.79 127.90 127.16 125.83 124.22 124.16 122.61 121.00
83.59 83.41 83.24 83.07
30.75
20.99
Chemical Shift (ppm)

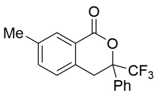

3da

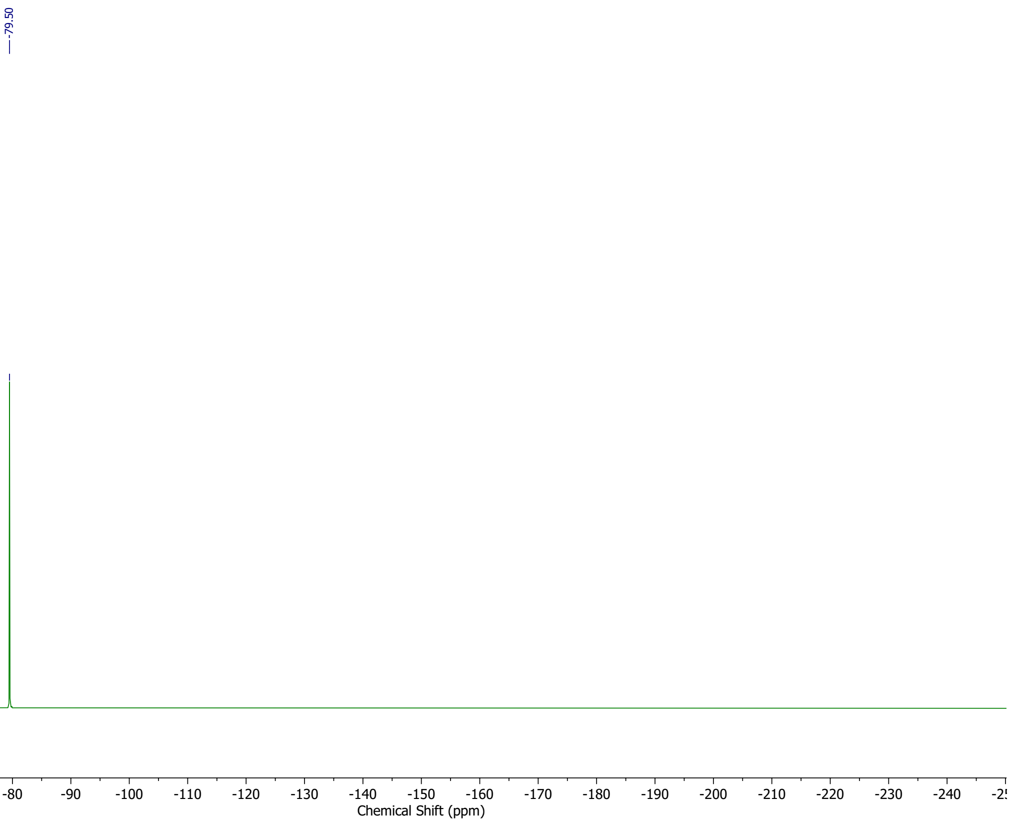
-79.50
-60
-70
-80
-90
-100
-110
-120
-130
-140
-150
-160
-170
-180
-190
-200
-210
-220
-230
-240
Chemical Shift (ppm)

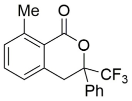

3ea

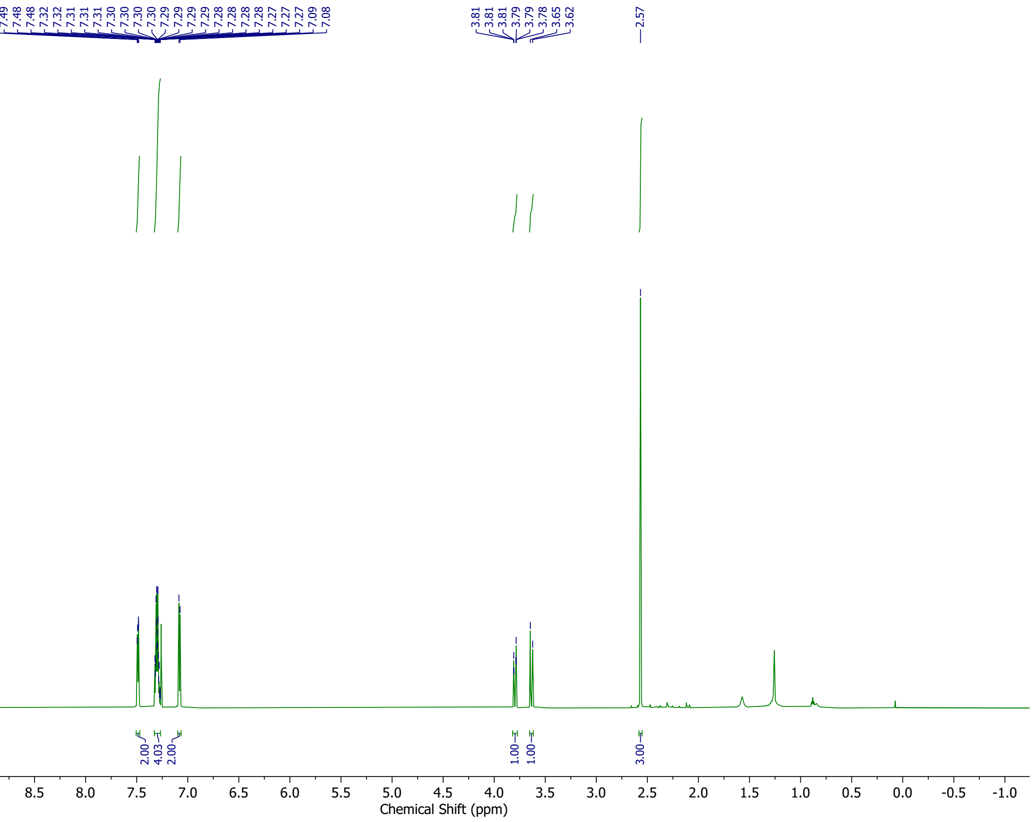
7.49
7.49
7.48
7.32
7.32
7.31
7.31
7.30
7.30
7.30
7.29
7.29
7.29
7.28
7.28
7.28
7.27
7.27
7.09
7.08
3.81
3.81
3.81
3.79
3.78
3.65
3.62
2.57
2.00
4.03
2.00
1.00
1.00
3.00
10.0
9.5
9.0
8.5
8.0
7.5
7.0
6.5
6.0
5.5
5.0
4.5
4.0
3.5
3.0
2.5
2.0
1.5
1.0
0.5
0.0
-0.5
-1.0
Chemical Shift (ppm)

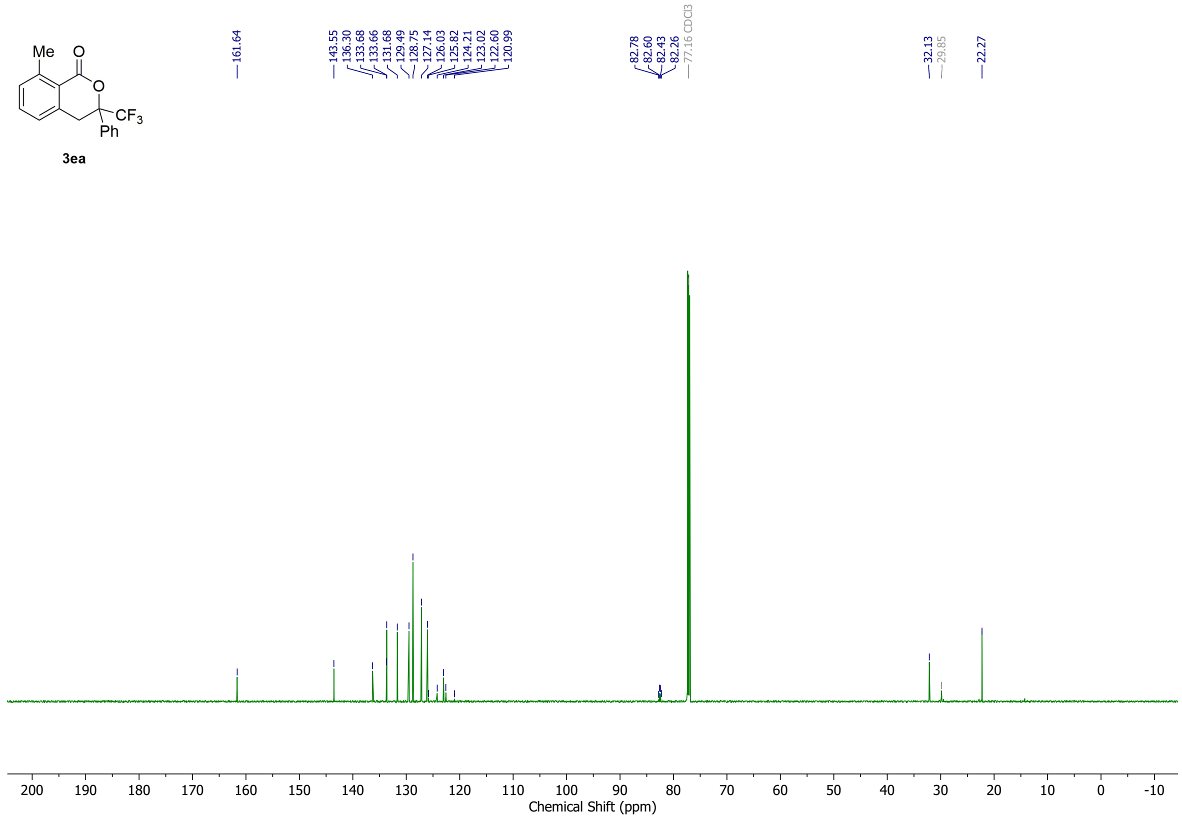
Me
O
O
CF3
Ph
3ea
161.64
143.55
136.30
133.68
133.66
131.68
129.49
128.75
127.14
126.03
125.82
124.21
123.02
122.60
120.99
82.78
82.60
82.43
82.26
77.16 CDCl3
32.13
29.85
22.27
Chemical Shift (ppm)

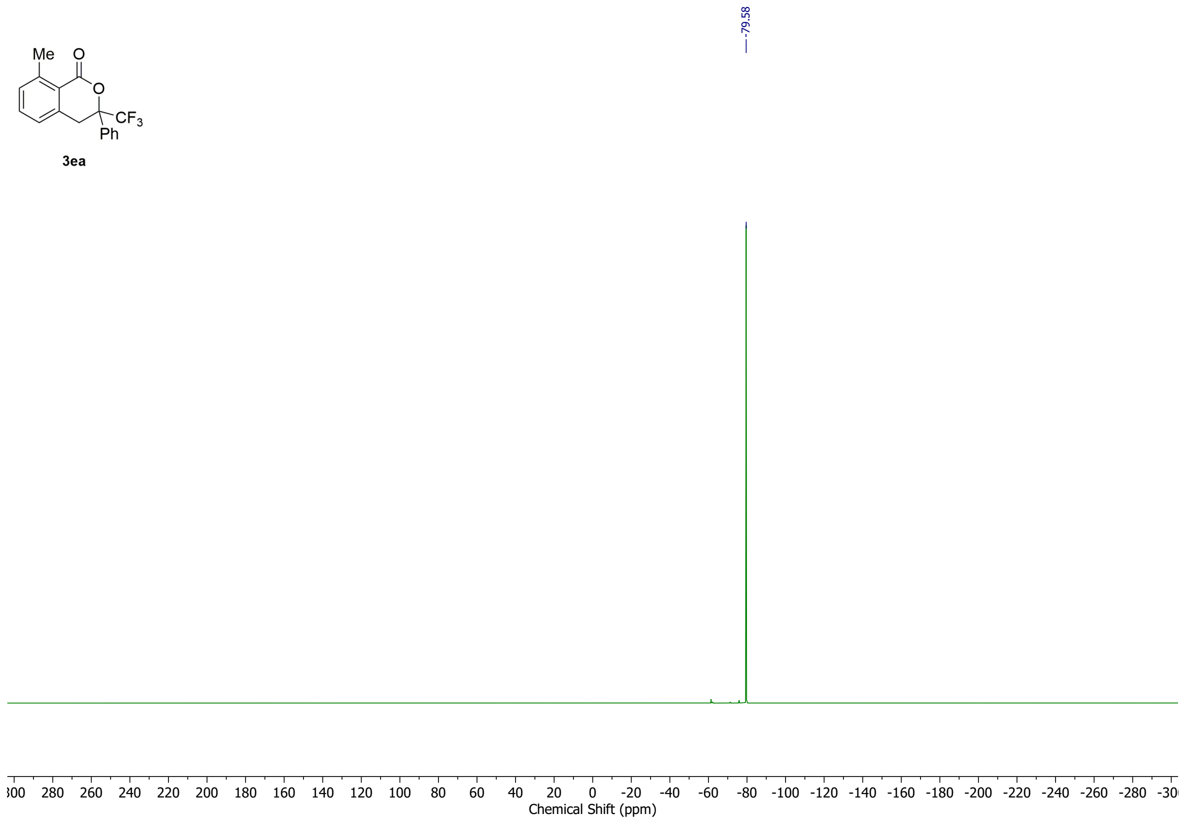
Me
O
O
CF3
Ph
3ea
-79.58
Chemical Shift (ppm)

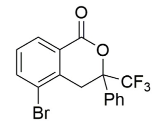
O
O
CF3
Ph
Br

3ha

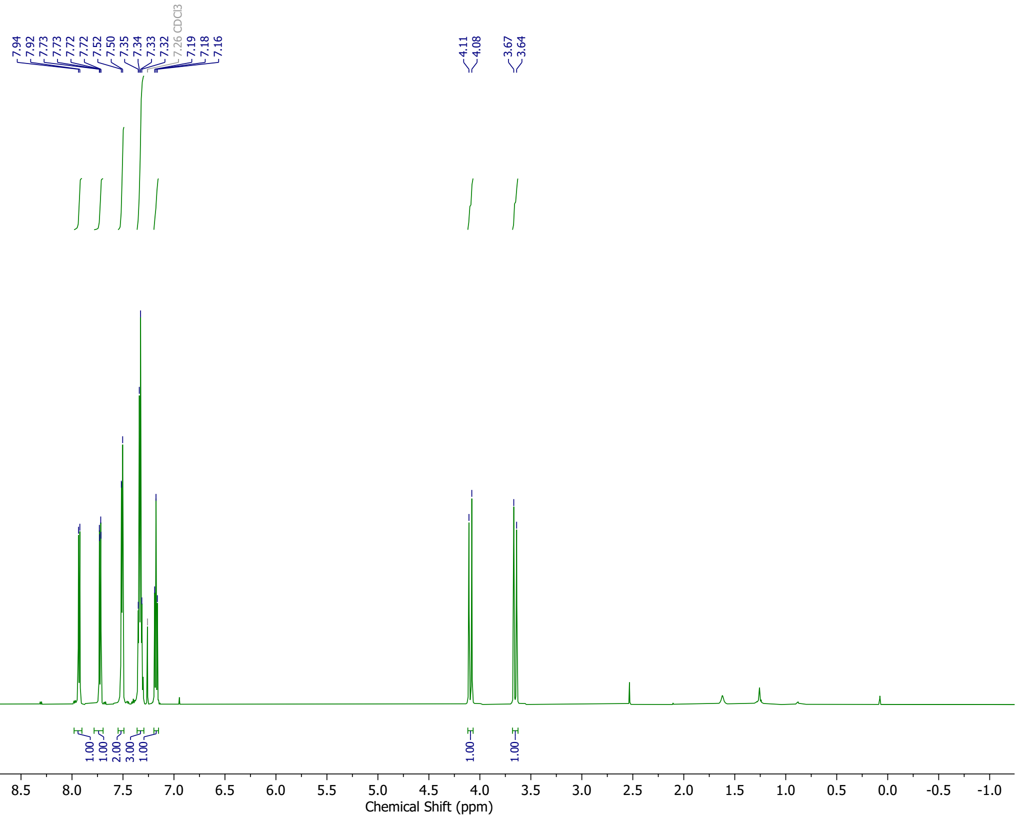
7.94
7.92
7.73
7.73
7.72
7.72
7.52
7.50
7.35
7.34
7.33
7.32
7.26 CDCl3
7.19
7.18
7.16
4.11
4.08
3.67
3.64
1.00
1.00
2.00
3.00
1.00
1.00
1.00
10.0 9.5 9.0 8.5 8.0 7.5 7.0 6.5 6.0 5.5 5.0 4.5 4.0 3.5 3.0 2.5 2.0 1.5 1.0 0.5 0.0 -0.5 -1.0
Chemical Shift (ppm)

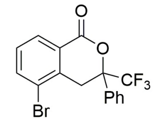
O
O
CF3
Ph
Br

3ha

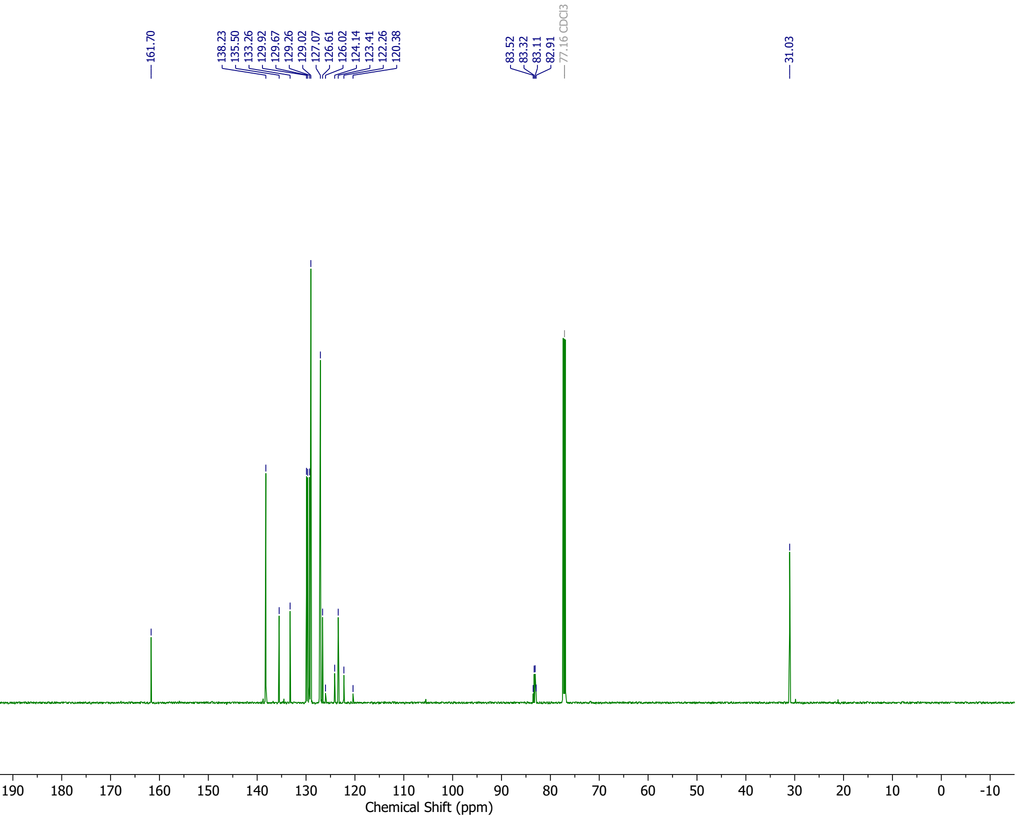
161.70
138.23
135.50
133.26
129.92
129.67
129.26
129.02
127.07
126.61
126.02
124.14
123.41
122.26
120.38
83.52
83.32
83.11
82.91
77.16 CDCl3
31.03
220 210 200 190 180 170 160 150 140 130 120 110 100 90 80 70 60 50 40 30 20 10 0 -10
Chemical Shift (ppm)

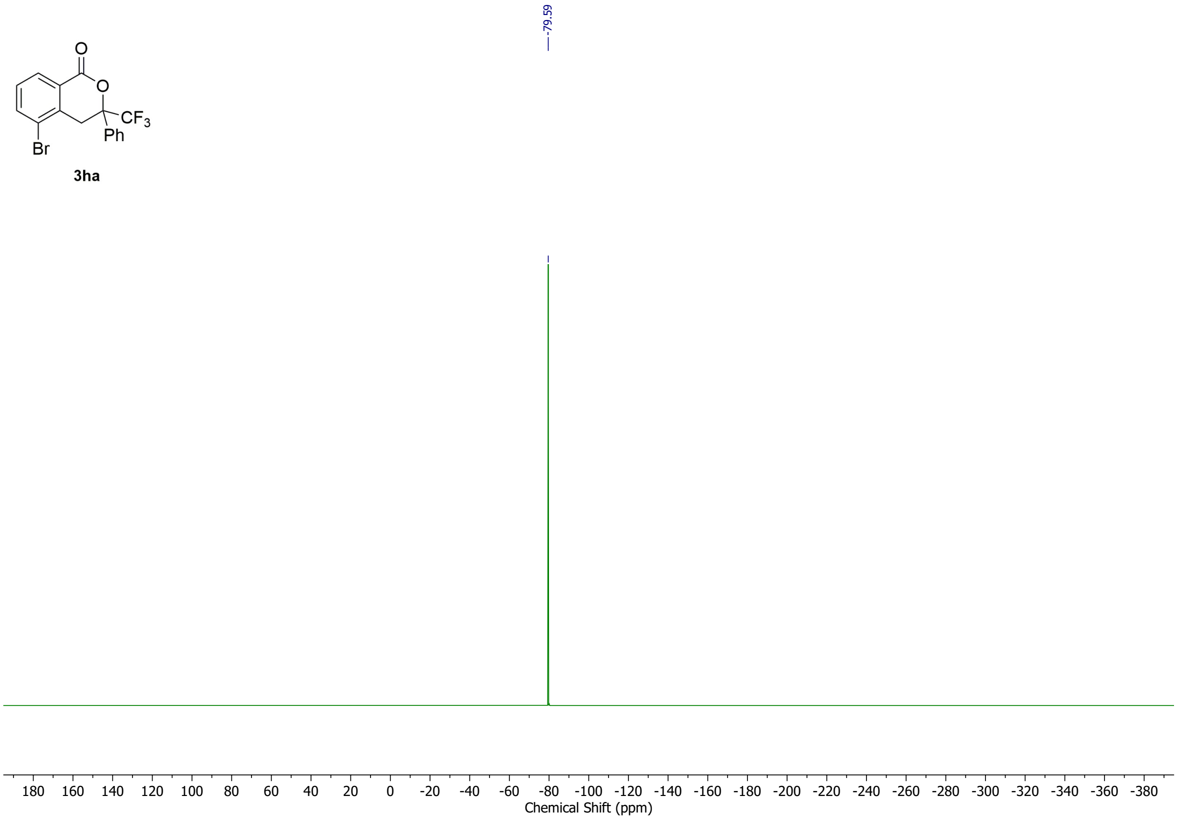
O
O
CF3
Ph
Br
3ha
-79.59
180 160 140 120 100 80 60 40 20 0 -20 -40 -60 -80 -100 -120 -140 -160 -180 -200 -220 -240 -260 -280 -300 -320 -340 -360 -380
Chemical Shift (ppm)

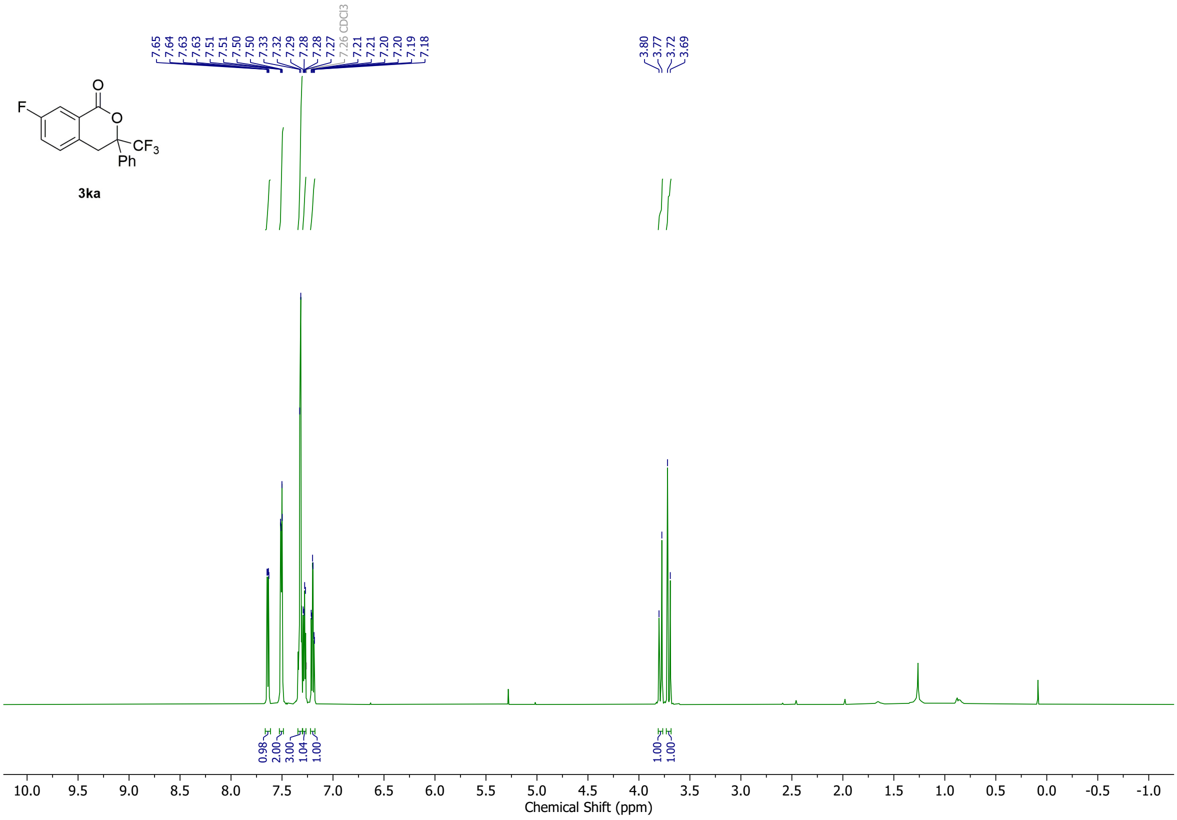
F
O
O
CF3
Ph
3ka
7.65
7.64
7.63
7.63
7.51
7.51
7.50
7.50
7.33
7.32
7.29
7.28
7.28
7.27
7.26 CDCl3
7.21
7.21
7.20
7.20
7.19
7.18
3.80
3.77
3.72
3.69
0.98
2.00
3.00
1.04
1.00
1.00
1.00
10.0 9.5 9.0 8.5 8.0 7.5 7.0 6.5 6.0 5.5 5.0 4.5 4.0 3.5 3.0 2.5 2.0 1.5 1.0 0.5 0.0 -0.5 -1.0
Chemical Shift (ppm)

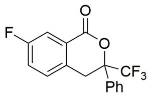
F
O
O
CF3
Ph

3ka

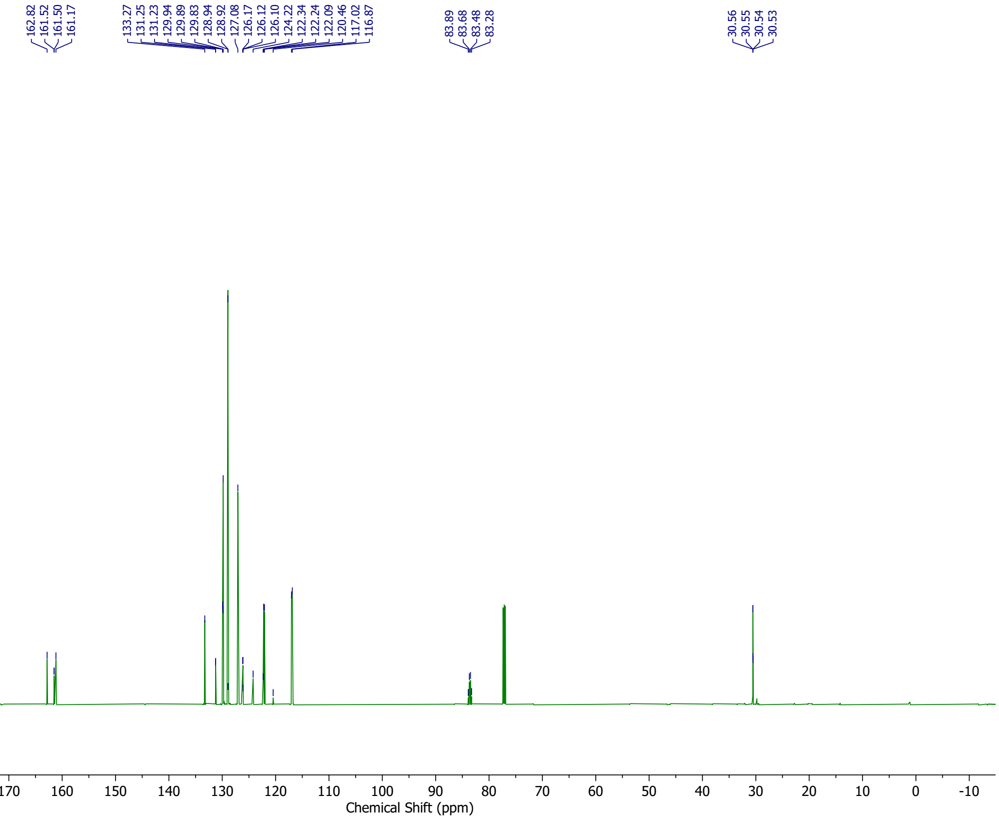
162.82
161.52
161.50
161.17
133.27
131.25
131.23
129.94
129.89
129.83
128.94
128.92
127.08
126.17
126.12
126.10
124.22
122.34
122.24
122.09
120.46
117.02
116.87
83.89
83.68
83.48
83.28
30.56
30.55
30.54
30.53
200 190 180 170 160 150 140 130 120 110 100 90 80 70 60 50 40 30 20 10 0 -10
Chemical Shift (ppm)

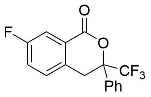
F
O
O
CF3
Ph

3ka

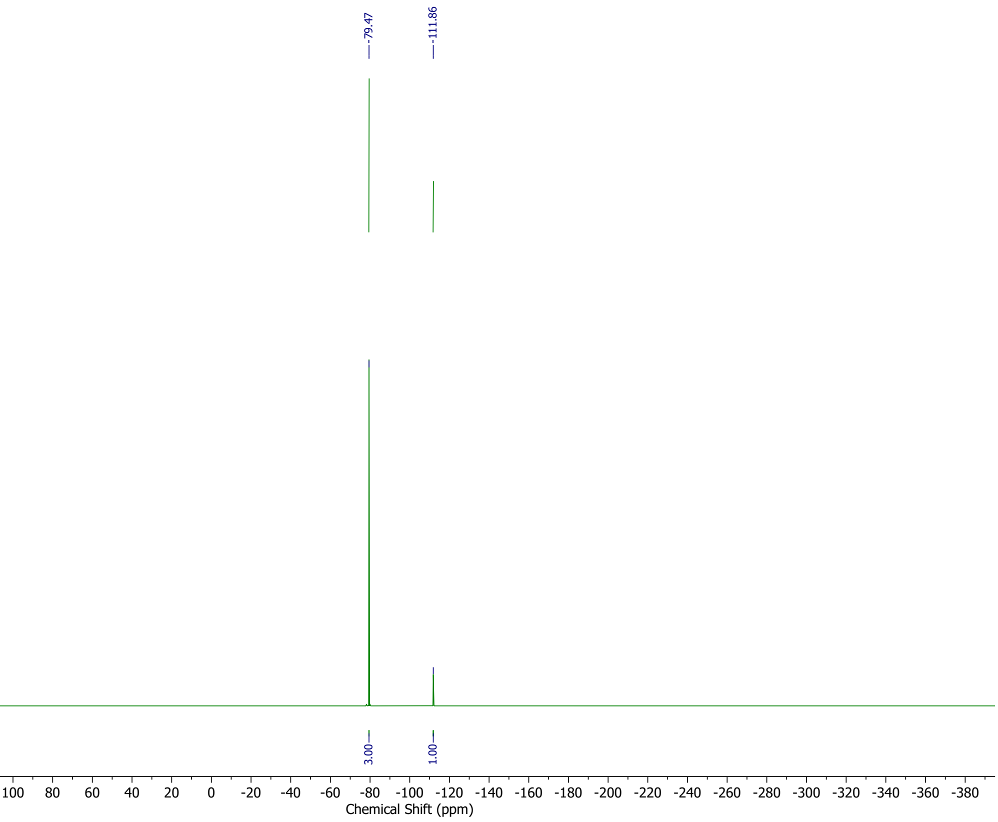
-79.47
-111.86
3.00
1.00
180 160 140 120 100 80 60 40 20 0 -20 -40 -60 -80 -100 -120 -140 -160 -180 -200 -220 -240 -260 -280 -300 -320 -340 -360 -380
Chemical Shift (ppm)

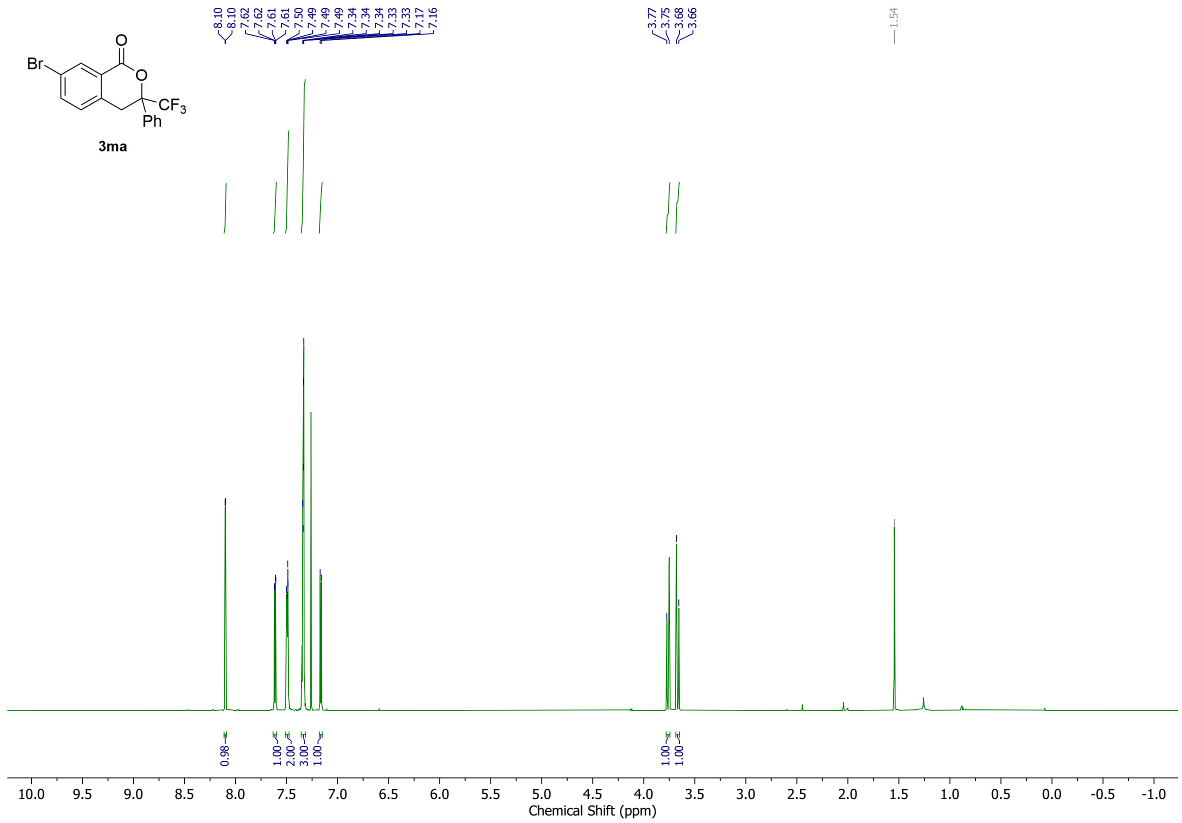
8.10
8.10
7.62
7.62
7.61
7.61
7.50
7.49
7.49
7.49
7.34
7.34
7.34
7.34
7.33
7.33
7.17
7.16
3.77
3.75
3.68
3.66
1.54
Br
O
O
CF3
Ph
3ma
0.98
1.00
2.00
3.00
1.00
1.00
1.00
10.0 9.5 9.0 8.5 8.0 7.5 7.0 6.5 6.0 5.5 5.0 4.5 4.0 3.5 3.0 2.5 2.0 1.5 1.0 0.5 0.0 -0.5 -1.0
Chemical Shift (ppm)

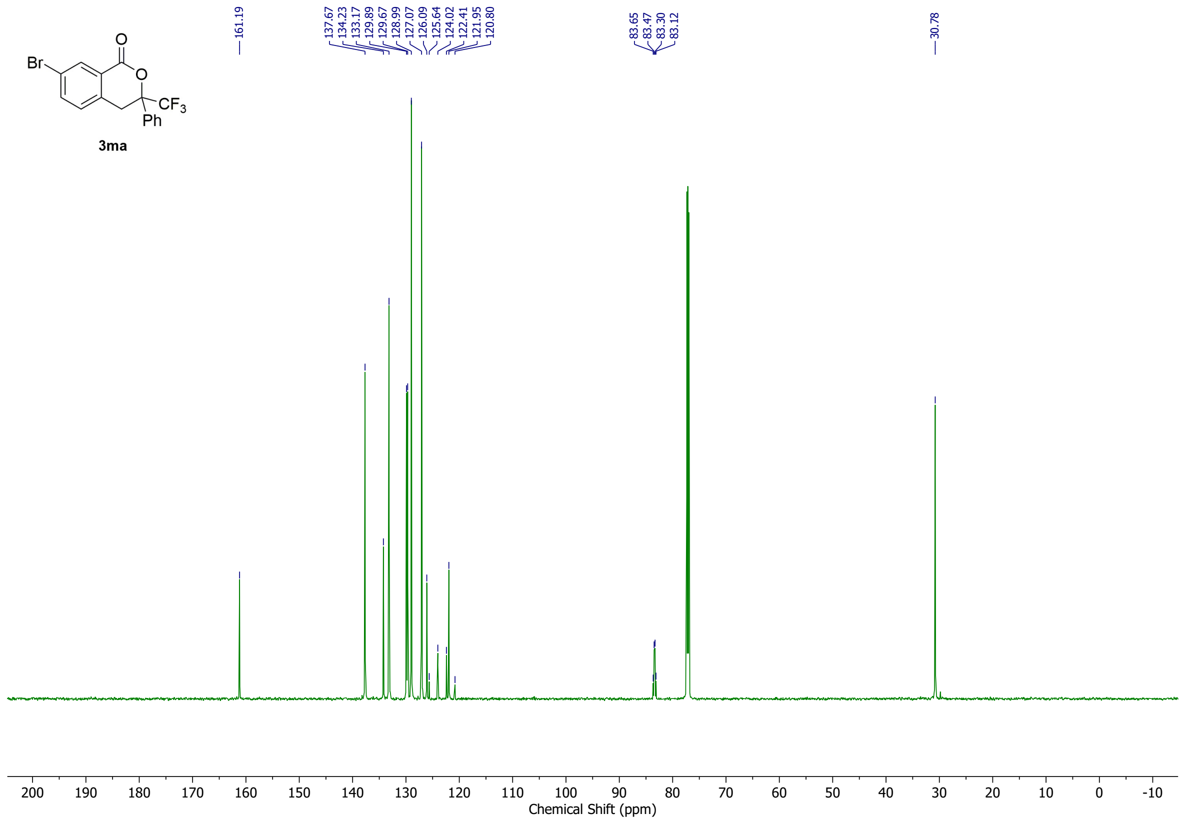
161.19
137.67
134.23
133.17
129.89
129.67
128.99
127.07
126.09
125.64
124.02
122.41
121.95
120.80
83.65
83.47
83.30
83.12
30.78
Br
O
O
CF3
Ph
3ma
200 190 180 170 160 150 140 130 120 110 100 90 80 70 60 50 40 30 20 10 0 -10
Chemical Shift (ppm)

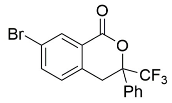
Br
O
O
CF3
Ph

3ma

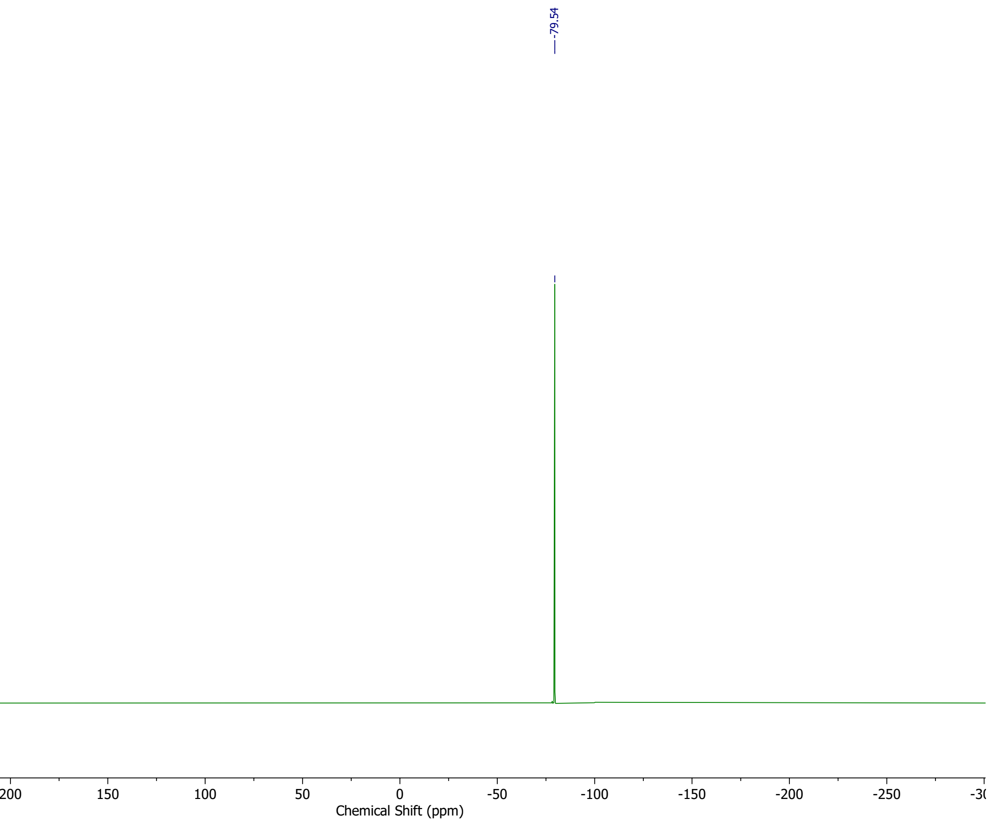
-79.54
Chemical Shift (ppm)

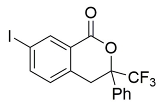
I
O
O
CF3
Ph

3na

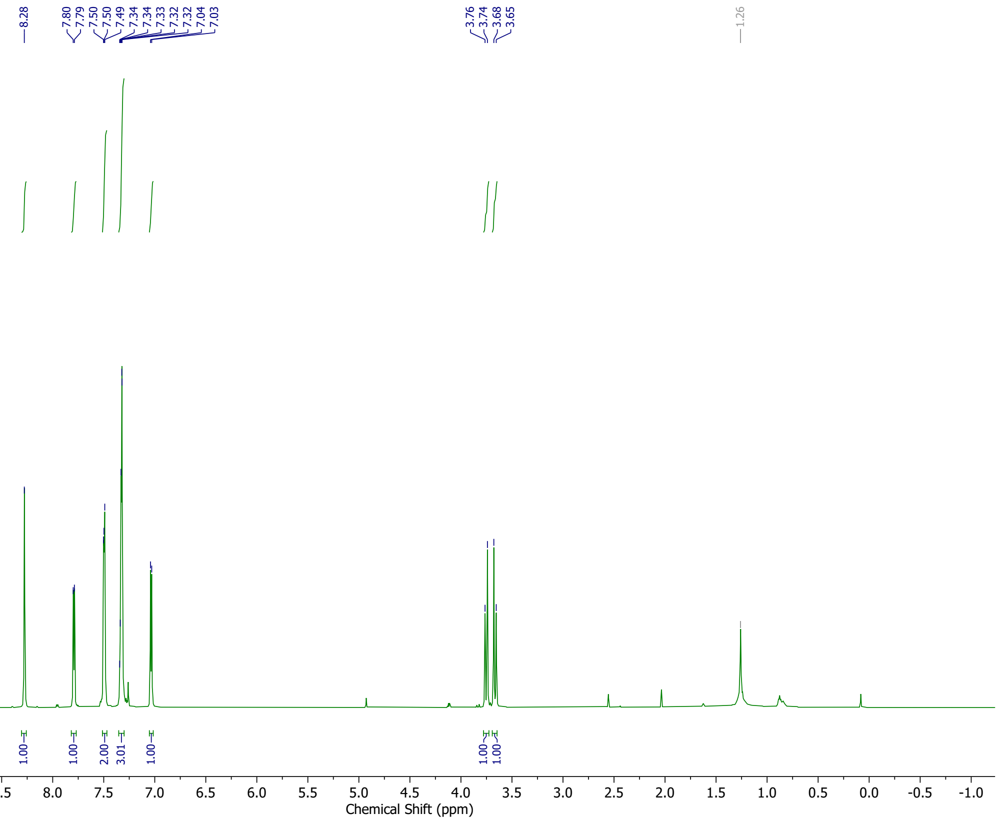
8.28
7.80
7.79
7.50
7.50
7.49
7.34
7.34
7.33
7.32
7.32
7.04
7.03
3.76
3.74
3.68
3.65
1.26
1.00
1.00
2.00
3.01
1.00
1.00
1.00
Chemical Shift (ppm)

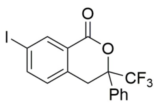
I
O
O
CF3
Ph

3na

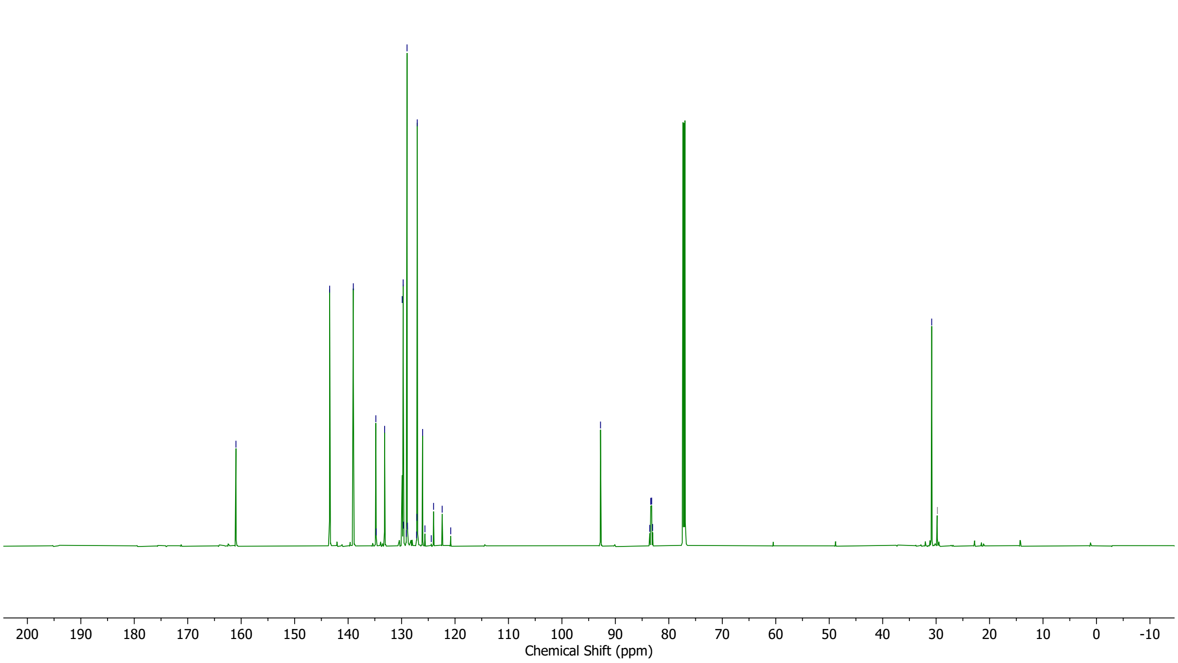
160.98
143.45
139.02
134.81
134.74
133.17
129.88
129.70
129.64
129.09
128.98
128.90
127.20
127.13
127.06
126.06
125.63
124.44
124.02
122.41
120.80
92.78
83.56
83.39
83.21
83.04
30.83
29.80
Chemical Shift (ppm)

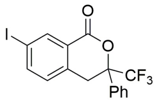
I
O
O
CF3
Ph

3na

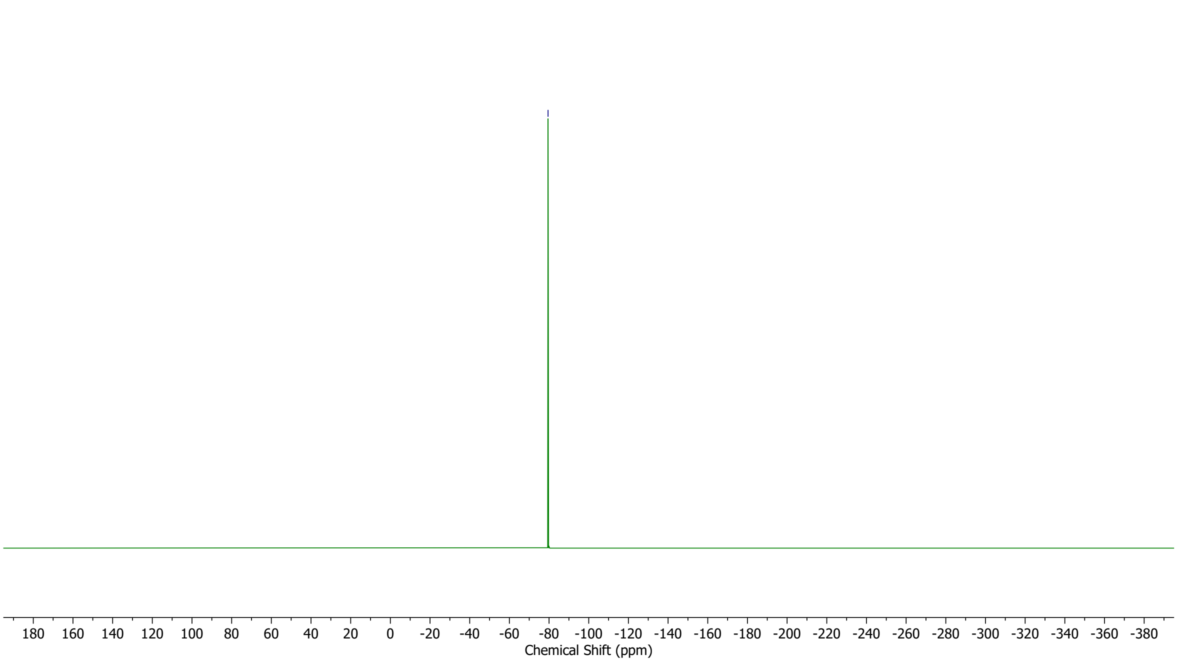
-79.49
Chemical Shift (ppm)

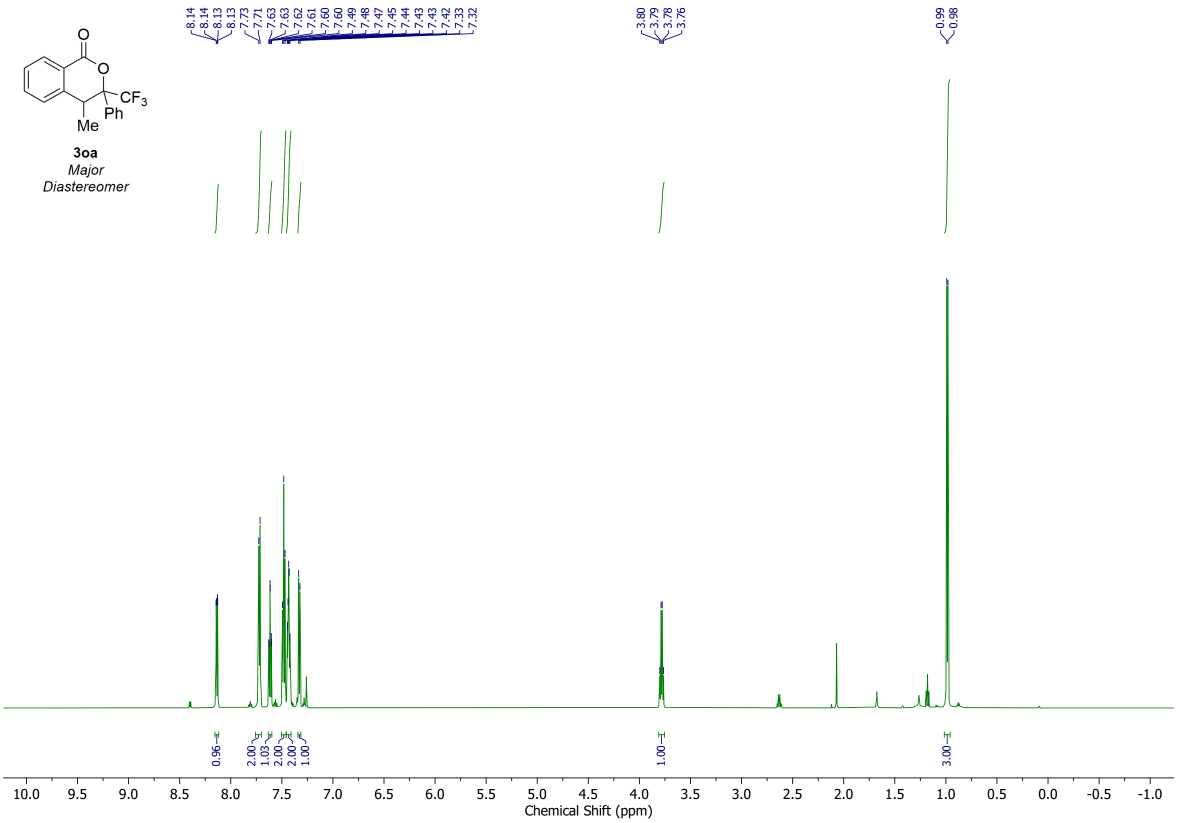
O
O
CF3
Ph
Me
3oa
Major
Diastereomer
8.14
8.14
8.13
8.13
7.73
7.71
7.63
7.63
7.62
7.61
7.60
7.49
7.48
7.47
7.45
7.44
7.43
7.43
7.42
7.33
7.32
3.80
3.79
3.78
3.76
0.99
0.98
0.96
2.00
1.03
2.00
2.00
1.00
1.00
3.00
10.0 9.5 9.0 8.5 8.0 7.5 7.0 6.5 6.0 5.5 5.0 4.5 4.0 3.5 3.0 2.5 2.0 1.5 1.0 0.5 0.0 -0.5 -1.0
Chemical Shift (ppm)

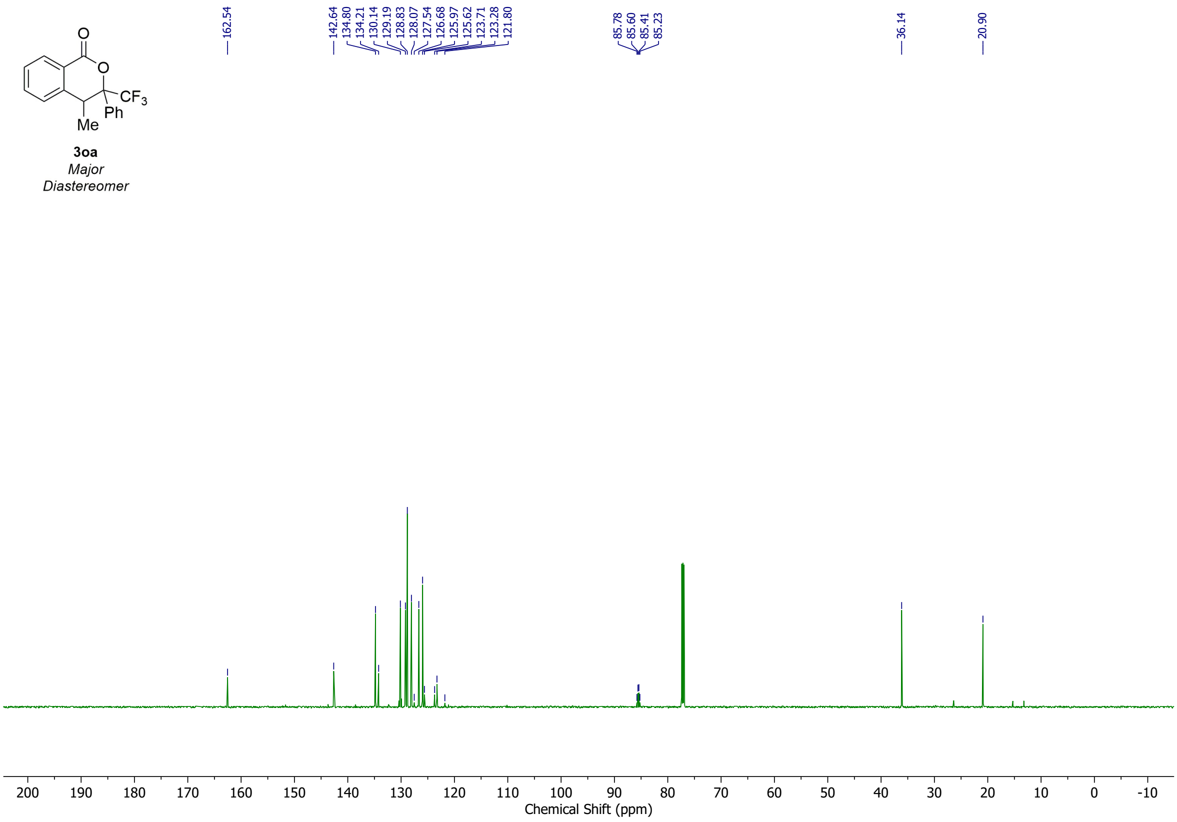
O
O
CF3
Ph
Me
3oa
Major
Diastereomer
162.54
142.64
134.80
134.21
130.14
129.19
128.83
128.07
127.54
126.68
125.97
125.62
123.71
123.28
121.80
85.78
85.60
85.41
85.23
36.14
20.90
200 190 180 170 160 150 140 130 120 110 100 90 80 70 60 50 40 30 20 10 0 -10
Chemical Shift (ppm)

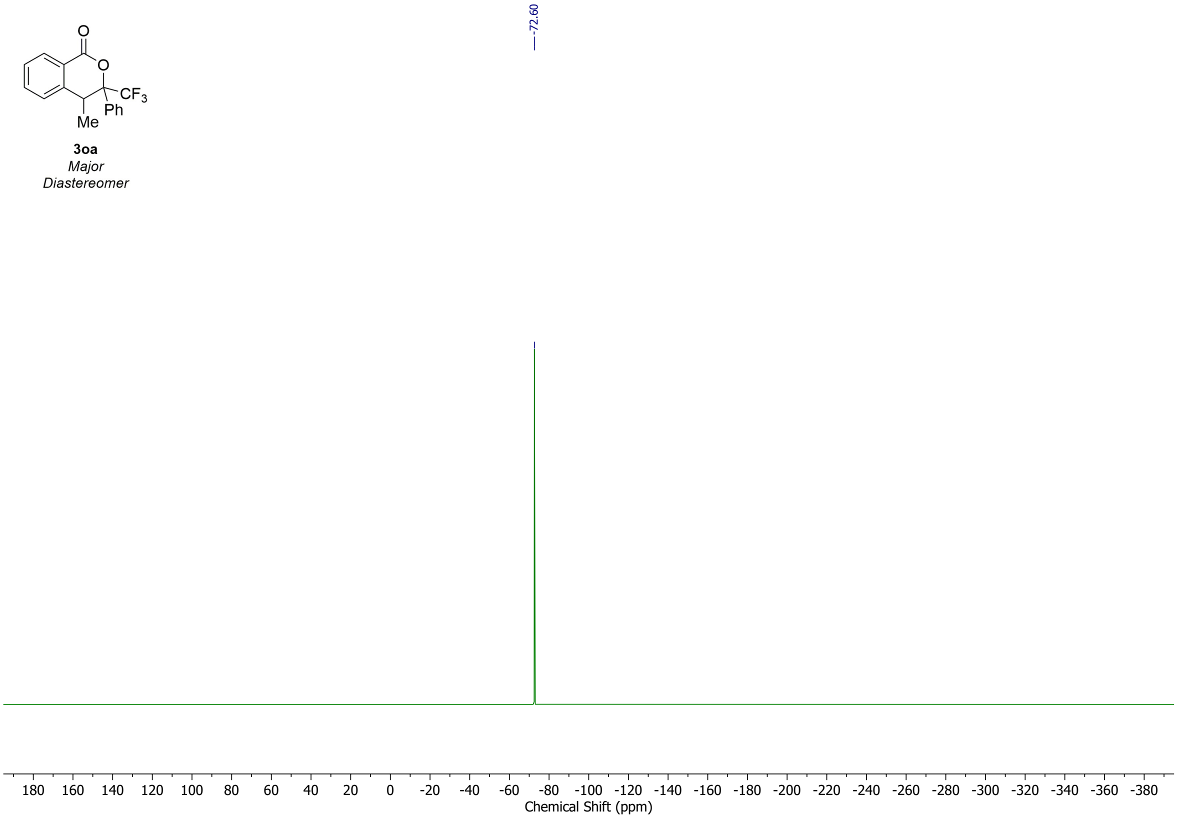
3oa
Major
Diastereomer
-72.60
180 160 140 120 100 80 60 40 20 0 -20 -40 -60 -80 -100 -120 -140 -160 -180 -200 -220 -240 -260 -280 -300 -320 -340 -360 -380
Chemical Shift (ppm)

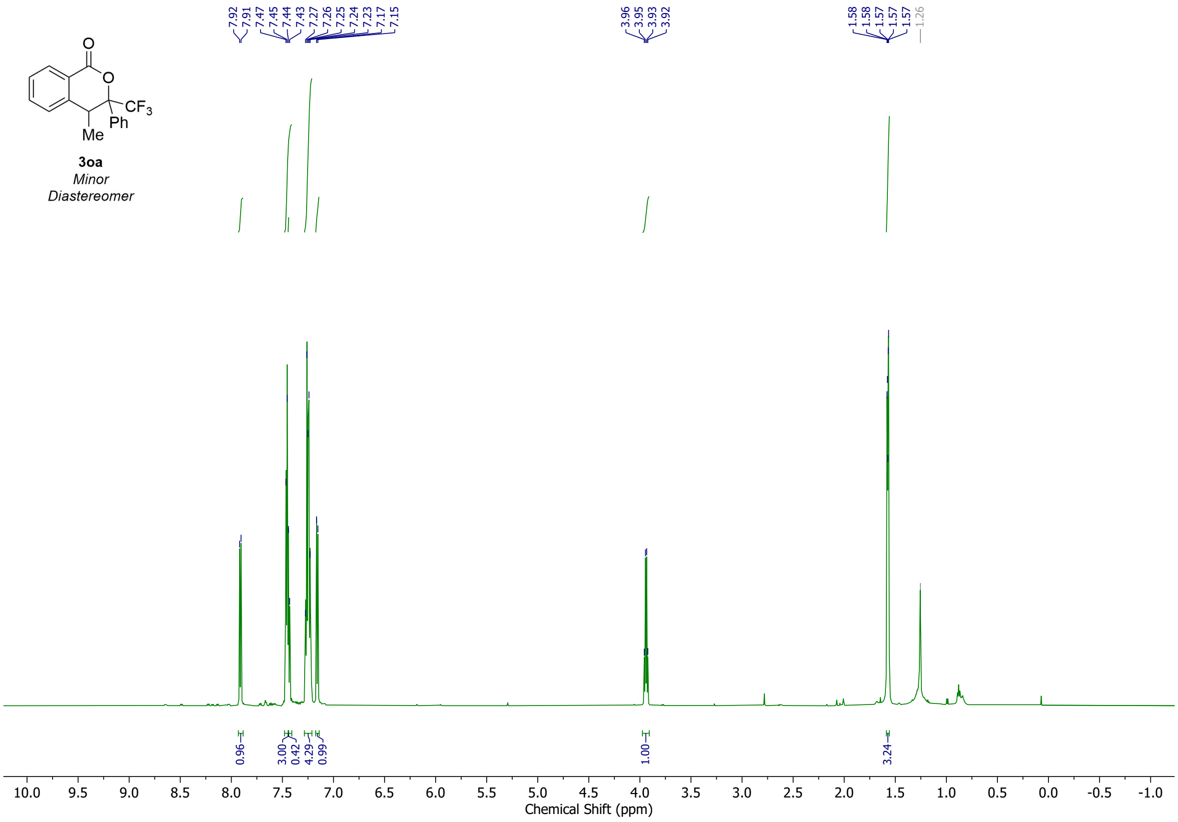
3oa
Minor
Diastereomer
7.92
7.91
7.47
7.45
7.44
7.43
7.27
7.26
7.25
7.24
7.23
7.17
7.15
3.96
3.95
3.93
3.92
1.58
1.58
1.57
1.57
1.57
1.26
0.96
3.00
0.42
4.29
0.99
1.00
3.24
10.0 9.5 9.0 8.5 8.0 7.5 7.0 6.5 6.0 5.5 5.0 4.5 4.0 3.5 3.0 2.5 2.0 1.5 1.0 0.5 0.0 -0.5 -1.0
Chemical Shift (ppm)

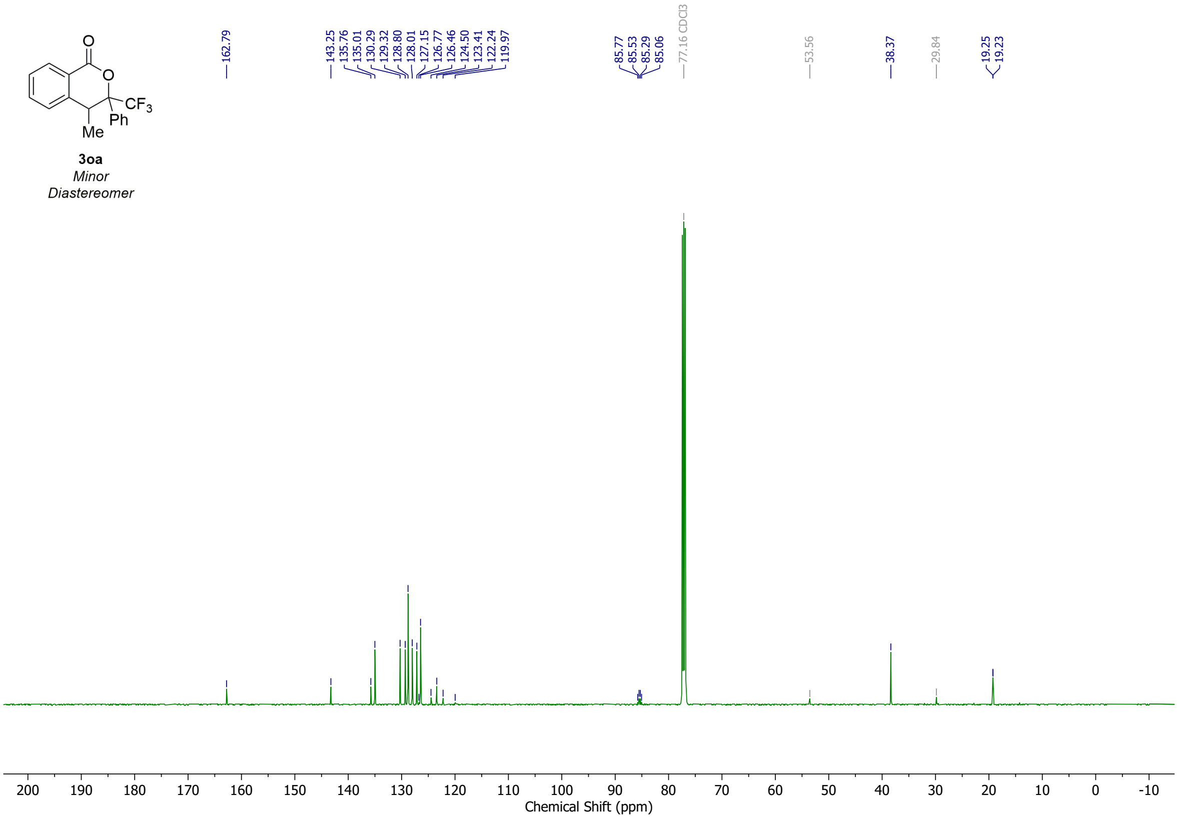
3oa
Minor
Diastereomer
162.79
143.25
135.76
135.01
130.29
129.32
128.80
128.01
127.15
126.77
126.46
124.50
123.41
122.24
119.97
85.77
85.53
85.29
85.06
77.16 CDCl3
53.56
38.37
29.84
19.25
19.23
Chemical Shift (ppm)

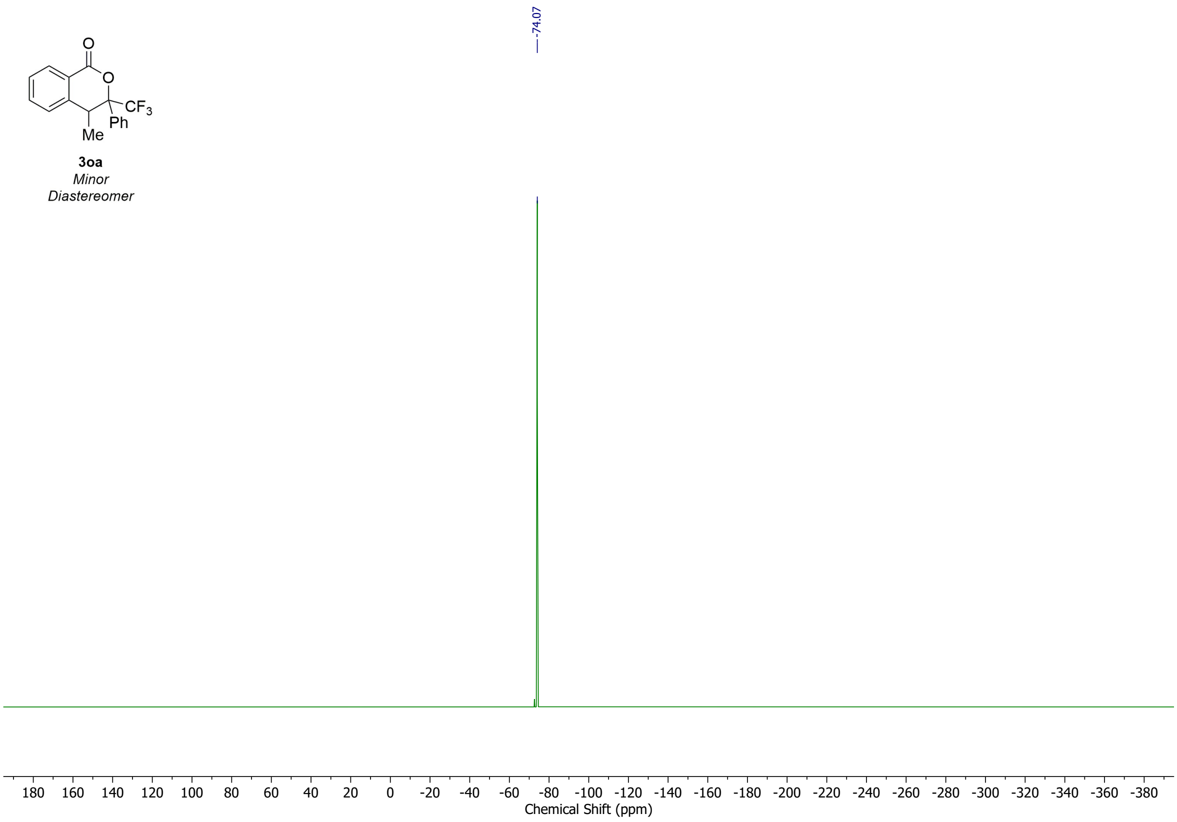
3oa
Minor
Diastereomer
-74.07
Chemical Shift (ppm)

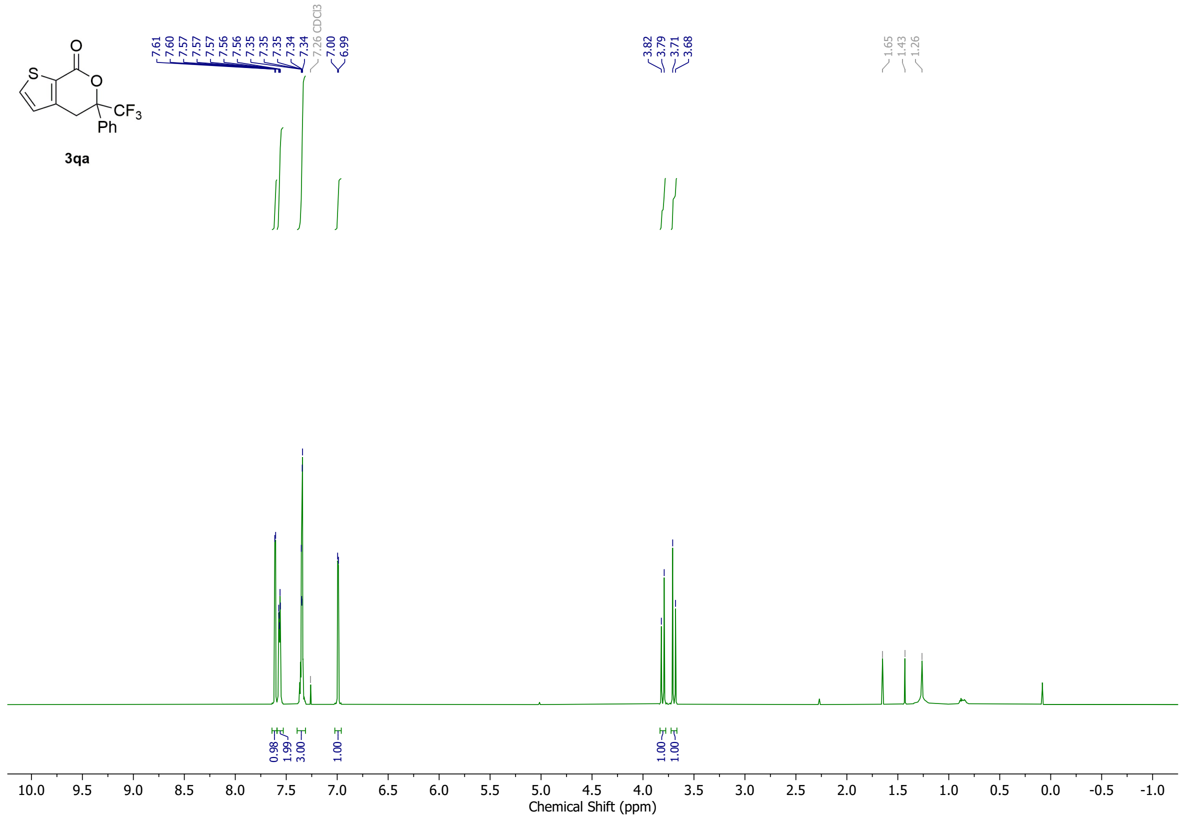
S
O
O
CF3
Ph
3qa
7.61
7.60
7.57
7.57
7.57
7.56
7.56
7.35
7.35
7.35
7.34
7.34
7.26 CDCl3
7.00
6.99
3.82
3.79
3.71
3.68
1.65
1.43
1.26
0.98
1.99
3.00
1.00
1.00
1.00
10.0 9.5 9.0 8.5 8.0 7.5 7.0 6.5 6.0 5.5 5.0 4.5 4.0 3.5 3.0 2.5 2.0 1.5 1.0 0.5 0.0 -0.5 -1.0
Chemical Shift (ppm)

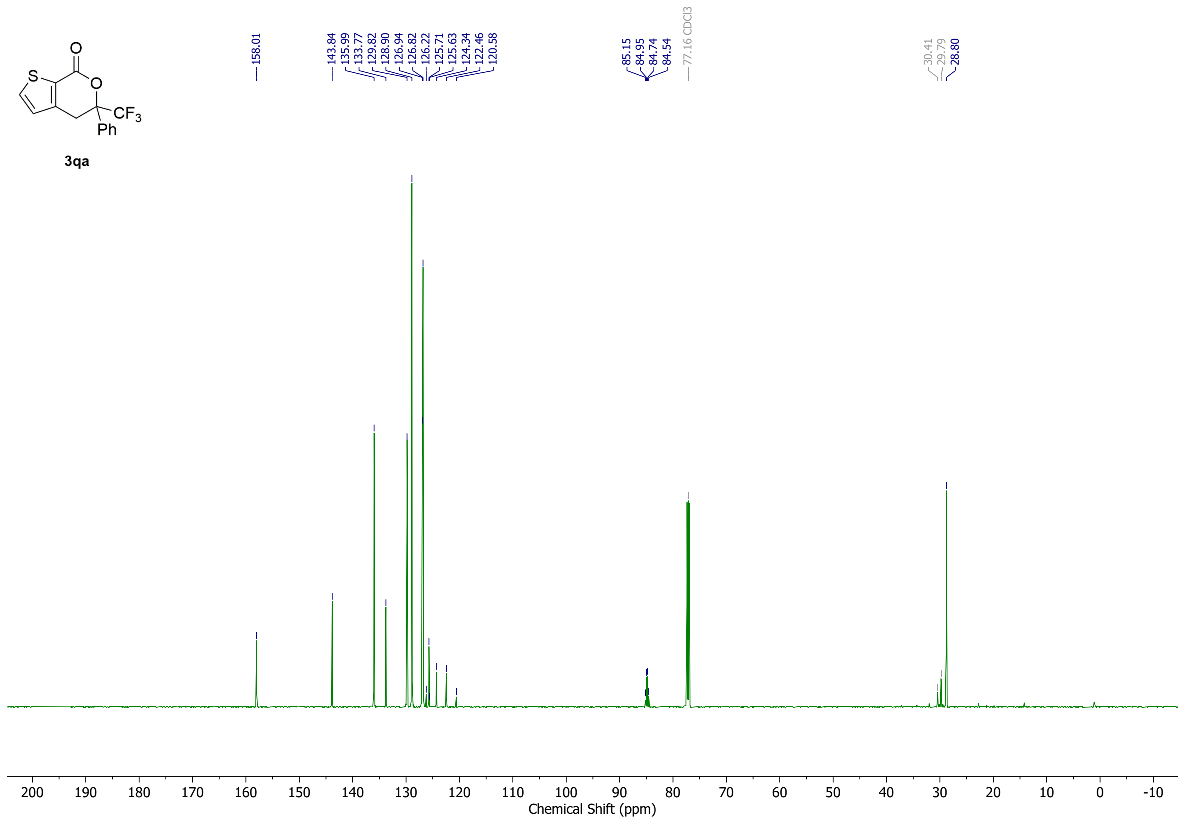
S
O
O
CF3
Ph
3qa
158.01
143.84
135.99
133.77
129.82
128.90
126.94
126.82
126.22
125.71
125.63
124.34
122.46
120.58
85.15
84.95
84.74
84.54
77.16 CDCl3
30.41
29.79
28.80
200 190 180 170 160 150 140 130 120 110 100 90 80 70 60 50 40 30 20 10 0 -10
Chemical Shift (ppm)

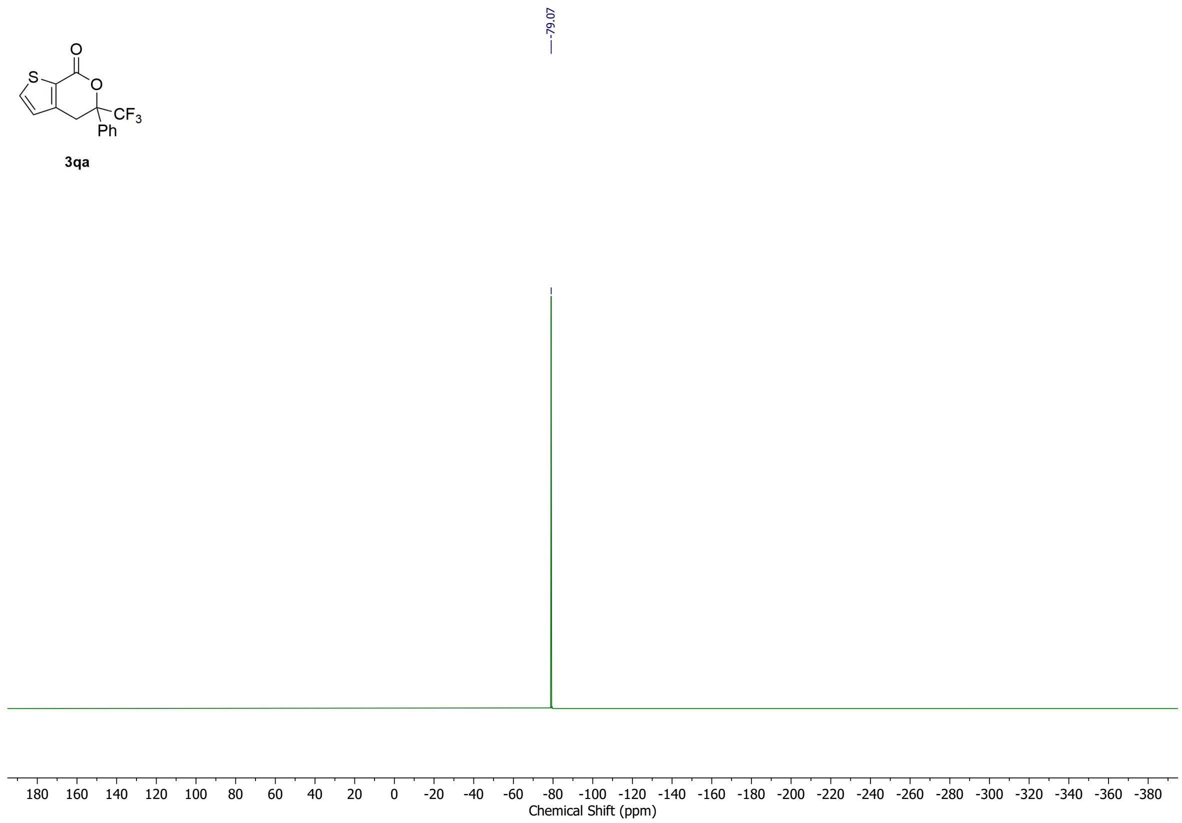

3qa
-79.07
Chemical Shift (ppm)

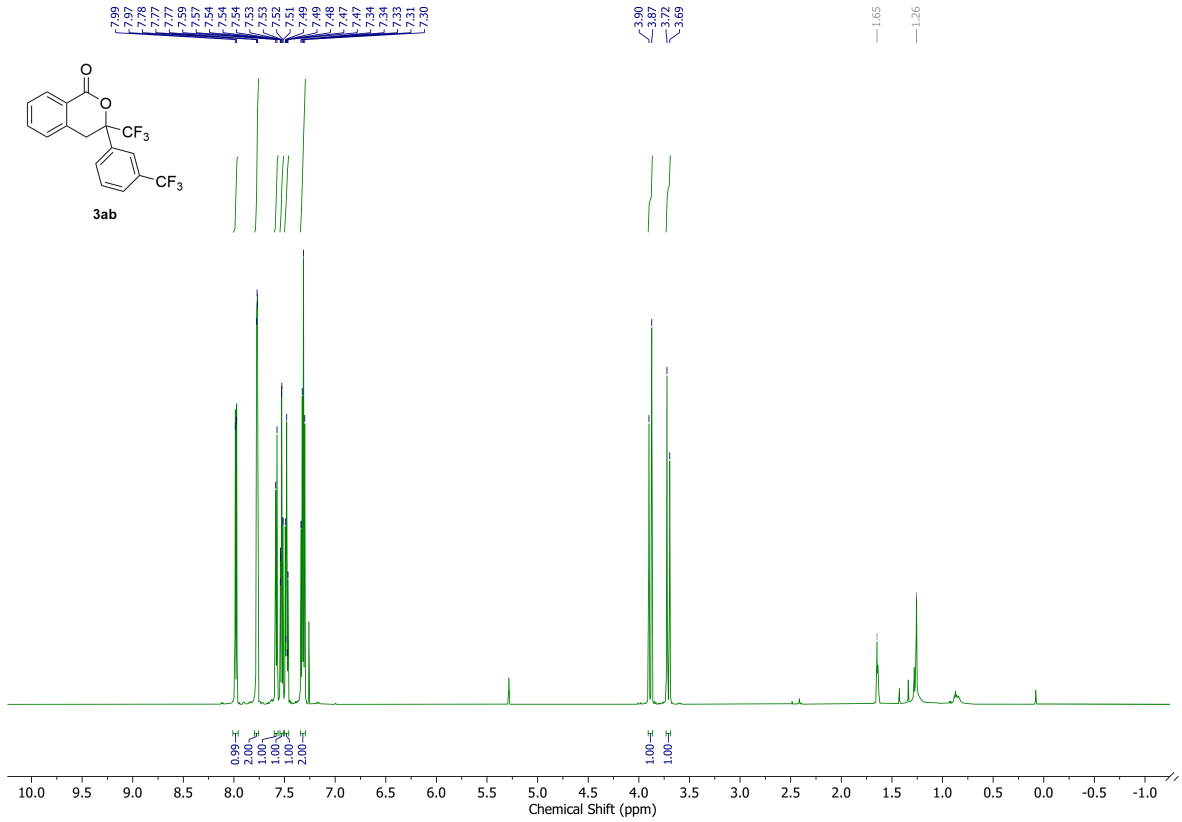

3ab
Chemical Shift (ppm)

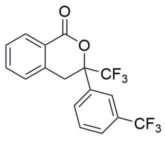
O
O
CF3
CF3

3ab

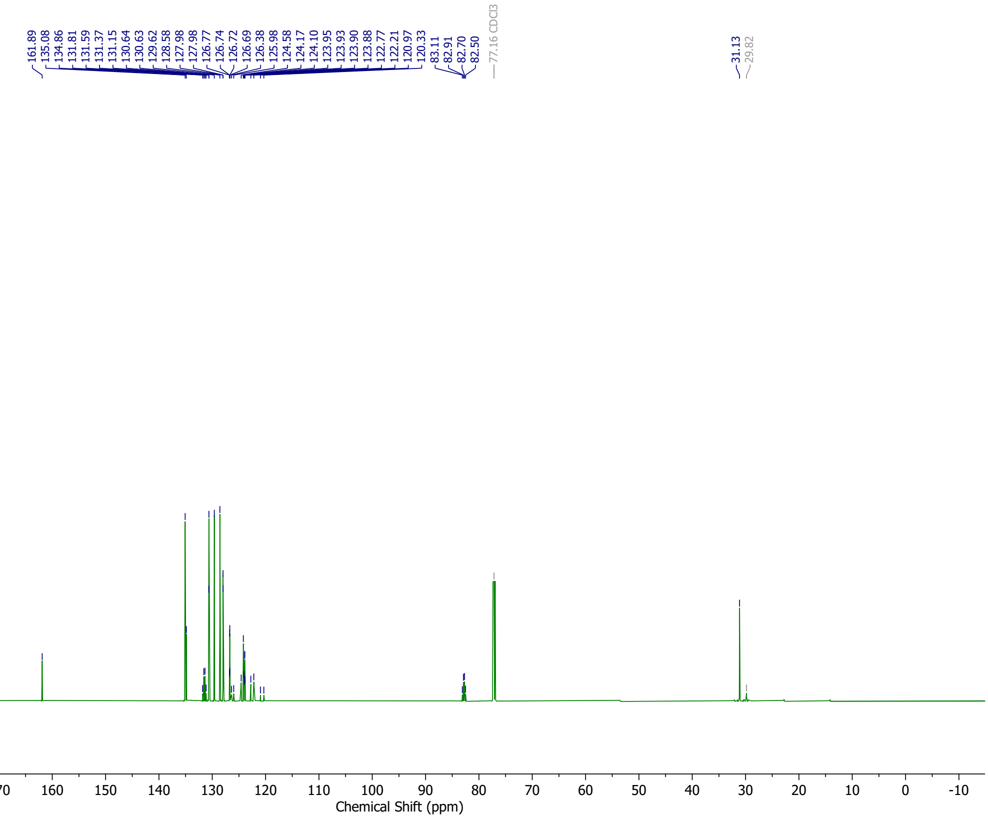
161.89
135.08
134.86
131.81
131.59
131.37
131.15
130.64
130.63
129.62
128.58
127.98
127.98
126.77
126.74
126.72
126.69
126.38
125.98
124.58
124.17
124.10
123.95
123.93
123.90
123.88
122.77
122.21
120.97
120.33
83.11
82.91
82.70
82.50
77.16 CDCl3
31.13
29.82
Chemical Shift (ppm)

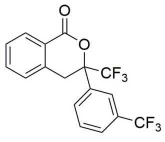
O
O
CF3
CF3

3ab

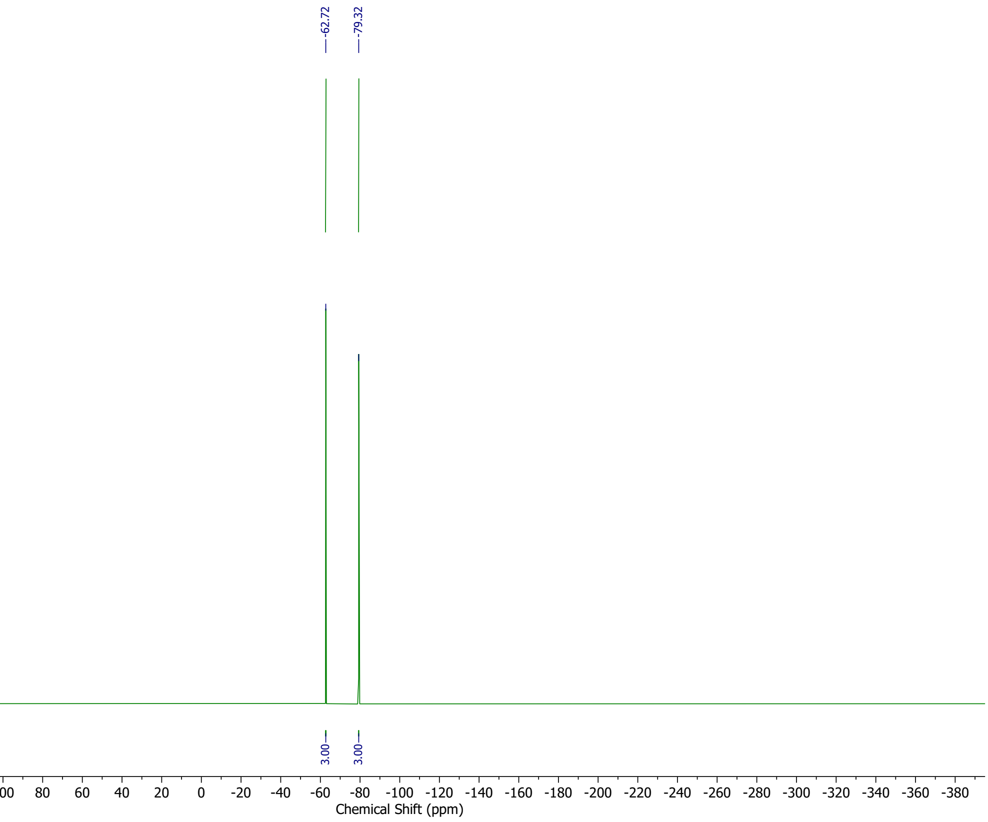
-62.72
-79.32
3.00
3.00
Chemical Shift (ppm)

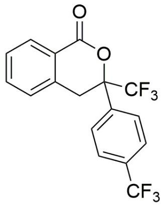
O
O
CF3
CF3

3ac

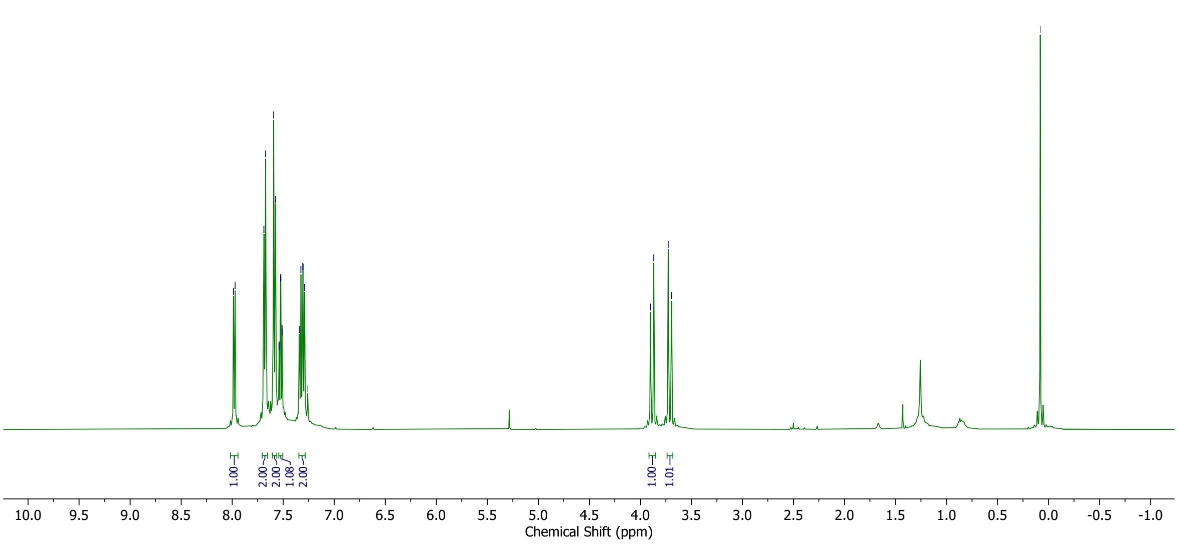
7.99
7.97
7.69
7.67
7.59
7.58
7.54
7.54
7.53
7.52
7.51
7.51
7.34
7.33
7.31
7.29
7.26 CDCl3
3.90
3.87
3.73
3.69
0.08
1.00
2.00
2.00
1.08
2.00
1.00
1.01
Chemical Shift (ppm)

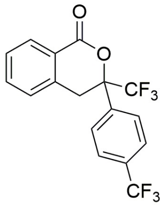
O
O
CF3
CF3

3ac

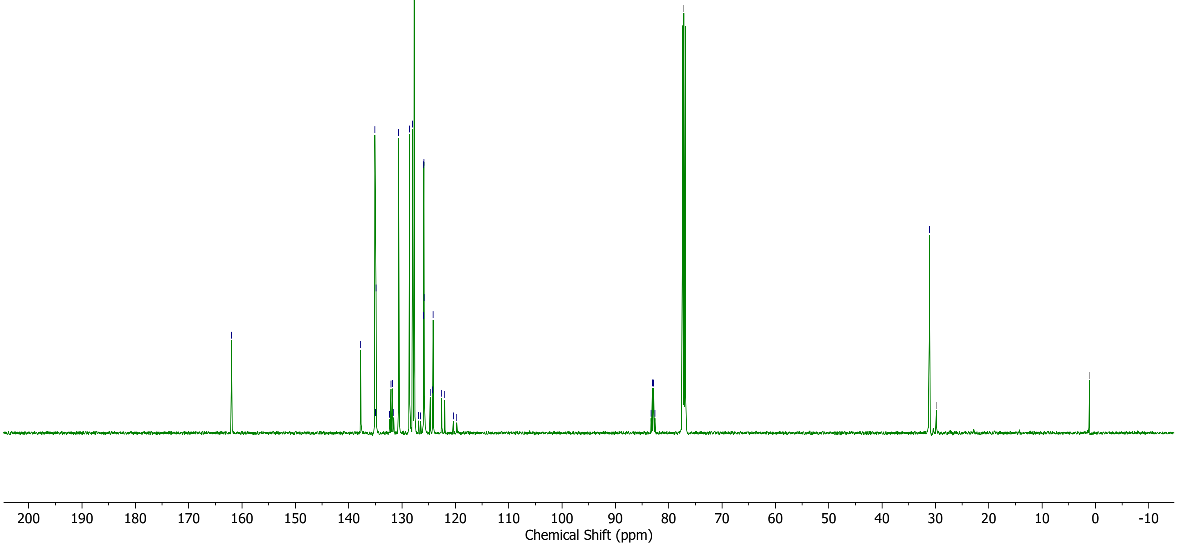
161.98
137.75
135.09
135.00
134.88
132.33
132.07
131.81
131.55
130.65
128.59
128.03
127.74
126.89
126.51
125.96
125.93
125.90
125.87
124.73
124.25
124.18
122.56
121.99
120.39
119.74
83.32
83.07
82.83
82.58
77.16 CDCl3
31.14
29.82
1.13
Chemical Shift (ppm)

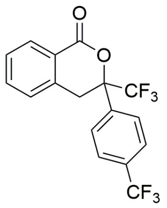
O
O
CF_3
CF_3

3ac

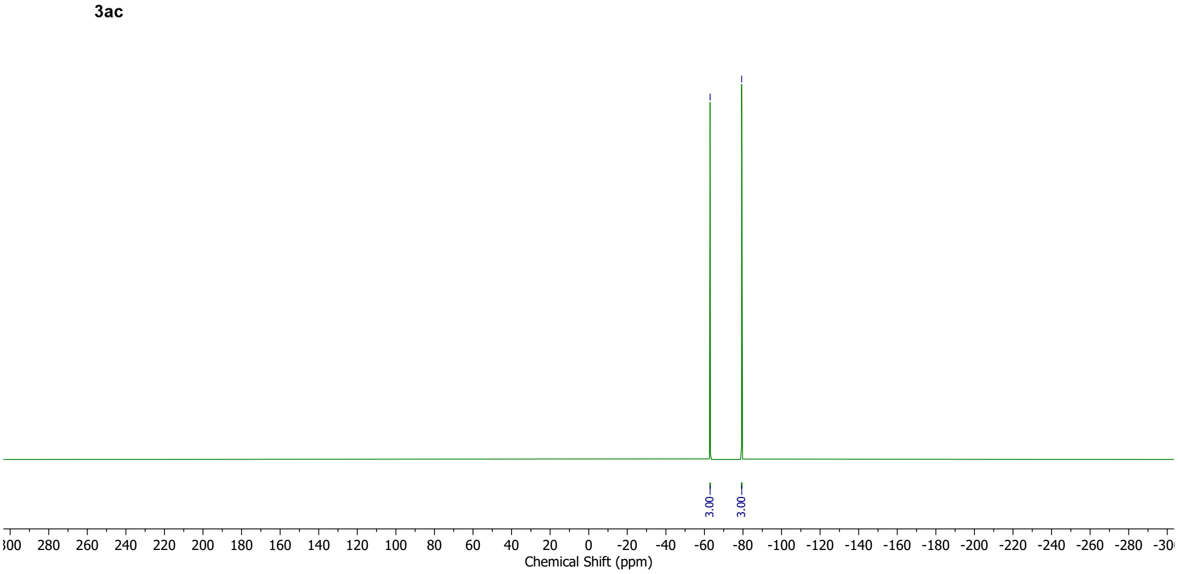
-62.96
-79.32
3.00
3.00
300 280 260 240 220 200 180 160 140 120 100 80 60 40 20 0 -20 -40 -60 -80 -100 -120 -140 -160 -180 -200 -220 -240 -260 -280 -300
Chemical Shift (ppm)

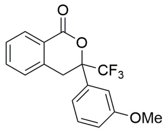
O
O
CF_3
OMe

3ad

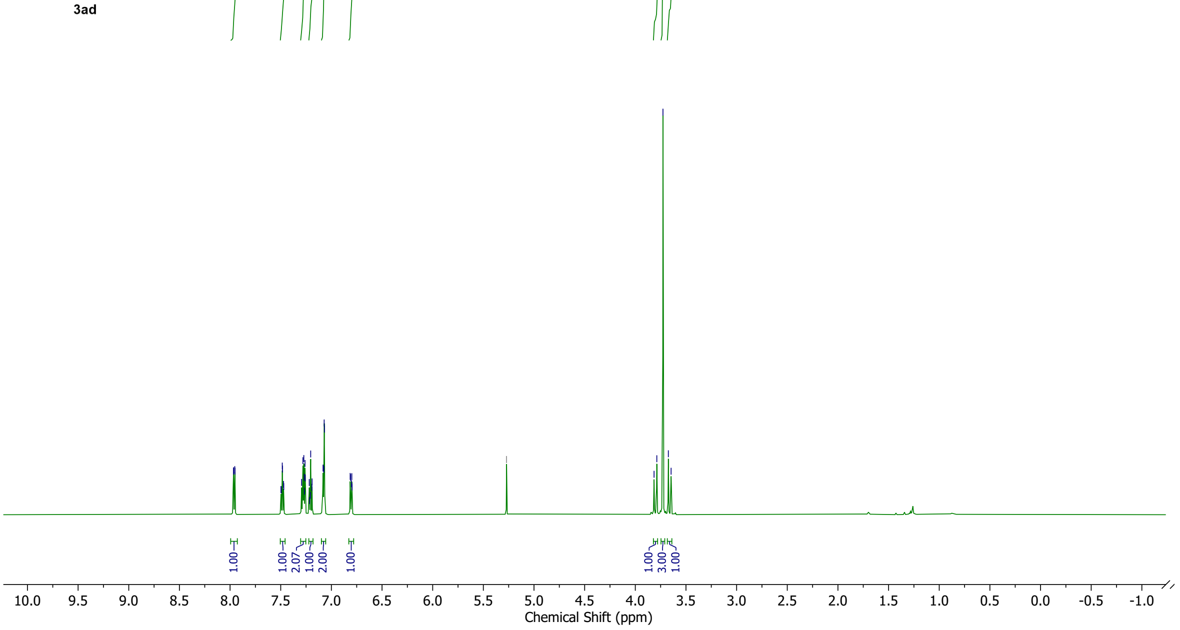
7.97
7.96
7.95
7.95
7.50
7.50
7.48
7.48
7.47
7.29
7.28
7.27
7.27
7.27
7.26
7.22
7.21
7.21
7.20
7.19
7.19
7.08
7.07
7.07
7.07
6.81
6.81
6.80
6.80
6.80
5.27
3.81
3.79
3.73
3.67
3.65
1.00
1.00
2.07
1.00
2.00
1.00
1.00
3.00
1.00
10.0 9.5 9.0 8.5 8.0 7.5 7.0 6.5 6.0 5.5 5.0 4.5 4.0 3.5 3.0 2.5 2.0 1.5 1.0 0.5 0.0 -0.5 -1.0
Chemical Shift (ppm)

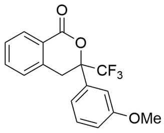
O
O
CF3
OMe

3ad

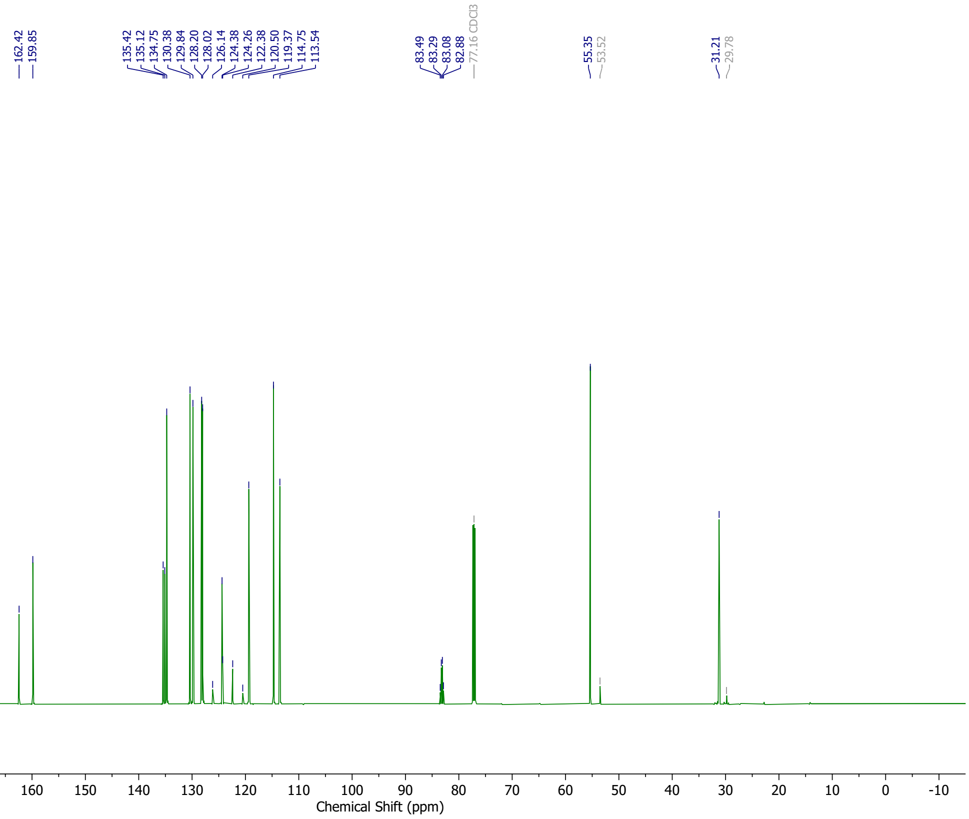
162.42
159.85
135.42
135.12
134.75
130.38
129.84
128.20
128.02
126.14
124.38
124.26
122.38
120.50
119.37
114.75
113.54
83.49
83.29
83.08
82.88
77.16 CDCl3
55.35
53.52
31.21
29.78
200 190 180 170 160 150 140 130 120 110 100 90 80 70 60 50 40 30 20 10 0 -10
Chemical Shift (ppm)

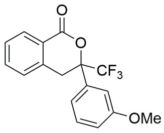
O
O
CF3
OMe

3ad

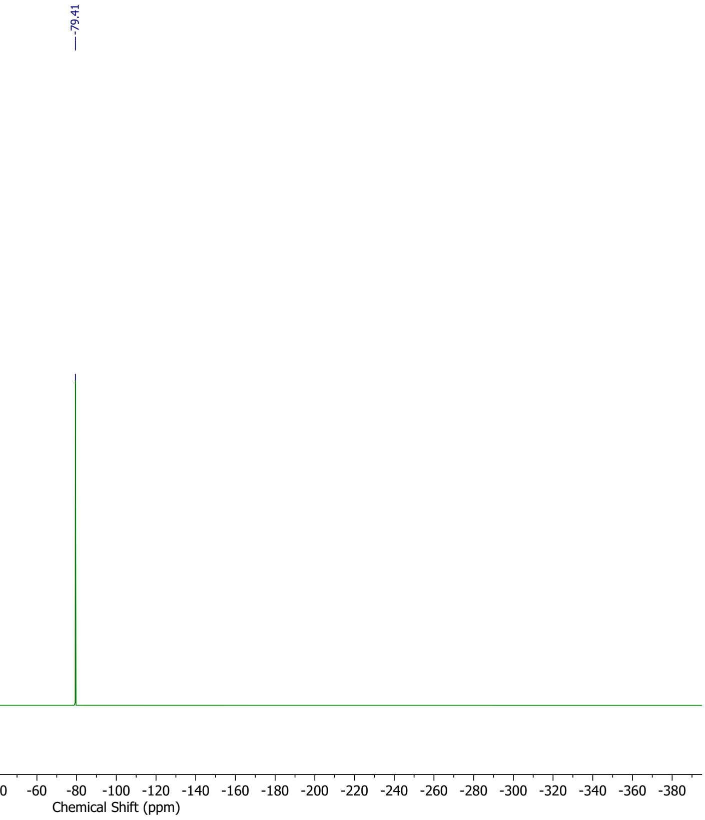
-79.41
180 160 140 120 100 80 60 40 20 0 -20 -40 -60 -80 -100 -120 -140 -160 -180 -200 -220 -240 -260 -280 -300 -320 -340 -360 -380
Chemical Shift (ppm)

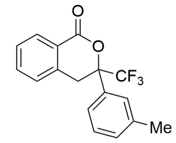
O
O
CF_3
Me

3ae

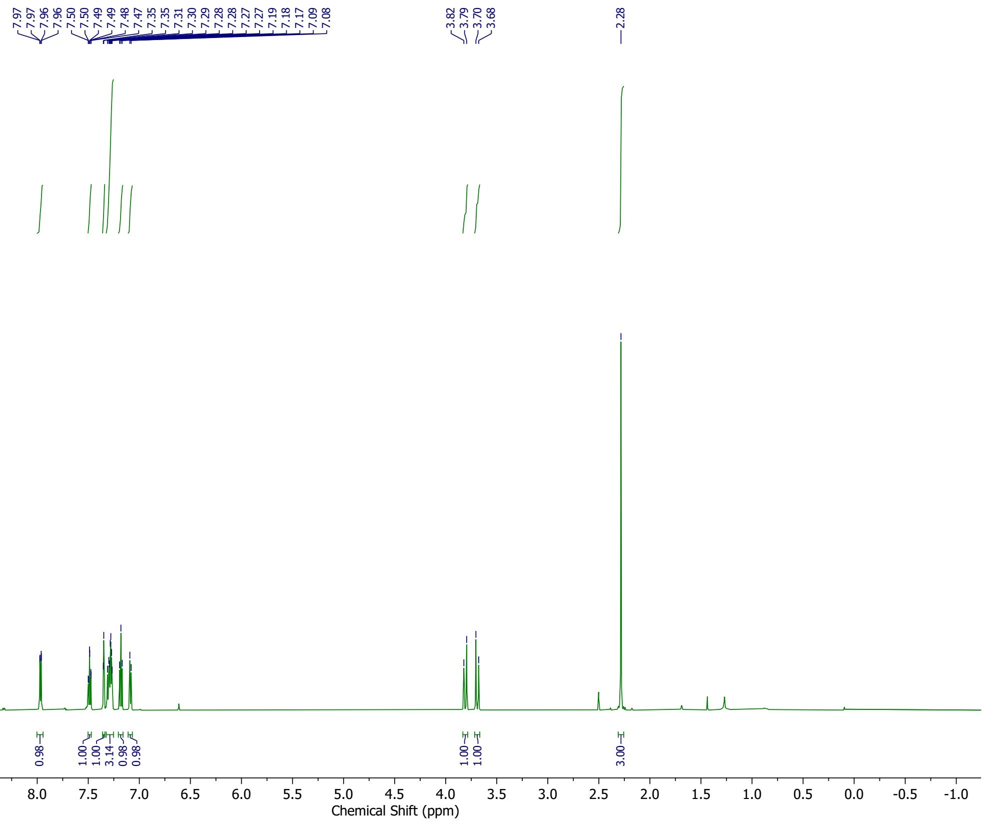
7.97 7.97 7.96 7.96 7.50 7.50 7.49 7.49 7.48 7.47 7.35 7.35 7.31 7.30 7.29 7.28 7.28 7.27 7.27 7.19 7.18 7.17 7.09 7.08
3.82 3.79 3.70 3.68
2.28
0.98
1.00 1.00 3.14 0.98 0.98
1.00 1.00
3.00
10.0 9.5 9.0 8.5 8.0 7.5 7.0 6.5 6.0 5.5 5.0 4.5 4.0 3.5 3.0 2.5 2.0 1.5 1.0 0.5 0.0 -0.5 -1.0
Chemical Shift (ppm)

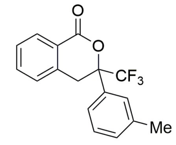
O
O
CF_3
Me

3ae

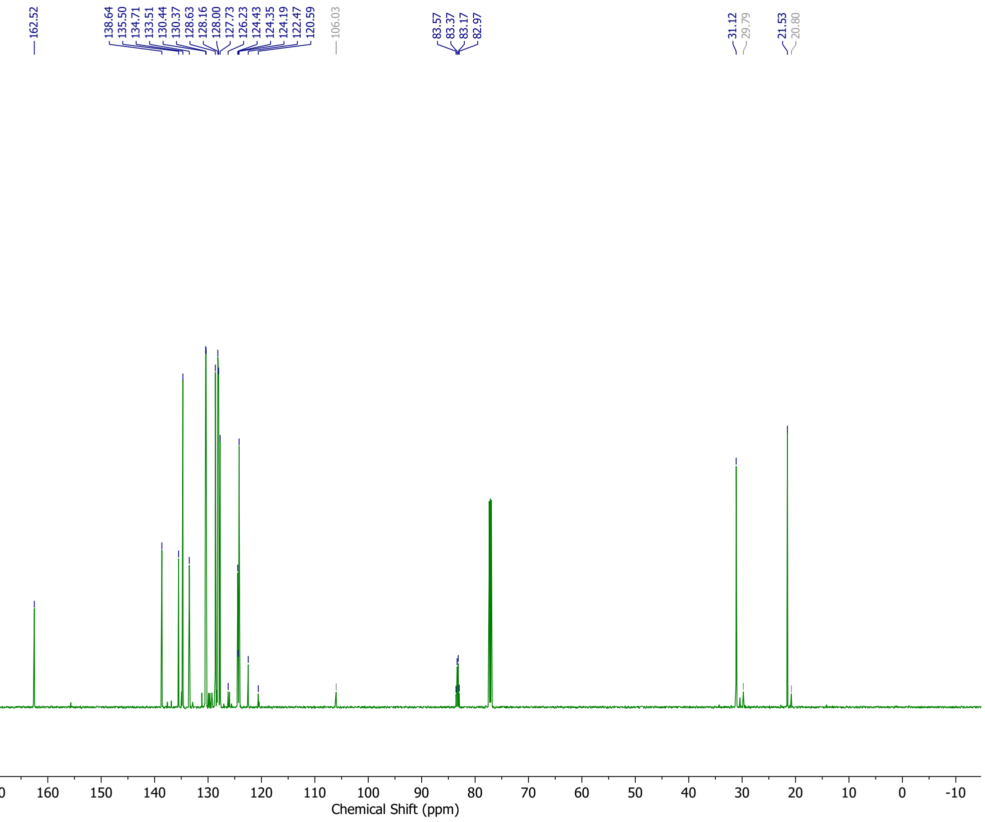
162.52
138.64 135.50 134.71 133.51 130.44 130.37 128.63 128.16 128.00 127.73 126.23 124.43 124.35 124.19 122.47 120.59
106.03
83.57 83.37 83.17 82.97
31.12 29.79
21.53 20.80
200 190 180 170 160 150 140 130 120 110 100 90 80 70 60 50 40 30 20 10 0 -10
Chemical Shift (ppm)

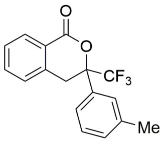

3ae

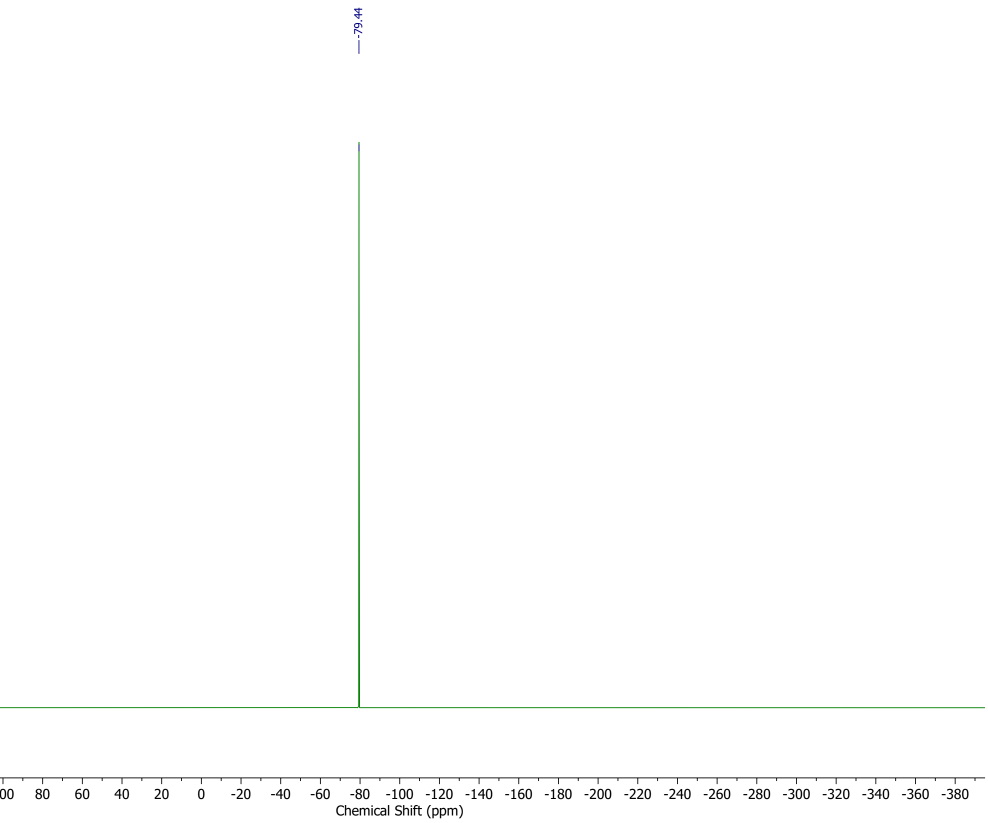

-79.44
180 160 140 120 100 80 60 40 20 0 -20 -40 -60 -80 -100 -120 -140 -160 -180 -200 -220 -240 -260 -280 -300 -320 -340 -360 -380
Chemical Shift (ppm)

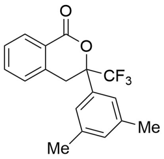

3ak

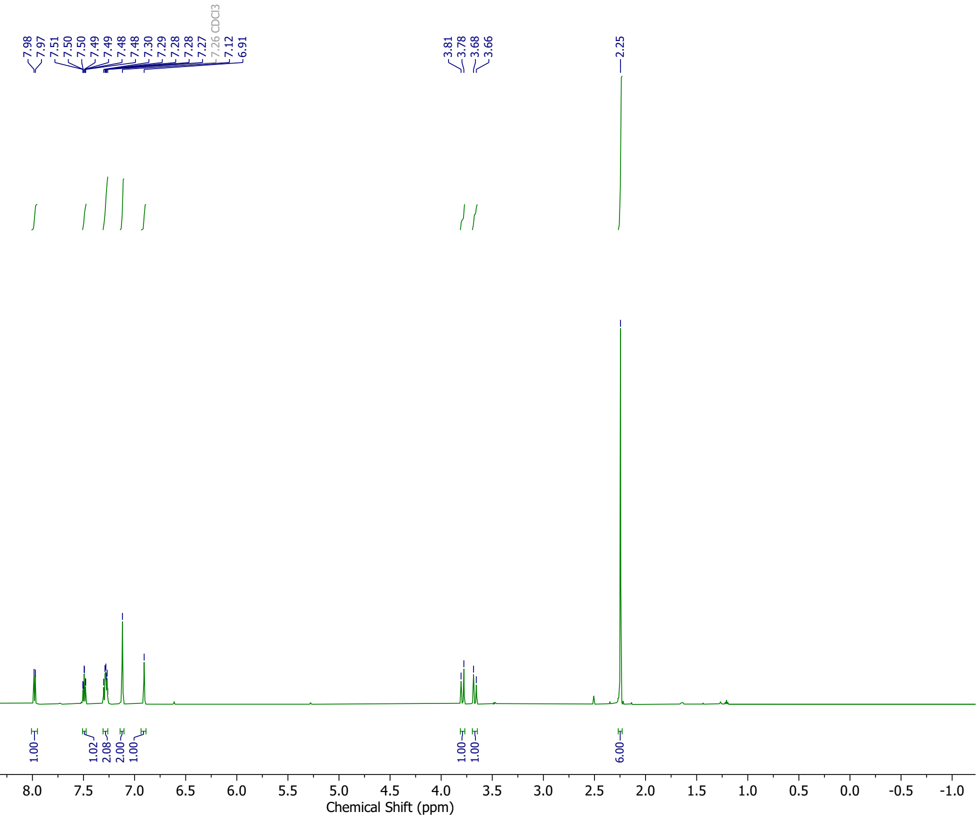

7.98
7.97
7.51
7.50
7.50
7.49
7.49
7.48
7.48
7.30
7.29
7.28
7.28
7.27
7.26 CDCl3
7.12
6.91
3.81
3.78
3.68
3.66
2.25
1.00
1.02
2.08
2.00
1.00
1.00
1.00
6.00
10.0 9.5 9.0 8.5 8.0 7.5 7.0 6.5 6.0 5.5 5.0 4.5 4.0 3.5 3.0 2.5 2.0 1.5 1.0 0.5 0.0 -0.5 -1.0
Chemical Shift (ppm)

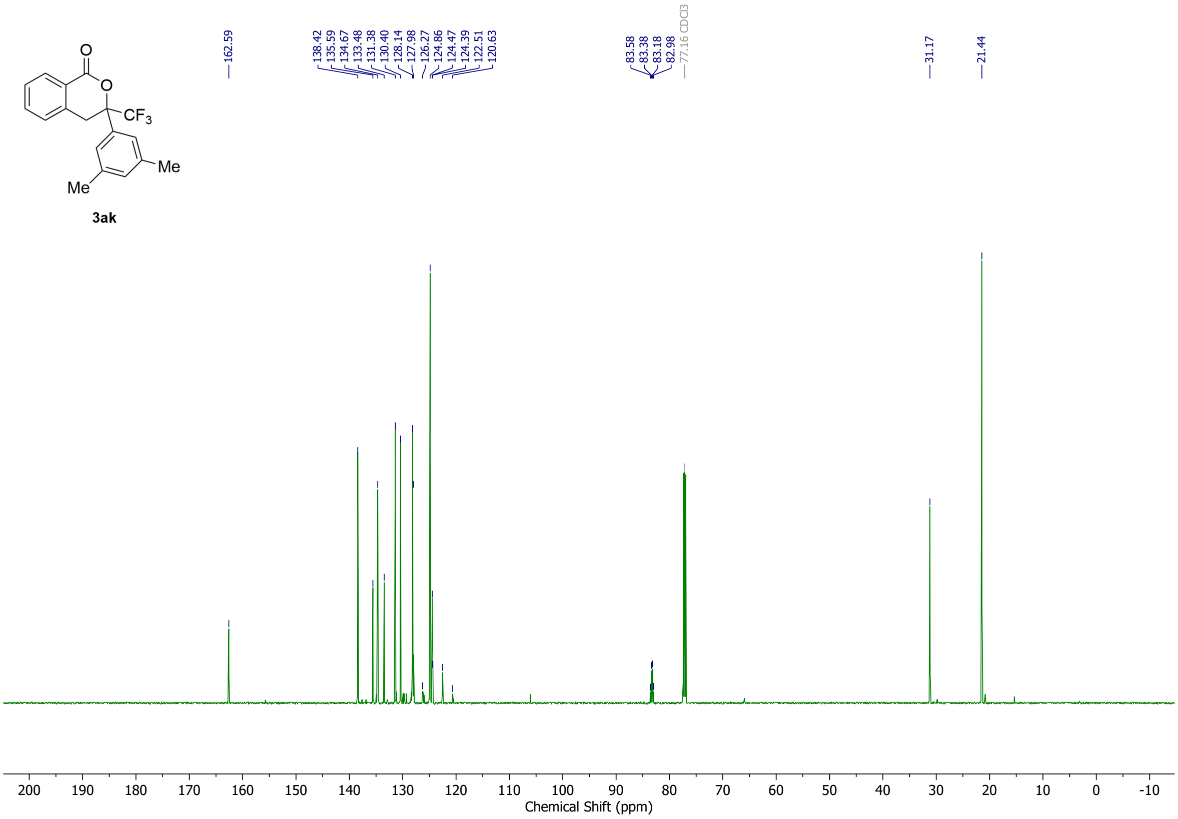

3ak
162.59
138.42
135.59
134.67
133.48
131.38
130.40
128.14
127.98
126.27
124.86
124.47
124.39
122.51
120.63
83.58
83.38
83.18
82.98
77.16 CDCl3
31.17
21.44
Chemical Shift (ppm)

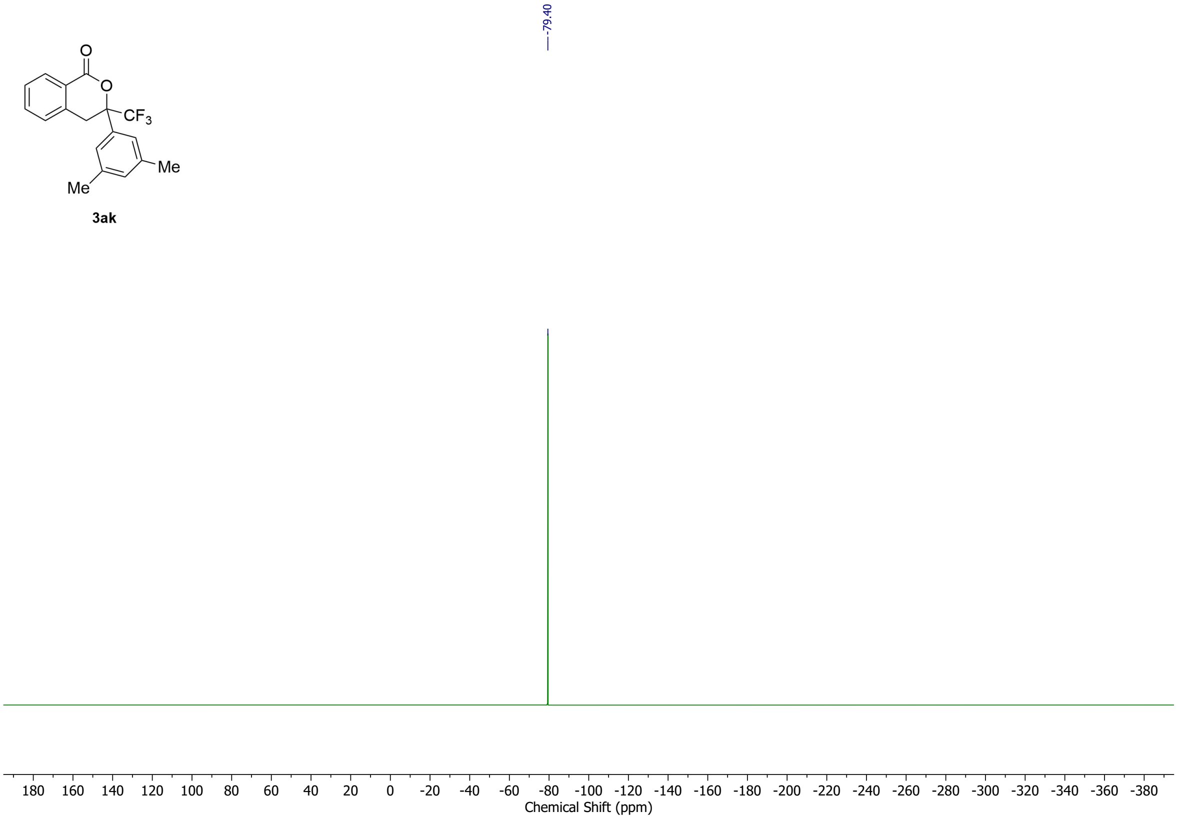

3ak
-79.40
Chemical Shift (ppm)

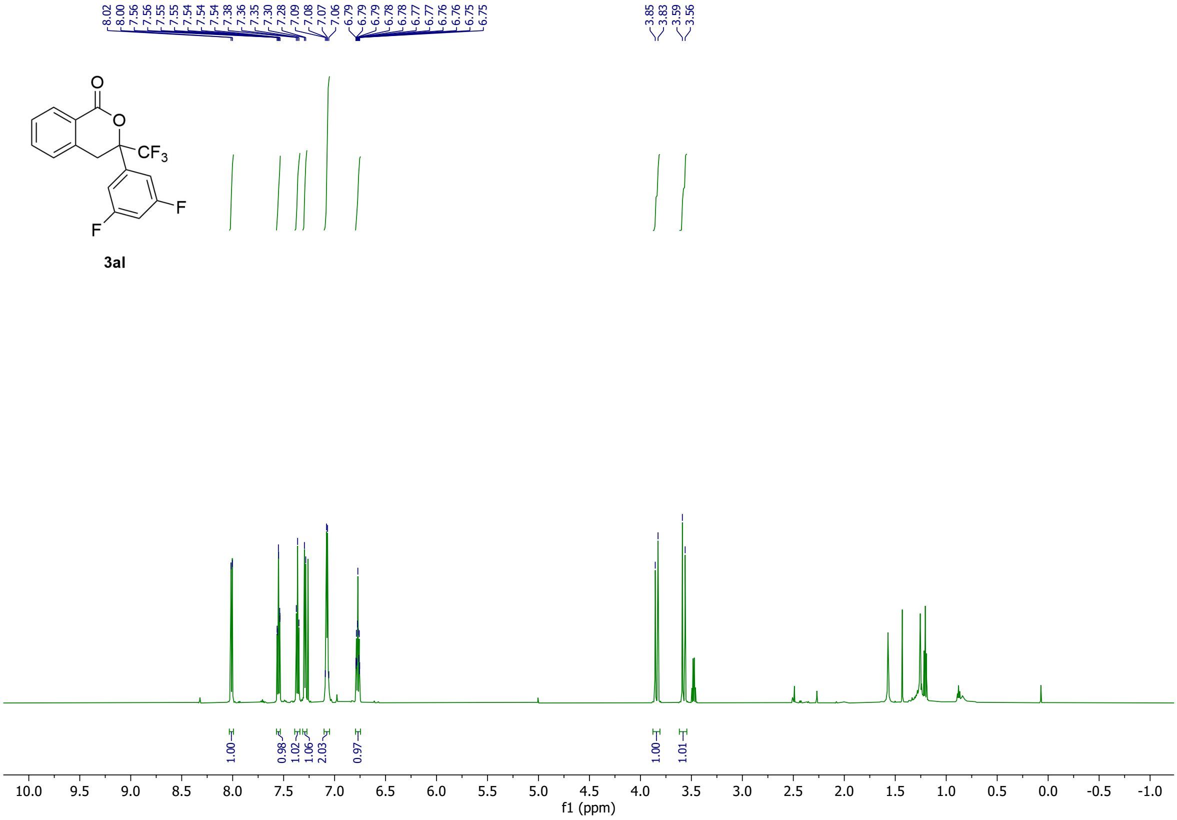
8.02 8.00 7.56 7.56 7.55 7.55 7.54 7.54 7.54 7.38 7.36 7.35 7.30 7.28 7.09 7.08 7.07 7.06 6.79 6.79 6.79 6.78 6.78 6.77 6.77 6.76 6.76 6.75 6.75
3.85 3.83 3.59 3.56
O
O
CF3
F
F
1.00
0.98
1.02
1.06
2.03
0.97
1.00
1.01
10.0 9.5 9.0 8.5 8.0 7.5 7.0 6.5 6.0 5.5 5.0 4.5 4.0 3.5 3.0 2.5 2.0 1.5 1.0 0.5 0.0 -0.5 -1.0
f1 (ppm)

3al

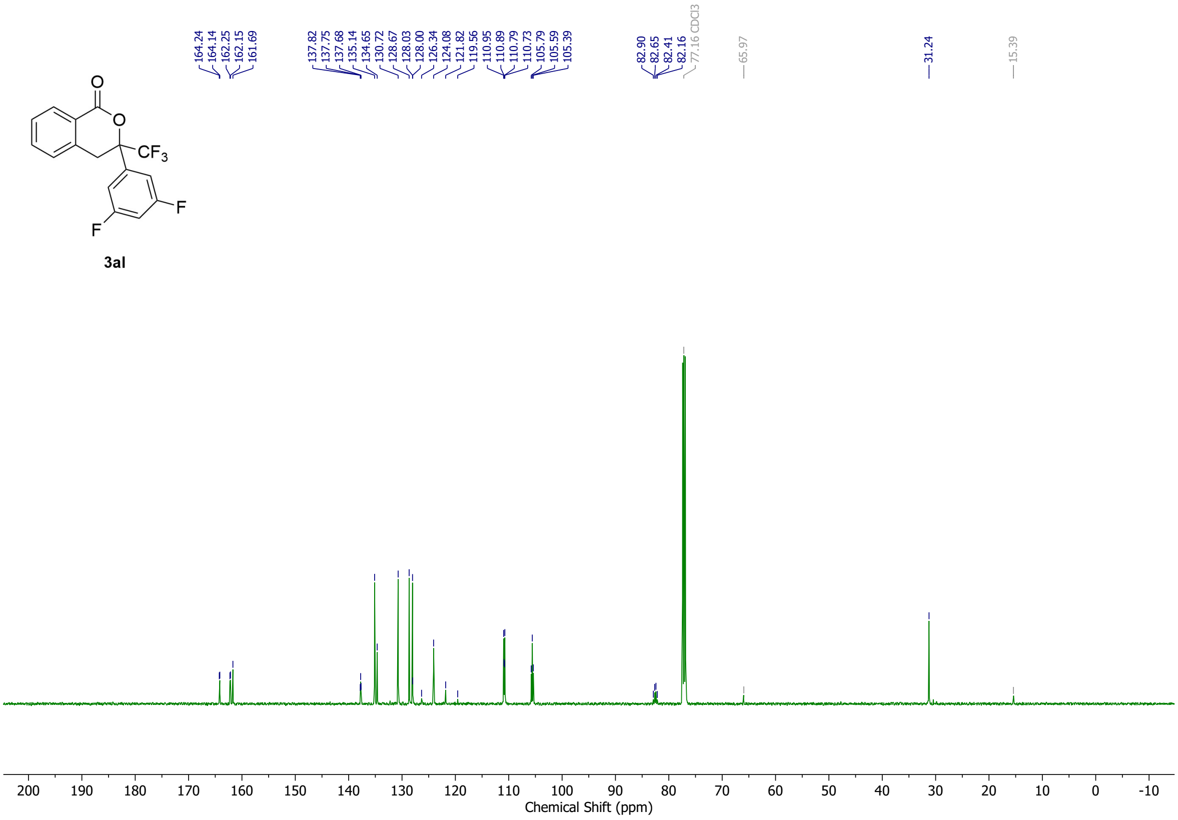
164.24 164.14 162.25 162.15 161.69
137.82 137.75 137.68 135.14 134.65 130.72 128.67 128.03 128.00 126.34 124.08 121.82 119.56 110.95 110.89 110.79 110.73 105.79 105.59 105.39
82.90 82.65 82.41 82.16
77.16 CDCl3
65.97
31.24
15.39
O
O
CF3
F
F
200 190 180 170 160 150 140 130 120 110 100 90 80 70 60 50 40 30 20 10 0 -10
Chemical Shift (ppm)

3al

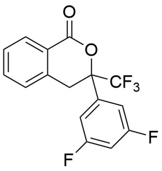

3al

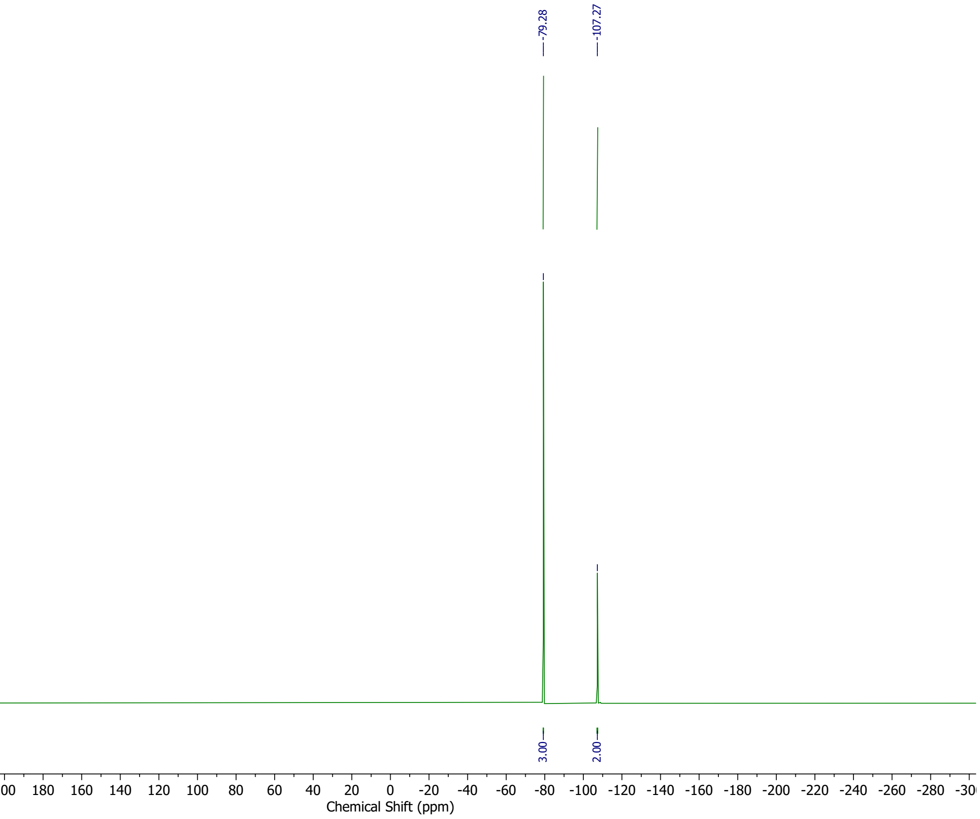

-79.28
-107.27
3.00
2.00
Chemical Shift (ppm)

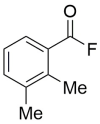

4b

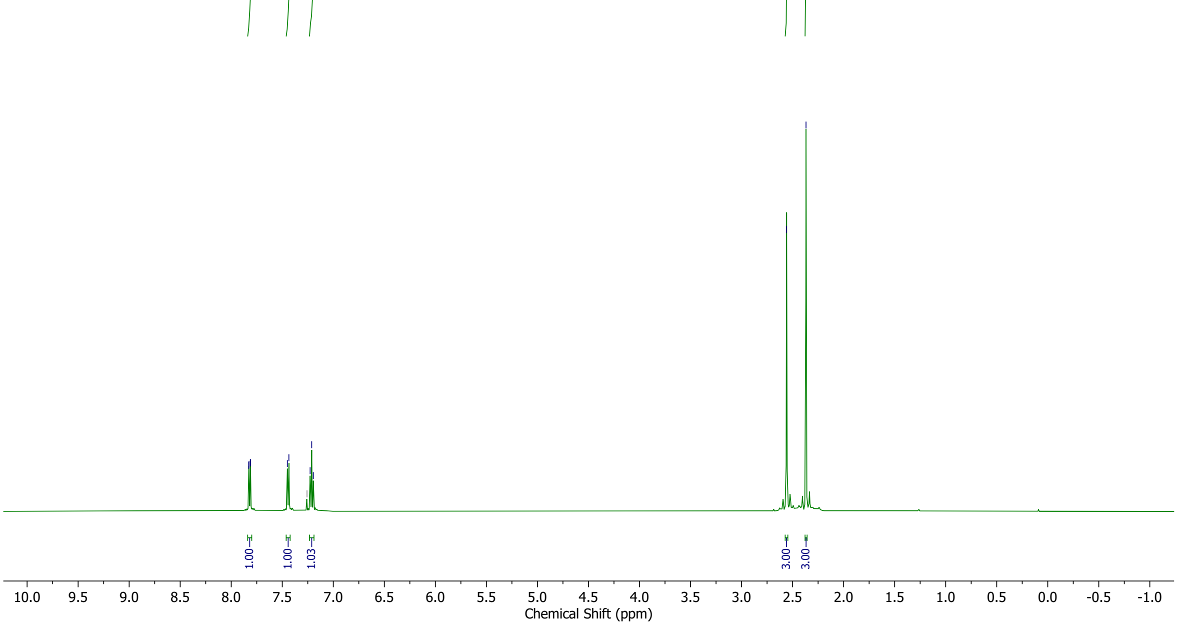

7.83
7.83
7.81
7.81
7.45
7.44
7.26 CDCl3
7.23
7.21
7.20
2.56
2.37
1.00
1.00
1.03
3.00
3.00
Chemical Shift (ppm)

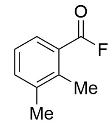
O
F
Me
Me

4b

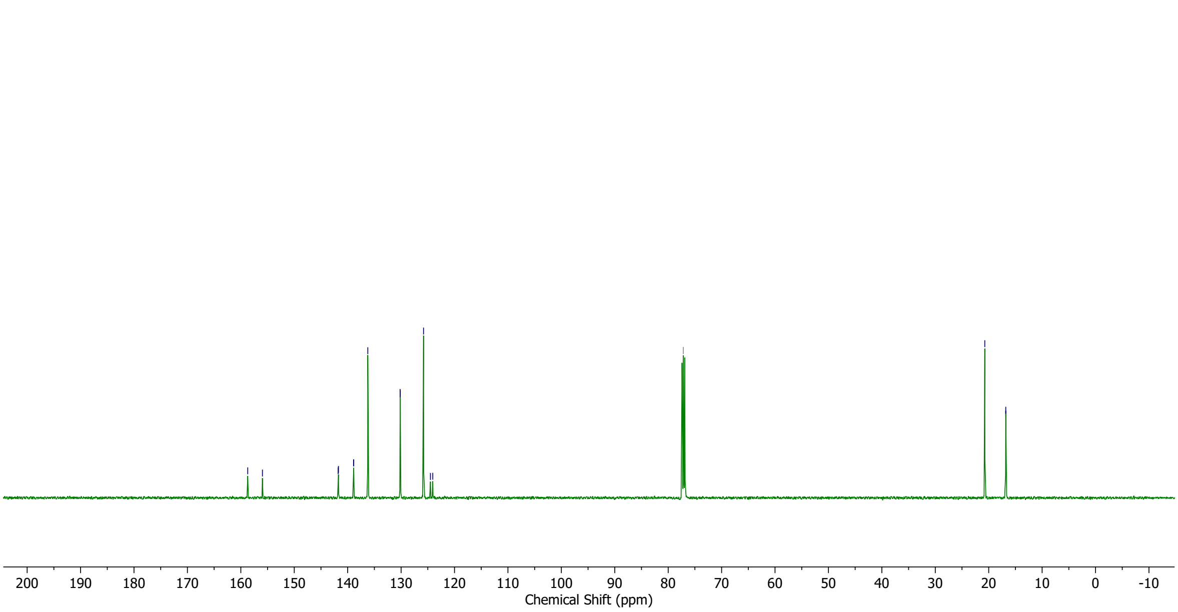
158.71
155.96
141.78
141.72
138.90
138.86
136.23
130.18
130.16
125.80
124.51
124.07
77.16 CDCl3
20.74
16.81
200 190 180 170 160 150 140 130 120 110 100 90 80 70 60 50 40 30 20 10 0 -10
Chemical Shift (ppm)

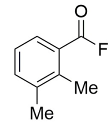
O
F
Me
Me

4b

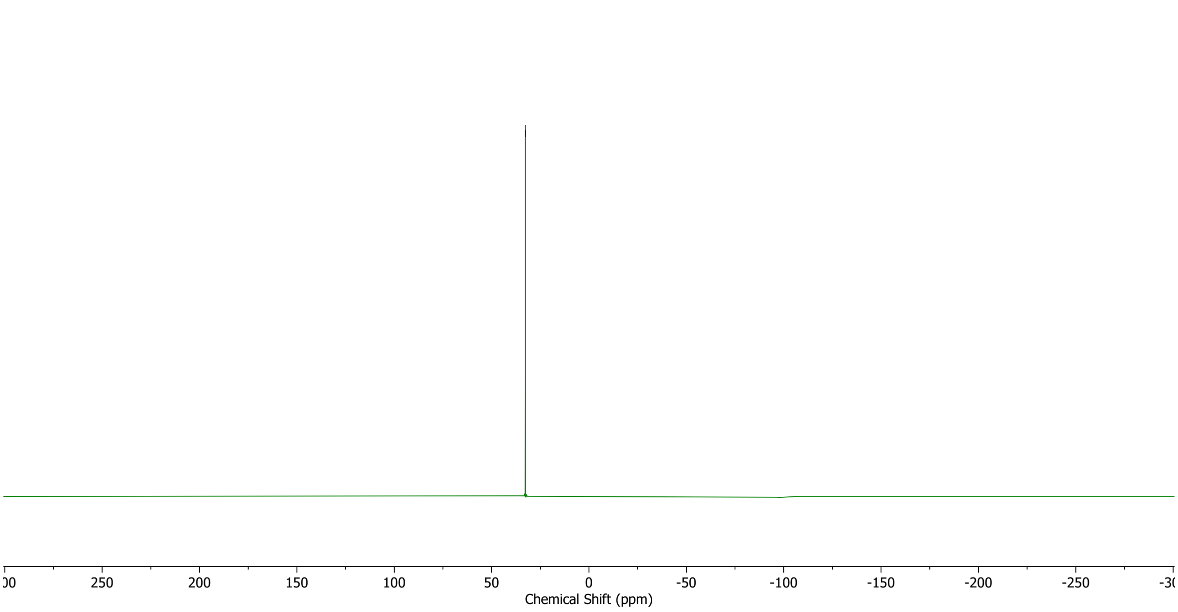
32.67
250 200 150 100 50 0 -50 -100 -150 -200 -250
Chemical Shift (ppm)

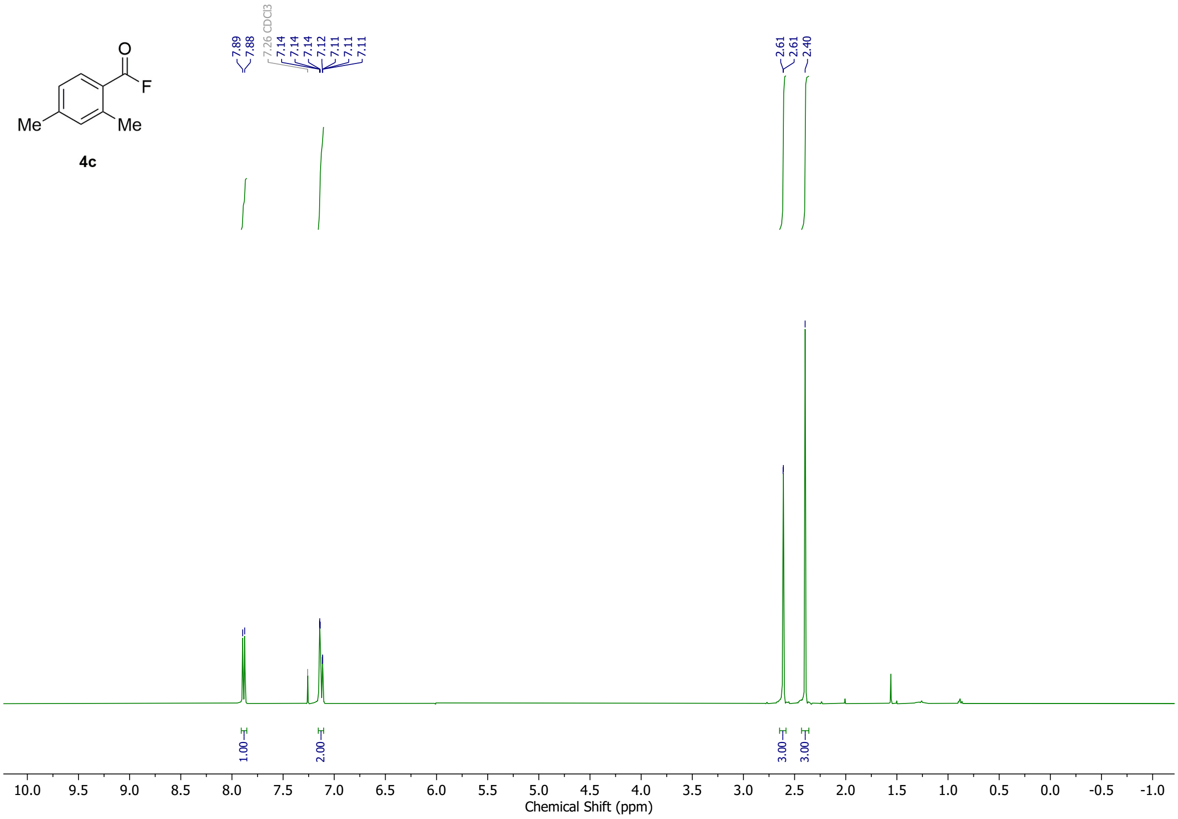
4c
7.89
7.88
7.26 CDCl3
7.14
7.14
7.14
7.12
7.11
7.11
7.11
2.61
2.61
2.40
1.00
2.00
3.00
3.00
Chemical Shift (ppm)

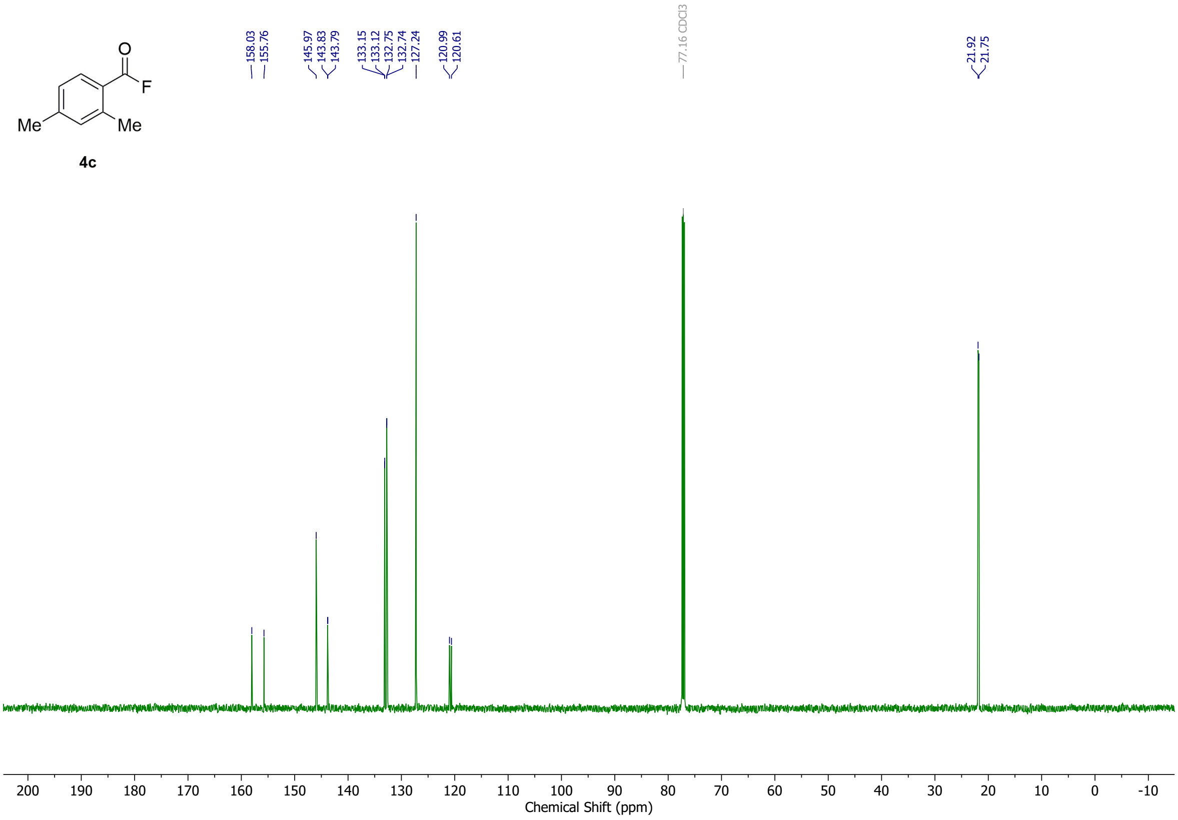
4c
158.03
155.76
145.97
143.83
143.79
133.15
133.12
132.75
132.74
127.24
120.99
120.61
77.16 CDCl3
21.92
21.75
Chemical Shift (ppm)

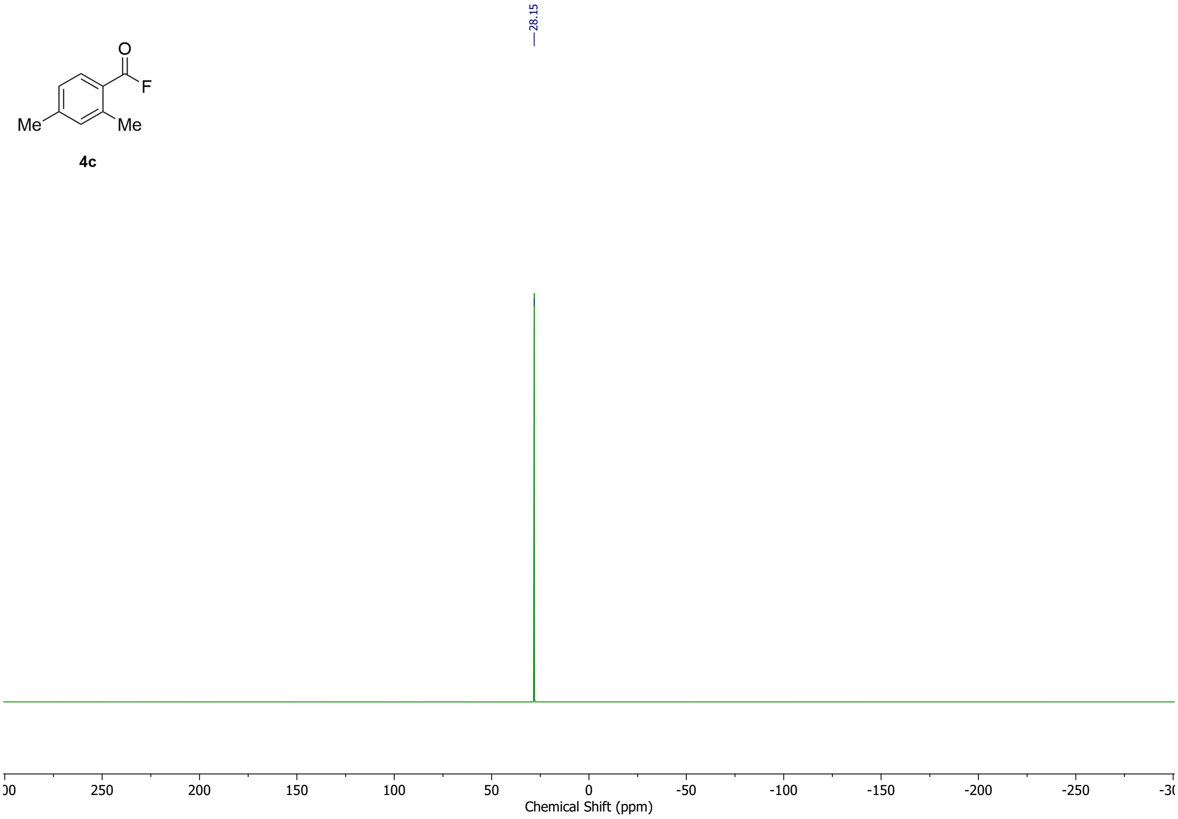

Me
Me
O
F
4c
28.15
Chemical Shift (ppm)

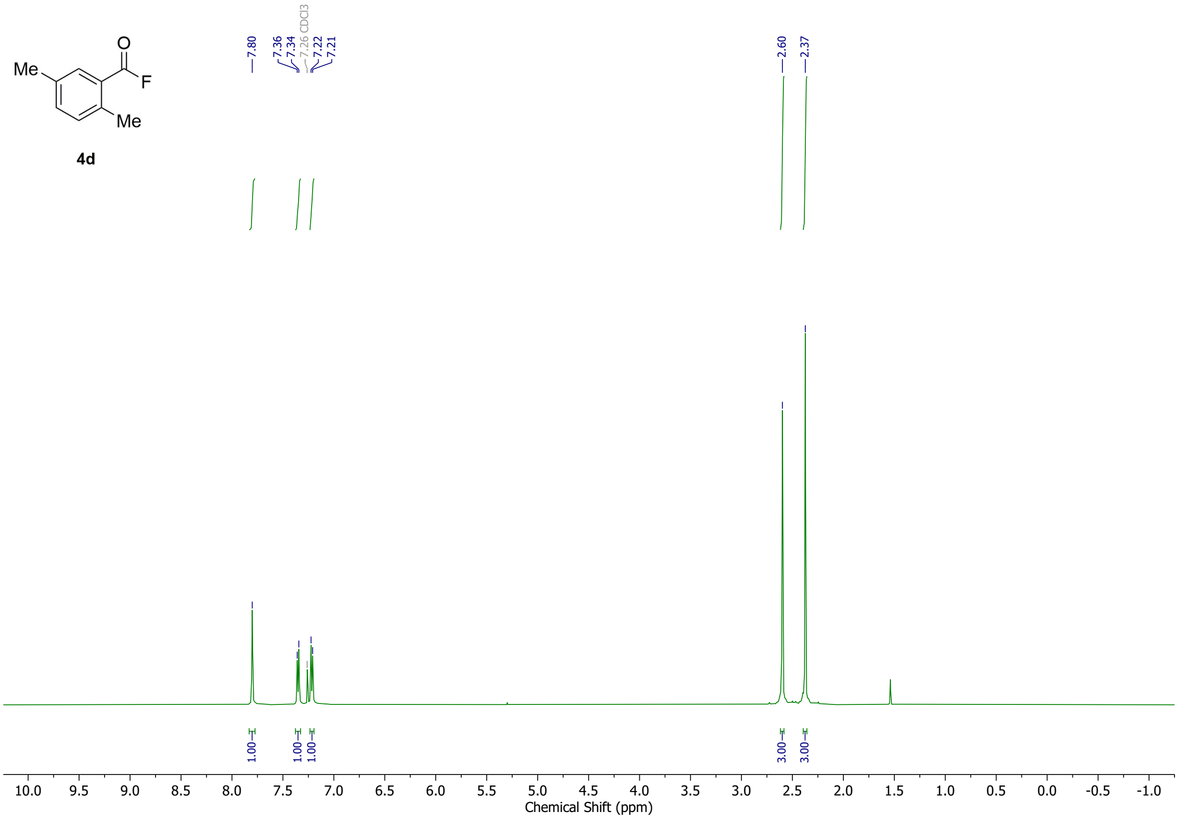

Me
Me
O
F
4d
7.80
7.36
7.34
7.26 CDCl3
7.22
7.21
2.60
2.37
1.00
1.00
1.00
3.00
3.00
Chemical Shift (ppm)

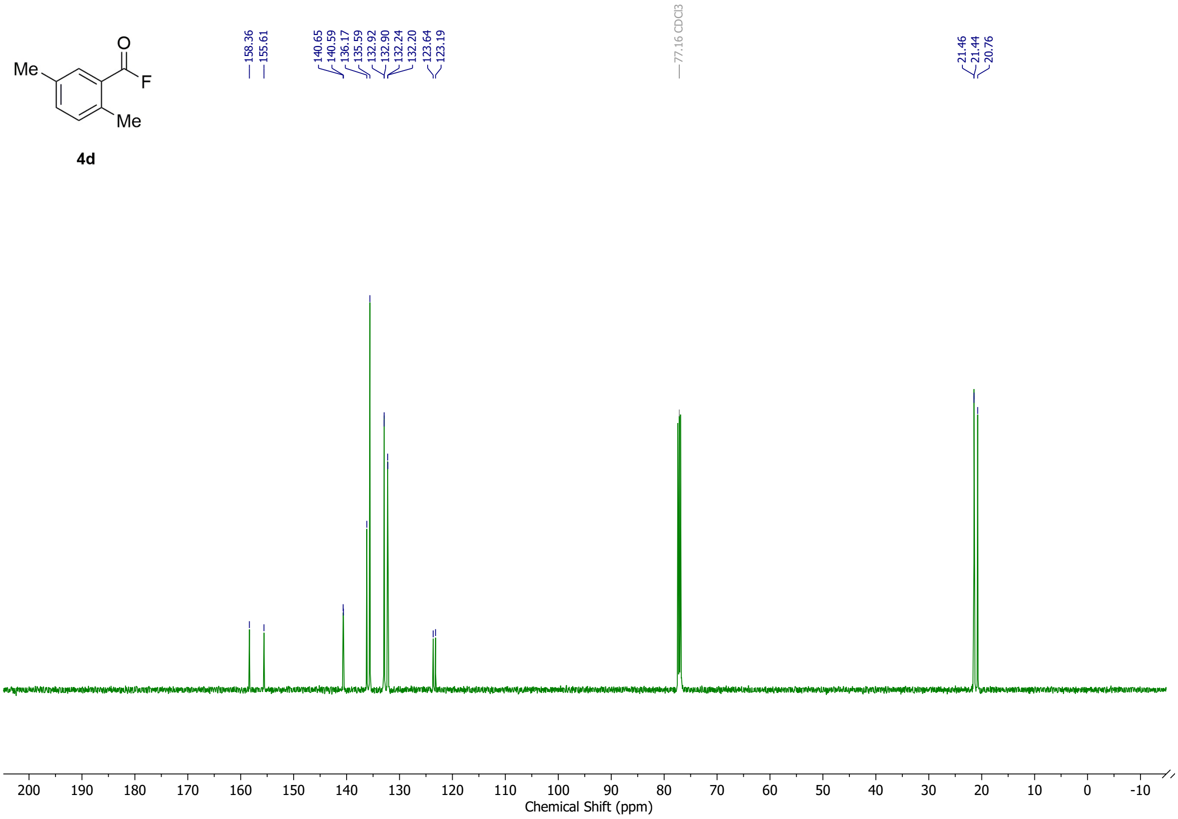
Me
O
F
Me
4d
158.36
155.61
140.65
140.59
136.17
135.59
132.92
132.90
132.24
132.20
123.64
123.19
77.16 CDCl3
21.46
21.44
20.76
200 190 180 170 160 150 140 130 120 110 100 90 80 70 60 50 40 30 20 10 0 -10
Chemical Shift (ppm)

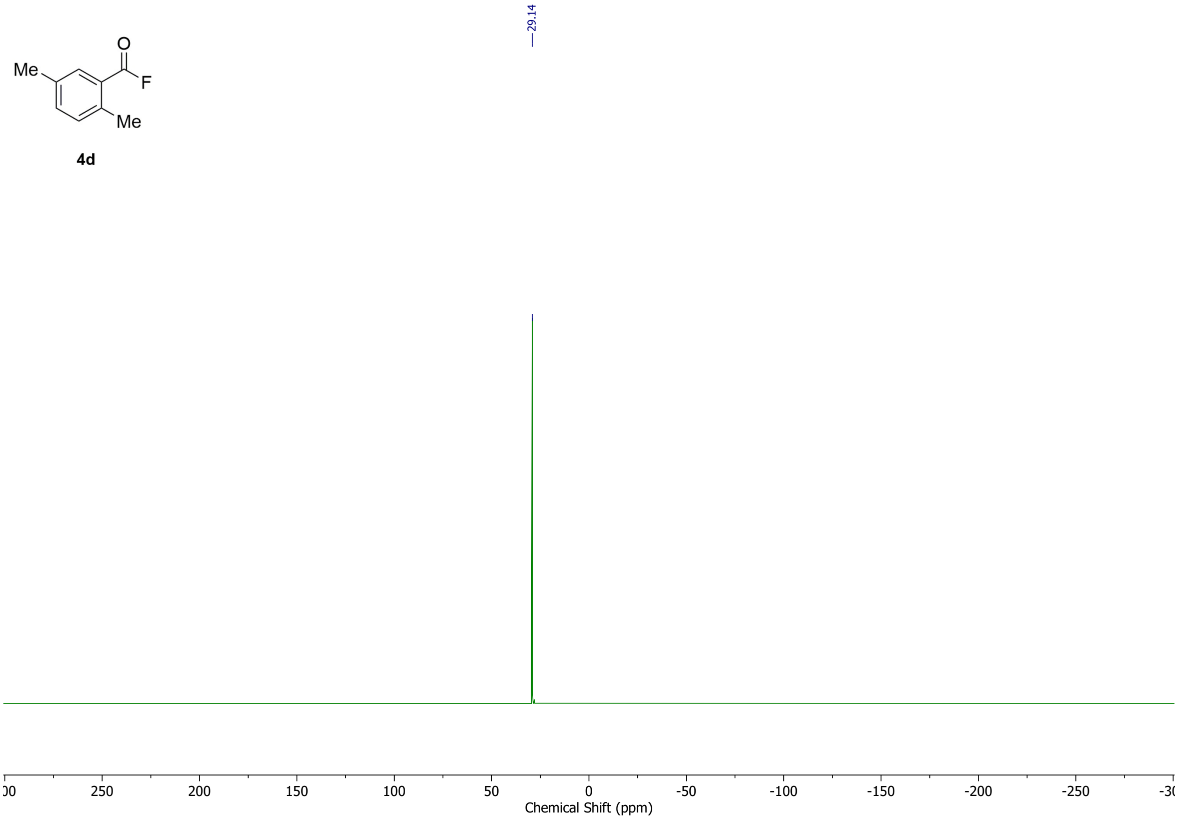
Me
O
F
Me
4d
29.14
250 200 150 100 50 0 -50 -100 -150 -200 -250
Chemical Shift (ppm)

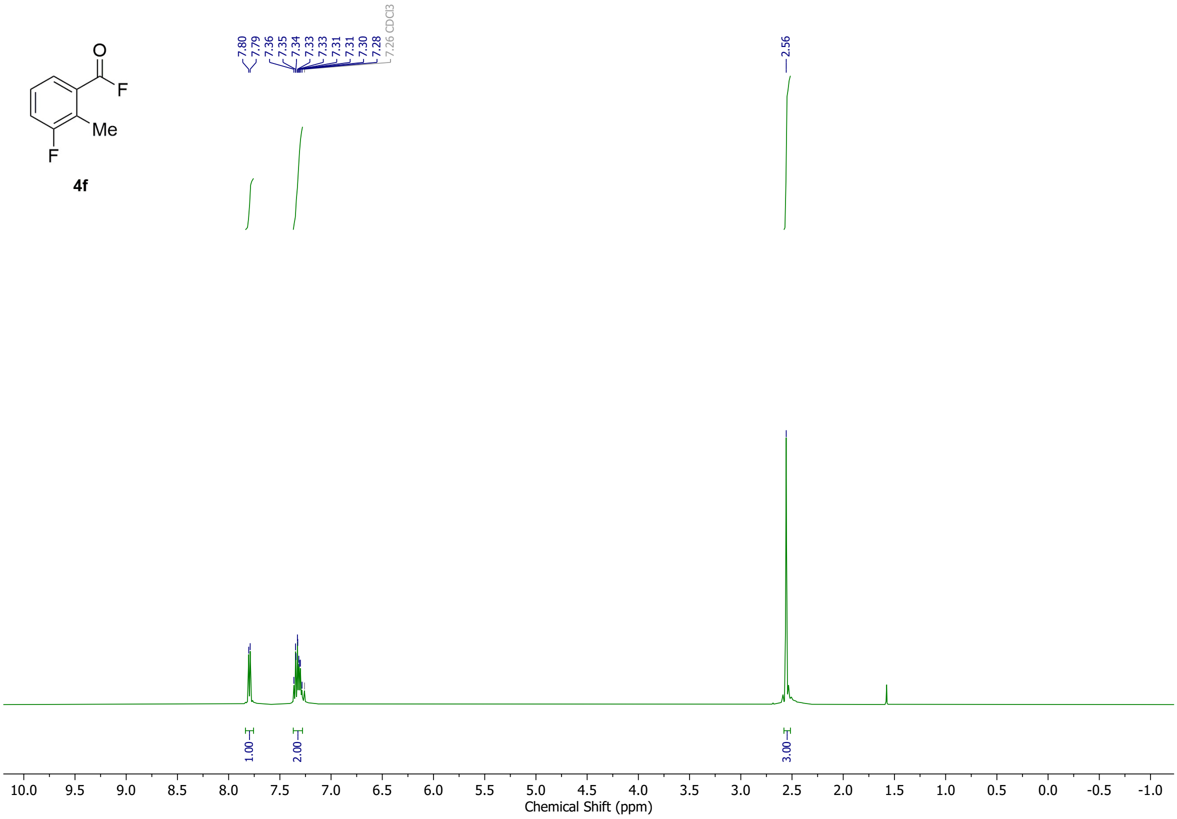
7.80
7.79
7.36
7.35
7.34
7.33
7.33
7.31
7.31
7.30
7.28
7.26 CDCl3
2.56
4f
1.00
2.00
3.00
Chemical Shift (ppm)

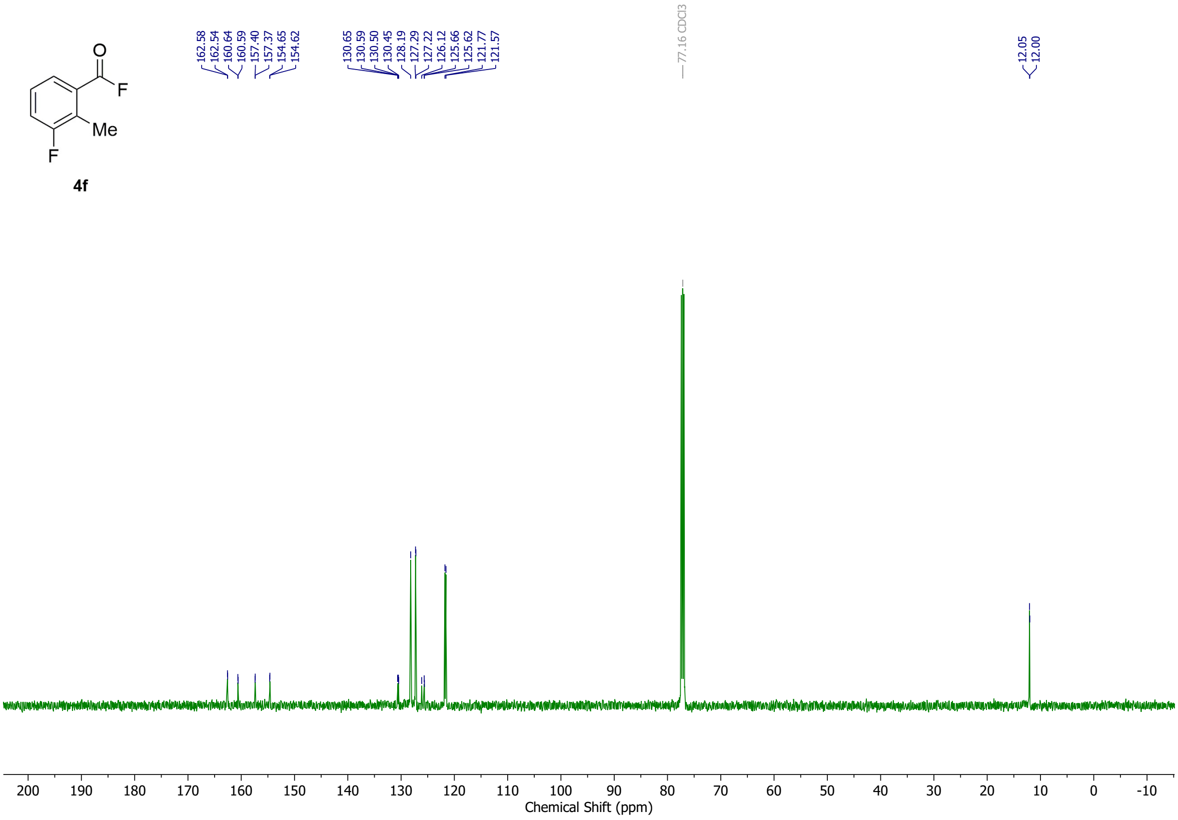
162.58
162.54
160.64
160.59
157.40
157.37
154.65
154.62
130.65
130.59
130.50
130.45
128.19
127.29
127.22
126.12
125.66
125.62
121.77
121.57
77.16 CDCl3
12.05
12.00
4f
Chemical Shift (ppm)

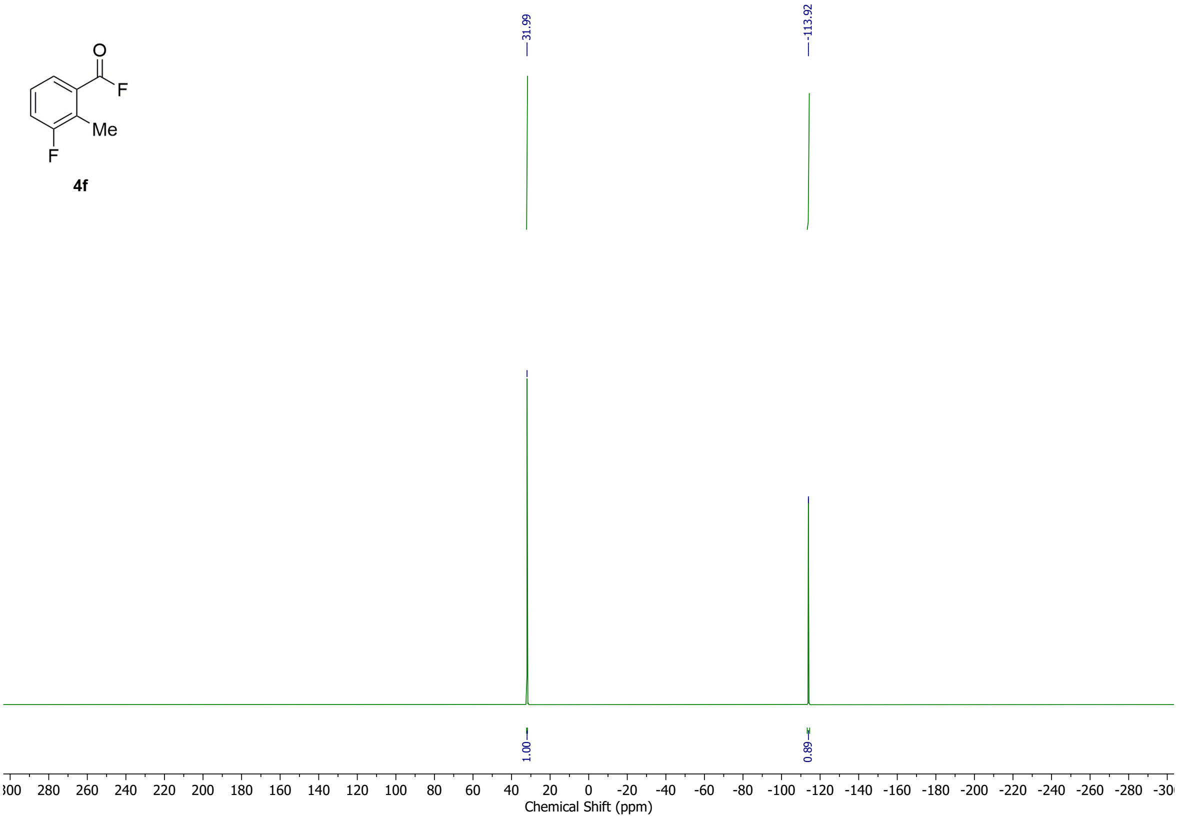

4f
31.99
-113.92
1.00
0.89
300 280 260 240 220 200 180 160 140 120 100 80 60 40 20 0 -20 -40 -60 -80 -100 -120 -140 -160 -180 -200 -220 -240 -260 -280 -300
Chemical Shift (ppm)

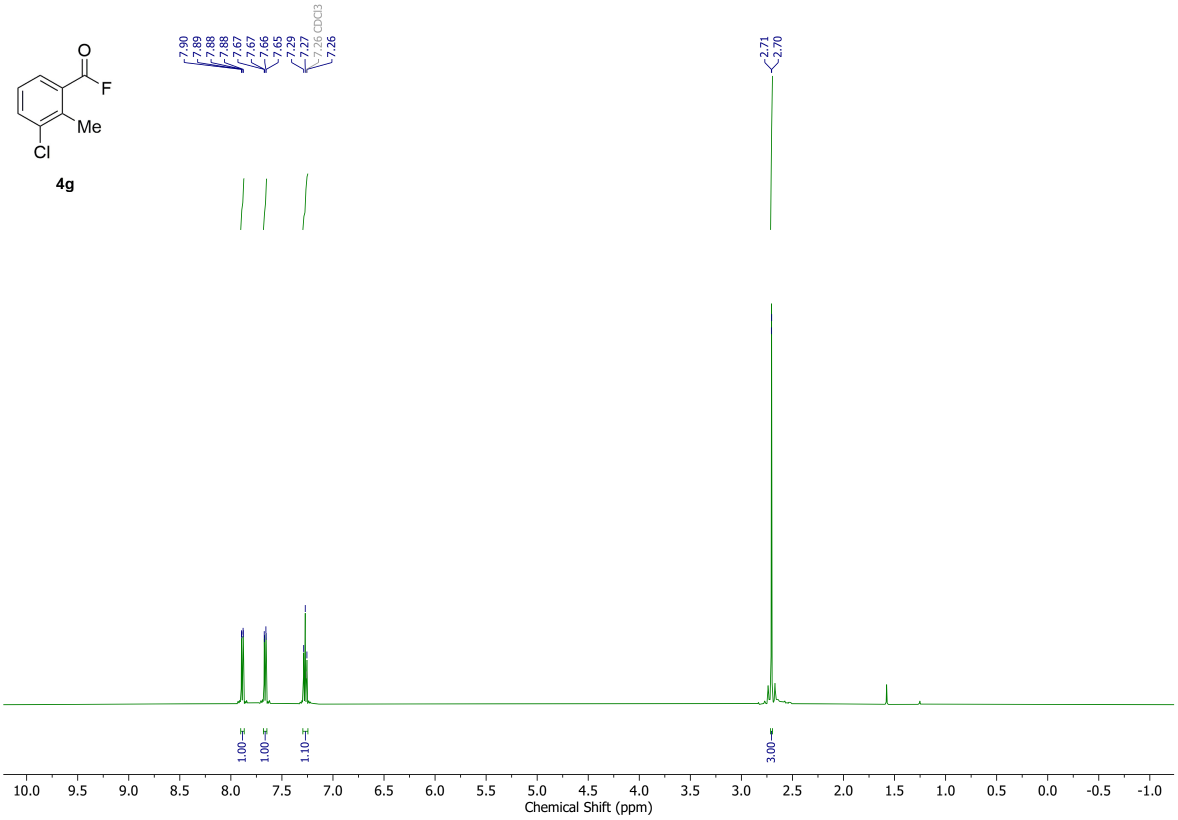

4g
7.90 7.89 7.88 7.88 7.67 7.67 7.66 7.65 7.29 7.27 7.26 CDCl3 7.26
2.71 2.70
1.00 1.00 1.10 3.00
10.0 9.5 9.0 8.5 8.0 7.5 7.0 6.5 6.0 5.5 5.0 4.5 4.0 3.5 3.0 2.5 2.0 1.5 1.0 0.5 0.0 -0.5 -1.0
Chemical Shift (ppm)

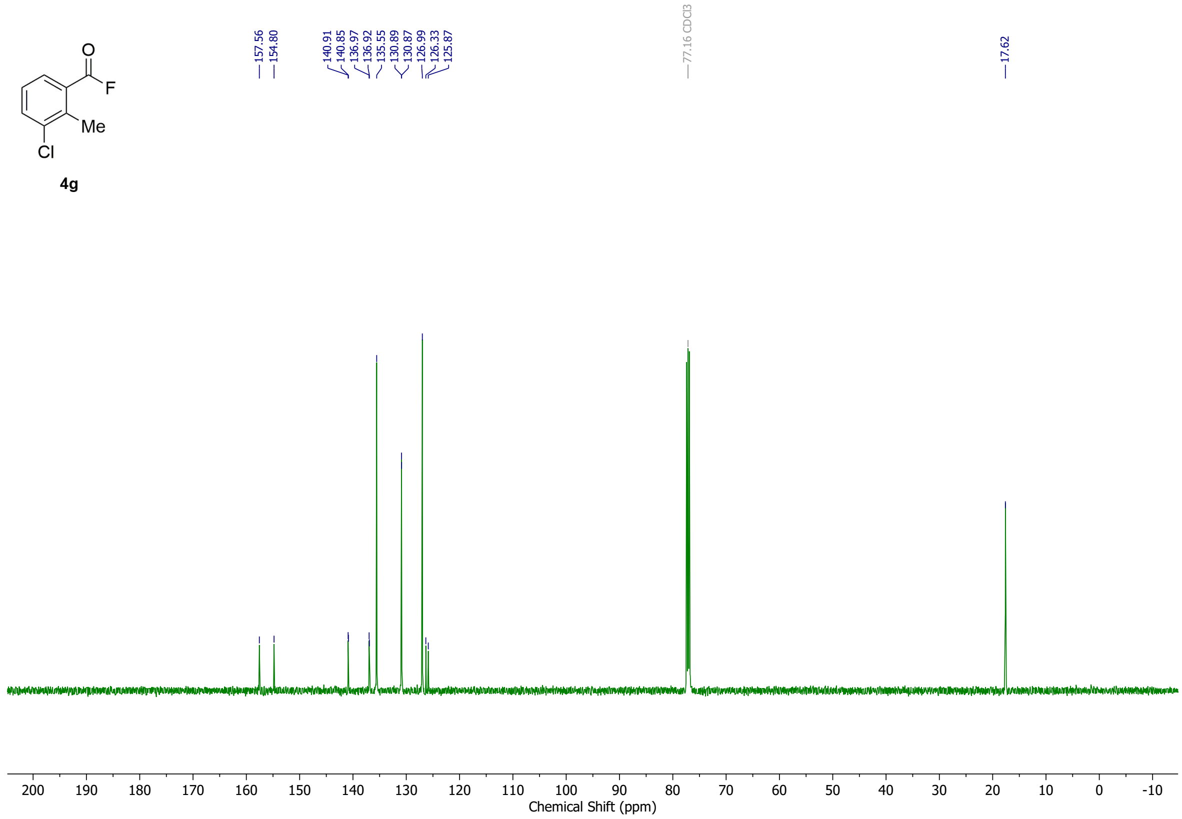
157.56
154.80
140.91
140.85
136.97
136.92
135.55
130.89
130.87
126.99
126.33
125.87
77.16 CDCl3
17.62
O
F
Me
Cl
4g
200 190 180 170 160 150 140 130 120 110 100 90 80 70 60 50 40 30 20 10 0 -10
Chemical Shift (ppm)

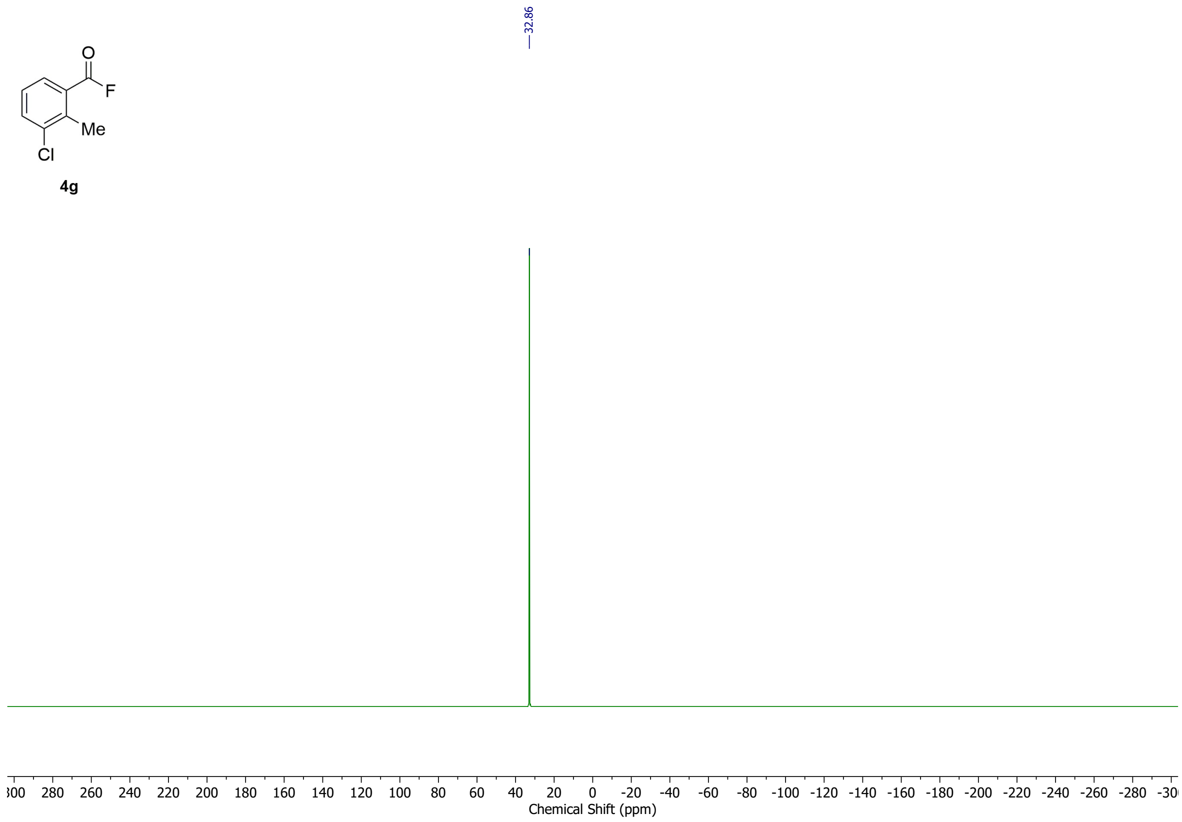
32.86
O
F
Me
Cl
4g
280 260 240 220 200 180 160 140 120 100 80 60 40 20 0 -20 -40 -60 -80 -100 -120 -140 -160 -180 -200 -220 -240 -260 -280
Chemical Shift (ppm)

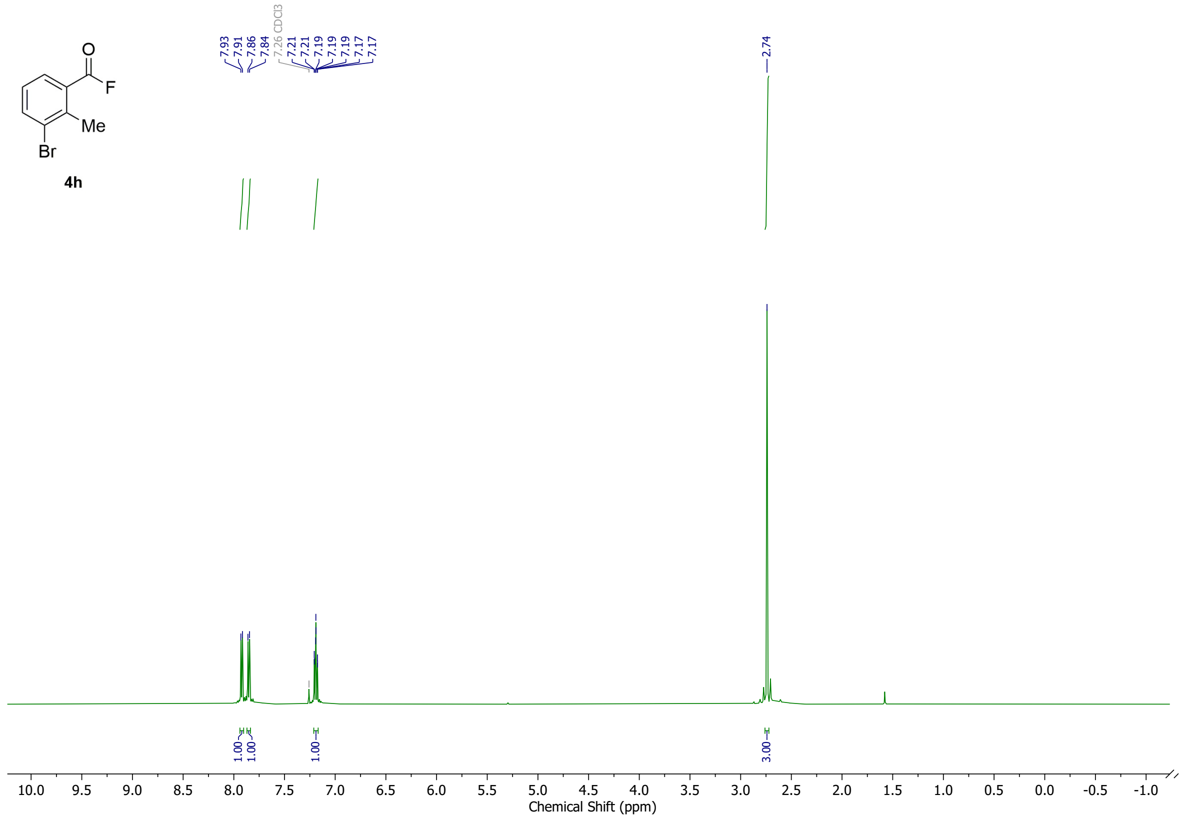
O
F
Me
Br
4h
7.93
7.91
7.86
7.84
7.26 CDCl3
7.21
7.21
7.19
7.19
7.19
7.19
7.17
7.17
2.74
1.00
1.00
1.00
3.00
Chemical Shift (ppm)

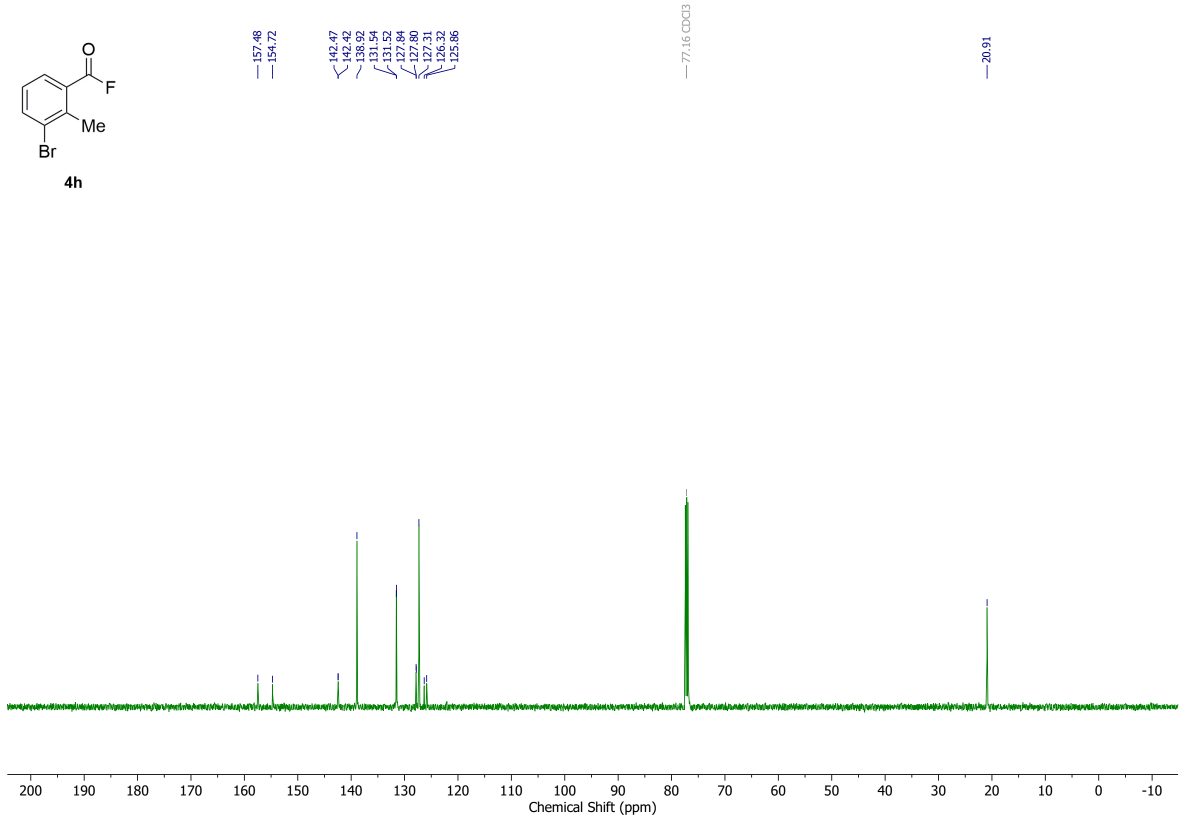
O
F
Me
Br
4h
157.48
154.72
142.47
142.42
138.92
131.54
131.52
127.84
127.80
127.31
126.32
125.86
77.16 CDCl3
20.91
Chemical Shift (ppm)

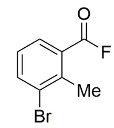

4h

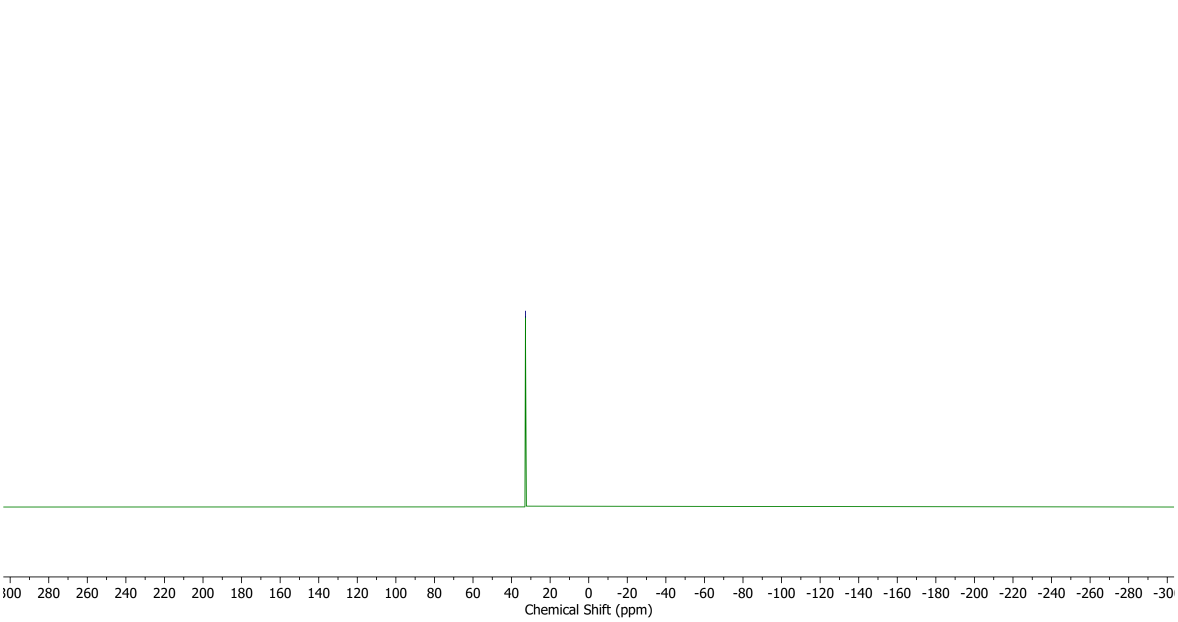

32.81
Chemical Shift (ppm)

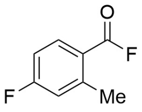

4i

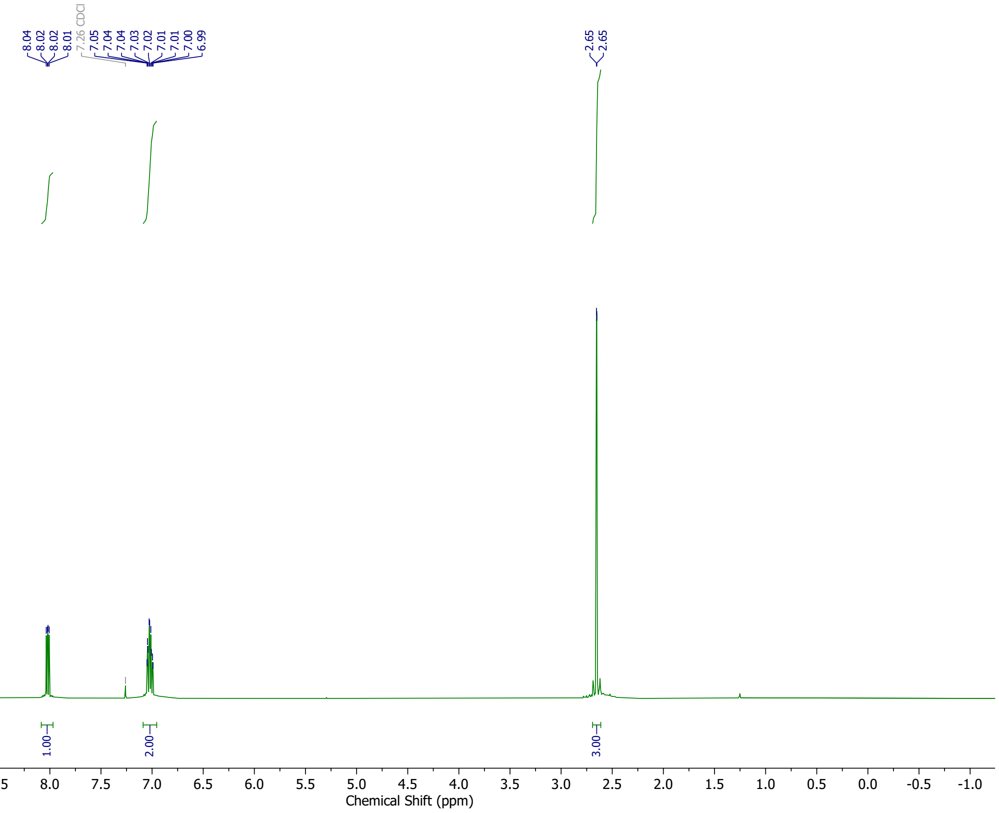

8.04
8.02
8.02
8.01
7.26 CDCl
7.05
7.04
7.04
7.03
7.02
7.01
7.01
7.00
6.99
2.65
2.65
1.00
2.00
3.00
Chemical Shift (ppm)

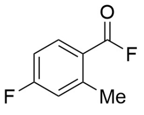

4i

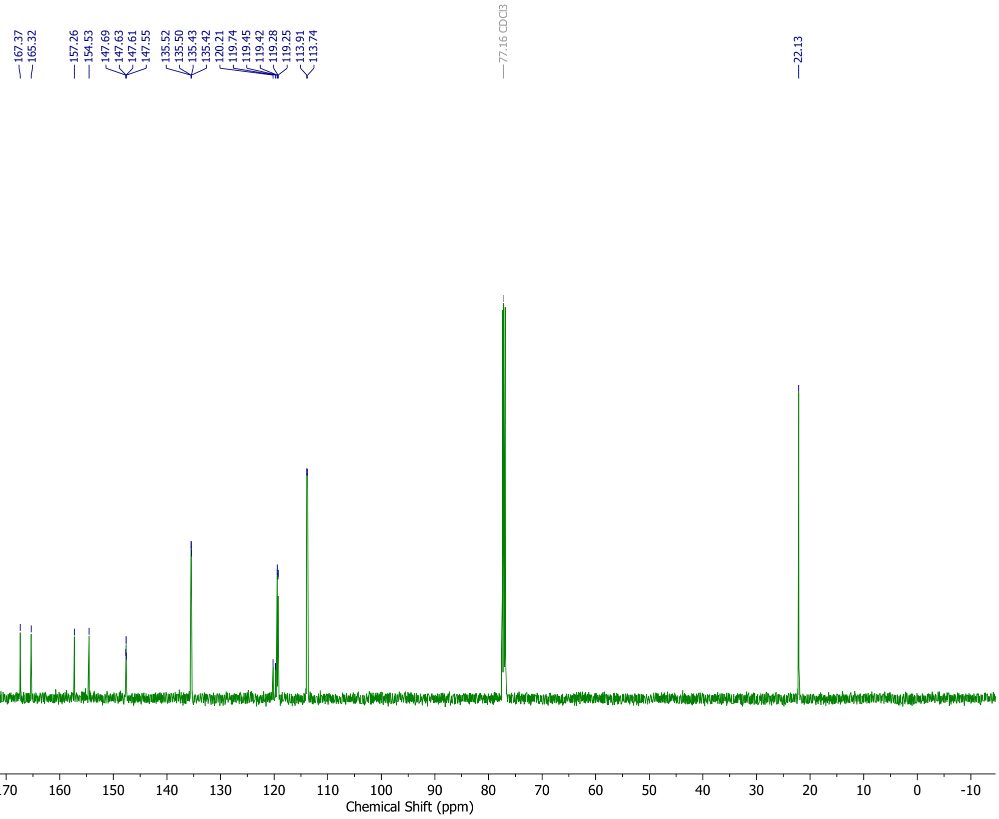

167.37
165.32
157.26
154.53
147.69
147.63
147.61
147.55
135.52
135.50
135.43
135.42
120.21
119.74
119.45
119.42
119.28
119.25
113.91
113.74
77.16 CDCl3
22.13
Chemical Shift (ppm)

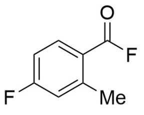

4i

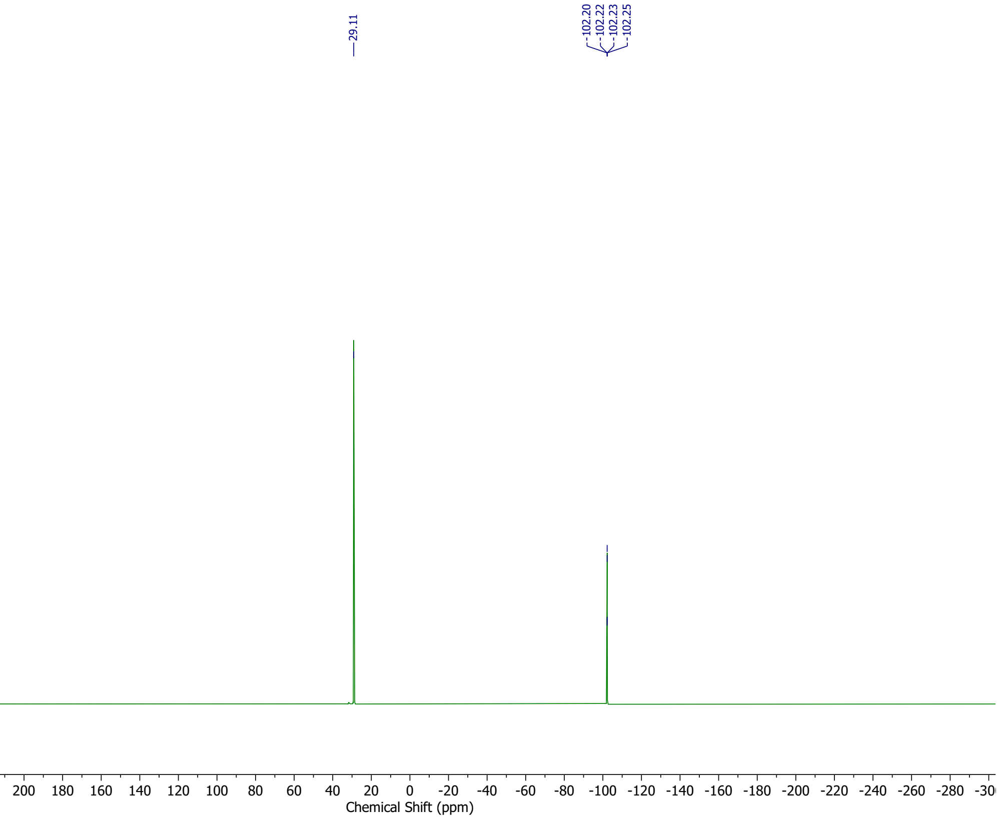

29.11
-102.20
-102.22
-102.23
-102.25
Chemical Shift (ppm)

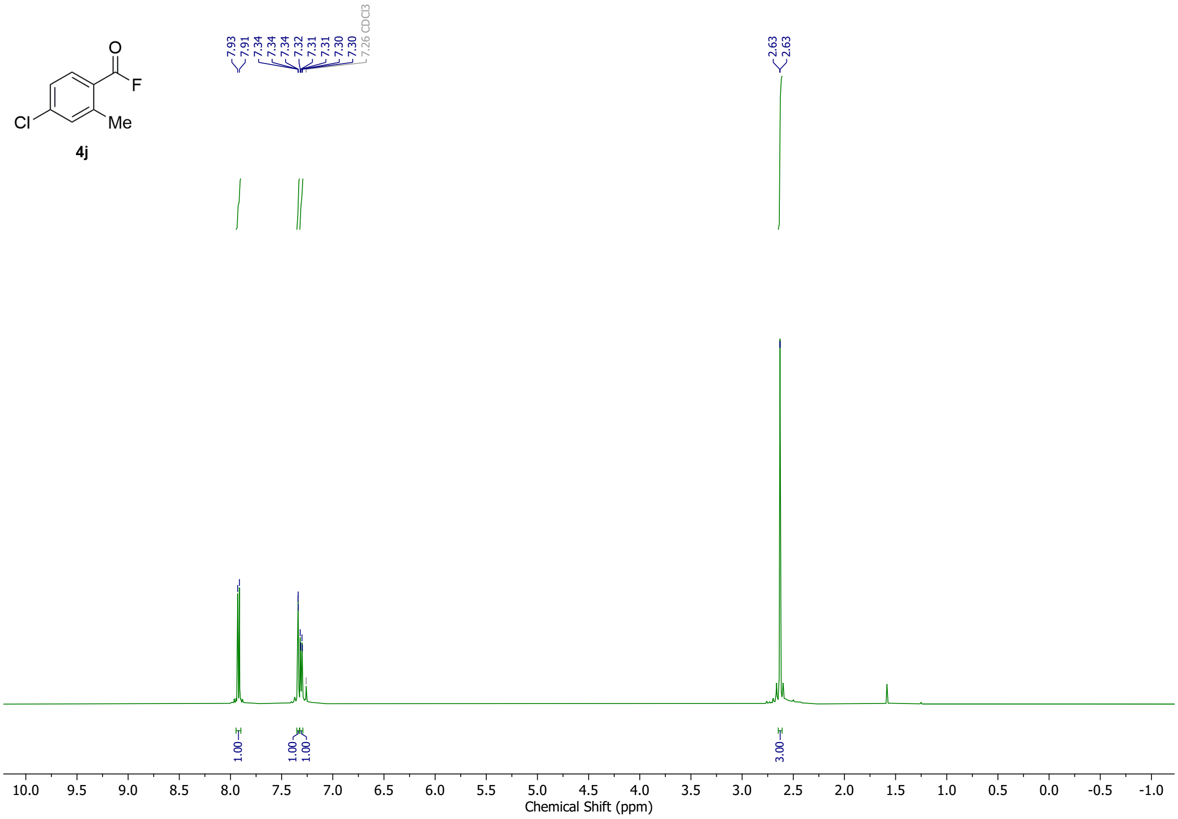
O
F
Cl
Me
4j
7.93
7.91
7.34
7.34
7.34
7.32
7.31
7.31
7.30
7.30
7.26 CDCl3
2.63
2.63
1.00
1.00
1.00
3.00
Chemical Shift (ppm)

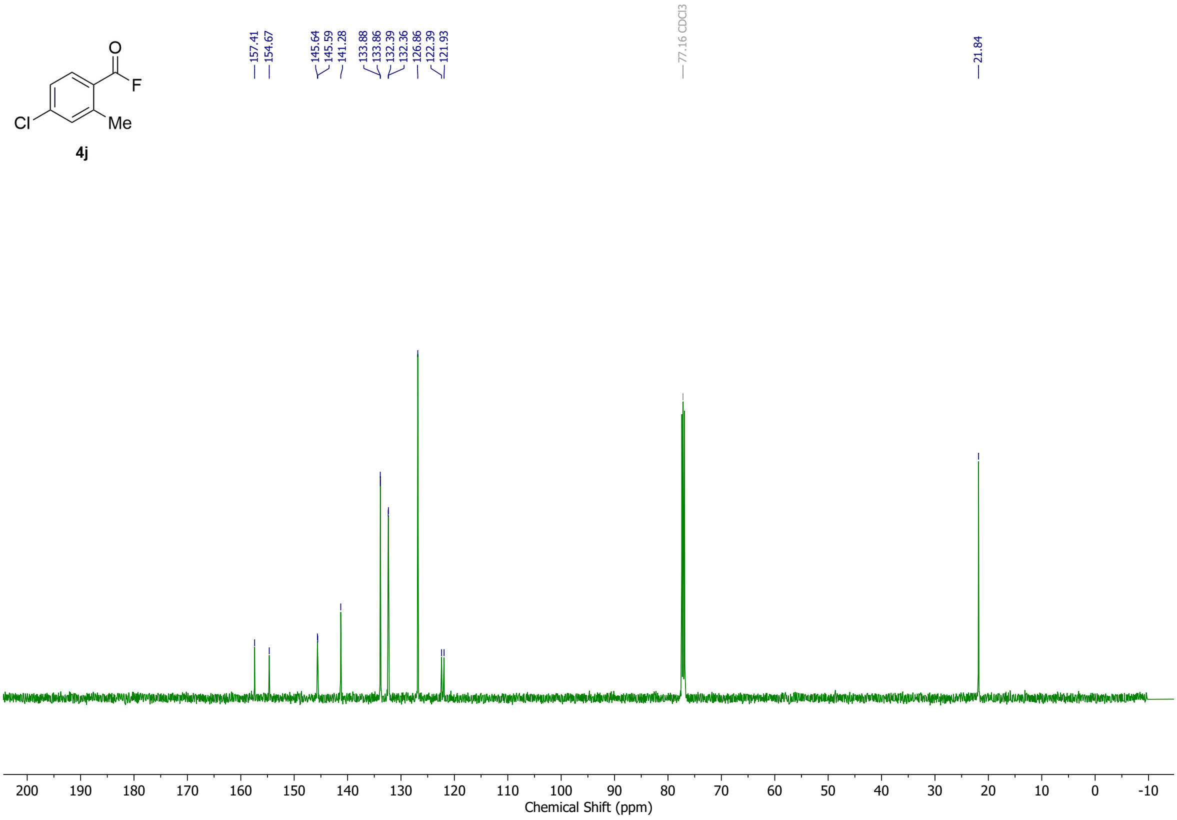
O
F
Cl
Me
4j
157.41
154.67
145.64
145.59
141.28
133.88
133.86
132.39
132.36
126.86
122.39
121.93
77.16 CDCl3
21.84
Chemical Shift (ppm)

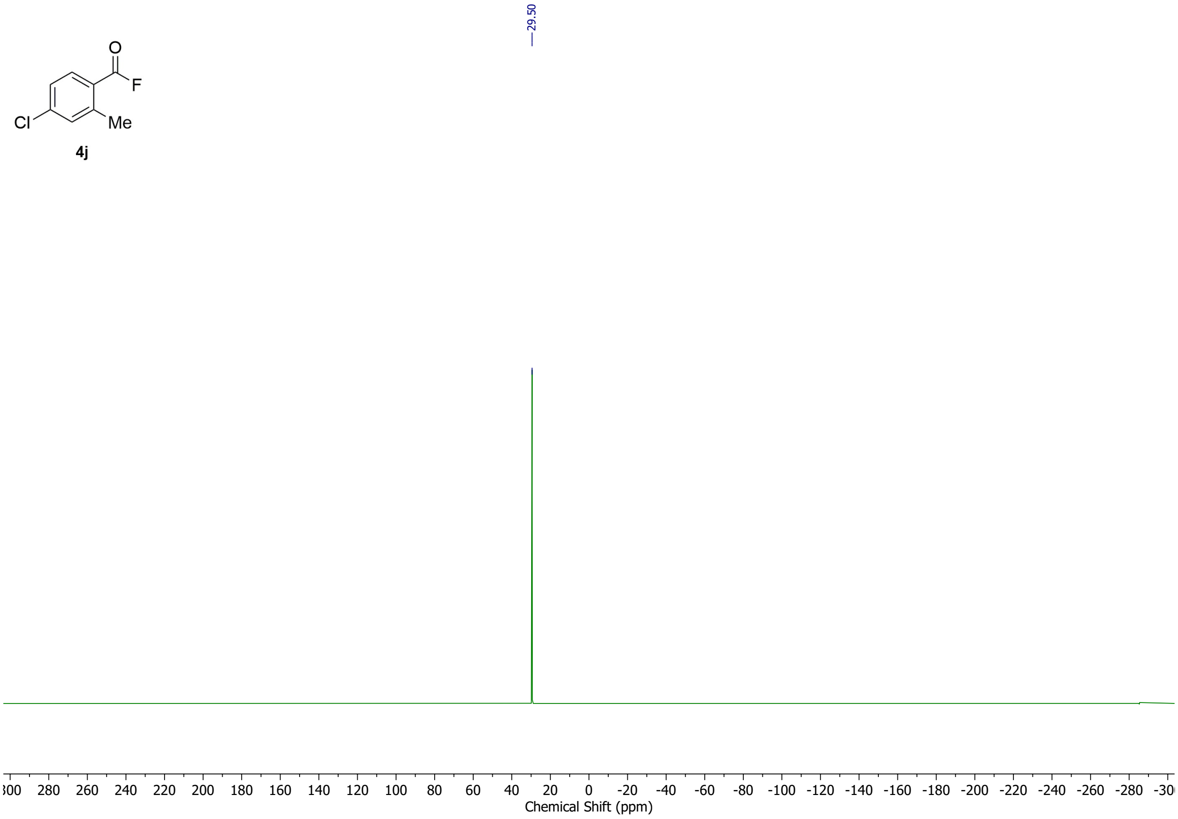
O
F
Cl
Me
4j
29.50
300 280 260 240 220 200 180 160 140 120 100 80 60 40 20 0 -20 -40 -60 -80 -100 -120 -140 -160 -180 -200 -220 -240 -260 -280 -300
Chemical Shift (ppm)

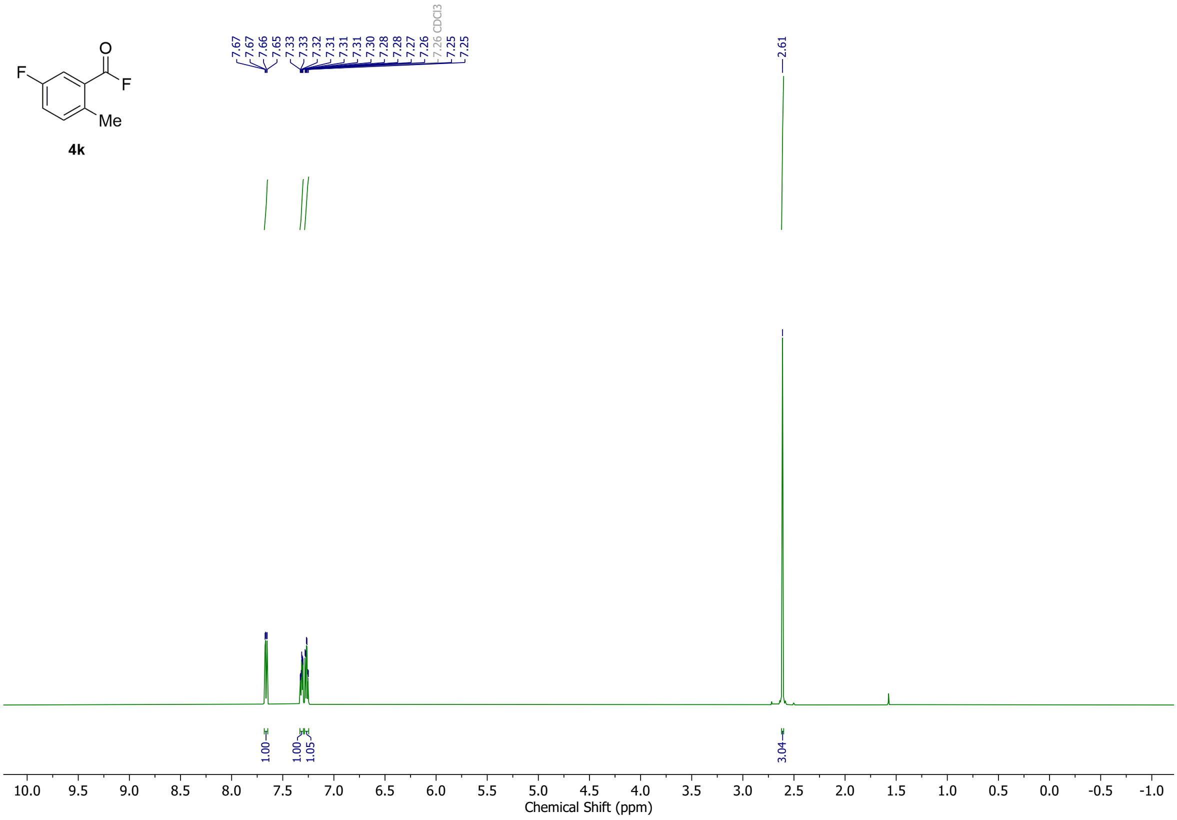
F
O
F
Me
4k
7.67 7.67 7.66 7.65 7.33 7.33 7.32 7.31 7.31 7.31 7.30 7.28 7.28 7.27 7.26 CDCl3 7.25 7.25
2.61
1.00
1.00
1.05
3.04
10.0 9.5 9.0 8.5 8.0 7.5 7.0 6.5 6.0 5.5 5.0 4.5 4.0 3.5 3.0 2.5 2.0 1.5 1.0 0.5 0.0 -0.5 -1.0
Chemical Shift (ppm)

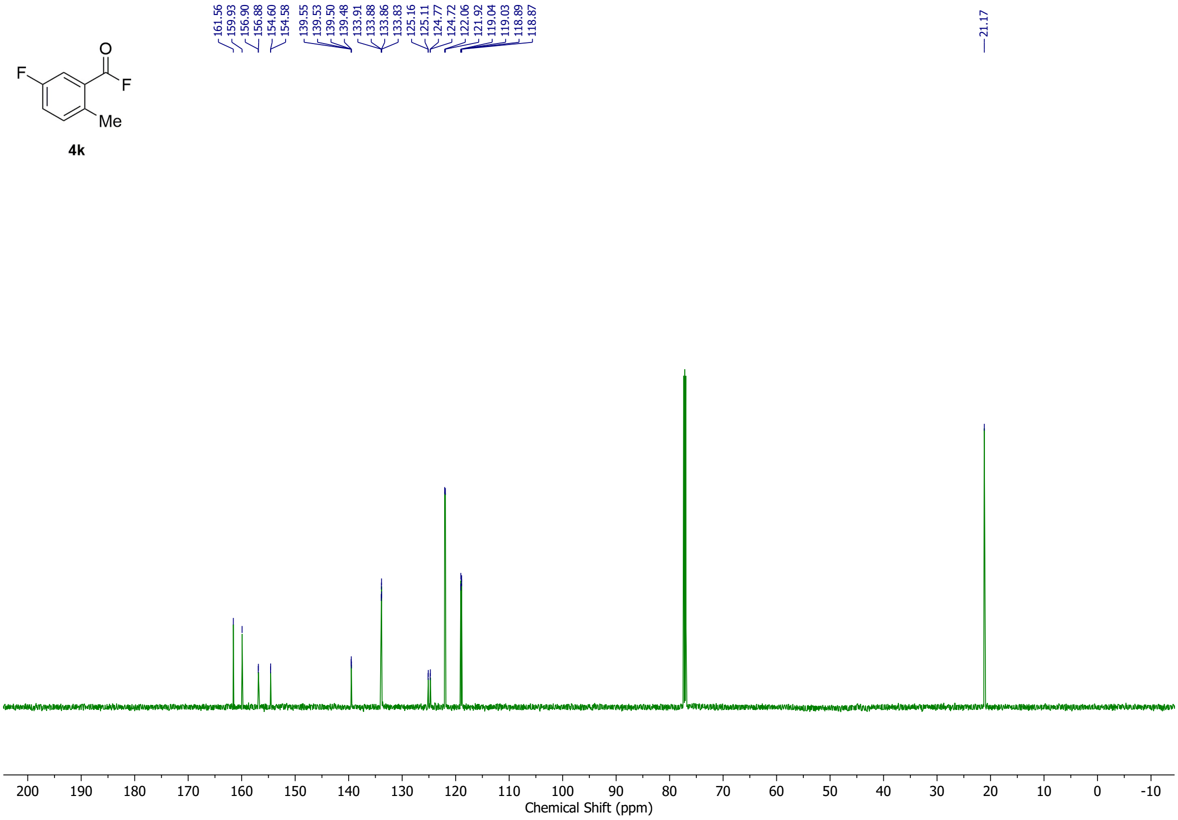

F
O
F
Me
4k
161.56
159.93
156.90
156.88
154.60
154.58
139.55
139.53
139.50
139.48
133.91
133.88
133.86
133.83
125.16
125.11
124.77
124.72
122.06
121.92
119.04
119.03
118.89
118.87
21.17
200 190 180 170 160 150 140 130 120 110 100 90 80 70 60 50 40 30 20 10 0 -10
Chemical Shift (ppm)

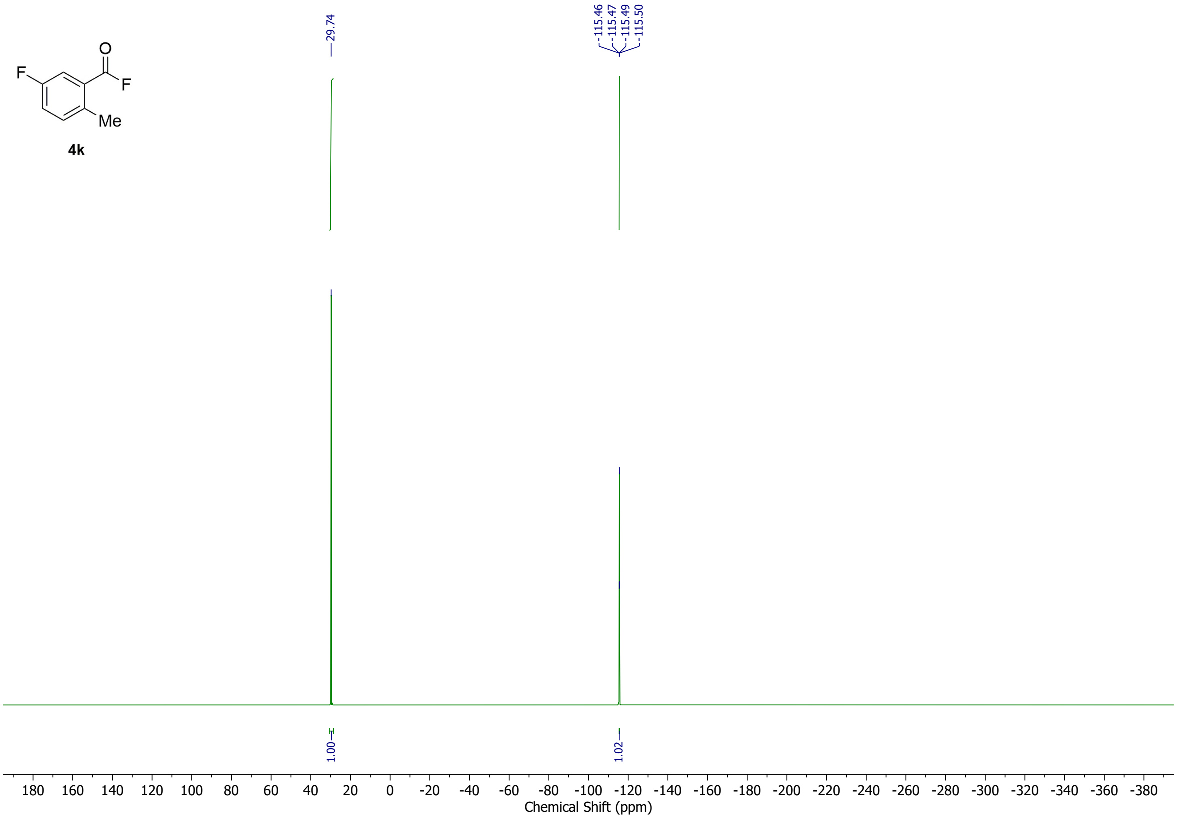

F
O
F
Me
4k
29.74
-115.46
-115.47
-115.49
-115.50
1.00
1.02
180 160 140 120 100 80 60 40 20 0 -20 -40 -60 -80 -100 -120 -140 -160 -180 -200 -220 -240 -260 -280 -300 -320 -340 -360 -380
Chemical Shift (ppm)

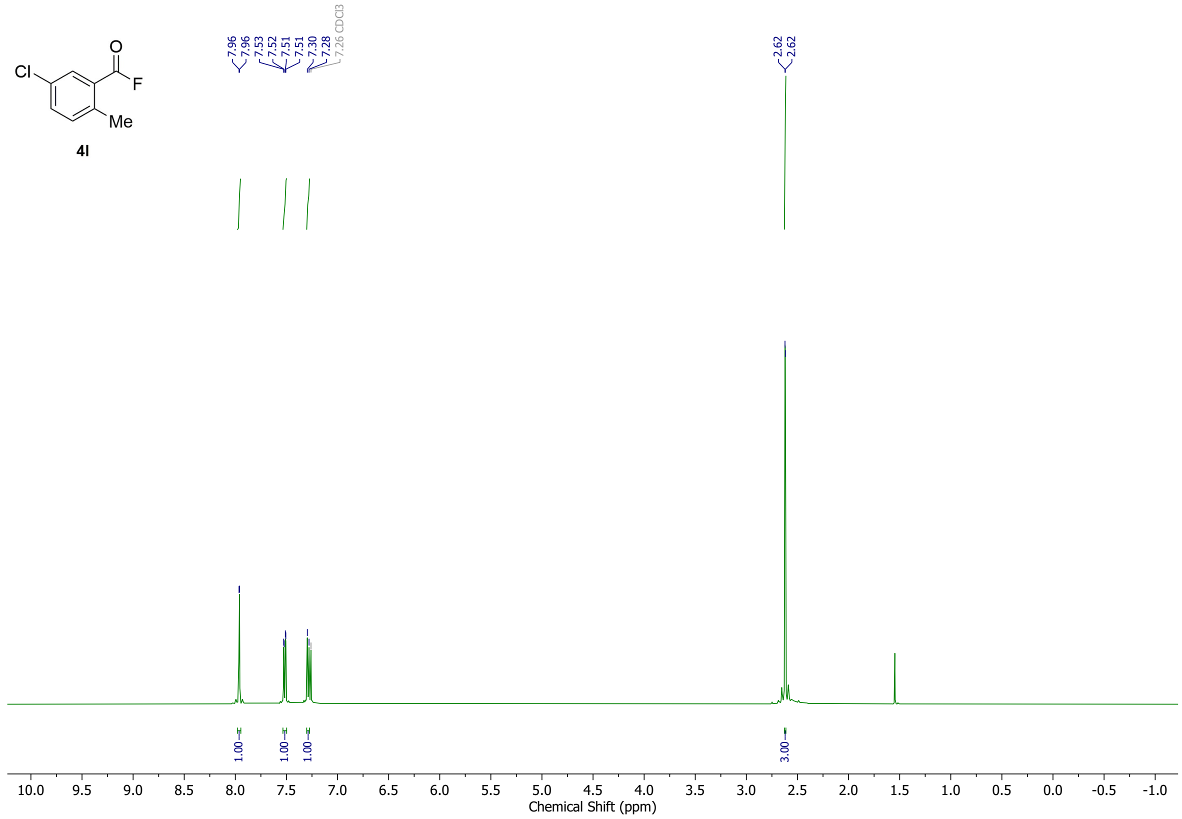
7.96
7.96
7.53
7.52
7.51
7.51
7.30
7.28
7.26 CDCl3
2.62
2.62
1.00
1.00
1.00
3.00
Chemical Shift (ppm)

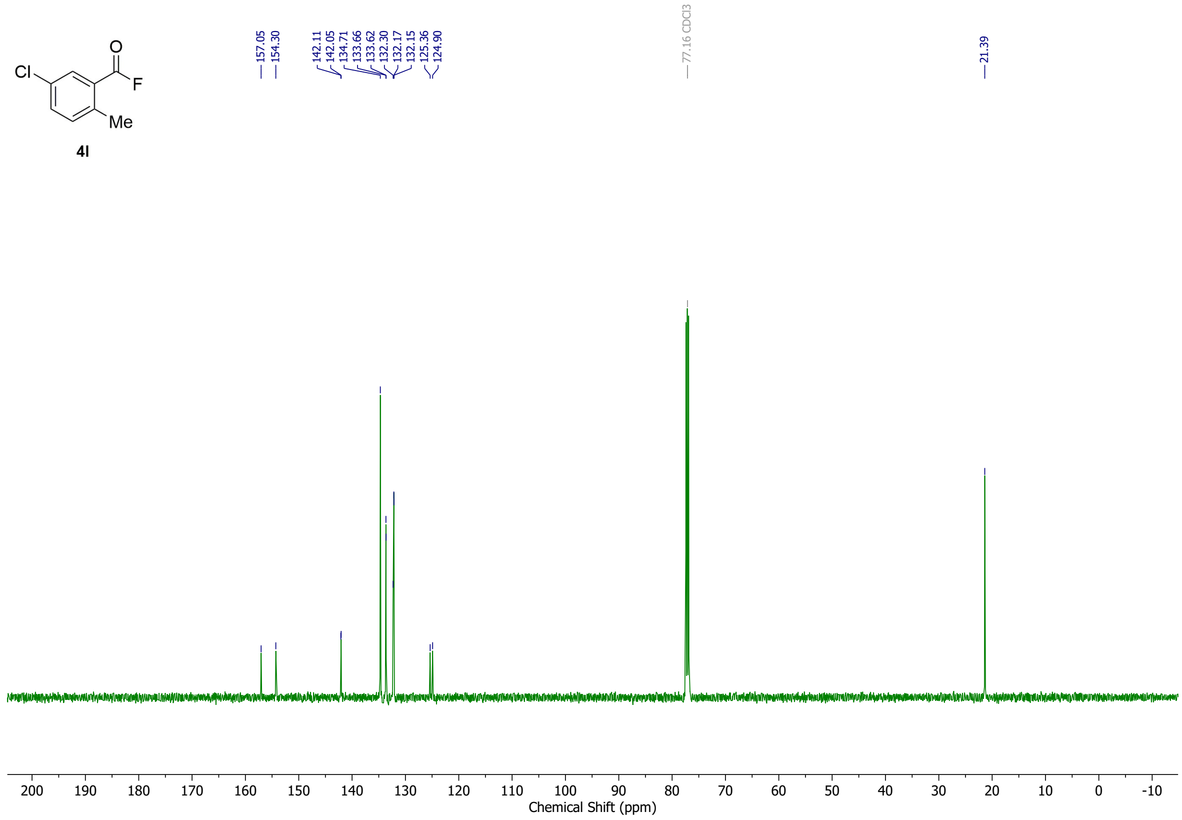
157.05
154.30
142.11
142.05
134.71
133.66
133.62
132.30
132.17
132.15
125.36
124.90
77.16 CDCl3
21.39
Chemical Shift (ppm)

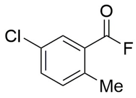

Cl
O
F
Me

4l

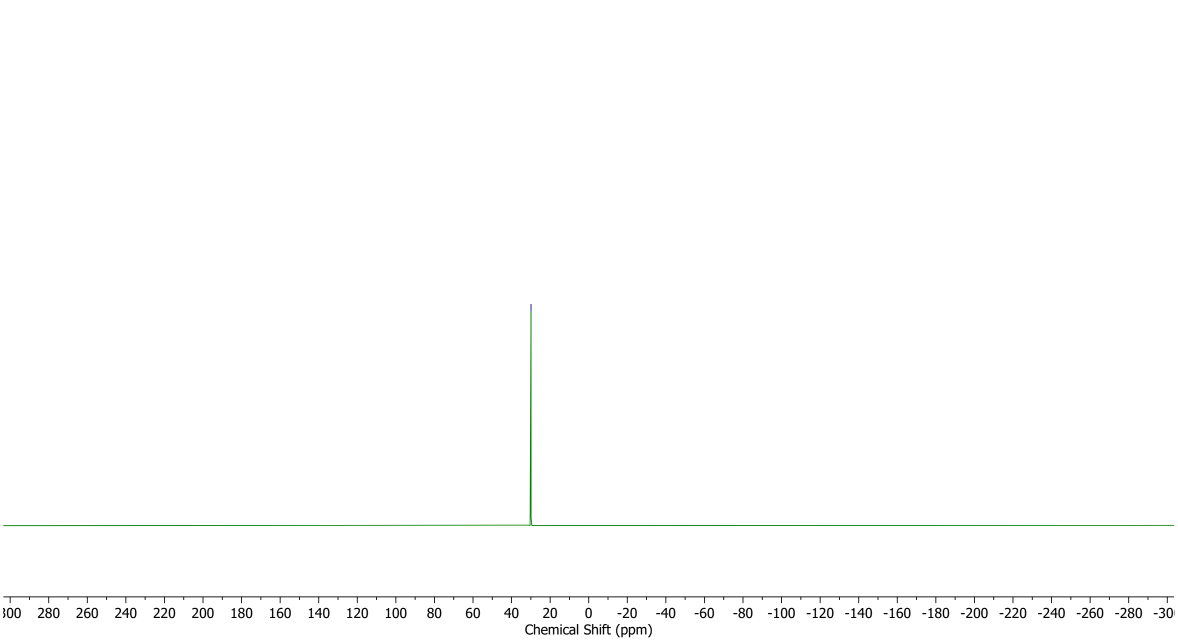

29.92
300 280 260 240 220 200 180 160 140 120 100 80 60 40 20 0 -20 -40 -60 -80 -100 -120 -140 -160 -180 -200 -220 -240 -260 -280 -300
Chemical Shift (ppm)

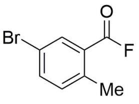

Br
O
F
Me

4m

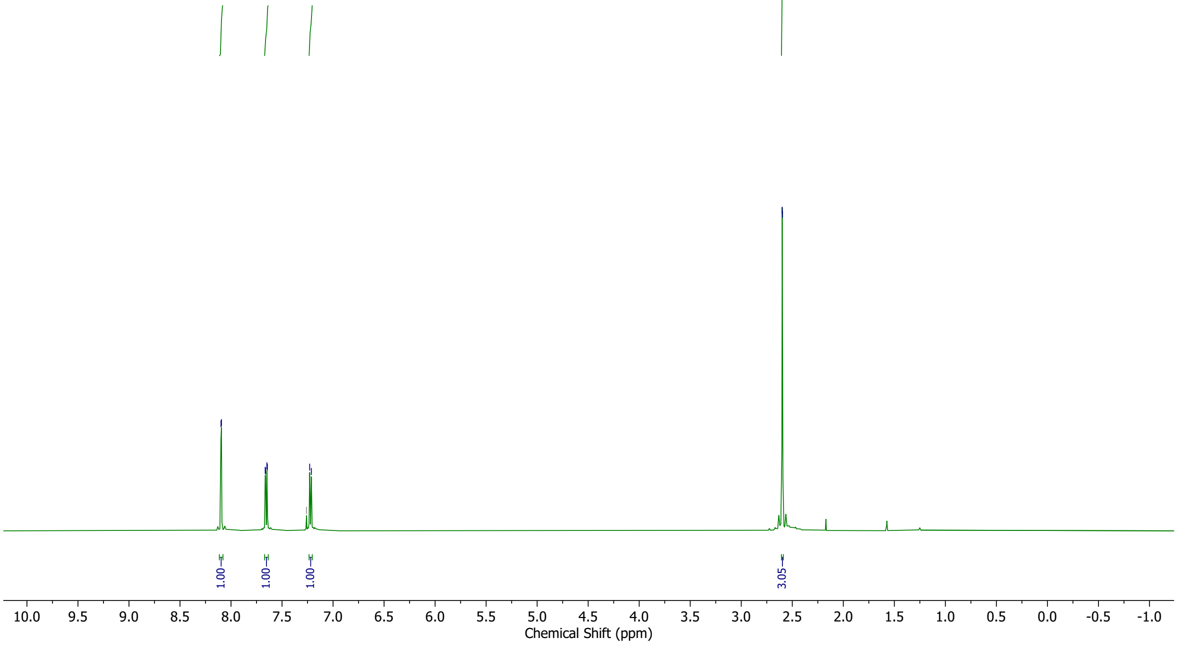

8.10
8.09
7.66
7.66
7.65
7.64
7.26 CDCl3
7.23
7.21
2.60
2.60
1.00
1.00
1.00
3.05
10.0 9.5 9.0 8.5 8.0 7.5 7.0 6.5 6.0 5.5 5.0 4.5 4.0 3.5 3.0 2.5 2.0 1.5 1.0 0.5 0.0 -0.5 -1.0
Chemical Shift (ppm)

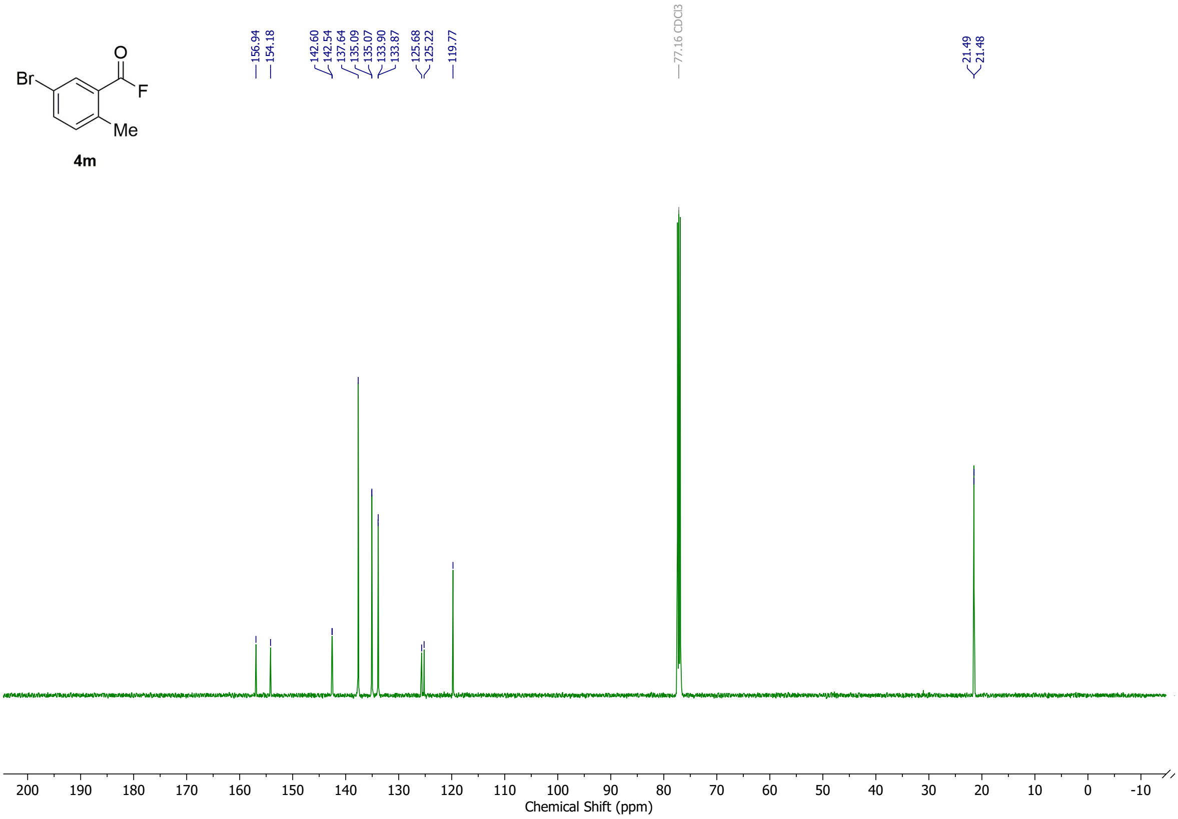
Br
O
F
Me
156.94
154.18
142.60
142.54
137.64
135.09
135.07
133.90
133.87
125.68
125.22
119.77
77.16 CDCl3
21.49
21.48
Chemical Shift (ppm)

4m

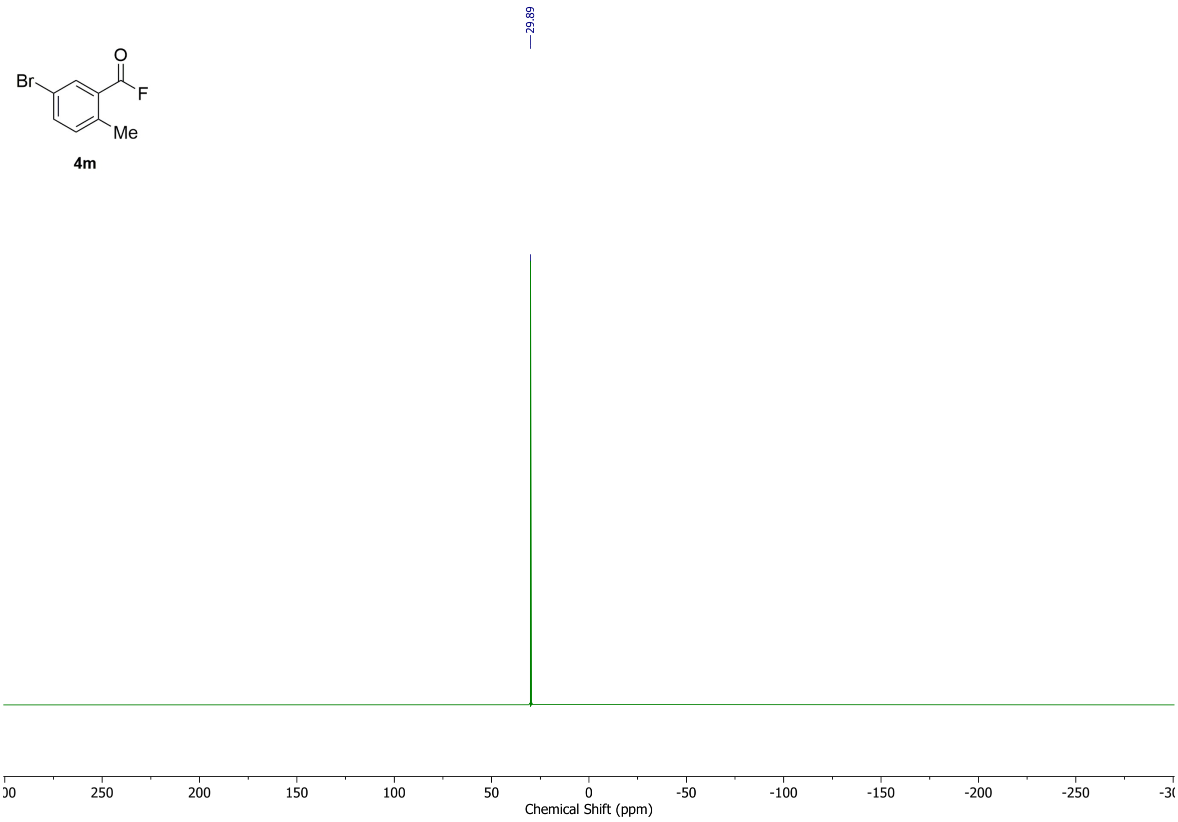
Br
O
F
Me
29.89
Chemical Shift (ppm)

4m

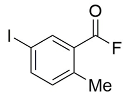

4n

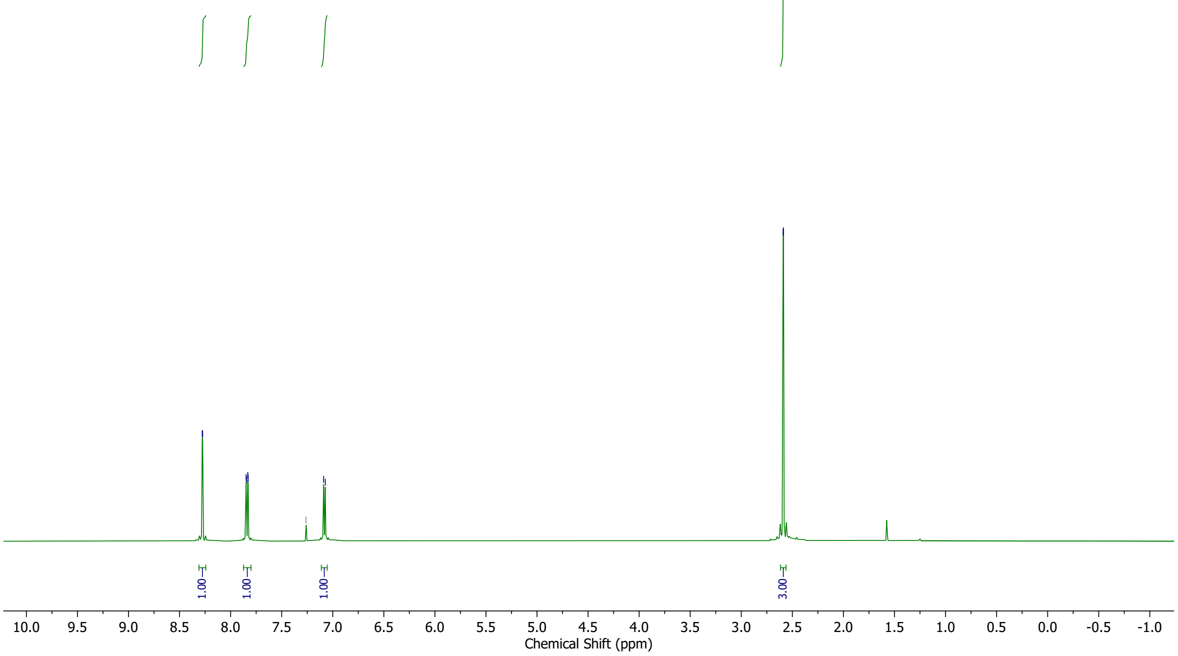

8.28
8.27
7.85
7.84
7.83
7.83
7.26 CDCl3
7.09
7.07
2.59
2.59
1.00
1.00
1.00
3.00
Chemical Shift (ppm)

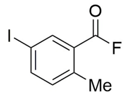

4n

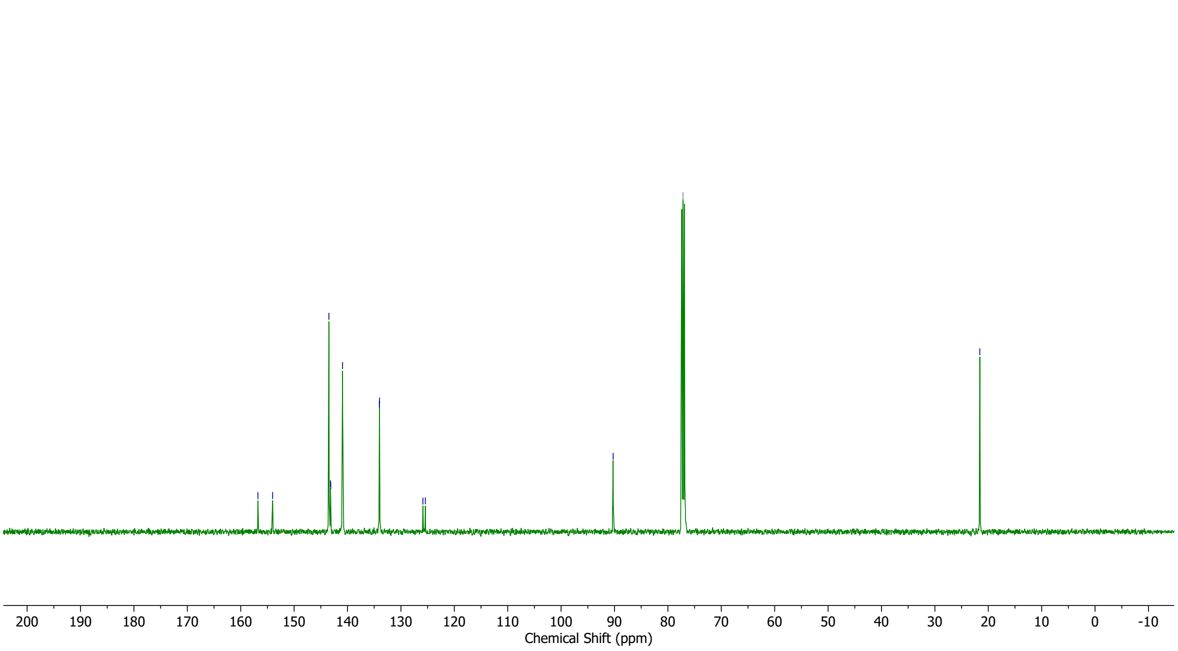

156.78
154.02
143.49
143.19
143.14
140.93
134.04
134.01
125.88
125.43
90.25
77.16 CDCl3
21.59
Chemical Shift (ppm)

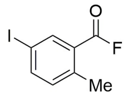

4n

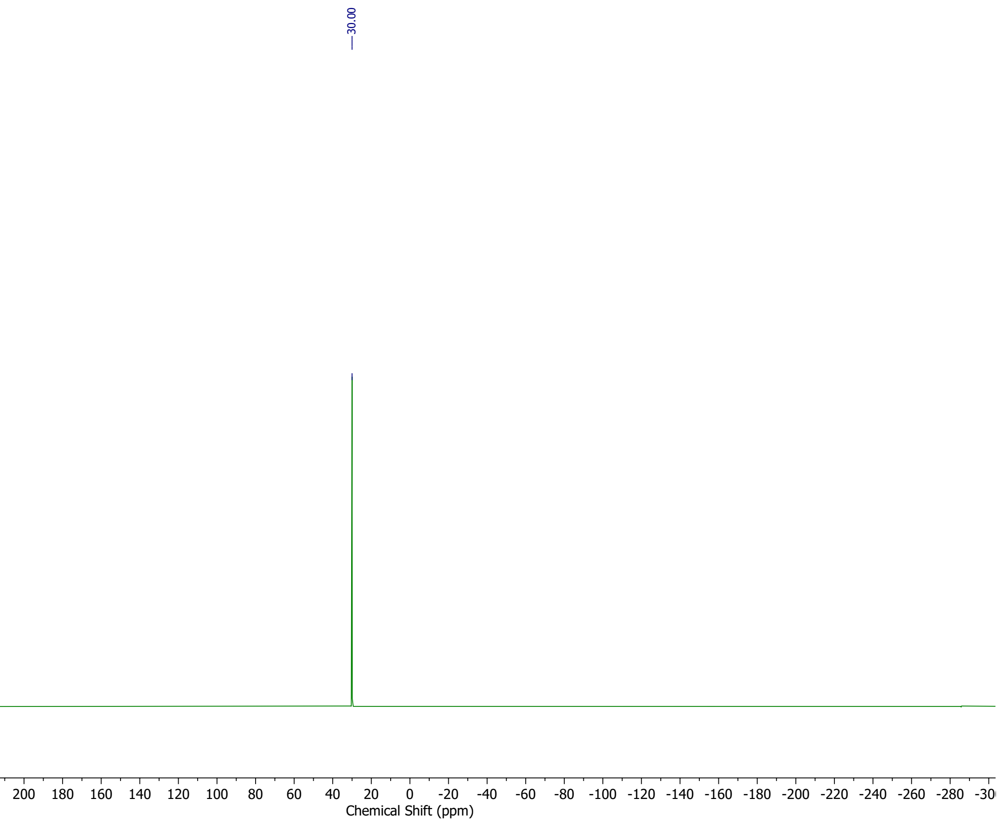

30.00
300 280 260 240 220 200 180 160 140 120 100 80 60 40 20 0 -20 -40 -60 -80 -100 -120 -140 -160 -180 -200 -220 -240 -260 -280 -300
Chemical Shift (ppm)

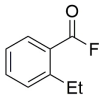

4o

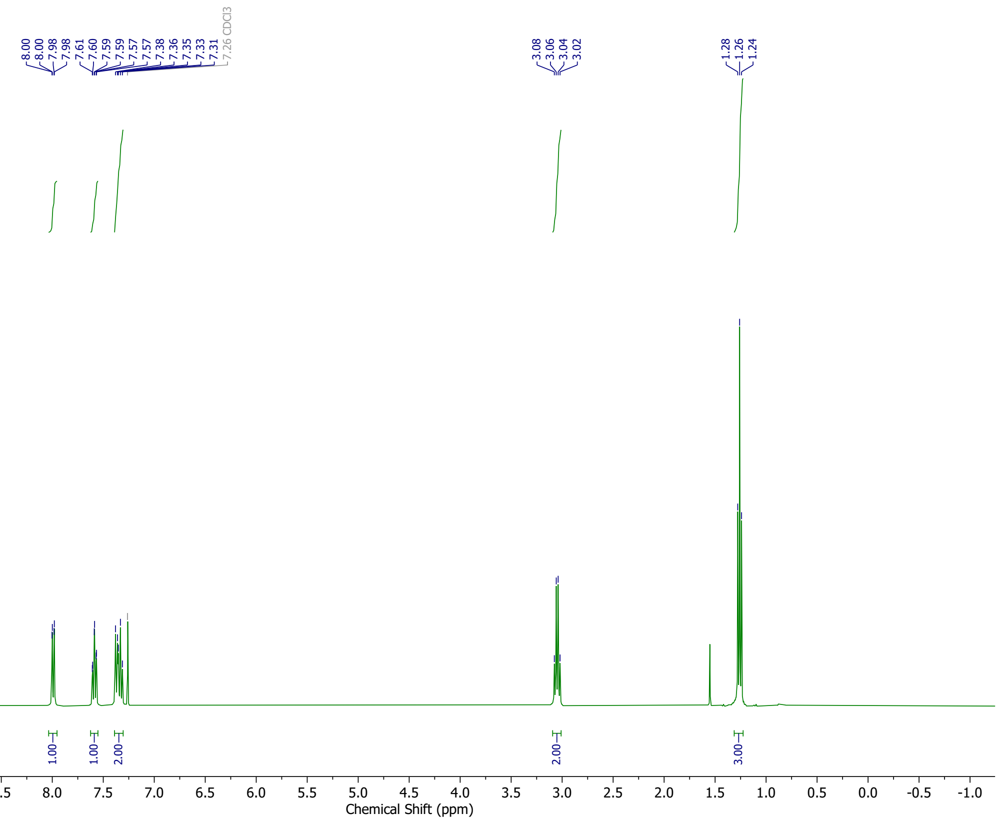

8.00
8.00
7.98
7.98
7.61
7.60
7.59
7.59
7.57
7.57
7.38
7.36
7.35
7.33
7.31
7.26 CDCl3
3.08
3.06
3.04
3.02
1.28
1.26
1.24
1.00
1.00
2.00
2.00
3.00
10.0 9.5 9.0 8.5 8.0 7.5 7.0 6.5 6.0 5.5 5.0 4.5 4.0 3.5 3.0 2.5 2.0 1.5 1.0 0.5 0.0 -0.5 -1.0
Chemical Shift (ppm)

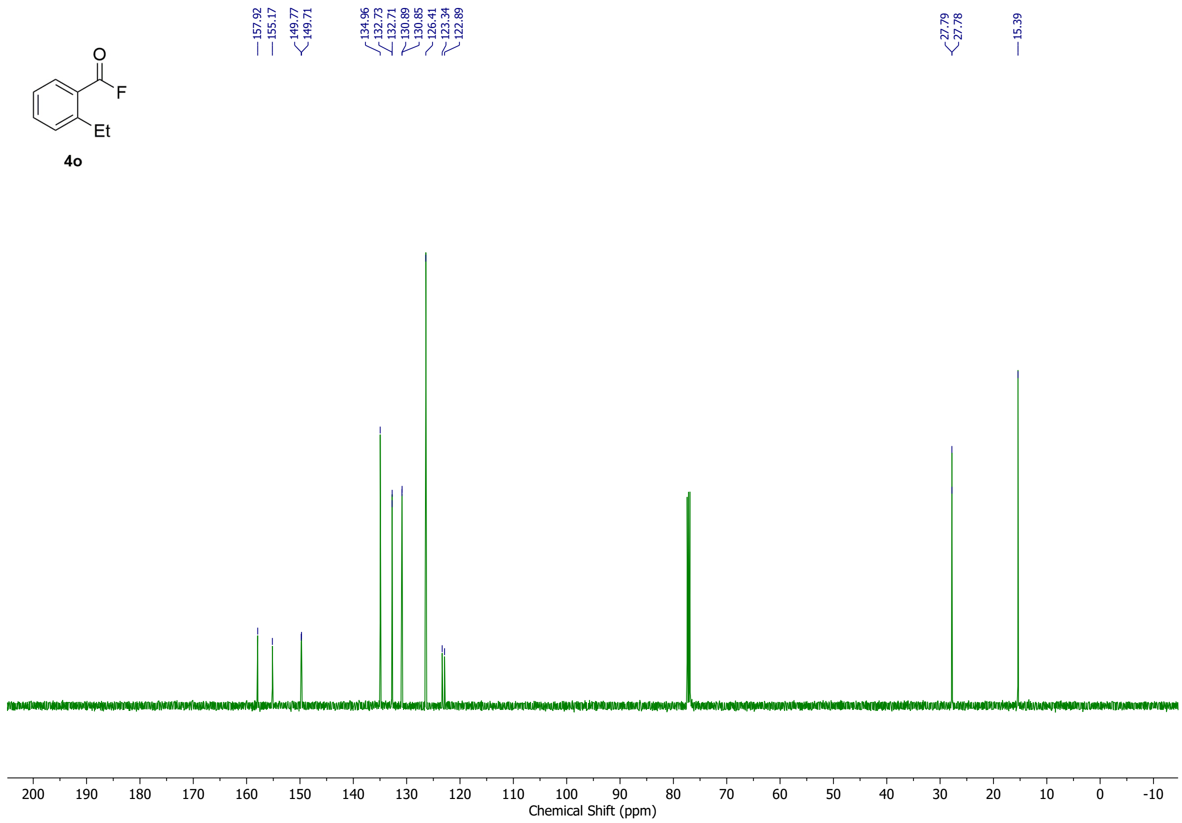

157.92
155.17
149.77
149.71
134.96
132.73
132.71
130.89
130.85
126.41
123.34
122.89
27.79
27.78
15.39
O
F
Et
4o
200 190 180 170 160 150 140 130 120 110 100 90 80 70 60 50 40 30 20 10 0 -10
Chemical Shift (ppm)

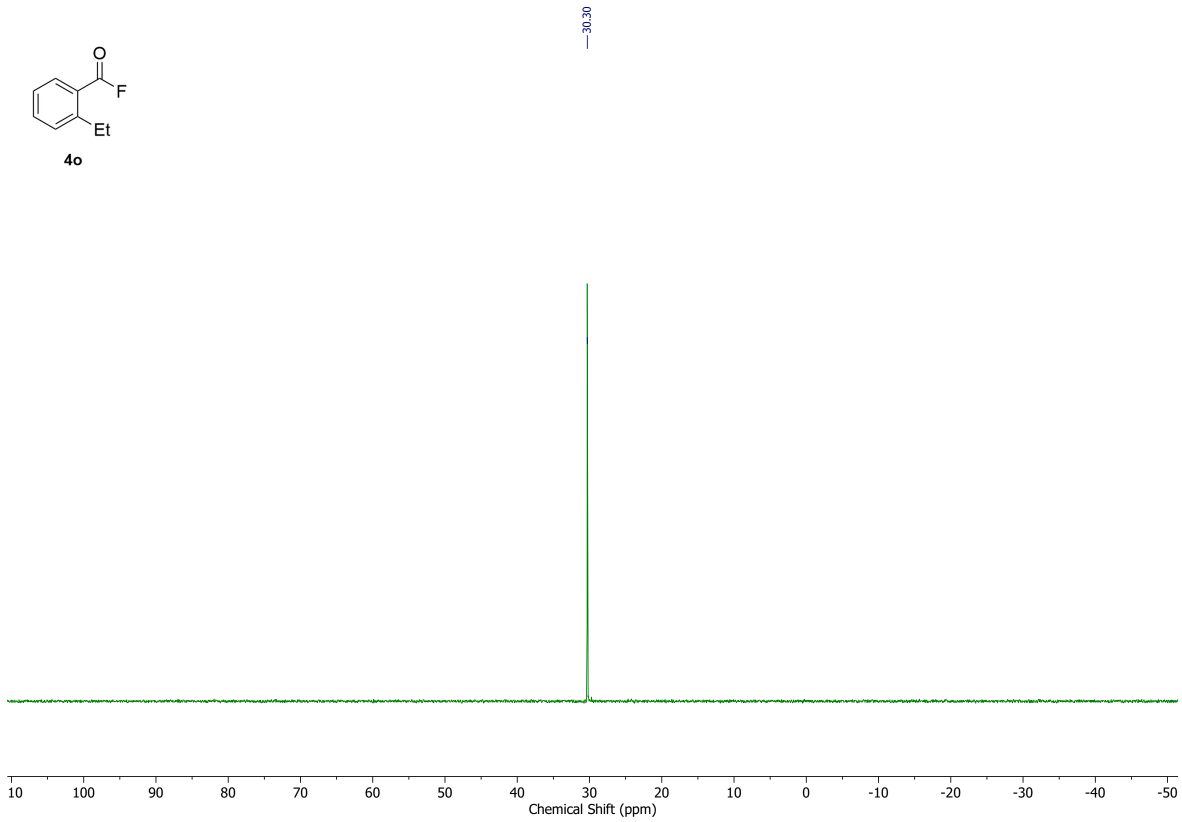

30.30
O
F
Et
4o
10 100 90 80 70 60 50 40 30 20 10 0 -10 -20 -30 -40 -50
Chemical Shift (ppm)

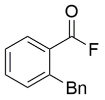
O
F
Bn

4p

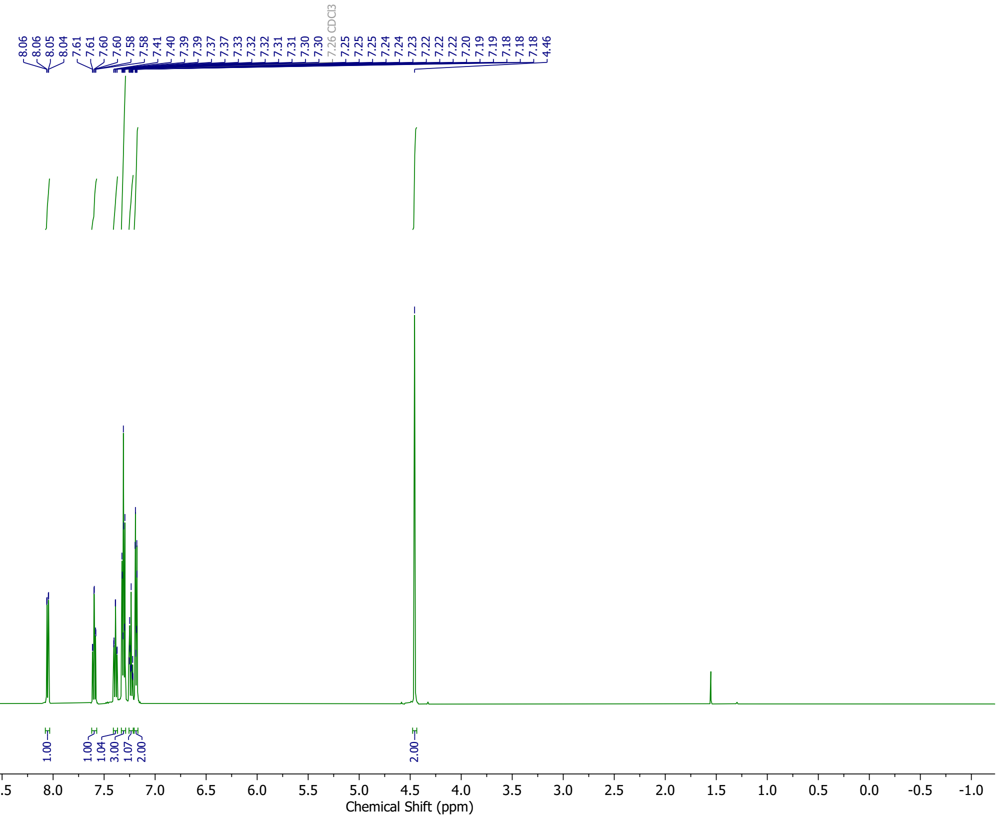
8.06 8.06 8.05 8.04 7.61 7.61 7.60 7.60 7.58 7.58 7.41 7.40 7.39 7.39 7.37 7.37 7.33 7.32 7.32 7.31 7.31 7.30 7.30 7.26 CDCl3 7.25 7.25 7.25 7.24 7.24 7.23 7.22 7.22 7.22 7.20 7.19 7.19 7.18 7.18 7.18 4.46
1.00 1.00 1.04 3.00 1.07 2.00 2.00
10.0 9.5 9.0 8.5 8.0 7.5 7.0 6.5 6.0 5.5 5.0 4.5 4.0 3.5 3.0 2.5 2.0 1.5 1.0 0.5 0.0 -0.5 -1.0
Chemical Shift (ppm)

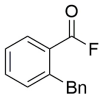
O
F
Bn

4p

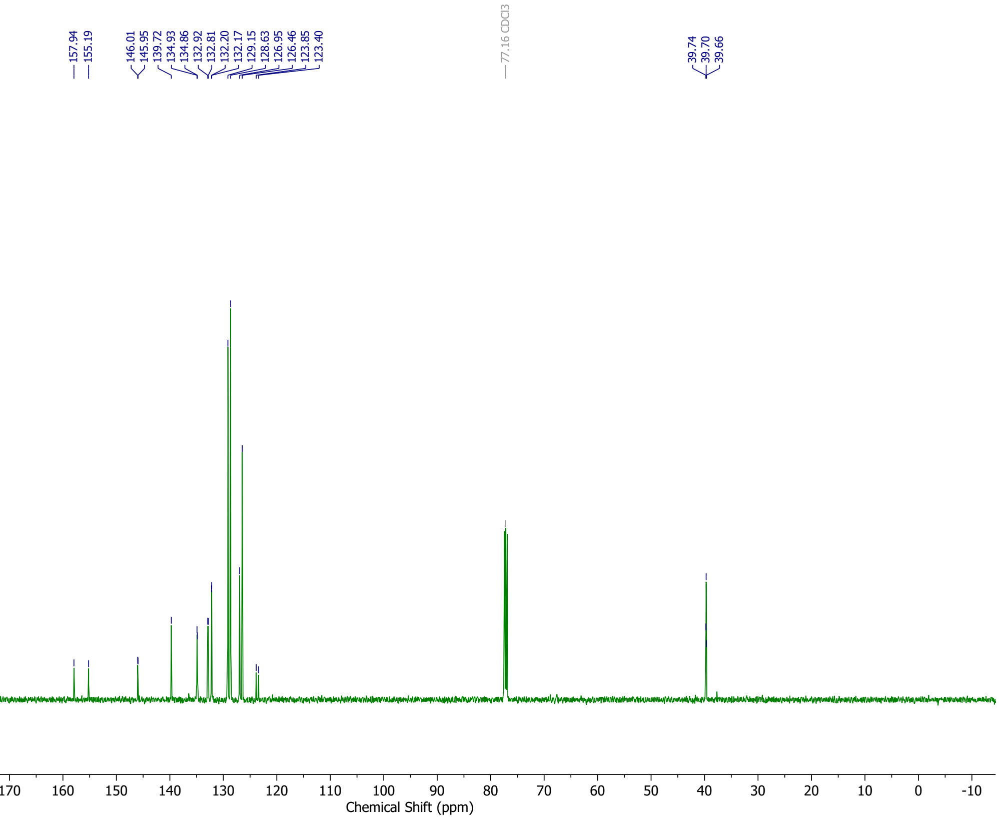
157.94 155.19 146.01 145.95 139.72 134.93 134.86 132.92 132.81 132.20 132.17 129.15 128.63 126.95 126.46 123.85 123.40
77.16 CDCl3
39.74 39.70 39.66
200 190 180 170 160 150 140 130 120 110 100 90 80 70 60 50 40 30 20 10 0 -10
Chemical Shift (ppm)

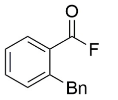

O
F
Bn

4p

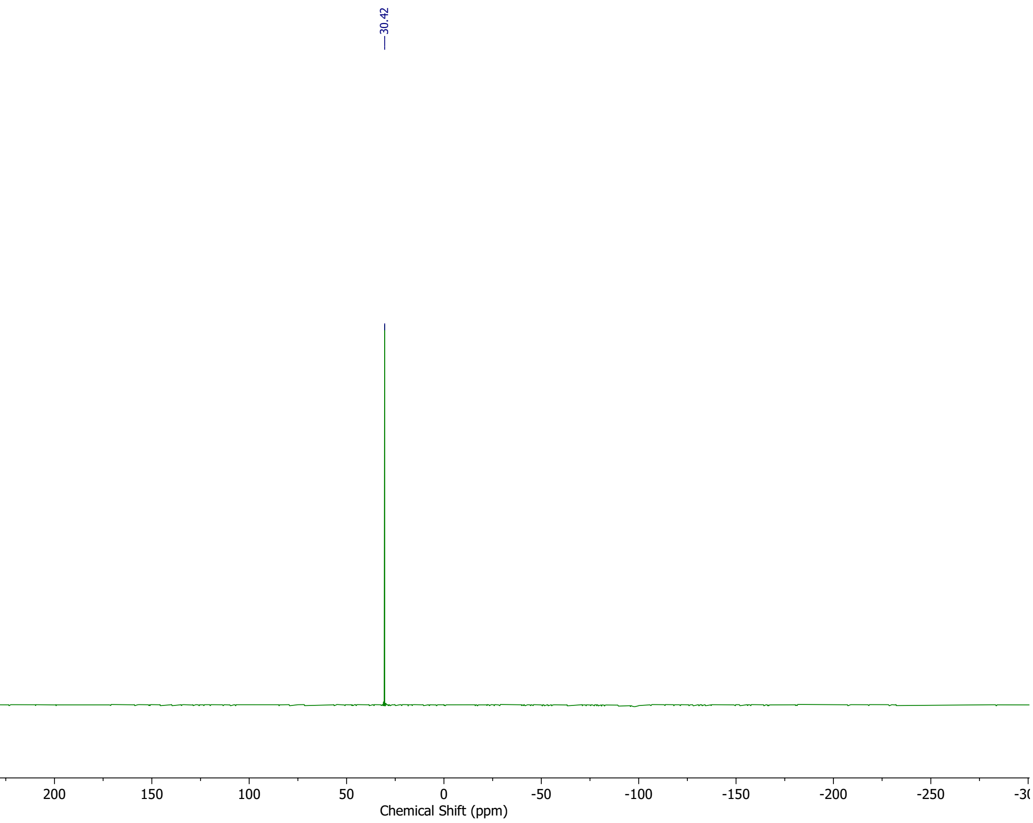

30.42
250
200
150
100
50
0
-50
-100
-150
-200
-250
Chemical Shift (ppm)

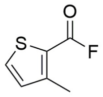

S
O
F

4q

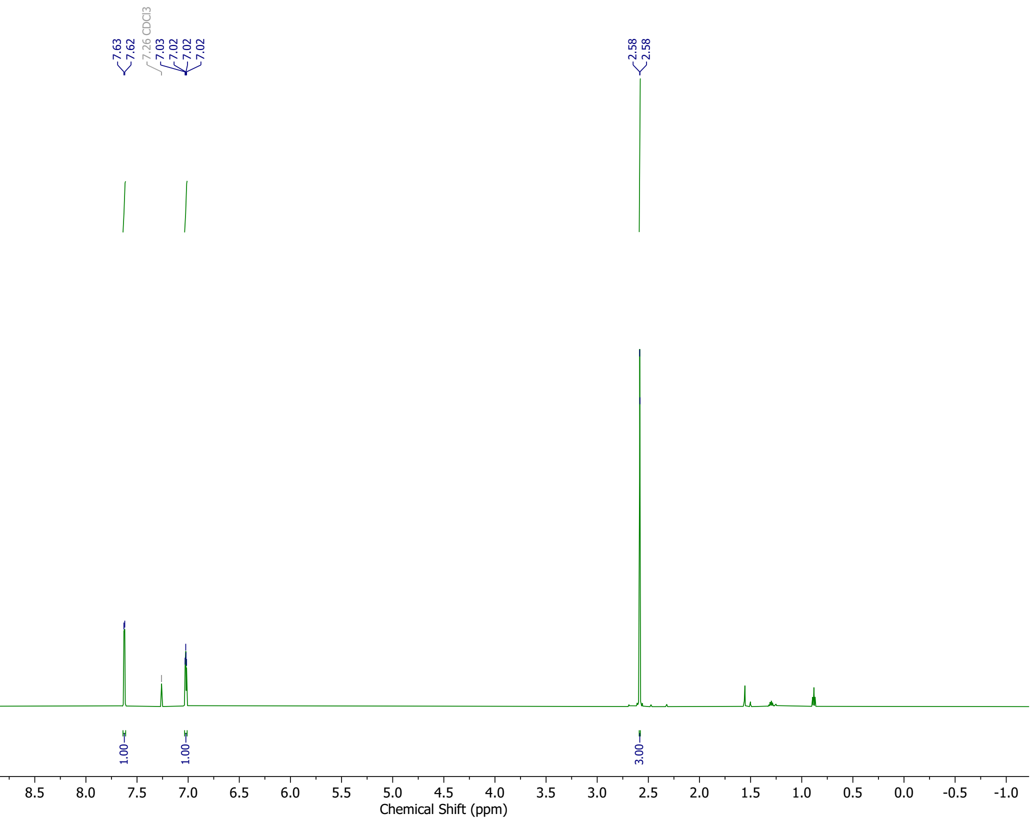

7.63
7.62
7.26 CDCl3
7.03
7.02
7.02
7.02
2.58
2.58
1.00
1.00
3.00
10.0
9.5
9.0
8.5
8.0
7.5
7.0
6.5
6.0
5.5
5.0
4.5
4.0
3.5
3.0
2.5
2.0
1.5
1.0
0.5
0.0
-0.5
-1.0
Chemical Shift (ppm)

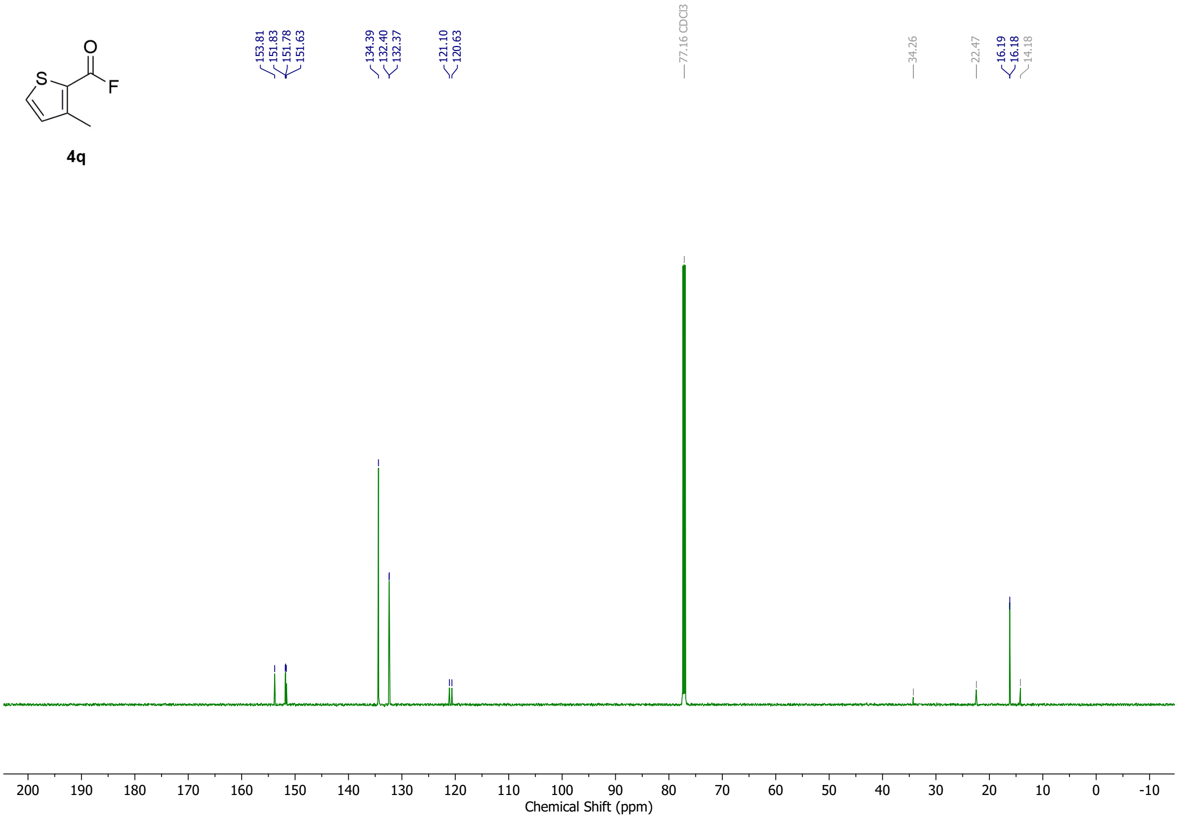

153.81
151.83
151.78
151.63
134.39
132.40
132.37
121.10
120.63
77.16 CDCl3
34.26
22.47
16.19
16.18
14.18
Chemical Shift (ppm)

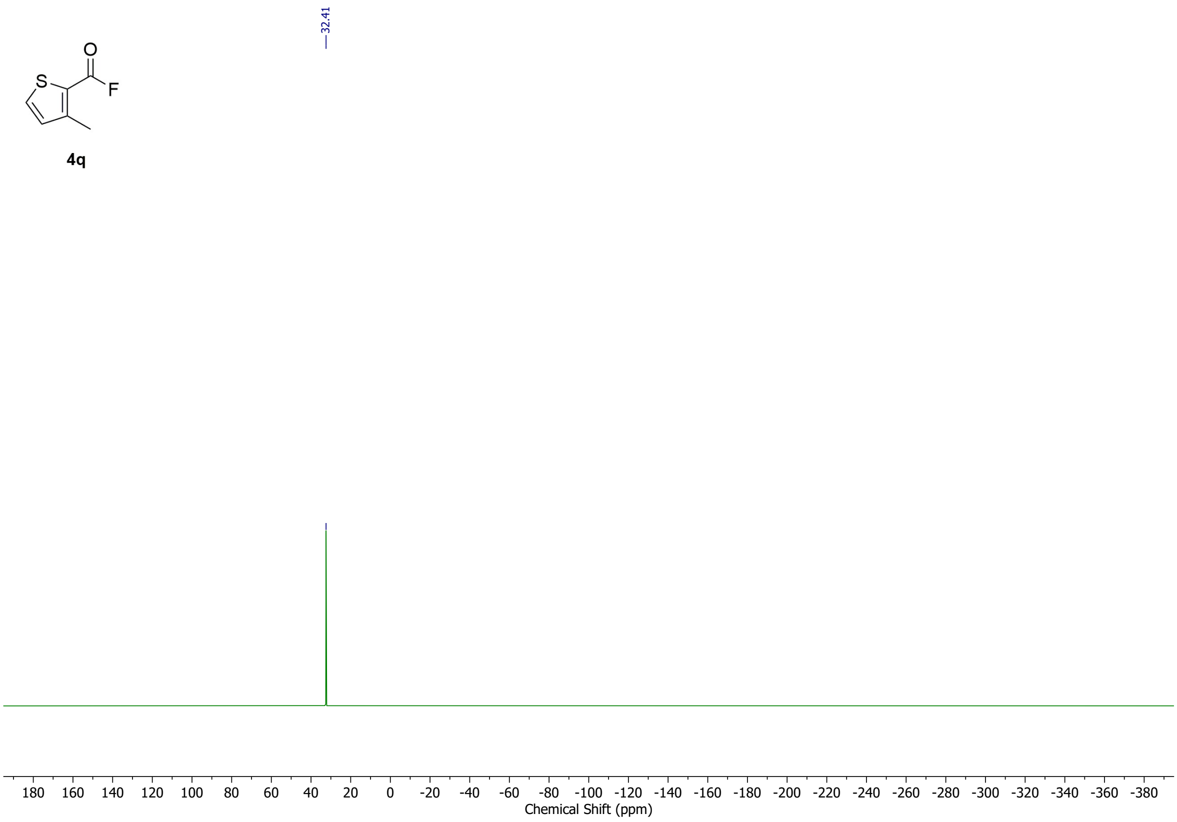

-32.41
Chemical Shift (ppm)
